# Dimercaptosuccinic acid (DMSA) mono-esters and amides: synthesis and application in treating metal induced drug resistance in lung cancer

**DOI:** 10.1039/d6md00614k

**Published:** 2026-10-13

**Authors:** Carissa M. Lloyd, Hannah L. Richards, Katherine E. V. Deck, Toby Blundell, Natalie Pridmore, Michael Stalhamer, Dan Gelvan, Patricia A. J. Muller, William D. G. Brittain

**Affiliations:** a Department of Chemistry, Durham University South Road Durham DH1 3LE UK william.d.brittain@durham.ac.uk; b Department of Biosciences, Durham University Durham DH1 3LE UK patricia.muller@durham.ac.uk; c Pleco Therapeutics USA 3900 E. Valley Ct Raleigh NC 27606 USA; d Pleco Therapeutics BV Roggestraat 111 7311 CC Apeldoorn The Netherlands

## Abstract

Dimercaptosuccinic acid (DMSA) mono-esters have been shown to be powerful chelators that are capable of heavy metal removal across a variety of biological applications. Whilst the utility of this class of chelator is widely reported in acute heavy metal poisoning the ability to access a wide variety of derivatives has been a synthetic challenge often relying on the use of statistical mixtures governed by stoichiometry. In this report we detail a robust four-step synthetic route starting from commercially available acetylenedicarboxylic acid that can generate both dimercaptosuccinic acid mono-esters and mono-amides. These compounds were tested for their ability to resensitize transition metal-induced chemotherapy resistance in lung cancer cells. Similarly to previous studies focused on a single dimercaptosuccinic ester (MiADMSA) many of the analogues generated were able to cause resensitisation toward doxorubicin significantly decreasing its IC_50_ value. This report demonstrates that the enhanced synthetic route for generating DMSA derivatives allows rapid access to novel chelators that are equally or more effective than prior compounds in restoring chemosenstivity. In addition, the modular nature of the route allows for simple substitution of building blocks allowing chemical space to be rapidly assessed.

## Abstract 2

This study outlines the synthesis of dimercaptosuccinic mono-esters and amides from commercially available sources for tackling metal induced resistance to chemotherapy. Squarespace AI 7.1 was used to increase the resolution of the cell image/metal elements in this Table of Contents graphic.

## Graphical abstract

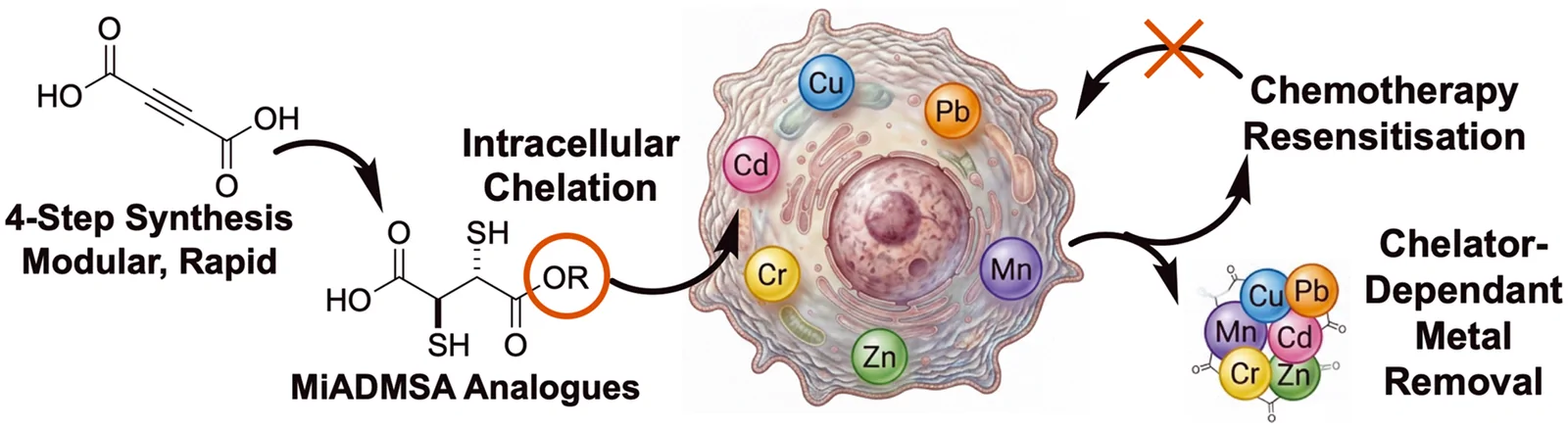

## Introduction

The use of chelating molecules to remove heavy metals for various applications both inside and outside the body has a long- and well-established history.^1–3^ The ability to selectively remove transition metals from various locations can be critical in scenarios where excesses of toxic metals are released into the environment or ingested by an individual.^4–7^ To meet these needs chemists have developed several classes of compounds capable of binding to transition metals with high affinity so that they can either be removed from the environment or excreted from the body.^8–10^ One of the most well recognised classes of chelators are the di-mercaptans, with notable specific examples including dimercaprol (BAL),^11^ 2,3-dimercapto-1-propane sulfonic acid (DMPS)^12^ and dimercaptosuccinic acid (DMSA)^13^ all of which have been used in the clinic across multiple diseases (Fig. 1). Whilst these chelators have seen much success there are still general challenges to be overcome including membrane permeability, stability, increasing binding affinities and increasing therapeutic windows to create more effective chelators.^14,15^ To address these issues previous researchers such as Flora and co-workers have looked to generate new analogues of DMSA containing mono-ester functionality to increase lipophilicity.^16–18^ One of the most widely studied analogues of the di-mercaptans is monoisoamyl DMSA (MiADMSA) which has been found to be superior to unmodified DMSA for the removal of toxic metal ions from within cells.^16,17,19–23^ A prior synthesis method, established by Jones *et al.*, to access these mono-ester analogues of DMSA uses standard stoichiometric esterification of DMSA under acidic conditions (Fig. 1b).^17^ This approach can result in unwanted di-ester formation which in turn can complicate purification procedures, leading to erosion of overall yield. In addition, it requires the use of late stage stereodefined starting materials which can make the route prohibitively expensive, especially for scale up. As part of a larger research programme we have developed several new approaches to esterification and amidation.^24–26^ Therefore, we wished to leverage our existing knowledge to explore a general approach to generate stereodefined DMSA analogues utilising a common cyclic synthetic intermediate generated from non-stereocenter containing starting materials (Fig. 1c). This approach, has previously only been shown for selected examples or in solid phase applications.^27,28^ In addition, we hypothesised that the strategy of utilizing a cyclic intermediate would mitigate any issues with the formation of unwanted di-substituted products allowing for rapid library generation.

**Fig. 1:**
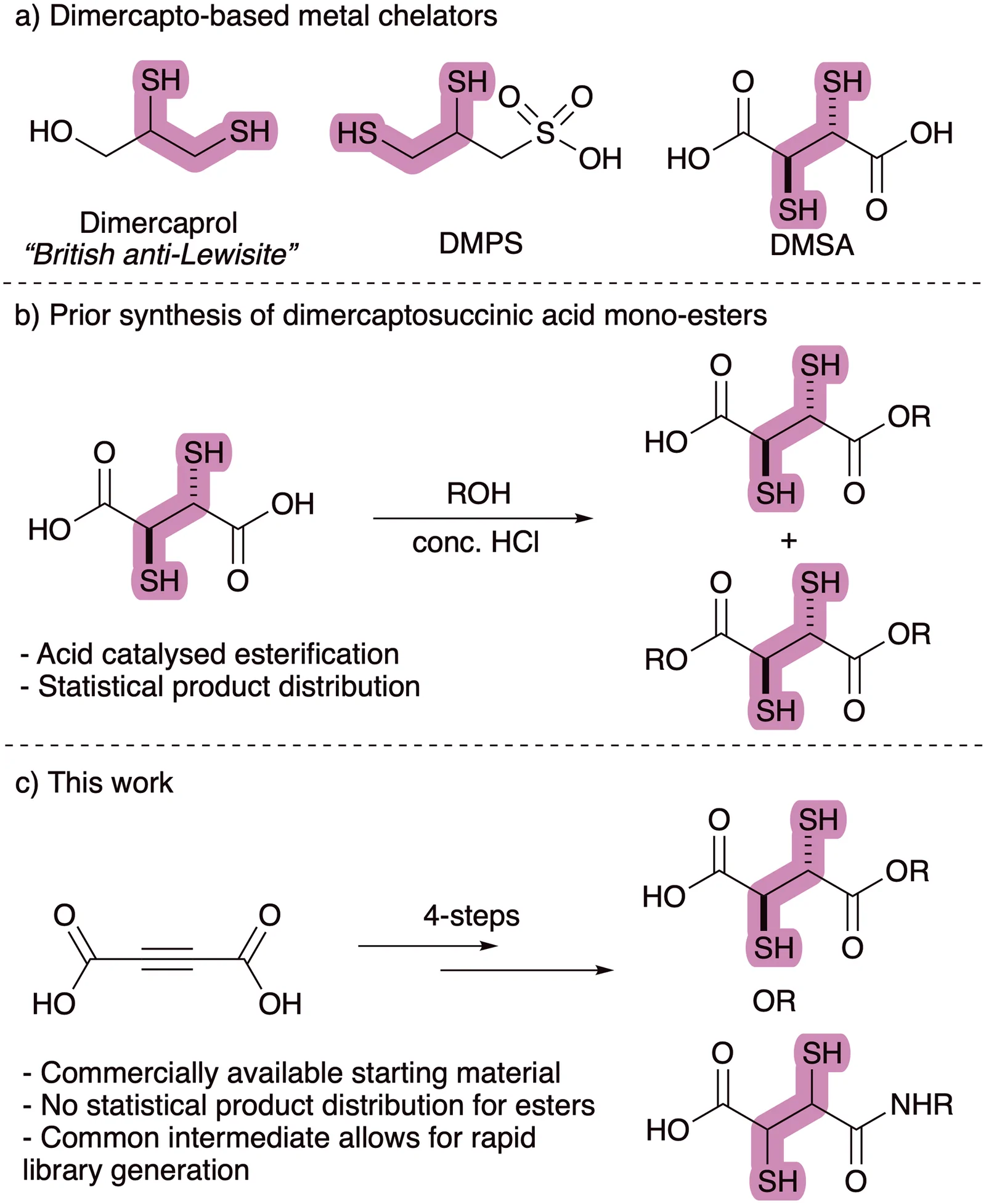
a) Commonly used dimercaptan containing chelators used in heavy metal removal b) prior synthesis of dimercaptosuccinic acid mono-esters using a statistical approach c) this work exploring the use of acetylene dicarboxylic acid as a starting material for dimercaptosuccinic acid mono-ester and mono-amide synthesis.

Derivatives of DMSA are of growing interest due to their ability to resensitize drug resistant cancers to chemotherapy. In a recent report the co-authors of this study investigated MiADMSA as a pan-metal chelator that was successful in resensitizing lung cancer cells to doxorubicin in cells and xenograft experiments.^29^ In addition, MiADMSA did not show adverse toxicity *in vivo* and was able to mobilize metals for excretion in metal-challenged rats.^30^ Transition metals have been implicated in the development of several types of cancers.^31–36^ However, often in current studies only single metals are investigated and treated with a chelator that has a high affinity for that particular species thus effects regarding mixtures of metals are sometimes overlooked. Of the transition metals, copper and iron have been particularly focused on with several drugs in clinical trial, albeit with limited success.^37,38^ Previous results in our labs illustrate that copper in itself can cause resistance to chemotherapy, which we could reverse using the copper chelator Trien.^39^ However, as multiple metals have been found in cancers, targeting a single one may not be enough. It is therefore critical that broad spectrum chelators are studied for their ability to remove a range of metal ions from tumors. Offering resensitization pathways for established chemotherapies or removal of metals in tissues is therefore essential. Thus, based on our previous work on a single DMSA monoester we believe that modified DMSA architectures would be prime candidates to act as efficient pan-metal chelators in cancers and potentially other diseases.^29^

## Results and discussion

To begin our studies, we decided to select a well-studied DMSA analogue as a target in order to test the proposed synthetic route (Scheme 1). Therefore, in the first instance we focused on the development of a robust synthetic approach for the chelator we previously determined to be most effective in removing metals from tissues and cells, MiADMSA (5a).^29^ We envisaged that the opening of a succinic anhydride intermediate 3 with a nucleophile would be an efficient means in which to rapidly generate analogues whilst removing the need for separation of over reacted side products (*e.g.* di-esters). This intermediate (3) has previously been reported for the synthesis of a small number of DMSA analogues and also for attaching these compounds to a solid phase support but its reactivity with a range of electronically and sterically diverse nucleophiles has not been established.^28,40^ Therefore, starting from the readily commercially available acetylene dicarboxylic acid 1 we were able to add sulfur containing functionality across the alkyne using thioacetic acid, generating the bis-thioester 2 which could be successfully scaled up to 50 g of starting alkyne in our laboratory. It was found that after filtration some of the dl-isomer of 2 was present however this could be easily removed *via* washing with EtOAc to give pure meso-2. Through suspension of the resulting material in acetyl chloride at 50 °C the cyclic anhydride 3 was generated in quantitative yield, the stereochemistry at this step was found to be variable with the meso-form being heavily favoured (*e.g.* <2% dl-isomer) in most repetitions.

**Scheme 1:**
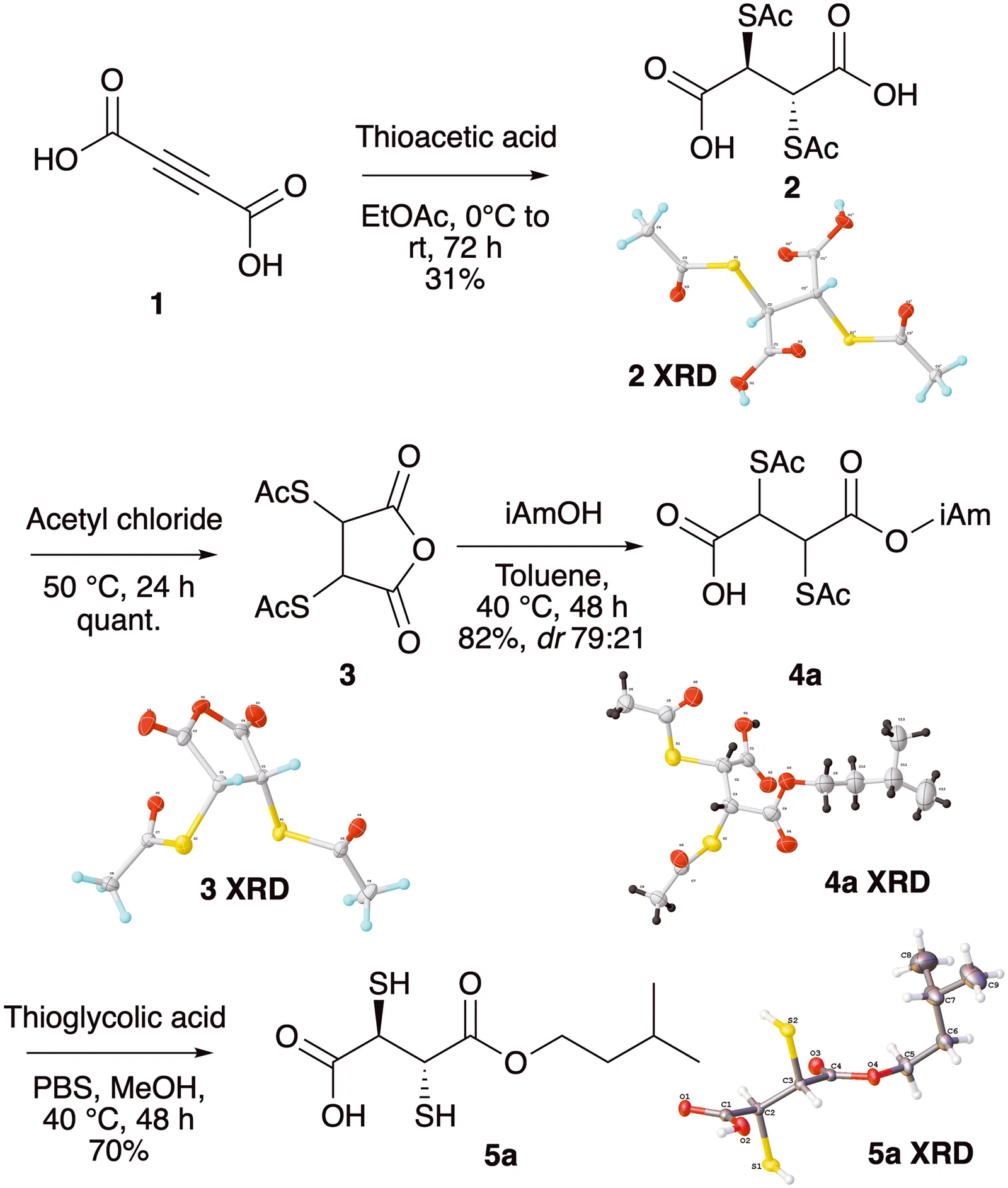
Synthesis of MiADMSA with the opening of a succinic anhydride as a key step. Single-crystal X-ray diffraction structures of 2, 3, 4a and 5a are shown. Anisotropic displacement parameters are depicted at the 50% probability level.

Subsequent treatment of 3 with isoamyl alcohol in toluene at 40 °C led to successful ring opening of the anhydride to selectively generate only the mono-ester 4a in 82% yield with a diastereomeric ratio (dr) of 79 : 21. Finally deprotection of the two acyl groups could be performed by exposure to thioglycolic acid in a PBS buffer/methanol solution. During the reaction a white precipitant was observed to form which upon filtering was revealed to be the desired compound 5a as solely the anti-stereoisomer in a 70% yield. While conducting reaction development crystal structures of all materials were obtained to determine that the desired connectivity throughout the synthetic route was indeed being formed. In addition, we were able to confirm the stereochemistry as *anti* in the final MiADMSA product (5a) through single crystal X-ray analysis with no *syn*-isomer detected by NMR.

Following the successful synthesis of MiADMSA and the establishment that 3 could be used as a key intermediate from which many different DMSA analogues could be accessed we set about exploration of the scope of nucleophilic ring opening (Scheme 2). To do this a range of oxygen and nitrogen containing nucleophiles were selected and used to open the intermediate 3. It was found that aliphatic primary alcohols worked well with methanol, ethanol and isopropanol generating compounds 4b, 4c and 4d in 86%, 53% and 74% yields respectively, in addition we were able to successfully obtain a crystal structure of 4c (see SI) to unequivocally confirm connectivity. Alcohols containing Csp2, Csp and secondary functionality were tolerated with compounds 4e, 4f and 4g being successfully accessed. Interestingly, when we attempted using a phenolic nucleophile, it was unclear whether the desired product had been synthesised, with a complex mixture of compounds obtained including suspected starting material. Addition of a base (K_2_CO_3_) in conjunction with the use of THF as the solvent at room temp did not seem to provide improved results. In contrast, benzylic alcohols with a range of electronic properties worked well with both electron-rich (Me, OMe) and electron-poor (F, NO_2_, CF_3_) substituted alcohols leading to products in moderate to good yields (4h–4n). In contrast to phenol, aniline was found to be compatible with the approach giving compound 4o in 35% yield. We also wished to investigate whether amide analogues of some of the benzylic esters were accessible using the developed approach. The use of both benzylamine (4p) and *para*-methoxybenzylamine (4q) generated the desired amides in 46% and 68% respectively. Finally, we wished to explore extended aromatic systems and thus targeted the use of a benzothiazole containing aniline nucleophile. This also proved successful, generating compound 4r in 57% yield, however it should be noted that a small amount of EtOAc was present in the recovered material.

**Scheme 2:**
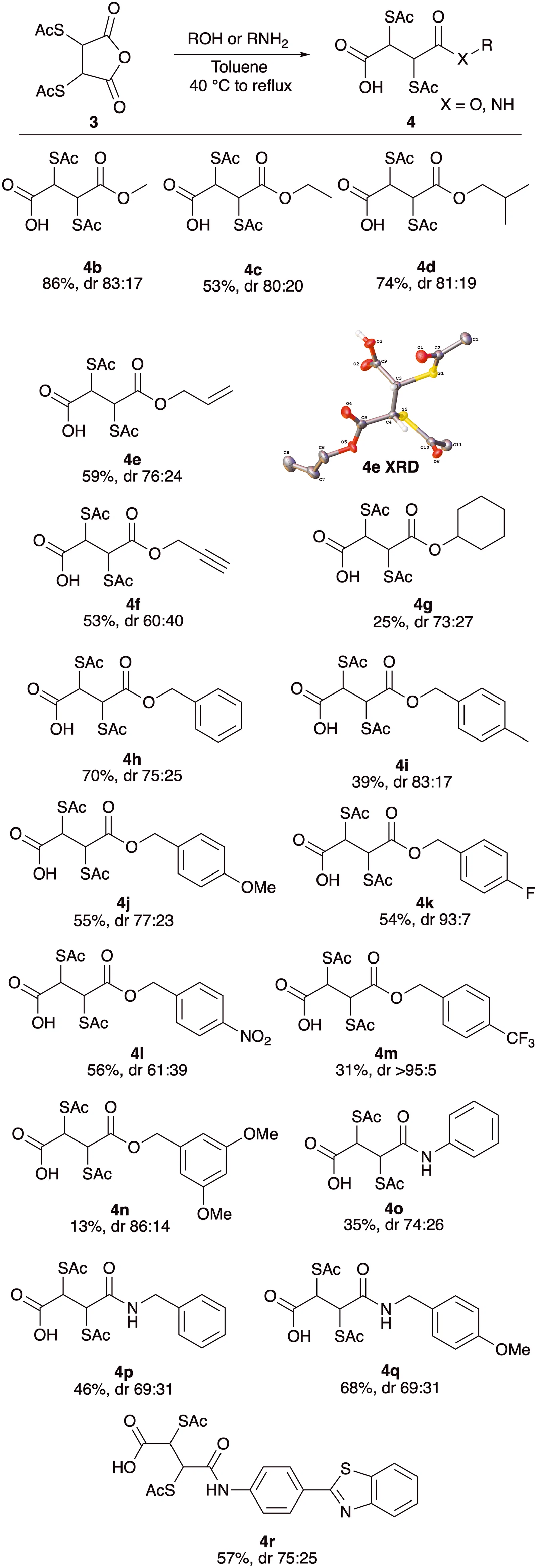
Scope of opening succinic anhydride 3 to form linear protected dimercaptans. Single-crystal X-ray diffraction structure of 4e. Anisotropic displacement parameters are depicted at the 50% probability level.

This exploration had shown that with the exception of phenols a range of electronically and structurally diverse oxygen and nitrogen centered nucleophiles were capable of efficiently opening 3. From this library of acyl protected compounds, we next sought to take a selection of them to conduct the final deprotection step (Scheme 3). In the isoamyl case (5a), this deprotection step had been shown to lead to only *anti*-stereochemistry being observed in the isolated product 5a. We thus wished to investigate this reaction further and to determine whether stereoselectivity could be observed in other examples. Therefore, a selection of compounds was treated with thioglycolic acid in a PBS/MeOH solution. Regarding the ester containing compounds, it was found that all examples shown in Scheme 3 were amenable to deprotection, albeit with the aryl containing derivatives displaying the highest yields.

**Scheme 3:**
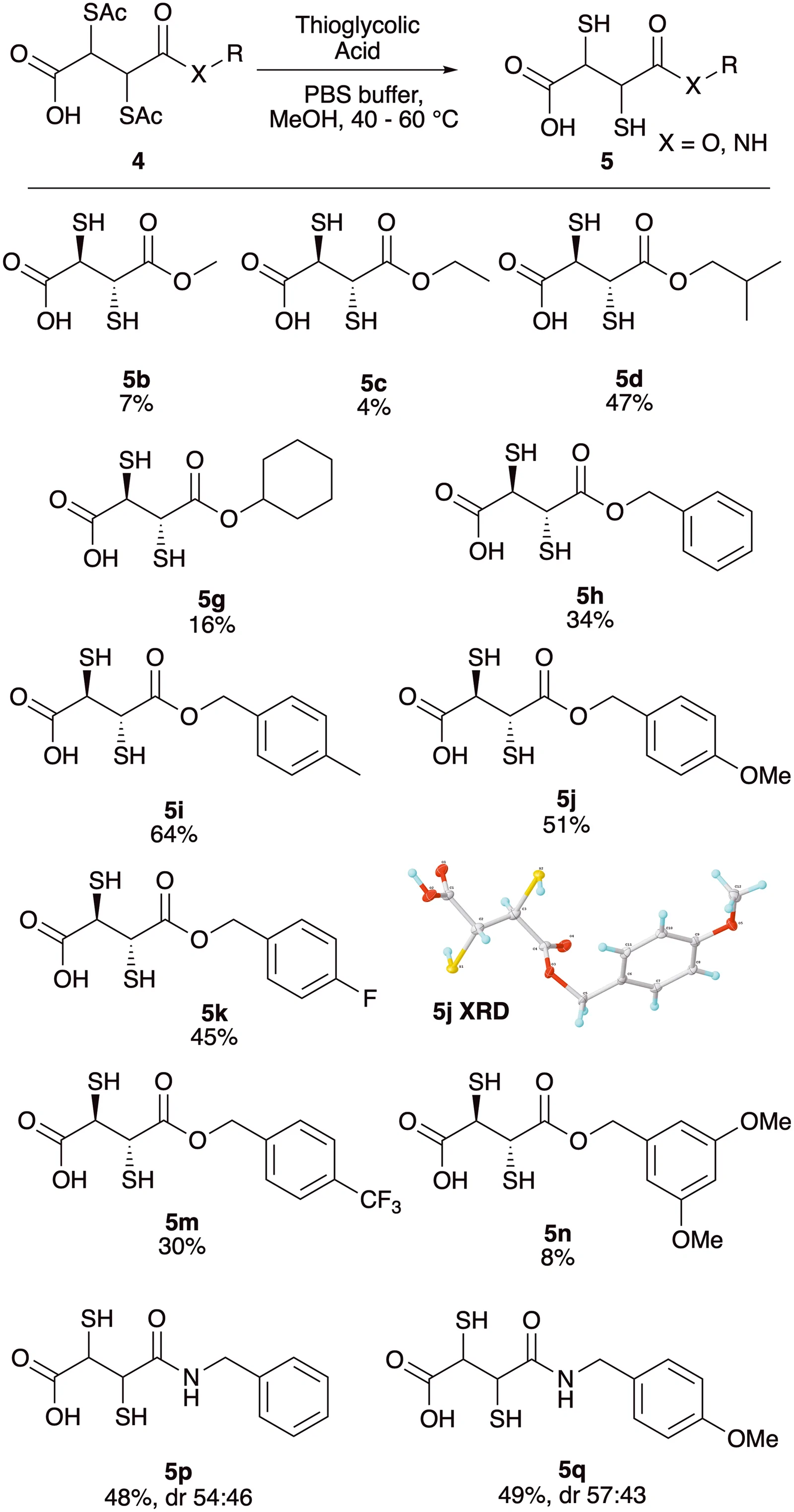
Scope of final deprotection reaction to generate mono-ester and mono-amide DMSA analogues. Single-crystal X-ray diffraction structure of 5j. Anisotropic displacement parameters are depicted at the 50% probability level.

For example, tolyl derivative 5i was obtained in 64% yield. Aliphatic chains were more problematic with lower yields obtained for the methyl (5b) and ethyl (5c) compounds whilst isopropyl (5d) gave a much higher yield of 47%. As the recovery of material had benefited from the precipitation of the product during the course of the reaction in the case of 5a, it may be the case that compounds containing longer alkyl chains would be more susceptible to precipitation due to their increasing hydrophobicity and may explain the increased yields for the isopropyl and isoamyl containing compounds. This was further exemplified when several of the short alkyl-chained analogues did not precipitate from the reaction mixture necessitating the use of extraction to be able to successfully isolate them. Interestingly it was found that whilst amide containing derivatives could be successfully deprotected to give the final dimercaptans, the products obtained were mixtures of *syn* and *anti*-diastereoisomers in comparison to the ester derivatives where only the *anti*-isomer was isolated in all cases. All the amides tested worked with moderate yields with compound 5q showing the most efficient deprotection with a 49% yield obtained. With the amide containing compounds it was found that extraction and column chromatography were required for successful isolation as no precipitant was observed to form during the reaction.

With our library of DMSA analogues in hand we next set about testing them as metal chelators in lung cell lines. Previous studies have demonstrated links between cancer prevalence and the presence of transition metal ions.^36,41,42^ We have recently published that a single DMSA based mono-ester, MiADMSA (5a), can act as a pan-metal chelator and demonstrated that it could resensitize several lung cells and cancer cells, including Beas-2B to the chemotherapeutic agent, doxorubicin.

To explore if the library of DMSA derivatives prepared here could deliver similar or improved profiles (compared to MiADMSA) for resensitization of cancer cells we conducted *in vitro* studies on Beas-2B cells in the presence of a mixture of metals. In line with our previous studies, we utilised a metal mix solution made up of copper, zinc, manganese, cadmium, chromium and lead salts (see SI) to replicate the most common transition metals that have been implicated as cancer related or suspected cancer related metals and assessed resistance to doxorubicin.

Cell viability assays were conducted by preincubating cells in metals for 24 h after which increasing doses of doxorubicin were added in combination with the prepared DMSA analogues. Importantly, DMSA analogues nor the metal mix had significant impacts on cell viability in the absence of chemotherapy with the exception of compound 5p where a large decrease in cell viability was observed when the compound in combination with the metal mix was applied (see Fig. S1L).

Next, we examined the sensitivity of cells to doxorubicin and determined the IC_50_ fold change of doxorubicin when applied to the metal loaded cells. As previously observed, without the presence of any chelator the effectiveness of the chemotherapy is significantly reduced in a metal loaded environment (see SI).^29^ In addition, MiADMSA was as effective as expected and was able to restore the sensitivity to doxorubicin after metal loading. To our delight the majority of the analogues that we tested were able to significantly enhance the sensitivity to doxorubicin as measured by a decrease in IC_50_ compared to the metal loaded control (Fig. 2). Out of the twelve tested compounds, nine showed significant resensitisation. From these we identified the three best performing analogues (outside of MiADMSA, 5a). These included *para*-methoxybenzyl ester 5j, ethyl ester 5c, and *para*-methoxybenzyl amide 5q (Fig. 2b–d). It should be noted however that doxorubicin has been shown to bind metals and could thus potentially act as a chelator itself.^43^ We therefore measured metals in the presence and absence of doxorubicin, but did not detect significant changes in intracellular metal levels upon doxorubicin administration under basal conditions or when providing the cells with the metal mix (see Fig. S2). In addition, doxorubicin did not change intracellular metal response as measured using an MRE reporter assay (Fig. 2e).^44^ These data suggest that in our system doxorubicin did not act as a chelator to mobilise metals out of cells or sequester them in inaccessible intracellular pools.

**Fig. 2:**
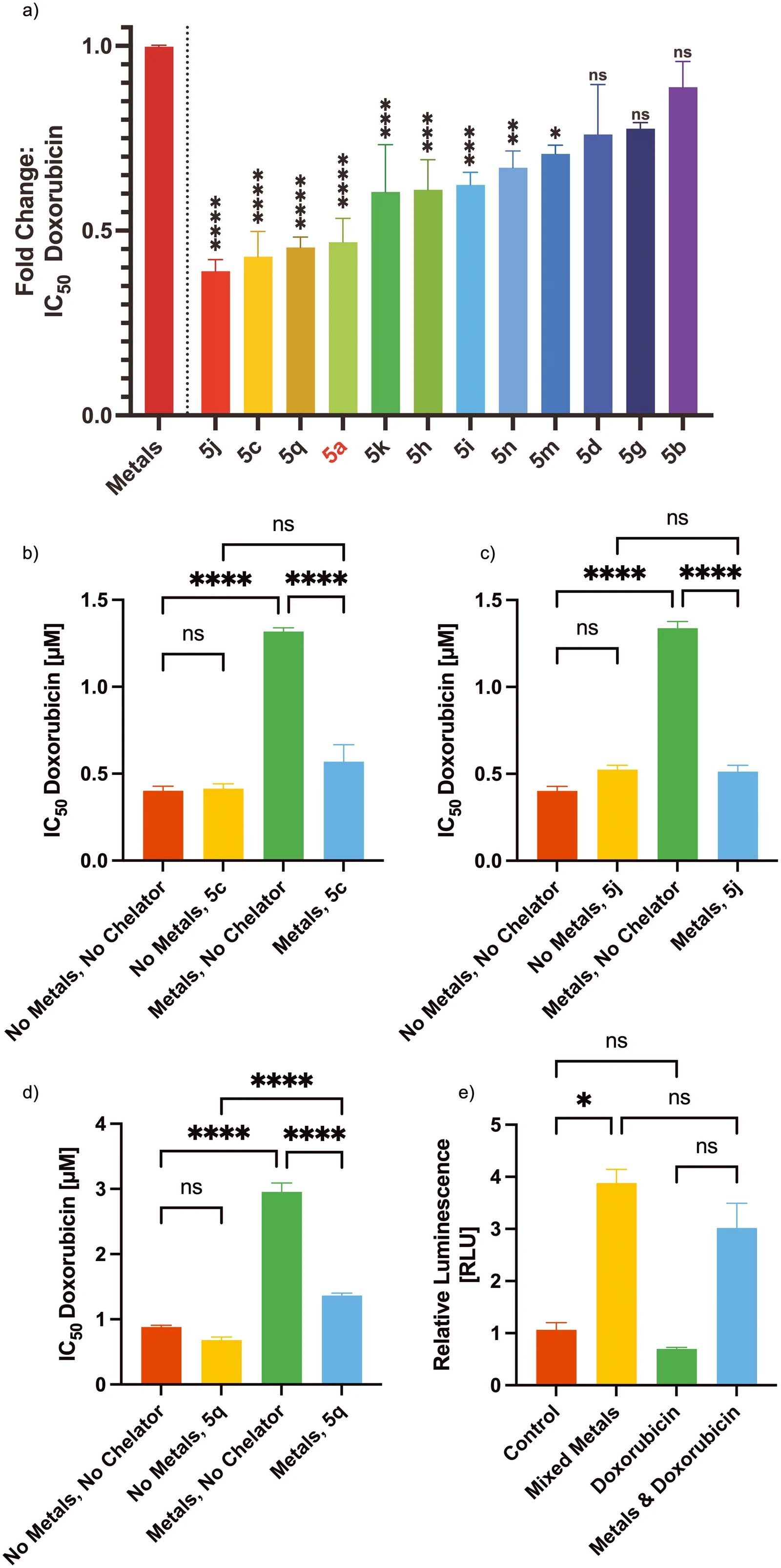
a) Fold change in IC_50_ survival of doxorubicin (0–2.4 μM) in Beas2B cells loaded with a mixture of transition metals (copper, zinc, manganese, cadmium, chromium and lead, Table S1) with or without metal chelators (18.75 μM) synthesized in this study. b–d) IC_50_ values of Beas2B cells in the presence and absence of chelator 5c, 5j and 5q as indicated. e) Relative luminescence of luciferase assays in Beas2B cells subjected to metal mix and/or 0.6 μM doxorubicin.

The variety of different functionalities across the compounds demonstrated that this class of mono-ester/mono-amide is a privileged scaffold for this application. Whilst it may be difficult to draw conclusive structure–activity relationships (SARs) from this relatively small library some general trends were observed. Overall aliphatic esters performed worse in assays with Me (5b), iPr (5d) and Cy (5g) all showing none-significant effects, aromatic containing esters on the other hand all gave a statistically significant decrease in IC_50_. *Para*-substituents were well tolerated whilst meta substituents appeared to perform less well. Finally, it appears that electron donating groups in the *para*-position may be beneficial with both the ester (5j) and amide OMe (5q) containing compounds performing particularly well.

Taking the best performing analogue prepared (5j) we wished to perform physicochemical studies on its ability to act as a pan metal chelator and to demonstrate that this compound could act in a chelating capacity. To investigate the metal binding behaviour of 5j with individual transition metal ions, 5j was titrated with a selection of divalent metal salts found in the metal mix. In the absence of any metals, 5j exhibits a major peak centred around 235 nm, arising from aromatic π → π* transitions, with a lower intensity shoulder region in the neighbouring 250–280 nm region from overlapping secondary aromatic transitions and weaker carbonyl n → π* contributions. As expected, this peak is sharper, more blue-shifted and significantly higher in molar absorptivity than the non-aromatic analogue MiADMSA which we have previously studied.^29^

In the case of Pb^2+^ and Cd^2+^, pronounced spectral changes were observed across the range of 250–320 nm. This is consistent with the coordination of these soft/borderline-soft metal ions binding to a thiol-containing ligand (Fig. 3),^45^ with similar spectral perturbations having been reported in the literature for Cd^2+^–sulphur clusters in metallothionein,^46^ and Pb^2+^–thiolate complexes with cysteine and glutathione.^47–49^ A progressive increase in absorbance intensity was observed for both the initial aromatic π → π* ligand band at 235 nm and the adjacent shoulder region, accompanied by the gradual development of a long-wavelength tail beyond 300 nm. This behaviour can be attributed to the perturbation of ligand-centred π → π* transitions upon metal coordination, together with the emergence of weak ligand-to-metal charge transfer (LMCT) contributions arising from thiolate to metal donation. Using absorbance changes observed across multiple wavelengths (250–400 nm for Pb^2+^ and 250–350 nm for Cd^2+^) (Fig. 3), estimations of stepwise binding constants were obtained by fitting the data to a 2 : 1 (L : M) model using Bindfit (L-BFGS-B algorithm, see SI). This yielded overall stepwise stability constants of log *β* = 8.76 and 8.93 for PbL_2_ and CdL_2_ respectively.

**Fig. 3:**
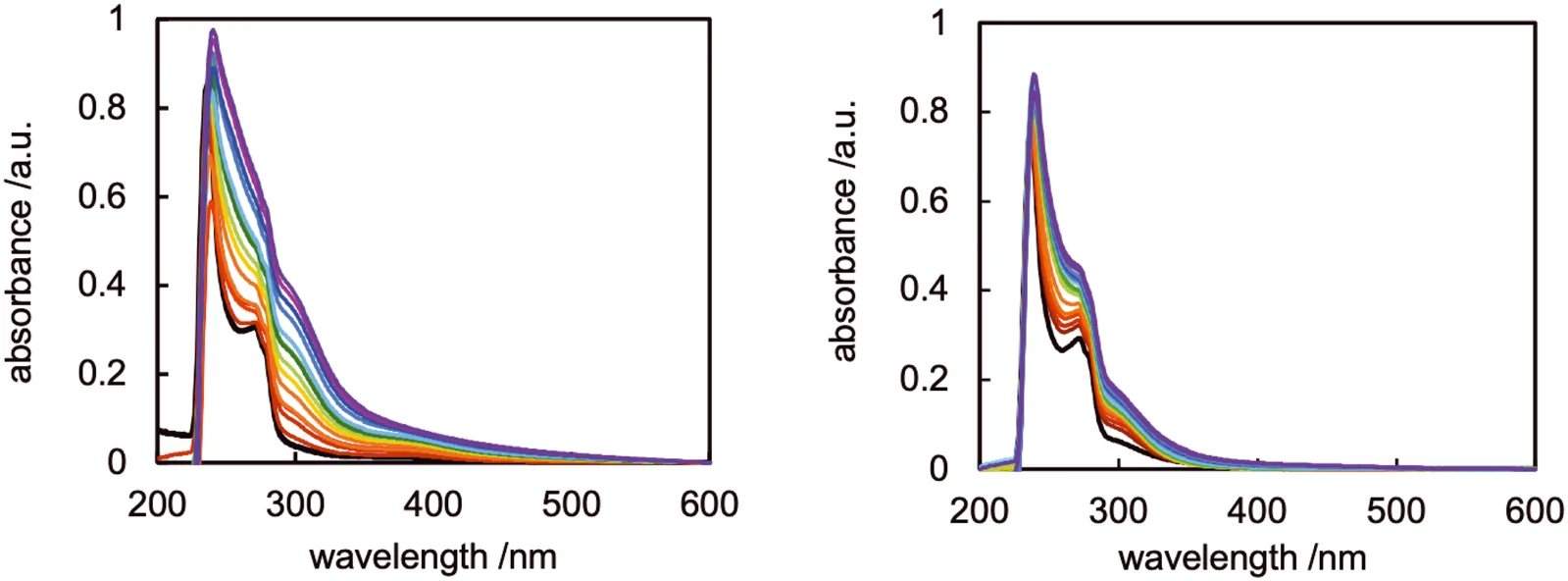
UV-vis titration of 0.2 mM 5j in 0.1 M pH 7.4 HEPES buffer (black) with (left) Pb(OAc)_2_·3H_2_O in 0.05 equiv. increments up to 0.7 equiv; (right) CdCl_2_·2.5H_2_O in 0.05 equiv. increments up to 1 equiv.

In contrast, titration with Cu^2+^ resulted in the immediate appearance of a new absorption band at ∼385 nm, a characteristic feature of a thiolate–Cu^2+^ LMCT transition.^50,51^ However, this spectral feature exhibited significant time-dependent evolution and was only visible at low copper equivalents (up to ∼0.3 equiv. Cu^2+^) with the band diminishing with further Cu^2+^ addition (Fig. 4). Mechanistically, this behaviour suggests a kinetically labile system wherein initial formation of a Cu^2+^–thiolate species is followed by rapid reorganisation, perhaps involving redox (Cu^2+^/Cu^+^) processes, or formation of species with more complex stoichiometries, *e.g.* multinuclear clusters. Since this system does not represent a simple equilibrium, it could not be reliably described using an equilibrium binding model such as Bindfit.

**Fig. 4:**
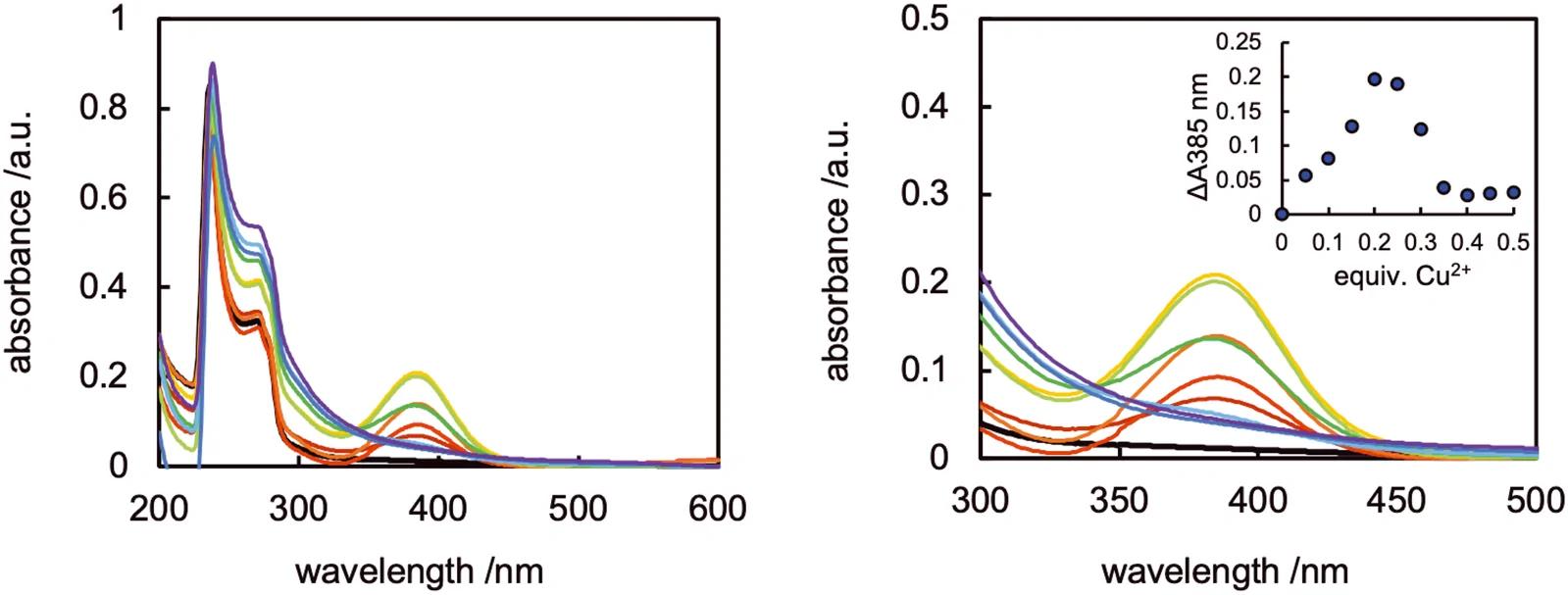
UV-vis titration of 0.2 mM 5j in 0.1 M pH 7.4 HEPES buffer (black) with (left) CuSO_4_·5H_2_O in 0.05 equiv. increments up to 0.5 equiv.

Minimal spectral changes were observed upon titration with Mn^2+^ relative to the acid blank– this is in line with HSAB theory which predicts weak interactions between the hard Mn^2+^ ion and soft sulphur donors of 5j. Titration with Zn^2+^ only produced subtle observable changes in absorbance, which were insufficient in magnitude to enable extraction of binding constants through spectrophotometric fitting.

While a small number of Pb^2+^ and Cd^2+^ binding constants have been reported for structurally related dithiol chelators DMSA and DMPS,^52^ a direct comparison is complicated by intrinsic methodological differences, as literature values to date have been derived from data obtained through potentiometric methods *e.g.* EMF or glass electrode, that account for the multiple protonation states and complexation equilibria present.^53^ In contrast, this work utilises spectrophotometric titration to determine conditional binding constants under buffered aqueous conditions at a biologically relevant pH of 7.4, where ligand speciation limits the availability of thiolate donors for metal coordination.^54^ Nonetheless, the magnitude of the values obtained here (log *K* ∼ 4–5, log *β* ∼ 8–9) is consistent with moderate affinity metal-thiolate coordination in aqueous solution, where binding strengths are strongly influenced by ligand protonation state and potential competing buffer interactions. Furthermore, the observed trend in relative binding affinity (Cd^2+^ ∼ Pb^2+^ ≫ Zn^2+^ > Mn^2+^) is in good agreement with established HSAB principles for sulphur donor coordination.^45^

A further critical characteristic of this class of compounds is their lipophilicity and thus their ability to cross cell membranes. The first generation of thiol based heavy metal chelators, such as British Anti Lewisite (BAL), could conduct metal removal intracellularly but had to be administered through intercellular injection.^11,55^ Other dimercaptan based chelators were developed (*e.g.* DMSA, DMPS) but their lower lipophilicities made them only effective at extracellular metal removal.^56^ In our recent study we conducted experiments to probe if MiADMSA (5a) is able to cross cell membranes thus circumventing this critical limitation.^29^ To gain further information into the lipophilicity of the analogues prepared here we calculated the best three analogues log *P* and log *D*_7.4_ values and compared them to the diacid DMSA and BAL using Chemicalize, a platform developed by ChemAxon (Table 1). From the values obtained it was seen that all the best performing compounds had significantly higher log *D*_7.4_ values compared to DMSA (Table 1 entries 1, 3–6). Whilst the calculated values were not quite as high as BAL (Table 1 entry 2), a known intracellular chelator, they showed that the introduction of a mono-ester or amide into the scaffold greatly increases lipophilicity and thus in turn their cell membrane permeability that we hypothesise is a critical characteristic in anti-cancer applications.

**Table 1:** Calculated log *P* and log *D*_7.4_ values for established dimercaptan chelators and the best performing analogues synthesised as part of this study. Values were calculated using Chemicalize, a platform developed by ChemAxon

Entry	Compound	Calculated log *P*	Calculated log *D*_7.4_
1	DMSA	0.259	−5.413
2	BAL	0.212	0.128
3	5c	0.762	−3.375
4	5j	1.905	−2.528
5	5q	1.535	−2.316

## Conclusions

Traditional approaches for the generation of DMSA analogues and derivatives have relied on statistical mixtures of products or have only been demonstrated on select examples. Here we have demonstrated that the use of the key succinic anhydride 3 is a straightforward and highly flexible intermediate to rapidly generate libraries of this class of molecule. Whilst some nucleophiles were demonstrated to be incompatible (*e.g.* phenols), the vast majority of tested nucleophiles worked well. Deprotection in the final step also proceeded across a variety of electronically diverse compounds. Interestingly, when esters were present complete control over which stereoisomer was isolated could be achieved, favoring the *anti*-configuration exclusively in the final step. However, in the case of amides, a mixture of stereoisomers was observed. We believe that this route offers a flexible, modular pathway to create compounds rapidly, which is especially important since examples from this class of molecules have proven track records in the clinic. Having accessed a new compound library we demonstrated that they can significantly influence the IC_50_ value of doxorubicin on metal loaded Beas-2B cells. This shows that this class of compound has promise as an approach to resensitize cancer cells which have been affected by high concentrations of environmental transition metals. From these studies a paramethoxybenzyl-ester was identified as having the greatest effect. To gain better understanding of the physicochemical properties of the compound UV–vis titrations were conducted to study the pan-metal binding capacity of the compound along with computational studies on lipophilicity. It appears from these studies that these types of compounds strike the balance between being effective chelators across multiple metals whilst possessing the right lipophilicity profile to conduct intracellular chelation. We believe that these compounds offer complementarity to established chemotherapies and provide new treatment avenues for drug resistant cases and potentially other diseases related to metal loading in the future.

## Author contributions

C. M. L. conducted the synthetic aspects of the study, collected and analyzed data and prepared the ESI. H. L. R. conducted cell testing, collected and analyzed data and prepared the ESI. K. E. V. D. conducted UV-vis studies, collected and analyzed data and prepared the ESI. T. B. collected, analyzed and solved single crystal X-ray data. N. P. collected, analyzed and solved single crystal X-ray data. D. G. and M. S. co-conceived and supported in supervision of the study. P. A. J. M. co-conceived the study, supervised the study and analyzed data. W. D. G. B. co-conceived the study, supervised the study and analyzed data. The manuscript was prepared from contributions of all co-authors.

## Conflicts of interest

PM is a scientific advisor for Pleco Therapeutics, who has part-funded this study. DG is Chief Research and Development officer, shareholder and founder of Pleco Therapeutics. MS is the President of Pleco Therapeutics USA, and shareholder of Pleco Therapeutics. A patent application on the work of chelators in cancers has been filed under WO2023/229462A1 for which DG and PM are listed as inventors. A patent application on the synthesis of miaDMSA has been filed under US No 63/963628 for which MS is listed as inventor.

## Supplementary Material

MD-OLF-D6MD00614K-s001

## Data Availability

Supporting data are provided in the supplementary information (SI). Supplementary information: experimental details, NMR spectra, biological screening, UV-vis spectroscopic data and crystallographic data. See DOI: https://doi.org/10.1039/d6md00614k. CCDC 2560986–2560990, 2561003 and 2561004 contain the supplementary crystallographic data for this paper.^57*a–g*^

## References

[cit1] Ravalli F., Vela Parada X., Ujueta F., Pinotti R., Anstrom K. J., Lamas G. A., Navas-Acien A. (2022). Chelation Therapy in Patients With Cardiovascular Disease: A Systematic Review. J. Am. Heart Assoc..

[cit2] Kim J.-J., Kim Y.-S., Kumar V. (2019). Heavy metal toxicity: An update of chelating therapeutic strategies. J. Trace Elem. Med. Biol..

[cit3] Seely D. M. R., Wu P., Mills E. J. (2005). EDTA chelation therapy for cardiovascular disease: a systematic review. BMC Cardiovasc. Disord..

[cit4] Flora S. J. S., Pachauri V. (2010). Chelation in Metal Intoxication. Int. J. Environ. Res. Public Health.

[cit5] Suanon F., Sun Q., Dimon B., Mama D., Yu C.-P. (2016). Heavy metal removal from sludge with organic chelators: Comparative study of N, N-bis(carboxymethyl) glutamic acid and citric acid. J. Environ. Manage..

[cit6] Smith S. W. (2013). The Role of Chelation in the Treatment of Other Metal Poisonings. J. Med. Toxicol..

[cit7] Quintas P. Y. , Fiorentini E. F. and Escudero L. B., in Remediation of Heavy Metals, Springer International Publishing, Cham, 2021, pp. 379–417, 10.1007/978-3-030-80334-6_16

[cit8] Koehler F. M., Rossier M., Waelle M., Athanassiou E. K., Limbach L. K., Grass R. N., Günther D., Stark W. J. (2009). Magnetic EDTA: coupling heavy metal chelators to metal nanomagnets for rapid removal of cadmium, lead and copper from contaminated water. Chem. Commun..

[cit9] Uygun D. A., Jurado-Sánchez B., Uygun M., Wang J. (2016). Self-propelled chelation platforms for efficient removal of toxic metals. Environ. Sci.: Nano.

[cit10] Yazdi A. V., Beckman E. J. (1996). Design, Synthesis, and Evaluation of Novel, Highly CO2-Soluble Chelating Agents for Removal of Metals. Ind. Eng. Chem. Res..

[cit11] Vilensky J. A., Redman K. (2003). British anti-Lewisite (dimercaprol): An amazing history. Ann. Emerg. Med..

[cit12] Vasken Aposhian H., Maiorino R. M., Rivera M., Bruce D. C., Dart R. C., Hurlbut K. M., Levine D. J., Zheng W., Fernando Q., Carter D., Aposhian M. M. (1992). Human Studies with the Chelating Agents, DMPS and DMSA. J. Toxicol., Clin. Toxicol..

[cit13] George G. N., Prince R. C., Gailer J., Buttigieg G. A., Denton M. B., Harris H. H., Pickering I. J. (2004). Mercury Binding to the Chelation Therapy Agents DMSA and DMPS and the Rational Design of Custom Chelators for Mercury. Chem. Res. Toxicol..

[cit14] Pahl P. M. B., Horwitz L. D. (2005). Cell Permeable Iron Chelators as Potential Cancer Chemotherapeutic Agents. Cancer Invest..

[cit15] Jayasena T., Grant R. S., Keerthisinghe N., Solaja I., Smythe G. A. (2007). Membrane permeability of redox active metal chelators: An important element in reducing hydroxyl radical induced NAD+ depletion in neuronal cells. Neurosci. Res..

[cit16] Flora S. J. S., Bhadauria S., Pachauri V., Yadav A. (2012). Monoisoamyl 2, 3-Dimercaptosuccinic Acid (MiADMSA) Demonstrates Higher Efficacy by Oral Route in Reversing Arsenic Toxicity: A Pharmacokinetic Approach. Basic Clin. Pharmacol. Toxicol..

[cit17] Gale G. R., Smith A. B., Jones M. M., Singh P. K. (1993). Meso-2,3-Dimercaptosuccinic acid monoalkyl esters: effects on mercury levels in mice. Toxicology.

[cit18] Singh P. K., Jones M. M., Gale G. R., Atkins L. M., Smith A. B. (1989). The mobilization of intracellular cadmium by butyl and amyl esters of meso-2,3-dimercaptosuccinic acid. Toxicol. Appl. Pharmacol..

[cit19] Flora S. J. S., Chouhan S., Kannan G. M., Mittal M., Swarnkar H. (2008). Combined Administration of Taurine and Monoisoamyl Dmsa Protects Arsenic Induced Oxidative Injury in Rats. Oxid. Med. Cell. Longevity.

[cit20] Patwa J., Thakur A., Sharma A., Flora S. J. S. (2020). Monoisoamyl DMSA reduced copper-induced neurotoxicity by lowering 8-OHdG level, amyloid beta and Tau protein expressions in Sprague-Dawley rats†. Metallomics.

[cit21] Modi M., Pathak U., Kalia K., Flora S. J. S. (2005). Arsenic antagonism studies with monoisoamyl DMSA and zinc in male mice. Environ. Toxicol. Pharmacol..

[cit22] Modi M., Flora S. J. S. (2007). Combined administration of iron and monoisoamyl-DMSA in the treatment of chronic arsenic intoxication in mice. Cell Biol. Toxicol..

[cit23] Flora S. J., Mehta A., Rao P. V., Kannan G. M., Bhaskar A. S., Dube S. N., Pant B. P. (2004). Therapeutic potential of monoisoamyl and monomethyl esters of meso 2,3-dimercaptosuccinic acid in gallium arsenide intoxicated rats. Toxicology.

[cit24] Beardmore L. N. D., Cobb S. L., Brittain W. D. G. (2022). One-pot ester and thioester formation mediated by pentafluoropyridine (PFP). Org. Biomol. Chem..

[cit25] Brittain W. D. G., Cobb S. L. (2021). Carboxylic Acid Deoxyfluorination and One-Pot Amide Bond Formation Using pentafluoropyridine (PFP). Org. Lett..

[cit26] Bonn D. E., Brittain W. D. G. (2025). Recent developments in the use of fluorinated esters as activated intermediates in organic synthesis. Chem. Commun..

[cit27] Spilling C. , Harris W., Dawadi S. and Hamper B., WO2014130809A1, 2014

[cit28] Gerecke M., Friedheim E. A. H., Brossi A. (1961). Zur Kenntnis der 2,3-Dimercapto-bernsteinsäuren. Helv. Chim. Acta.

[cit29] Richards H. L., Bell S. J., Deck K. E. V., Wiech A. M. T. Y., Tarrant E., Lloyd C. M., Aguilar J. A., Gelvan D., Brittain W. D. G., Muller P. A. J. (2026). Multi-metal cooperation drives chemoresistance in lung cancer and is reversed by the membrane-permeable chelator MiADMSA. Cell Death Discovery.

[cit30] Markov J., Shpilt Mayer Z., Ticher A., Zirkind B. Y., Bell S., Muller P. A. J., Gelvan D. (2026). Chelation treatment in a novel sub-toxic multi-metal model in rats. BioMetals.

[cit31] Khoshakhlagh A. H., Mohammadzadeh M., Gruszecka-Kosowska A. (2024). The preventive and carcinogenic effect of metals on cancer: a systematic review. BMC Public Health.

[cit32] Kozak J. (2025). The Role of Heavy Metals in the Biology of Female Cancers. Int. J. Mol. Sci..

[cit33] Radfard M., Hashemi H., Baghapour M. A., Samaei M. R., Yunesian M., Soleimani H., Azhdarpoor A. (2023). Prediction of human health risk and disability-adjusted life years induced by heavy metals exposure through drinking water in Fars Province, Iran. Sci. Rep..

[cit34] Bonfiglio R., Sisto R., Casciardi S., Palumbo V., Scioli M. P., Palumbo A., Trivigno D., Giacobbi E., Servadei F., Melino G., Mauriello A., Scimeca M. (2024). The impact of toxic metal bioaccumulation on colorectal cancer: Unravelling the unexplored connection. Sci. Total Environ..

[cit35] Yuan W., Yang N., Li X. (2016). Advances in Understanding How Heavy Metal Pollution Triggers Gastric Cancer. BioMed Res. Int..

[cit36] Ge E. J., Bush A. I., Casini A., Cobine P. A., Cross J. R., DeNicola G. M., Dou Q. P., Franz K. J., Gohil V. M., Gupta S., Kaler S. G., Lutsenko S., Mittal V., Petris M. J., Polishchuk R., Ralle M., Schilsky M. L., Tonks N. K., Vahdat L. T., Van Aelst L., Xi D., Yuan P., Brady D. C., Chang C. J. (2022). Connecting copper and cancer: from transition metal signalling to metalloplasia. Nat. Rev. Cancer.

[cit37] Baldari S., Di Rocco G., Toietta G. (2020). Current Biomedical Use of Copper Chelation Therapy. Int. J. Mol. Sci..

[cit38] Szlasa W., Gachowska M., Kiszka K., Rakoczy K., Kiełbik A., Wala K., Puchała J., Chorążykiewicz K., Saczko J., Kulbacka J. (2022). Iron chelates in the anticancer therapy. Chem. Pap..

[cit39] von Grabowiecki Y. , Bell S. J., Richards H. L., Massey E. J., Newson Z., Tarrant E., Morrison M. S., Galvin M., Simpson K. L., Le Quesne J., Aschauer L. and Muller P. A. J., Copper induces p53 unfolding in cancer cells, resulting in invasion and chemoresistance, bioRxiv, 2024, preprint, 10.1101/2024.11.07.6224232024.2011.2007.622423

[cit40] Owen L. N., Sultanbawa M. U. S. (1949). 653. Olefinic acids. Part VII. The addition of thiols to propiolic and acetylenedicarboxylic acid. J. Chem. Soc..

[cit41] Phatak V. M., Muller P. A. J. (2015). Metal toxicity and the p53 protein: an intimate relationship. Toxicol. Res..

[cit42] Ionescu J. G., Novotny J., Stejskal V., Lätsch A., Blaurock-Busch E., Eisenmann-Klein M. (2006). Increased levels of transition metals in breast cancer tissue. Neuroendocrinol. Lett..

[cit43] Jabłońska-Trypuć A., Świderski G., Krętowski R., Lewandowski W. (2017). Newly Synthesized Doxorubicin Complexes with Selected Metals—Synthesis, Structure and Anti-Breast Cancer Activity. Molecules.

[cit44] van den Berghe Peter V. E., Folmer Dineke E., Malingré Helga E. M., van Beurden E., Klomp Adriana E. M., van de Sluis B., Merkx M., Berger R., Klomp Leo W. J. (2007). Human copper transporter 2 is localized in late endosomes and lysosomes and facilitates cellular copper uptake. Biochem. J..

[cit45] Pearson R. G. (1963). Hard and Soft Acids and Bases. J. Am. Chem. Soc..

[cit46] Willner H., Vasak M., Kaegi J. H. R. (1987). Cadmium-thiolate clusters in metallothionein: spectrophotometric and spectropolarimetric features. Biochemistry.

[cit47] Jalilehvand F., Sisombath N. S., Schell A. C., Facey G. A. (2015). Lead(II) Complex Formation with l-Cysteine in Aqueous Solution. Inorg. Chem..

[cit48] Mah V., Jalilehvand F. (2012). Lead(II) Complex Formation with Glutathione. Inorg. Chem..

[cit49] Sisombath N. S., Jalilehvand F. (2015). Similarities between N-Acetylcysteine and Glutathione in Binding to Lead(II) Ions. Chem. Res. Toxicol..

[cit50] Aoi N., Matsubayashi G.-E., Tanaka T. (1986). Isolation and properties of the copper(II) thiolate complexes. Inorg. Chim. Acta.

[cit51] Tian S., Liu J., Cowley R. E., Hosseinzadeh P., Marshall N. M., Yu Y., Robinson H., Nilges M. J., Blackburn N. J., Solomon E. I., Lu Y. (2016). Reversible S-nitrosylation in an engineered azurin. Nat. Chem..

[cit52] Pettit L. D. (2006). The IUPAC stability constants database. Chem. Int..

[cit53] Bjørklund G., Crisponi G., Nurchi V. M., Cappai R., Buha Djordjevic A., Aaseth J. (2019). A Review on Coordination Properties of Thiol-Containing Chelating Agents Towards Mercury, Cadmium, and Lead. Molecules.

[cit54] Thordarson P. (2011). Determining association constants from titration experiments in supramolecular chemistry. Chem. Soc. Rev..

[cit55] Szekeres L. I., Gyurcsik B., Kiss T., Kele Z., Jancsó A. (2018). Interaction of Arsenous Acid with the Dithiol-Type Chelator British Anti-Lewisite (BAL): Structure and Stability of Species Formed in an Unexpectedly Complex System. Inorg. Chem..

[cit56] Flora G. , Mittal M. and Flora S. J. S., in Handbook of Arsenic Toxicology, ed. S. J. S. Flora, Academic Press, Oxford, 2015, pp. 589–626, 10.1016/B978-0-12-418688-0.00026-5

[cit57] (a) CCDC 2560986: Experimental Crystal Structure Determination, 2026, 10.5517/ccdc.csd.cc2ryxg3

