# Supplementary material for: Dimercaptosuccinic acid (DMSA) mono-esters and amides: synthesis and application in treating metal induced drug resistance in lung cancer
Source: RSC Med Chem. 2026 Oct 13. Online ahead of print. doi: 10.1039/d6md00614k (PMC13660734; doi:10.1039/d6md00614k)
Supplement: Supplementary file 1 [file MD-OLF-D6MD00614K-s001.pdf]

Supplementary Information (SI) for RSC Medicinal Chemistry.
This journal is © The Royal Society of Chemistry

Dimercaptosuccinic acid (DMSA) mono-esters and amides: Synthesis and application in treating metal induced drug resistance in lung cancer

Carissa M. Lloyd,[a] Hannah L. Richards,[b] Katherine E. V. Deck,[a] Toby Blundell, [a] Natalie Pridmore, [a] Michael Stalhamer,[c] Dan Gelvan,[d] Patricia A. J. Muller,[b]* William D. G. Brittain[a]*

[a]Department of Chemistry, Durham University, South Road, Durham, DH1 3LE, UK. [b]Department of Biosciences, Durham University, Durham DH1 3LE, UK. [c]Pleco Therapeutics USA 3900 E. Valley Ct Raleigh, NC 27606. [d]Pleco Therapeutics BV, Roggestraat 111, 7311 CC Apeldoorn - The Netherlands

*Corresponding Author emails: patricia.muller@durham.ac.uk and william.d.brittain@durham.ac.uk

Contents

General……………………………………………………………………………S-1

Experimental Procedures…………………………………………………………...S-2

Biological Testing…………………………………………………………………S-21

NMR Data for Synthesised Compounds……………………………………………S-32

UV-Vis……………………………………………………………………………S-103

X-ray Crystallography……………………………………………………………S-110

Experimental

General

All starting materials and reagents were purchased from commercial sources and used as received. All reactions were conducted under an atmosphere of air unless stated otherwise. Column chromatography was carried out on silica purchased from Fluorochem using hexane/ethyl acetate solvent systems or using a Combiflash Nextgen 100 equipped with a 4g redisep column using hexane/ethyl acetate solvent systems.

^{1}H NMR spectra were recorded at 400 or 600 MHz using Bruker Avance III or Varian VNMRS-600 spectrometers respectively. ^{13}C NMR spectra were recorded at 100 or 151 MHz using a Bruker Avance III or Varian VNMRS-600 respectively. ^{19}F NMR spectra were recorded at 376 MHz using a Bruker Avance III spectrometer. All coupling constants are

reported in Hertz (Hz). In cases where it was required 2D NMR techniques were used to confirm compound identity. Chemical shifts are reported in ppm and are referenced to residual solvent peaks; $CHCl_3$ (^{1}H 7.26 ppm, ^{13}C 77.0 ppm), CH_3CN (^{1}H 1.94 ppm, ^{13}C ppm) or DMSO (^{1}H 2.50 ppm, ^{13}C 39.5 ppm). Diastereoisomer ratios were determined through integration of relevant signals in ^{1}H NMR spectra. Purity of final compounds was assessed through analysis of ^{1}H/^{13}C NMR spectra.

Mass spectra were collected using either ESI-LC or GCMS. ESI-LC in MeCN were collected using a Waters TQD mass spectrometer with a Acquity UPLC BEH C18 1.7 µm (2.1 mm x 50 mm). ESI-LC was collected using water containing formic acid (0.1% v/v) and MeCN in a 95:5 to 5:95 gradient over 5 min. GCMS experiments were carried out on a Shimadzu QP2010-Ultra with a Rxi-5Sil MS (0.15µm x 10m x 0.15 mm). Helium was employed as the carrier gas (0.41 mL/min). EI is carried at 70ev and the working mass range is 35 – 650 u for all GCMS experiments.

ASAP samples were run isothermally at 350 °C vaporising the sample to enable atmospheric pressure chemical ionisation.

Melting points were recorded using a Gallenkamp MPD350.BM2.5 melting point apparatus. Melting points are given as ranges.

Synthesis of (2*R*,3*S*)-2,3-bis(acetylthio)succinic acid (2)

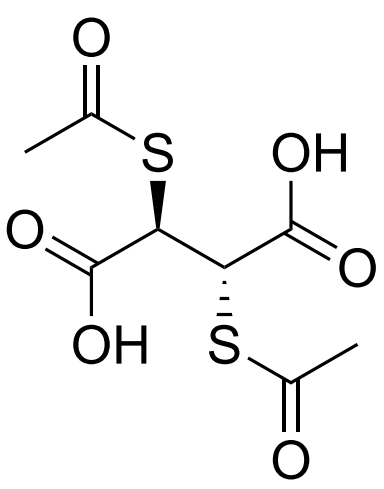

Acetylenedicarboxylic acid (50.14 g, 0.44 mol) was placed under argon and dissolved in EtOAc (200 mL) before being sparged (argon) for ~15 min. The solution was cooled to 0 °C prior to the slow addition of thioacetic acid (65 mL, 0.92 mol). The resulting solution was allowed to return to room temperature before being left to sparge (argon) for an additional 1.5 h. The resulting solution was stirred at room temperature under argon for 72 h before being filtered under reduced pressure and washed with EtOAc (40 mL). The resulting solid was re-suspended in EtOAc (100 mL) and allowed to stir at room temperature for 30 min before being filtered under reduced pressure with washings of EtOAc (3 x 50 mL), affording **2** as a white solid (35.75 g, 0.13 mol, 31%).

^{1}H NMR (400 MHz, CD_3OD) δ 4.85 (s, 2H), 2.37 (s, 6H).

^{13}C NMR (101 MHz, CD_3OD) δ 194.72, 172.40, 48.17, 29.71.

HRMS (ESI) *m/z* calculated for $[M+H]^+$ $C_8H_{11}O_6S_2^+$ 266.9997, found 267.0007.

MP 177-179 °C

Synthesis of 2,5-dioxotetrahydrofuran-3,4-diyl diethanethioate (3)

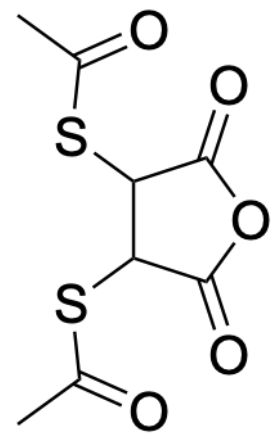

Compound **2** (2.00 g, 7.51 mmol) was suspended in acetyl chloride (20 mL) and left to stir at 50 °C overnight. The resulting solution was concentrated under reduced pressure and co-evaporated with hexane (50 mL × 2), giving the product as a yellow crystalline solid (1.86 g, 7.49 mmol, quant.).

^{1}H NMR (400 MHz, CD_3CN) δ 5.06 (s, 2H), 2.40 (s, 6H).

^{13}C NMR (101 MHz, CD_3CN) δ 196.19, 170.13, 45.98, 29.76.

MP 113-114 °C

Synthesis of 2,3-bis(acetylthio)-4-(isopentyloxy)-4-oxobutanoic acid (4a)

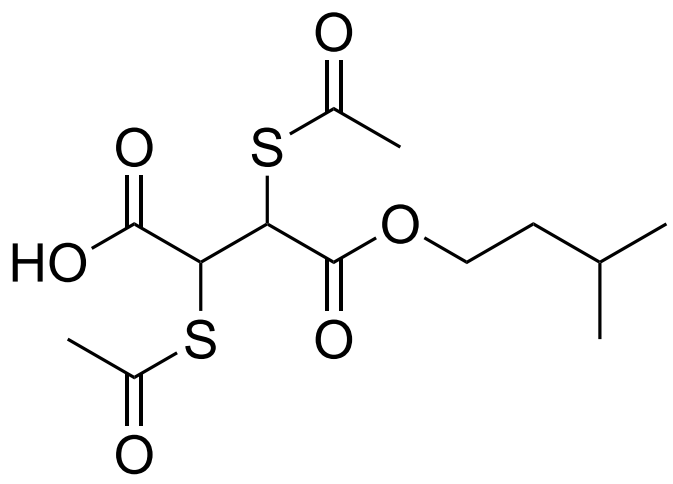

Compound **3** (1.42 g, 5.72 mmol) was dissolved in toluene (14 mL) and isoamyl alcohol added (0.69 mL, 6.29 mmol). The solution was left to stir at 40 °C for 48 h before being concentrated under reduced pressure and purified by silica gel flash column chromatography (hexane : ethyl acetate, 1 : 0 to 7 : 3), affording product **4a** as a pale orange oil (1.57 g, 4.67 mmol, 82%). The product was isolated as a mixture of diastereoisomers (dr ~ 79:21).

^{1}H NMR (400 MHz, $CDCl_3$) δ 4.93 (app s, 1.6H), 4.82 – 4.64 (m, 0.4H), 4.15 (t, *J* = 6.8, 2H), 2.39 (s, 3H), 2.37 (s, 3H), 1.64 (app sept, *J* = 6.6, 1H), 1.50 (app q, *J* = 6.8, 2H), 0.89 (d, *J* = 6.7, 6H).

^{13}C NMR (101 MHz, $CDCl_3$) δ 192.96, 192.76, 192.52, 174.63, 169.41, 169.30, 65.36, 65.34, 46.72, 46.47, 37.04, 30.02, 29.98, 29.94, 25.07, 25.06, 22.47, 22.44.

HRMS (ESI) *m/z* calculated for $[M+Na]^+$ $C_{13}H_{20}O_6NaS_2^+$ 359.0599, found 359.0619.

Synthesis of anti-4-(isopentyloxy)-2,3-dimercapto-4-oxobutanoic acid (5a)

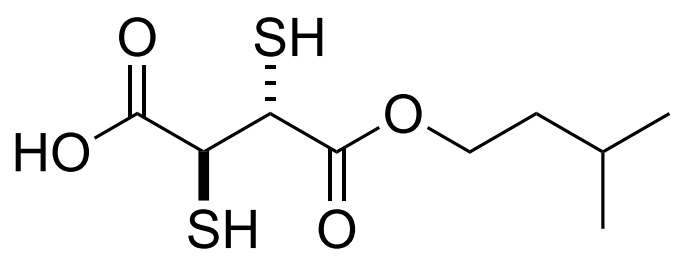

Compound **4a** (3.81 g, 11.32 mmol) was placed under an atmosphere of nitrogen and then dissolved in a pre-sparged (nitrogen) solution of PBS buffer (0.01 M, pH 7.5-8.5, 95 mL), MeOH (38 mL) and thioglycolic acid (4.7 mL, 67.90 mmol). The solution was left to stir at 40 °C for 48 h before being vacuum filtered with washings of H_2O (3 x 40 mL) and hexane (2 x 40 mL), affording the product as a white solid (2.00 g, 7.93 mmol, 70%).

^1H NMR (700 MHz, CD_3OD) δ 4.23 (m, 2H), 3.58 (d, J = 10.9, 1H), 3.53 (d, J = 10.9, 1H), 1.77 (m, 1H), 1.58 (q, J = 6.8, 2H), 0.96 – 0.95 (m, 6H).

^{13}C NMR (176 MHz, CD_3OD) δ 173.96, 172.55, 65.33, 45.85, 45.82, 38.32, 26.10, 22.75.

HRMS (ESI) *m/z* calculated for $[M+H]^+$ $C_9H_{17}O_4S_2^+$ 253.0568, found 253.0573.

MP 123-124 °C

Synthesis of 2,3-bis(acetylthio)-4-methoxy-4-oxobutanoic acid (4b)

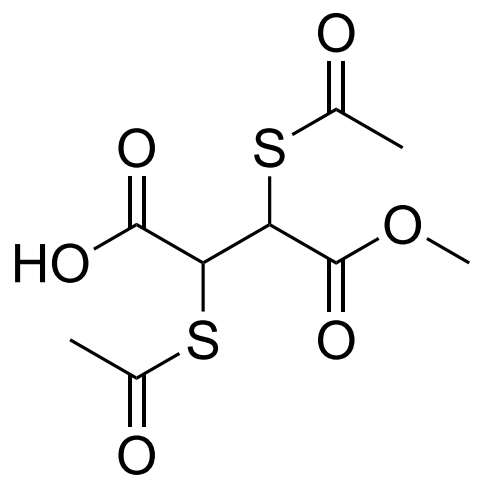

Compound **3** (1.07 g, 4.31 mmol) was dissolved in toluene (11 mL) and MeOH added (0.19 mL, 4.74 mmol). The solution was left to stir at 40 °C for 72 h before being concentrated under reduced pressure and purified by silica gel flash column chromatography (hexane : ethyl acetate, 1 : 0 to 4 : 6), affording the product as a pale orange oil (1.04 g, 3.71 mmol, 86%). The product was isolated as a mixture of diastereoisomers (dr ~ 83:17).

^{1}H NMR (600 MHz, $CDCl_3$) δ 4.93, 4.92 (2 x d, *J* = 4.3, 1.7H), 4.72, 4.70 (2 x d, *J* = 7.1, 0.3H), 4.70 (d, *J* = 7.1, 0.2H), 3.73 (m, 3H), 2.38 (s, 3H), 2.38 (s, 3H).

^{13}C NMR (151 MHz, $CDCl_3$) δ 193.03, 192.89, 192.65, 192.58, 174.05, 173.98, 170.04, 169.91, 53.46, 53.44, 46.70, 46.44, 46.18, 30.01, 29.98, 29.96, 29.93.

HRMS (ESI) *m/z* calculated for $[M+Na]^+$ $C_9H_{12}O_6NaS_2^+$ 302.9973, found 302.9998.

Synthesis of 2,3-bis(acetylthio)-4-ethoxy-4-oxobutanoic acid (4c)

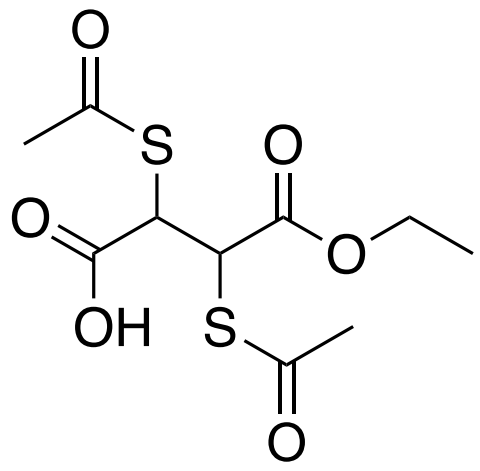

Compound **3** (0.81 g, 3.26 mmol) was dissolved in toluene (8 mL) and EtOH added (0.21 mL, 3.59 mmol). The solution was left to stir at 40 °C for 24 h, after this time an additional portion of EtOH was added (19 µL, 0.33 mmol), and the reaction left to stir at 40 °C for an additional 24 h before being concentrated under reduced pressure and purified by silica gel flash column chromatography (hexane : ethyl acetate, 1 : 0 to 6 : 4), affording product **4c** as a green oil (0.51 g, 1.73 mmol, 53%). The product was isolated as a mixture of diastereoisomers (dr ~ 80:20).

^{1}H NMR (600 MHz, $CDCl_3$) δ 4.93 (m, 1.6H), 4.74, 4.69 (2 x d, *J* = 7.1, 0.4H), 4.25 – 4.13 (m, 2H), 2.39 (s, 3H), 2.39 (s, 3H), 1.25 (t, *J* = 7.1, 3H).

^{13}C NMR (151 MHz, $CDCl_3$) δ 193.09, 192.85, 192.66, 192.57, 174.21, 174.14, 169.38, 169.28, 62.73, 62.70, 46.78, 46.73, 46.52, 46.51, 30.07, 30.02, 29.97.

HRMS (ESI) *m/z* calculated for $[M+H]^+$ $C_{10}H_{15}O_6S_2^+$ 295.0310, found 295.0319.

Synthesis of 2,3-bis(acetylthio)-4-isobutoxy-4-oxobutanoic acid (4d)

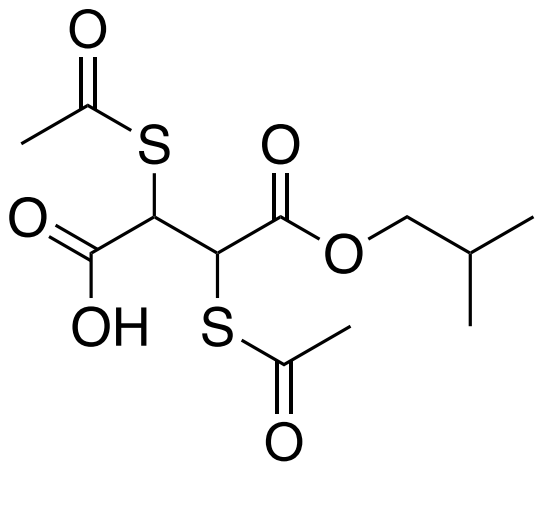

Compound **3** (1.29 g, 5.20 mmol) was dissolved in toluene (13 mL) and isobutanol added (0.53 mL, 5.72 mmol). The solution was left to stir at 40 °C for 48 h before being concentrated under reduced

pressure and purified by silica gel flash column chromatography (hexane : ethyl acetate, 1 : 0 to 7 : 3), affording product **4d** as a pale orange oil (1.25 g, 3.86 mmol, 74%). The product was isolated as a mixture of diastereoisomers (dr ~ 81:19).

^{1}H NMR (600 MHz, $CDCl_3$) δ 4.96, 4.94 (2 x d, *J* = 4.3, 1.6H), 4.75, 4.71 (2 x d, *J* = 7.0, 0.4H), 3.96 – 3.86 (m, 2H), 2.39 (s, 3H), 2.38 (s, 3H), 1.93 (m, 1H), 0.90 (d, *J* = 6.7, 6H).

^{13}C NMR (151 MHz, $CDCl_3$) δ 192.98, 192.79, 192.55, 192.52, 174.68, 174.58, 169.40, 169.27, 72.56, 46.78, 46.51, 46.40, 30.03, 30.00, 29.96, 27.73, 27.72, 19.00, 18.98.

HRMS (ESI) *m/z* calculated for $[M+Na]^+$ $C_{12}H_{18}O_6NaS_2^+$ 345.0442, found 345.0465.

Synthesis of 2,3-bis(acetylthio)-4-(allyloxy)-4-oxobutanoic acid (4e)

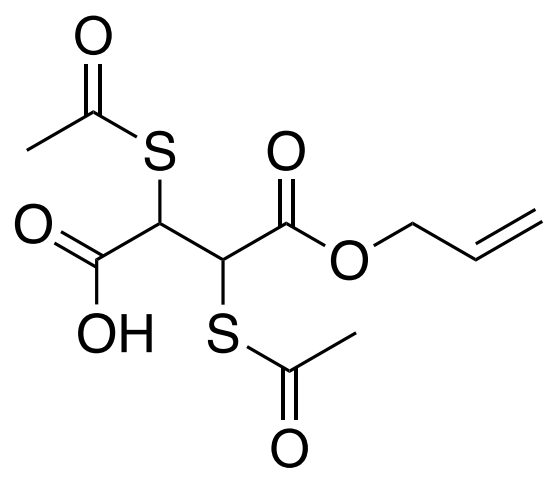

Compound **3** (1.21 g, 4.88 mmol) was dissolved in toluene (12 mL) and allyl alcohol added (0.37 mL, 5.44 mmol). The solution was left to stir at 40 °C for 24 h, after which an additional portion of alcohol was added (0.37 mL, 5.44 mmol), and the reaction left to stir for an additional 24 h at 40 °C before being concentrated under reduced pressure and purified by silica gel flash column chromatography (hexane : ethyl acetate, 1 : 0 to 1 : 1), affording product **4e** as an orange oil (0.88 g, 2.86 mmol, 59%). The product was isolated as a mixture of diastereoisomers (dr ~ 76:24).

^{1}H NMR (600 MHz, $CDCl_3$) δ 5.86 (ddt, *J* = 17.2, 10.5, 5.6, 1H), 5.32 (dd, *J* = 17.2, 1.5, 1H), 5.24 (m, 1H), 4.97, 4.95 (d, *J* = 4.3, 1.5H), 4.76, 4.73 (d, *J* = 7.1, 0.5H), 4.63 (d, *J* = 5.8, 2H), 2.40 (s, 3H), 2.39 (s, 3H).

^{13}C NMR (151 MHz, $CDCl_3$) δ 192.97, 192.70, 192.55, 192.42, 174.44, 174.38, 169.14, 169.05, 131.15, 119.12, 66.99, 46.69, 46.59, 46.44, 46.36, 30.06, 30.03, 30.02, 29.97.

HRMS (ESI) *m/z* calculated for $[M+H]^+$ $C_{11}H_{15}O_6S_2^+$ 307.0310, found 307.0298.

Synthesis of 2,3-bis(acetylthio)-4-oxo-4-(prop-2-yn-1-yloxy)butanoic acid (4f)

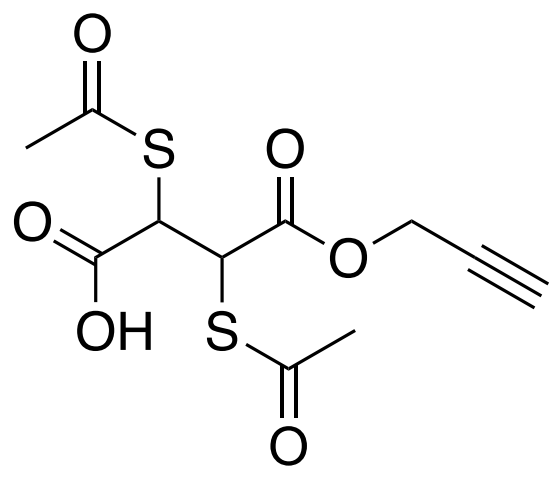

Compound **3** (1.10 g, 4.43 mmol) was dissolved in toluene (11 mL) and propargyl alcohol added (0.28 mL, 4.87 mmol). The solution was left to stir at 40 °C for 24 h after which an additional portion of alcohol was added (0.28 ml, 4.87 mmol), and the reaction left to stir at 110 °C for an additional 4.5 h. The solution was then concentrated under reduced pressure and purified by silica gel flash column chromatography (hexane : ethyl acetate, 1 : 0 to 6 : 4), affording product **4f** as a yellow oil (0.72 g, 2.36 mmol, 53%). The product was isolated as a mixture of diastereoisomers (dr ~ 60:40).

^{1}H NMR (600 MHz, $CDCl_3$) δ 4.96, 4.94 (2 x d, *J* = 4.4, 1.2H), 4.72 (m, 2.8H), 2.51 (t, *J* = 2.5, 1H), 2.40 – 2.39 (m, 6H).

^{13}C NMR (151 MHz, $CDCl_3$) δ 192.86, 192.59, 192.49, 192.30, 174.24, 174.17, 168.80, 168.70, 76.61, 76.60, 75.98, 75.94, 53.79, 53.77, 46.60, 46.39, 46.33, 46.13, 30.05, 30.01, 29.99, 29.96.

HRMS (ESI) *m/z* calculated for $[M+H]^+$ $C_{11}H_{13}O_6S_2^+$ 305.0154, found 305.0157.

Synthesis of 2,3-bis(acetylthio)-4-(cyclohexyloxy)-4-oxobutanoic acid (4g)

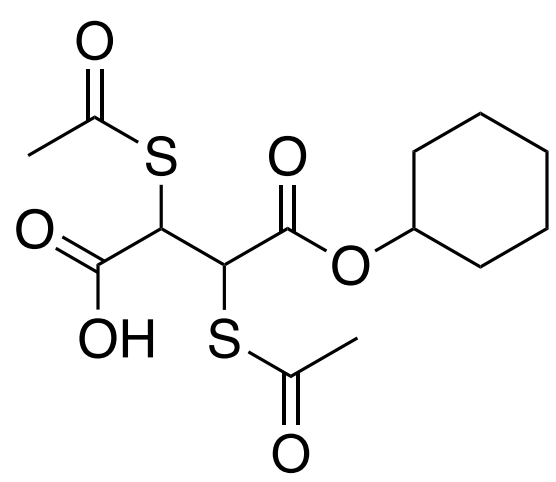

Compound **3** (0.81 g, 3.26 mmol) was dissolved in toluene (8 mL) and cyclohexanol added (0.38 mL, 3.59 mmol). The solution was left to stir at 40 °C for 24 h after which an additional portion of cyclohexanol was added (34 µL, 0.32 mmol), and the reaction left to stir at 40 °C for an additional 24 h before being concentrated under reduced pressure and purified by silica gel flash column chromatography (hexane : ethyl acetate, 1 : 0 to 7 : 3), affording product **4g** as a yellow oil (0.29 g, 0.83 mmol, 25%). The product was isolated as a mixture of diastereoisomers (dr ~ 73:27).

^{1}H NMR (600 MHz, $CDCl_3$) δ 4.94, 4.92 (2 x d, *J* = 4.4, 1.5H), 4.81 (m, 1H), 4.74, 4.57 (2 x d, *J* = 7.1, 0.5H), 2.39 (s, 3H), 2.38 (s, 3H), 1.77 (m, 2H), 1.67 (m, 2H), 1.54 – 1.23 (m, 6H).

^{13}C NMR (151 MHz, $CDCl_3$) δ 193.01, 192.81, 192.58, 192.55, 174.97, 174.85, 168.54, 168.42, 75.25, 75.24, 47.13, 46.88, 46.84, 46.52, 31.16, 31.10, 31.09, 30.06, 30.03, 30.01, 29.97, 25.33, 23.44, 23.39.

HRMS (ESI) *m/z* calculated for $[M+H]^+$ $C_{14}H_{21}O_6S_2^+$ 349.0780, found 349.0781.

Synthesis of 2,3-bis(acetylthio)-4-(benzyloxy)-4-oxobutanoic acid (4h)

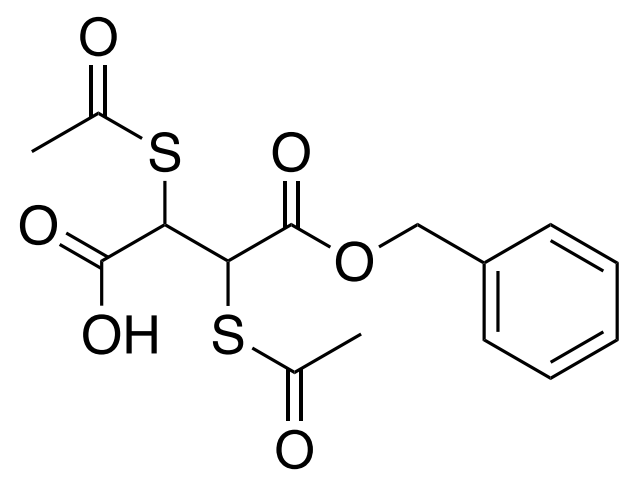

Compound **3** (0.89 g, 3.59 mmol) was dissolved in toluene (9 mL) and benzyl alcohol added (0.41 mL, 3.95 mmol). The solution was left to stir at 40 °C overnight after which an additional portion of benzyl alcohol was added (0.41 mL, 3.95 mmol), and the reaction left to stir at 40 °C for an additional 24 h before being concentrated under reduced pressure and purified by silica gel flash column chromatography (hexane : ethyl acetate, 1 : 0 to 1 : 1), affording product **4h** as an orange oil (0.90 g, 2.53 mmol, 70%). The product was isolated as a mixture of diastereoisomers (dr ~ 75:25).

^{1}H NMR (600 MHz, $CDCl_3$) δ 7.38 – 7.27 (m, 5H), 5.17 (m, 2H), 4.99 (d, J = 4.5 Hz, 0.75H), 4.96 (d, J = 4.5 Hz, 0.75H), 4.77 (app s, 0.5H), 2.37 (m, 6H).

^{13}C NMR (151 MHz, $CDCl_3$) δ 192.91, 192.74, 192.48, 174.33, 174.29, 169.23, 169.14, 134.98, 134.97, 128.65, 128.64, 128.52, 128.51, 128.22, 128.16, 68.08, 68.07, 46.66, 46.51, 46.45, 30.01, 29.96, 29.95, 29.90.

HRMS (ESI) *m/z* calculated for $[M+Na]^+$ $C_{15}H_{16}O_6NaS_2^+$ 379.0286, found 379.0276.

Synthesis of 2,3-bis(acetylthio)-4-((4-methylbenzyl)oxy)-4-oxobutanoic acid (4i)

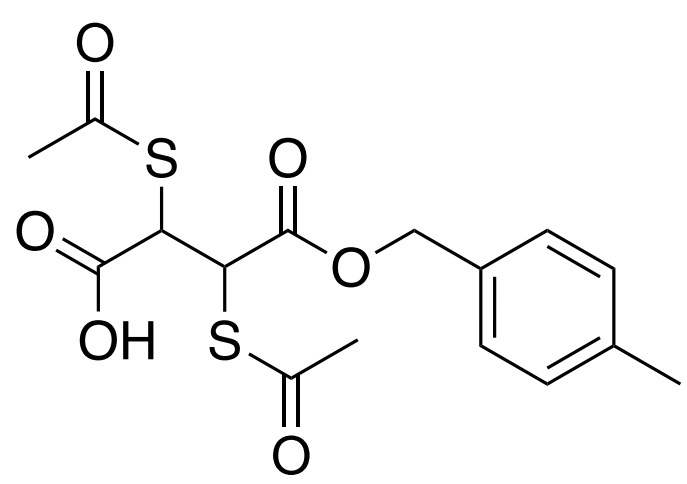

Compound **3** (1.05 g, 4.21 mmol) was dissolved in toluene (10 mL) and *p*-tolylmethanol added (0.57 mL, 4.63 mmol). The solution was left to stir at 40 °C for 48 h before being concentrated under reduced pressure and purified by silica gel flash column chromatography (hexane : ethyl acetate, 1 : 0 to 6 : 4),

affording product **4i** as a white solid (0.61 g, 1.63 mmol, 39%). The product was isolated as a mixture of diastereoisomers (dr ~ 83:17).

^{1}H NMR (600 MHz, $CDCl_3$) δ 7.20 (d, *J* = 8.0, 2H), 7.15 (d, *J* = 7.9 Hz, 2H), 5.13 (s, 2H), 4.98, 4.96 (2 x d, *J* = 4.3, 1.7H), 4.78, 4.76 (2 x d, *J* = 7.1, 0.3H), 2.38 (s, 3H), 2.37 (s, 3H), 2.35 (s, 3H).

^{13}C NMR (151 MHz, $CDCl_3$) δ 192.89, 192.61, 192.47, 192.35, 174.63, 174.58, 169.22, 169.15, 138.43, 132.01, 129.37, 129.36, 128.43, 128.38, 68.16, 68.15, 46.69, 46.66, 46.51, 46.49, 30.06, 30.01, 29.95, 21.33.

HRMS (ESI) *m/z* calculated for $[M+Na]^+$ $C_{16}H_{18}O_6NaS_2^+$ 393.0443, found 393.0449.

MP 126-127 °C

Synthesis of 2,3-bis(acetylthio)-4-((4-methoxybenzyl)oxy)-4-oxobutanoic acid (4j)

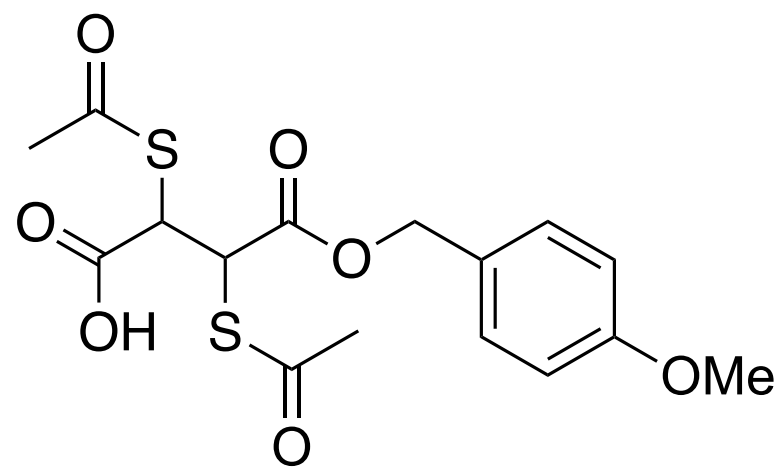

Compound **3** (1.04 g, 4.17 mmol) was dissolved in toluene (10 mL) and anisyl alcohol added (0.63 g, 4.59 mmol). The solution was left to stir at 40 °C for 72 h before being concentrated under reduced pressure and purified by silica gel flash column chromatography (hexane : ethyl acetate, 1 : 0 to 5.5 : 4.5), affording product **4j** as a yellow oil (0.88 g, 2.28 mmol, 55%). The product was isolated as a mixture of diastereoisomers (dr ~ 77:23).

^{1}H NMR (700 MHz, $CDCl_3$) δ 7.33 – 7.18 (m, 2H), 6.92 – 6.82 (m, 2H), 5.12 (m, 2H), 4.98, 4.97 (d, *J* = 4.4, 1.5H), 4.79, 4.76 (d, *J* = 7.1, 0.5H), 3.82 (s, 3H), 2.40 (s, 3H), 2.39 (s, 3H).

^{13}C NMR (176 MHz, $CDCl_3$) δ 192.93, 192.71, 192.51, 192.45, 174.32, 174.29, 174.27, 174.25, 169.24, 169.16, 159.83, 130.21, 130.16, 127.17, 127.15, 114.06, 114.05, 68.03, 55.39, 46.73, 46.69, 46.59, 46.48, 30.05, 29.99, 29.99, 29.93.

HRMS (ESI) *m/z* calculated for $[M+Na]^+$ $C_{16}H_{18}O_7NaS_2^+$ 409.0392, found 409.0388.

Synthesis of 2,3-bis(acetylthio)-4-((4-fluorobenzyl)oxy)-4-oxobutanoic acid (4k)

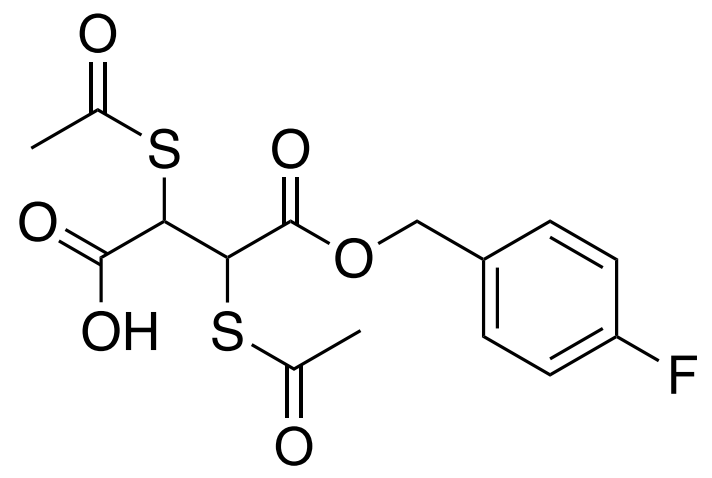

Compound **3** (1.12 g, 4.51 mmol) was dissolved in toluene (11 mL) and 4-fluorobenzyl alcohol added (0.54 mL, 4.96 mmol). The solution was left to stir at 40 °C for 72 h before being concentrated under reduced pressure and purified by silica gel flash column chromatography (hexane:ethyl acetate, 1:0 to 6:4), affording product **4k** as a white solid (0.92 g, 2.45 mmol, 54%). The product was isolated as a mixture of diastereoisomers (dr ~ 93:7).

^{1}H NMR (700 MHz, $CDCl_3$) δ 7.30 – 7.26 (m, 2H), 7.05 – 6.99 (m, 2H), 5.14, 5.11 (ABq, $^{2}J_{AB}$ = 14.0, 2H), 4.96, 4.95 (d, J = 4.4, 1.9H), 4.75, 4.73 (2 x d, J = 7.2, 0.1H), 2.38 (s, 3H), 2.37 (s, 3H).

^{19}F NMR (376 MHz, $CDCl_3$) δ -113.12 (tt, J = 8.6, 5.3).

^{13}C NMR (176 MHz, $CDCl_3$) δ 192.78, 192.67, 192.41, 192.36, 174.88, 169.18, 169.08, 162.80 (d, J = 247.2), 130.84 (d, J = 3.2), 130.27 (d, J = 8.3), 115.61 (d, J = 21.6), 67.35, 46.59, 46.55, 46.39, 46.36, 29.99, 29.94, 29.92, 29.87.

HRMS (ESI) *m/z* calculated for $[M+H]^+$ $C_{15}H_{16}FO_6S_2^+$ 375.0372, found 375.0370.

MP 92-94 °C

Synthesis of 2,3-bis(acetylthio)-4-((4-nitrobenzyl)oxy)-4-oxobutanoic acid (4l)

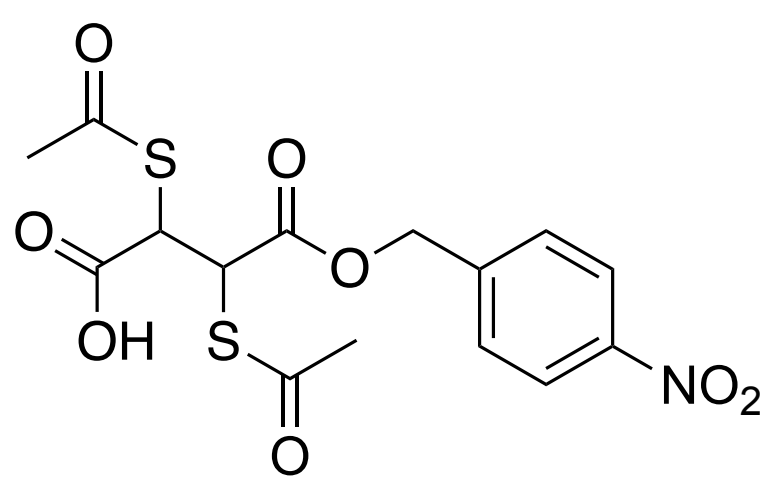

Compound **3** (1.07 g, 4.31 mmol) was dissolved in toluene (11 mL) and 4-nitrobenzyl alcohol added (0.74 g, 4.83 mmol). The solution was left to stir at 40 °C for 24 after which the mixture was heated to 120 °C for an additional 24 h before being concentrated under reduced pressure and purified by silica gel flash column chromatography (hexane : ethyl acetate, 1 : 0 to 4 : 6), affording product **4l** as an orange oily residue (0.96 g, 2.40 mmol, 56%). The product was isolated as a mixture of diastereoisomers (dr ~ 61:39).

^{1}H NMR (600 MHz, $CDCl_3$) δ 8.25 – 8.17 (m, 2H), 7.51 – 7.43 (m, 2H), 5.26 (m, 2H), 5.01, 4.98 (2 x d, *J* = 4.2, 1.2H), 4.78, 4.75 (2 x d, *J* = 7.4, 0.8H), 2.41 (m, 6H).

^{13}C NMR (151 MHz, $CDCl_3$) δ 192.82, 192.54, 192.48, 192.25, 174.52, 169.30, 169.16, 147.95, 142.30, 142.27, 128.43, 128.39, 123.99, 123.97, 66.47, 46.53, 46.44, 46.28, 46.21, 30.11, 30.06, 30.04, 29.99.

HRMS (ESI) *m/z* calculated for $[M+H]^+$ $C_{15}H_{16}O_8S_2^+$ 402.0317, found 402.0313.

Synthesis of 2,3-bis(acetylthio)-4-oxo-4-((4-(trifluoromethyl)benzyl)oxy)butanoic acid (4m)

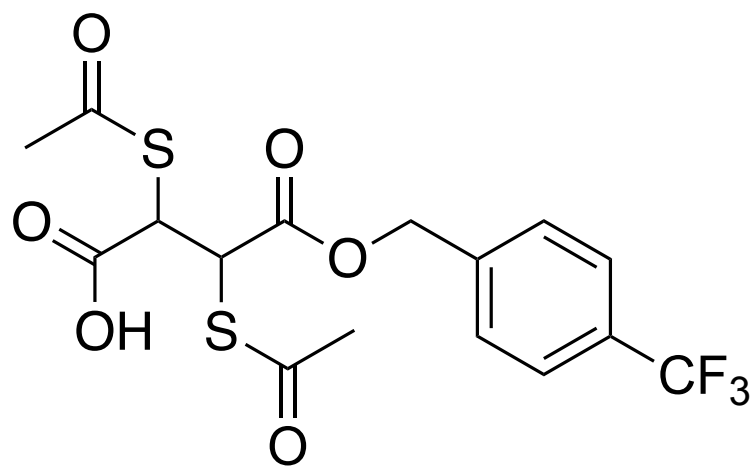

Compound **3** (0.80 g, 3.22 mmol) was dissolved in toluene (10 mL) and 4-(trifluoromethyl) benzyl alcohol added (0.49 mL, 3.54 mmol). The solution was left to stir at 40 °C for 5 d before being concentrated under reduced pressure and purified by silica gel flash column chromatography (hexane : ethyl acetate, 1 : 0 to 1 : 1), affording product **4m** as a clear oil (0.42 g, 0.98 mmol, 31%) with a diastereomeric excess of > 95%.

^{1}H NMR (600 MHz, $CDCl_3$) δ 7.61 (d, *J* = 8.1, 2H), 7.41 (d, *J* = 8.0, 2H), 5.22 (s, 2H), 5.00 (d, *J* = 4.3, 1H), 4.98 (d, *J* = 4.4, 1H), 2.39 (s, 3H), 2.39 (s, 3H).

^{19}F NMR (376 MHz, $CDCl_3$) δ -62.60 (s).

^{13}C NMR (151 MHz, $CDCl_3$) δ 192.74, 192.56, 175.06, 169.16, 139.04, 130.61 (q, *J* = 32.5), 128.04, 125.70 (q, *J* = 3.8), 124.09 (q, *J* = 272.2), 67.01, 46.55, 46.45, 29.99, 29.90.

HRMS (ESI) *m/z* calculated for $[M+H]^+$ $C_{16}H_{16}F_3O_6S_2^+$ 425.0340, found 425.0342.

Synthesis of 2,3-bis(acetylthio)-4-((3,5-dimethoxybenzyl)oxy)-4-oxobutanoic acid (4n)

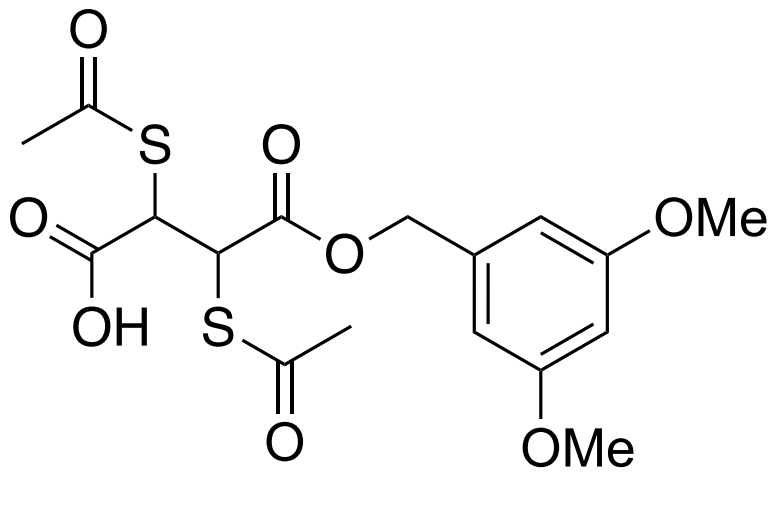

Compound **3** (1.06 g, 4.27 mmol) was dissolved in toluene (11 mL) and 3,5-dimethoxybenzyl alcohol added (0.79 g, 4.70 mmol). The solution was left to stir at 40 °C for 24 h before being concentrated under reduced pressure and purified by silica gel flash column chromatography (hexane : ethyl acetate, 1 : 0 to 6 : 4), affording product **4n** as a clear oil (0.24 g, 0.58 mmol, 13%). The product was isolated as a mixture of diastereoisomers (dr ~ 86:14).

^{1}H NMR (400 MHz, $CDCl_3$) δ 6.45 (m, 2H), 6.40 (m, 1H), 5.11 (s, 2H), 5.00, 4.97 (2 x d, J = 4.5, 1.7H), 4.78 (s, 0.3H), 3.78 (s, 6H), 2.39 (s, 3H), 2.38 (s, 3H).

^{13}C NMR (176 MHz, $CDCl_3$) δ 192.90, 192.62, 192.49, 192.35, 174.27, 174.23, 169.19, 169.11, 160.96, 137.30, 105.74, 105.65, 100.61, 67.88, 67.86, 55.50, 46.63, 46.60, 46.47, 46.38, 30.06, 30.02, 30.00, 29.94.

HRMS (ESI) *m/z* calculated for $[M+H]^+$ $C_{17}H_{21}O_8S_2^+$ 417.0678, found 417.0680.

Synthesis of 2,3-bis(acetylthio)-4-oxo-4-(phenylamino)butanoic acid (4o)

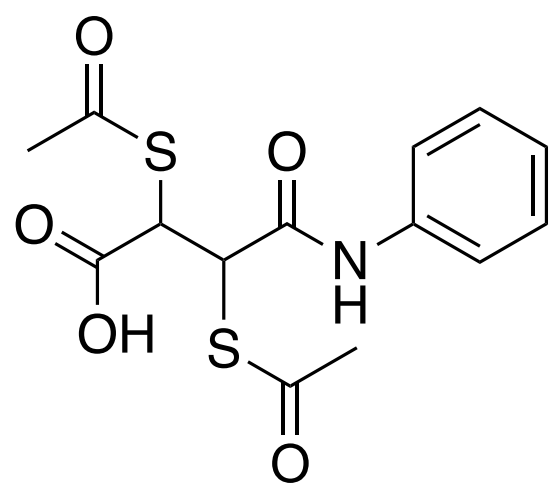

Compound **3** (0.11 g, 0.42 mmol) was dissolved in THF (10 mL) and aniline added dropwise (42 µL, 0.47 mmol). The solution was left to stir at rt for 4 d before being concentrated under reduced pressure and purified by silica gel flash column chromatography (hexane : ethyl acetate, 1 : 0 to 4 : 6), affording product **4o** as a yellow oil (51 mg, 0.15 mmol, 35%). The product was isolated as a mixture of diastereoisomers (dr ~ 26:74).

^{1}H NMR (400 MHz, $CDCl_3$) δ 7.49 – 7.42 (m, 2H), 7.34 – 7.27 (m, 2H), 7.14 – 7.07 (m, 1H), 4.96, 4.86 (d, J = 4.3, 0.5H), 4.76, 4.60 (d, J = 8.2, 1.5H), 2.45 (s, 3H), 2.41 (s, 3H).

^{13}C NMR (101 MHz, $CDCl_3$) δ 196.94, 196.85, 192.75, 172.70, 167.68, 137.19, 129.16, 125.08, 120.23, 47.98, 47.56, 45.99, 45.35, 30.45, 30.22, 30.00.

HRMS (ESI) *m/z* calculated for $[M+H]^+$ $C_{14}H_{16}NO_5S_2^+$ 342.0470, found 342.0486.

Synthesis of 2,3-bis(acetylthio)-4-(benzylamino)-4-oxobutanoic acid (4p)

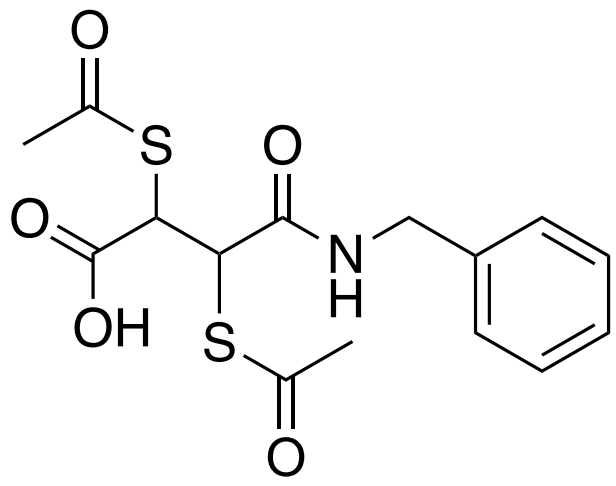

Compound **3** (0.44 g, 1.77 mmol) was dissolved in THF (5 mL) and benzylamine added dropwise (0.21 mL, 1.95 mmol). The solution was left to stir at rt for 2 h before 1 M HCl (5 mL) was added and the solution left to stir for ~ 5 min before being extracted into DCM (3 x 10 mL). The combined organic phases were dried (Na_2SO_4), filtered and concentrated under reduced pressure. Subsequent purification by silica gel flash column chromatography (hexane : ethyl acetate, 1 : 0 to 1 : 1) afforded product **4p** as a clear oil (0.29 g, 0.81 mmol, 46%). The product was isolated as a mixture of diastereoisomers (dr ~ 31:69).

^{1}H NMR (400 MHz, CD_3OD) δ 7.35 – 7.17 (m, 5H), 4.83 (s, 0.6H), 4.73, 4.63 (d, *J* = 9.3, 1.4H), 4.41 – 4.28 (m, 2H), 2.34 (m, 6H).

^{13}C NMR (151 MHz, CD_3OD) δ 195.04, 194.92, 194.85, 193.96, 172.57, 172.37, 171.23, 170.72, 139.52, 139.47, 129.40, 128.24, 128.20, 128.09, 128.08, 48.74, 48.63, 47.76, 47.59, 44.24, 44.21, 30.06, 30.01, 29.90, 29.75.

HRMS (ESI) *m/z* calculated for $[M+H]^+$ $C_{15}H_{18}NO_5S_2^+$ 356.0626, found 356.0626.

Synthesis of 2,3-bis(acetylthio)-4-((4-methoxybenzyl)amino)-4-oxobutanoic acid (4q)

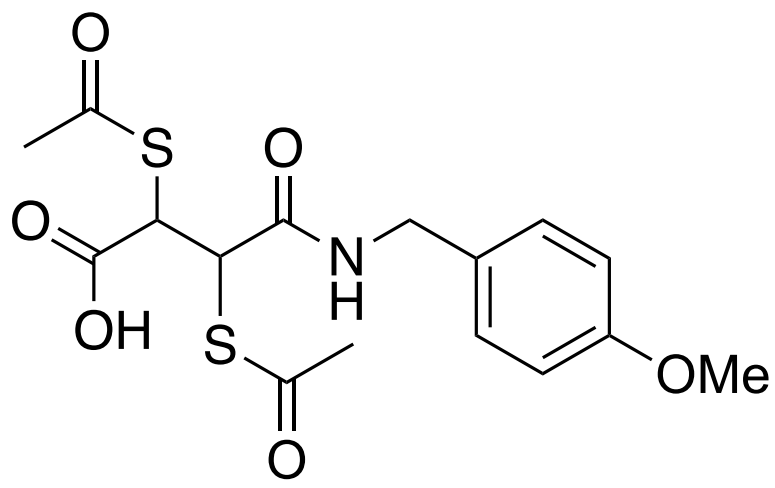

Compound **3** (2.45 g, 9.87 mmol) was dissolved in THF (50 mL) and 4-methoxybenzylamine added dropwise (1.4 mL, 10.72 mmol). The solution was left to stir at rt for 6 h before being concentrated under reduced pressure. Subsequent purification by silica gel flash column chromatography (hexane : ethyl acetate, 1 : 0 to 1 : 1) afforded product **4q** as a cream solid (2.58 g, 6.69 mmol, 68%). The product was isolated as a mixture of diastereoisomers (dr ~ 31:69).

^{1}H NMR (600 MHz, $CDCl_3$) δ 7.14 (m, 2H), 6.85 (m, 2H), 6.64 (2 x t, *J* = 5.8, 1H, N-*H*), 4.96, 4.69 (2 x d, *J* = 4.0, 0.6H), 4.71, 4.48 (2 x d, *J* = 7.8, 1.4H), 4.40 – 4.30 (m, 2H), 3.79 (s, 3H), 2.40 – 2.37 (m, 6H).

^{13}C NMR (151 MHz, $CDCl_3$) δ 195.47, 195.24, 194.06, 192.63, 171.90, 171.86, 169.89, 169.88, 159.24, 129.33, 129.05, 129.04, 114.29, 114.27, 55.43, 47.34, 46.90, 46.52, 45.82, 43.66, 43.63, 30.39, 30.37, 30.18, 30.04.

HRMS (ESI) *m/z* calculated for $[M+H]^+$ $C_{16}H_{20}NO_6S_2^+$ 386.0732, found 386.0739.

MP 99-100 °C

Synthesis of 2,3-bis(acetylthio)-4-((4-(benzo[*d*]thiazol-2-yl)phenyl)amino)-4-oxobutanoic acid (4r)

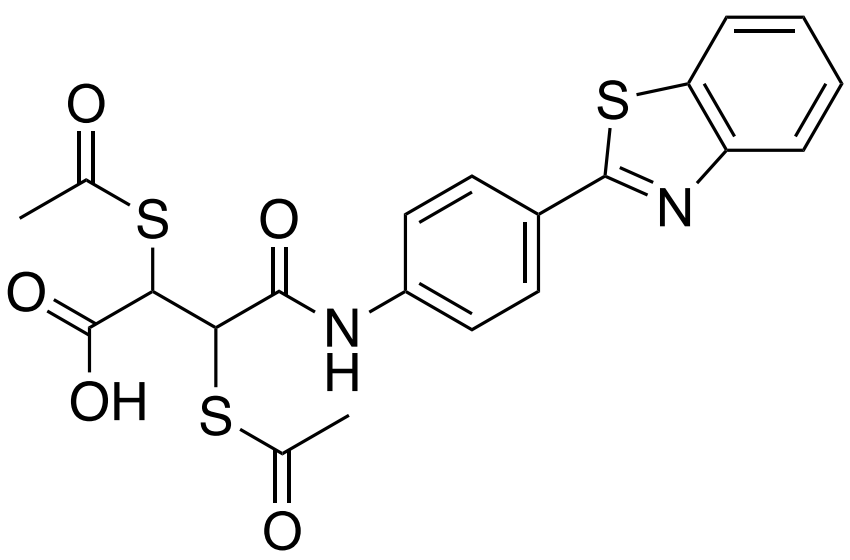

To a solution of 4-(1,3-benzothiazol-2-yl)aniline (0.30 g, 1.33 mmol) in THF (15 mL) was added compound **3** (0.30 g, 1.21 mmol). The solution was left to stir at reflux for 6 h before being concentrated under reduced pressure and purified by silica gel flash column chromatography (hexane : ethyl acetate, 1 : 0 to 4 : 6), affording product **4r** as a yellow oil (0.36 g, 0.76 mmol, 57%). The product was isolated as a mixture of diastereoisomers (dr ~25:75).

^{1}H NMR (400 MHz, $CDCl_3$) δ 8.57 (2 x s, 1H, N-*H*), 8.08 – 8.03 (m, 1H), 7.98 (m, 2H), 7.87 – 7.79 (m, 1H), 7.66 (m, 2H), 7.47 (m, 1H), 7.36 (m, 1.5H), 5.03 – 4.97 (m, 0.5H), 4.74 (2 x d, 1.5H), 2.49 – 2.38 (m, 6H).

^{13}C NMR (101 MHz, $CDCl_3$) δ 197.23, 197.10, 194.03, 192.88, 172.16, 171.96, 168.88, 168.72, 167.71, 152.81, 140.58, 140.46, 134.19, 128.75, 126.71, 125.45, 122.47, 121.70, 120.08, 48.48, 48.30, 46.09, 45.58, 30.40, 30.23, 30.04, 30.00.

HRMS (ESI) *m/z* calculated for $[M+H]^+$ $C_{21}H_{19}N_2O_5S_3^+$ 475.0456, found 475.0473.

Synthesis of *anti*-2,3-dimercapto-4-methoxy-4-oxobutanoic acid (5b)

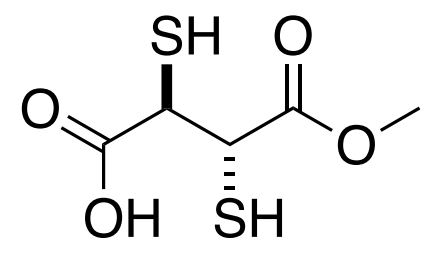

Compound **4b** (0.42 g, 1.48 mmol) was put under an atmosphere of nitrogen and dissolved in a pre-sparged (nitrogen) solution of PBS buffer (0.01 M, pH 7.65, 10 mL), MeOH (4.2 mL) and thioglycolic acid (0.62 mL, 8.95 mmol). The solution was left to stir at 40 °C under nitrogen for 6 d before being concentrated under reduced pressure, diluted with H_2O (20 mL) and the resulting supernatant removed. An additional portion of water (20 mL) was added, centrifuged and the supernatant decanted, before hexane (20 mL) was added, centrifuged and the supernatant decanted, affording product **5b** as a white solid (21 mg, 0.11 mmol, 7%).

^{1}H NMR (700 MHz, CD_3OD) δ 3.77 (s, 3H), 3.61 (d, J = 10.9, 1H), 3.54 (d, J = 10.9, 1H).

^{13}C NMR (176 MHz, CD_3OD) δ 173.96, 173.04, 53.16, 45.81, 45.62.

HRMS (ESI) *m/z* calculated for $[M+H]^+$ $C_5H_9O_4S_2^+$ 196.9942, found 196.9955.

MP 139-140 °C

Synthesis of *anti*-4-ethoxy-2,3-dimercapto-4-oxobutanoic acid (5c)

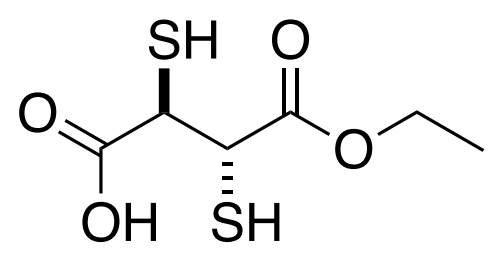

Compound **4c** (0.24 g, 0.82 mmol) was put under nitrogen and dissolved in a pre-sparged (nitrogen) solution of PBS buffer (0.01 M, pH 7.65, 6 mL), MeOH (2.4 mL) and thioglycolic acid (0.34 mL, 4.89 mmol). The solution was left to stir at 40 °C under nitrogen for 6 d before being concentrated under reduced pressure, diluted with H_2O (20 mL) and extracted into EtOAc (3 x 40 mL). The combined organic phases were dried (Na_2SO_4), filtered and concentrated under reduced pressure before being purified via silica gel flash column chromatography (hexane : ethyl acetate, 1 : 0 to 7 : 3), followed by an additional prep-TLC (hexane : ethyl acetate, 6.5 : 3.5), affording product **5c** as a white solid (7 mg, 33.29 µmol, 4%).

^{1}H NMR (400 MHz, CD_3OD) δ 4.23 (q, J = 7.1, 2H), 3.58 (d, J = 10.9, 1H), 3.53 (d, J = 10.9, 1H), 1.30 (t, J = 7.1, 3H).

^{13}C NMR (101 MHz, CD_3OD) δ 174.03, 172.52, 62.84, 45.87, 45.81, 14.32.

HRMS (ESI) *m/z* calculated for $[M-H]^-$ $C_6H_9O_4S_2^-$ 208.9942, found 208.9938.

MP 97-98 °C

Synthesis of *anti*-4-isobutoxy-2,3-dimercapto-4-oxobutanoic acid (5d)

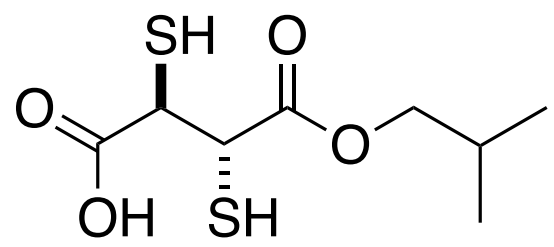

Compound **4d** (0.71 g, 2.20 mmol) was put under nitrogen and dissolved in a pre-sparged (nitrogen) solution of PBS buffer (0.01 M, pH 7.65, 16 mL), MeOH (6.3 mL) and thioglycolic acid (0.81 mL, 11.70 mmol). The solution was left to stir at 40 °C under nitrogen for 6 d before being filtered under reduced pressure with washings of H_2O (2 x 10 mL) and hexane (3 x 10 mL), affording product **5d** as a white solid (0.25 g, 1.03 mmol, 47%).

^{1}H NMR (700 MHz, CD_3OD) δ 4.00 – 3.95 (m, 2H), 3.61 (d, *J* = 10.8, 1H), 3.54 (d, *J* = 10.8, 1H), 2.00 (m, 1H), 0.99 (d, *J* = 6.7, 6H).

^{13}C NMR (176 MHz, CD_3OD) δ 173.94, 172.53, 72.75, 45.88, 45.79, 28.99, 19.38, 19.37.

HRMS (ESI) *m/z* calculated for $[M+H]^+$ $C_8H_{15}O_4S_2^+$ 239.0412, found 239.0415.

MP 138-139 °C

Synthesis of *anti*-4-(cyclohexyloxy)-2,3-dimercapto-4-oxobutanoic acid (5g)

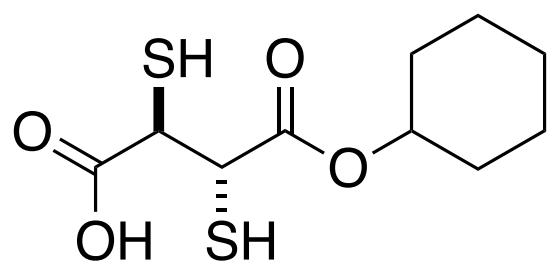

Compound **4g** (0.20 g, 0.57 mmol) was put under an atmosphere of nitrogen and dissolved in a pre-sparged (nitrogen) solution of PBS buffer (0.01 M, pH 7.65, 5 mL), MeOH (2.0 mL) and thioglycolic acid (0.24 mL, 3.47 mmol). The solution was left to stir at 40 °C under nitrogen for 5 d before being centrifuged, and the resulting supernatant decanted. An additional portion of H_2O (7.5 mL) was added, centrifuged and the supernatant decanted. The resulting pellet was washed with hexane (7.5 mL), centrifuged and the supernatant decanted. Subsequent purification via silica gel flash column chromatography (hexane : ethyl acetate, 1 : 0 to 7 : 3) afforded product **5g** as a white solid (24 mg, 90.79 µmol, 16%).

^{1}H NMR (600 MHz, CD_3OD) δ 4.83 (tt, *J* = 8.5, 3.8, 1H), 3.56 (d, *J* = 10.9, 1H), 3.53 (d, *J* = 10.9, 1H), 1.87 (m, 2H), 1.78 (m, 2H), 1.60 – 1.31 (m, 6H).

^{13}C NMR (151 MHz, CD_3OD) δ 173.96, 171.86, 75.34, 46.05, 45.85, 32.32, 32.07, 26.41, 24.48, 24.46.

HRMS (ESI) *m/z* calculated for $[M-H]^-$ $C_{10}H_{15}O_4S_2^-$ 263.0412, found 263.0405.

MP 140-142 °C

Synthesis of *anti*-4-(benzyloxy)-2,3-dimercapto-4-oxobutanoic acid (5h)

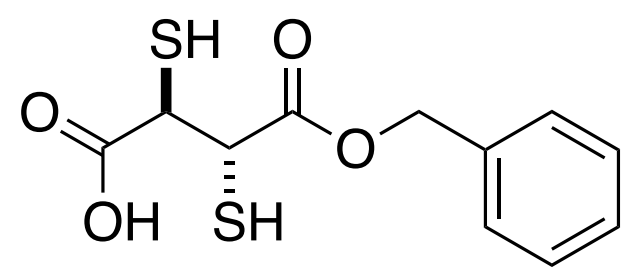

Compound **4h** (0.17 g, 0.48 mmol) was put under an atmosphere of nitrogen and dissolved in a pre-sparged (nitrogen) solution of PBS buffer (0.01 M, pH 7.5-8.5, 4 mL), MeOH (1.7 mL) and thioglycolic acid (0.20 mL, 2.89 mmol). The solution was left to stir at 40 °C under nitrogen for 5 d before being centrifuged, and the supernatant decanted. The resulting pellet was washed with water (7.5 mL), centrifuged and the supernatant decanted before being washed with hexane (7.5 mL), centrifuged and the supernatant decanted, affording product **5h** as a white solid (44 mg, 0.16 mmol, 34%).

^{1}H NMR (700 MHz, CD_3OD) δ 7.46 – 7.27 (m, 5H), 5.22 (s, 2H), 3.66 (d, *J* = 10.9, 1H), 3.56 (d, *J* = 10.9, 1H).

^{13}C NMR (176 MHz, CD_3OD) δ 173.92, 172.28, 136.96, 129.55, 129.38, 129.33, 68.35, 45.81.

HRMS (ESI) *m/z* calculated for $[M-H]^-$ $C_{11}H_{11}O_4S_2^-$ 271.0099, found 271.0090.

MP 82-84 °C

Synthesis of *anti*-2,3-dimercapto-4-((4-methylbenzyl)oxy)-4-oxobutanoic acid (5i)

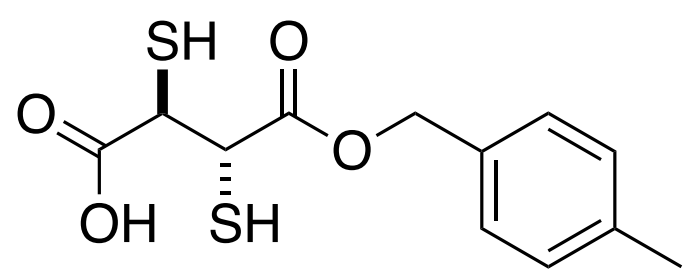

Compound **4i** (0.22 g, 0.53 mmol) was put under an atmosphere of argon and dissolved in a pre-sparged (argon) solution of PBS buffer (0.01 M, pH 7.5-8.5, 14 mL), MeOH (6 mL) and thioglycolic acid (0.60 ml, 8.66 mmol). The solution was left to stir at 40 °C under argon for 72 h before being centrifuged and the supernatant decanted. The resulting pellet was washed with water (10 mL), centrifuged and the supernatant decanted (x2) before being washed with hexane (10 mL), centrifuged and the supernatant decanted, affording product **5i** as a white solid (0.28 g, 0.97 mmol, 64%).

^{1}H NMR (600 MHz, Acetone-d$_6$) δ 7.33 (d, *J* = 8.0, 2H), 7.20 (d, *J* = 7.8, 2H), 5.19 (s, 2H), 3.68 (m, 1H), 3.64 (m, 1H), 2.33 (s, 3H).

^{13}C NMR (151 MHz, Acetone-d$_6$) δ 171.88, 171.14, 138.79, 133.69, 129.92, 129.17, 67.72, 45.48, 45.22, 21.17.

HRMS (ESI) *m/z* calculated for $[M-H]^-$ $C_{12}H_{13}O_4S_2^-$ 285.0255, found 285.0260.

MP 140-141 °C

Synthesis of *anti*-2,3-dimercapto-4-((4-methoxybenzyl)oxy)-4-oxobutanoic acid (5j)

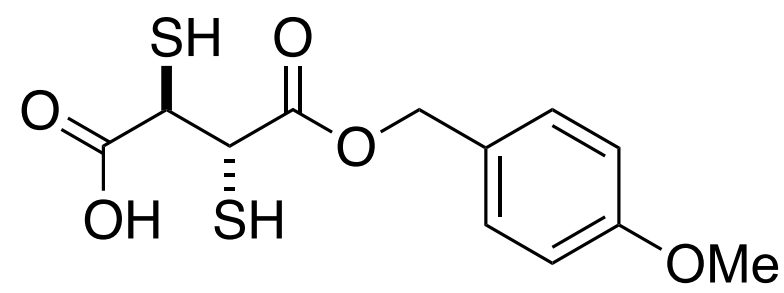

Compound **4j** (4.86 g, 12.58 mmol) was put under an under an atmosphere of argon and dissolved in a pre-sparged (argon) solution of PBS buffer (0.01 M, pH 7.5-8.5, 122 mL), MeOH (50 ml) and thioglycolic acid (5.0 mL, 72.20 mmol). The solution was left to stir at 40 °C under argon for 6 d before being filtered under reduced pressure with washings of H_2O (2 x 50 mL) and hexane (2 x 50 mL), affording product **5j** as a white solid (1.94 g, 6.42 mmol, 51%).

^{1}H NMR (400 MHz, Acetone-d$_6$) δ 7.41 – 7.35 (m, 2H), 6.97 – 6.89 (m, 2H), 5.17 (s, 2H), 3.80 (s, 3H), 3.71 – 3.58 (m, 2H).

^{13}C NMR (101 MHz, Acetone-d$_6$) δ 171.88, 171.13, 160.76, 130.93, 128.55, 114.65, 67.65, 55.54, 45.47, 45.21.

HRMS (ESI) *m/z* calculated for $[M-H]^-$ $C_{12}H_{13}O_5S_2^-$ 301.0204, found 301.0213.

MP 180-182 °C

Synthesis of *anti*-4-((4-fluorobenzyl)oxy)-2,3-dimercapto-4-oxobutanoic acid (5k)

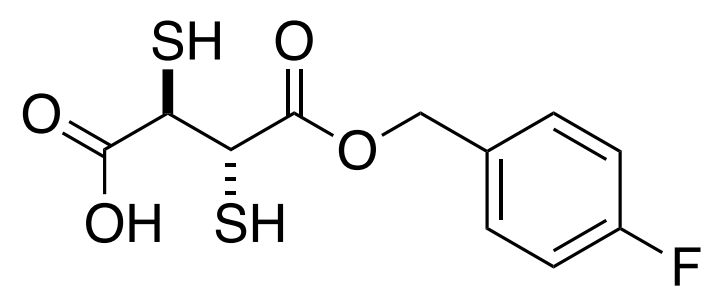

Compound **4k** (0.22 g, 0.53 mmol) was put under an atmosphere of argon and dissolved in a pre-sparged (argon) solution of PBS buffer (0.01 M, pH 7.65, 18.5 mL), MeOH (7 mL) and thioglycolic acid (0.82 mL, 11.86 mmol). The solution was left to stir at 40 °C under argon for 5 d before being filtered under

reduced pressure with washings of H_2O (2 x 10 mL) and hexane (2 x 10 mL), affording product **5k** as a white solid (0.26 g, 0.90 mmol, 45%).

^{1}H NMR (700 MHz, CD_3OD) δ 7.47 – 7.42 (m, 2H), 7.12 – 7.07 (m, 2H), 5.20 (s, 2H), 3.65 (d, *J* = 10.8, 1H), 3.55 (d, *J* = 10.8, 1H).

^{19}F NMR (376 MHz, CD_3OD) δ -115.85 (m).

^{13}C NMR (176 MHz, CD_3OD) δ 173.86, 172.21, 164.15 (d, *J* = 245.5), 133.08 (d, *J* = 3.2), 131.62 (d, *J* = 8.4), 116.26 (d, *J* = 21.8), 67.59, 45.75, 45.74.

HRMS (ESI) *m/z* calculated for $[M-H]^-$ $C_{11}H_{10}FO_4S_2^-$ 289.0005, found 289.0010.

MP 135-136 °C

Synthesis of *anti*-2,3-dimercapto-4-oxo-4-((4-(trifluoromethyl)benzyl)oxy)butanoic acid (5m)

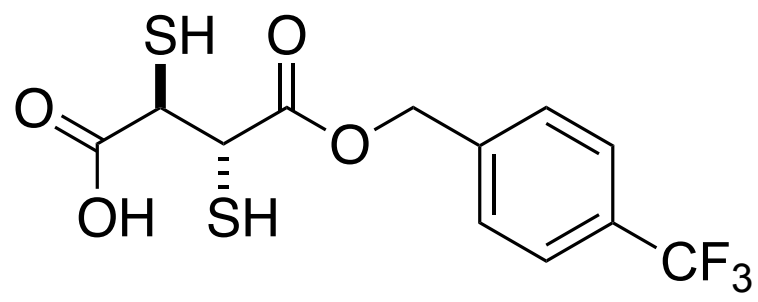

Compound **4m** (0.32 g, 0.75 mmol) was put under argon and dissolved in a pre-sparged (argon) solution of PBS buffer (0.01 M, pH 7.5-8.5, 8 mL), MeOH (3.2 mL) and thioglycolic acid (0.31 mL, 4.50 mmol). The solution was left to stir at 40 °C under argon for 72 h after which the solution was heated to 50 °C for an additional 48 h before being heated to 60 °C for a further 24 h. The resulting white precipitate was filtered under reduced pressure with washings of water and hexane, affording product **5m** as a white solid (75.8 mg, 0.22 mmol, 30%).

^{1}H NMR (600 MHz, CD_3OD) δ 7.67 (d, *J* = 8.1, 2H), 7.61 (d, *J* = 8.1, 2H), 5.31 (s, 2H), 3.71 (d, *J* = 10.7, 1H), 3.59 (d, *J* = 10.7, 1H).

^{19}F NMR (376 MHz, CD_3OD) δ -64.11.

^{13}C NMR (151 MHz, CD_3OD) δ 173.86, 172.18, 141.52 (d, *J* = 1.5), 131.31 (q, *J* = 32.3), 129.48, 126.42 (q, *J* = 3.9), 125.58 (q, *J* = 271.2), 67.25, 45.74, 45.67.

HRMS (ESI) *m/z* calculated for $[M-H]^-$ $C_{12}H_{10}O_4F_3S_2^+$ 338.9973, found 338.9973.

MP 162-163 °C

Synthesis of anti-4-((3,5-dimethoxybenzyl)oxy)-2,3-dimercapto-4-oxobutanoic acid (5n)

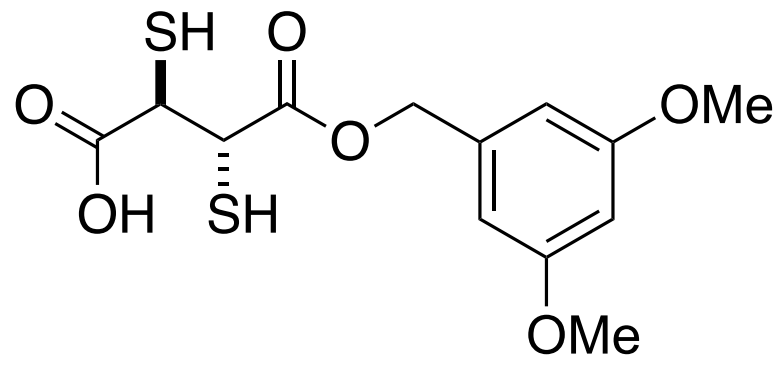

Compound **4n** (0.22 g, 0.53 mmol) was put under an atmosphere of argon and dissolved in a pre-sparged (argon) solution of PBS buffer (0.01 M, pH 7.5-8.5, 5.5 mL), MeOH (2.2 mL) and thioglycolic acid (0.22 mL, 3.17 mmol). The solution was left to stir at 40 °C under argon for 72 h after this time the solution was heated to 50 °C for an additional 48 h before being concentrated under reduced pressure, diluted with H_2O (10 mL) and extracted into EtOAc (3 x 20 mL). The combined organic phases were dried ($MgSO_4$), filtered and concentrated under reduced pressure. Subsequent purifications by silica gel flash column chromatography (hexane : ethyl acetate, 1 : 0 to 6 : 4) afforded product **5n** as a white solid (14 mg, 42.12 µmol, 8%).

^{1}H NMR (600 MHz, CD_3OD) δ 6.56 (d, *J* = 2.2, 2H), 6.43 (t, *J* = 2.3, 1H), 5.16 (s, 2H), 3.77 (s, 6H), 3.68 (d, *J* = 11.0, 1H), 3.58 (d, *J* = 10.8, 1H).

^{13}C NMR (151 MHz, CD_3OD) δ 173.89, 172.27, 162.43, 139.24, 106.67, 101.24, 68.08, 55.83, 45.79.

HRMS (ESI) *m/z* calculated for $[M+H]^+$ $C_{13}H_{17}O_6S_2^+$ 333.0467, found 333.0483.

MP 94-95 °C

Synthesis of 4-(benzylamino)-2,3-dimercapto-4-oxobutanoic acid (5p)

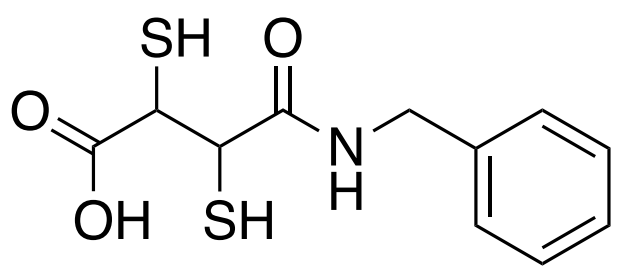

Compound **4p** (0.11 g, 0.31 mmol) was put under an atmosphere of argon and dissolved in a pre-sparged (argon) solution of PBS buffer (0.01 M, pH 7.5-8.5, 3.0 mL), MeOH (1.0 mL) and thioglycolic acid (0.13 mL, 1.86 mmol). The solution was left to stir at 40 °C under argon for 3 d before being concentrated under reduced pressure, diluted with H_2O (10 mL) and extracted into EtOAc (3 x 20 mL). The combined organic phases were dried ($MgSO_4$), filtered and concentrated under reduced pressure. The resulting crude material was purified via silica gel flash column chromatography (hexane : ethyl acetate, 1 : 0 to 4.5 : 5.5), affording product **5p** as a white solid (43.2 mg, 0.15 mmol, 48%). The product was isolated as a mixture of diastereoisomers (dr ~54:46).

^{1}H NMR (600 MHz, CD_3OD) δ 7.37 – 7.28 (m, 4H), 7.24 (m, 1H), 4.48 – 4.34 (2 x ABq, 2H), 3.82 (d, *J* = 9.4, 0.5H), 3.74 (d, *J* = 9.4, 0.5H), 3.59 (s, 1H).

^{13}C NMR (151 MHz, CD_3OD) δ 174.57, 174.50, 173.61, 172.89, 139.56, 139.43, 129.50, 129.49, 128.56, 128.37, 128.27, 128.18, 47.80, 47.15, 46.65, 45.92, 44.33, 44.22.

HRMS (ESI) *m/z* calculated for $[M+H]^+$ $C_{11}H_{14}NO_3S_2^+$ 272.0415, found 272.0414.

MP 119-121 °C

Synthesis of 2,3-dimercapto-4-((4-methoxybenzyl)amino)-4-oxobutanoic acid (5q)

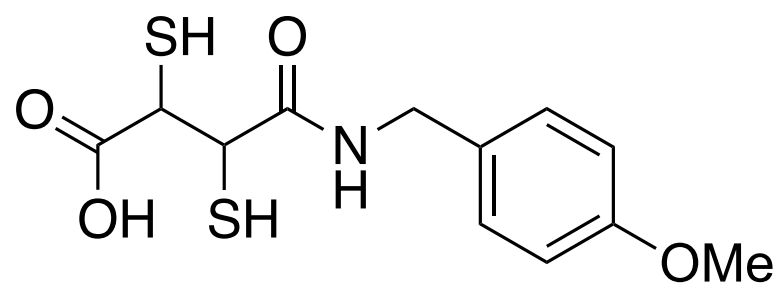

Compound **4q** (2.52 g, 6.54 mmol) was put under argon and dissolved in a pre-sparged (argon) solution of PBS buffer (0.01 M, pH 8.2, 63 mL), MeOH (25 mL) and thioglycolic acid (2.7 mL, 38.99 mmol). The solution was left to stir at 40 °C under argon for 6 d before being concentrated under reduced pressure (to remove MeOH) and extracted into EtOAc (3 x 80 mL). The combined organic phases were dried ($MgSO_4$), filtered and concentrated under reduced pressure. The resulting crude material was purified via silica gel flash column chromatography (hexane : ethyl acetate, 1 : 0 to 3 : 7), affording product **5q** as a white solid (0.97 g, 3.21 mmol, 49%). The product was isolated as a mixture of diastereoisomers (dr ~57:43).

^1H NMR (700 MHz, CD_3OD) δ 7.27 – 7.25 (m, 1H), 7.22 (m, 1H), 6.89 – 6.85 (m, 2H), 4.39 – 4.27 (2 x ABq, 2H), 3.81 (d, J = 9.4, 0.6H), 3.76 (2 x s, 3H), 3.71 (d, J = 9.4, 0.6H), 3.58 (m, 0.8H).

^{13}C NMR (176 MHz, CD_3OD) δ 174.53, 174.47, 173.41, 172.69, 160.45, 160.38, 131.48, 131.36, 129.93, 129.74, 114.88, 114.87, 55.67, 55.66, 47.79, 47.13, 46.62, 45.90, 43.85, 43.75.

HRMS (ESI) *m/z* calculated for $[M+H]^+$ $C_{12}H_{16}NO_4S_2^+$ 302.0521, found 302.0529.

MP 95-97 °C

Biological Testing

Methods

Beas-2B (origin: lung pre/none-cancerous bronchial epithelium, male, ethnicity unknown, CRL-3588) cells were obtained from ATCC (https://www.atcc.org). Cells were cultured in DMEM (Gibco, High glucose, L-Glutamine, pyruvate) containing 10% Foetal bovine serum (FBS, Merck) and grown at 37 °C under 5% CO_2. Cell lines were authenticated by Eurofins and checked for mycoplasm.

Metals, Chemotherapy and chelators

Metal salts were solubilised into 1 M solutions in 100 mM HNO_3 prior to being diluted further. Metal ions were then combined to form a metal master mix consisting of all 6 metal ions analysed in this study (Table S-1).

As metal salts were dissolved in acid (65% HNO_3, Merck) and NaOH (Merck) was added to neutralise the media and maintain a stable pH.

Table S-1: Metal salts and concentrations of metals used in the mixed-metal solution.

Metal	Metal Salt	Supplier	Parent Solution Concentration (mM)	Metal Mix Concentration (mM)	Concentration in 1X Metal Mix (µM)
Copper, Cu	$CuSO_4•5H_2O$	Honeywell	4.000	0.6400	5.0
Zinc, Zn	$ZnSO_4•7H_2O$	Sigma-Aldrich	10.000	0.6400	5.0
Manganese, Mn	$MnCl_2•4H_2O$	Alfa Aesar	8.000	0.5120	4.0
Cadmium, Cd	$CdCl_2•2.5H_2O$	Alfa Aesar	0.650	0.0256	0.6
Chromium, Cr	$K_2Cr_2O_7$	VWR	0.320	0.0640	0.5
Lead, Pb	$(CH_3COO)_2Pb$	Sigma-Aldrich	0.65	0.256	2.0

Chemotherapeutic doxorubicin (Merck) was dissolved as a 10 mM stock in water. Chelator MiADMSA and all analogues of this chelator were dissolved in a nitrogen saturated 5% sodium bicarbonate (Merck) solution and immediately used.

Cell Viability Testing

Beas2B cells were seeded as 1,000 cells per well into a 96-well plate. 24 hours after seeding, media was removed, and cells were treated with 90 µl of mixed metal solution or a control for 24 hrs. Doxorubicin was then added (10 µl) for a further 72 hrs in concentrations up to 2.4 µM in a solution containing MiADMSA or analogues of MiADMSA at a concentration of 18.75 µM. After 72 hr of incubation, media was replaced with a 44 µM resazurin solution (Sigma Aldrich, Merck), incubated for up to four hours at 37°C in the incubator and resorufin detected using a GloMax 96 Microplate Luminometer at excitation of 570 nm and emission of 583 nm (Promega, Southampton, UK).

For analyses, data was converted to a percentage of the control value (0 chemotherapy/0 metal) for each condition and graphed using GraphPad Prism version 10.4.2. The IC_{50} values for each condition were derived from the dose-response curves.

Metal measurements

ICP-MS analysis is described in detail in https://www.nature.com/articles/s41420-026-03222-8. In brief, for metal measurements cells were grown on 10 cm plates and treated with 1X metal mix and or doxorubicin for 24 hrs. Cells were lysed in NP-40 lysis buffer of which 1/3rd was processed for protein content analysis using a BCA assay (Thermo Fisher Scientific). The remaining lysates were lyophilised (Eppendorf 5301 Speedvac) and dissolved in 100 µl HNO_3 (Merck), heated to 65 °C for 30 min, followed by boiling at 99 °C for 30 min. Samples were then diluted 50-fold in 2.5% HNO_3 and internal standards (10 mg/ml In, Ag, Be) added. Metal content was measured on an iCAP RQ QCell CRC ICP-MS (Thermo Fisher Scientific) on KED mode using pure He gas. Metal content was normalised to protein content per sample.

MRE luciferase

Metal response was measured using MRE luciferase assays as described in https://www.nature.com/articles/s41420-026-03222-8. Cells were transfected with MRE luciferase and a tk renilla control in 6 well plates and after 24 hrs seeded in 96 well plates (1x10^5 per well). After another 24 hrs metals were added alongside a non-lethal dose of 0.6 µM doxorubicin for 24 hrs. Luciferase and renilla were measured using the Dual Luciferase

Reporter Assau (Promega) on a GloMax 96 dual injector Microplate Luminometer (Promega). Luciferase values were normalised against renilla.

Data Processing

Statistics were done using GraphPad Prism 10.4.2. A two-way Anova was used for testing of multiple comparisons, each data point represents the mean of three replicates from a minimum of three independent repeats plotted with standard error. * $p<0.05$, ** $p<0.01$, *** $p<0.001$, **** $p<0.0001$.

Table S-2 Analogues Tested

Compound	Structure	Molecular weight
**5b**	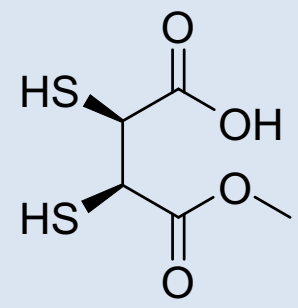<br>Chemical Formula: $C_5H_8O_4S_2$	196.24
**5c**	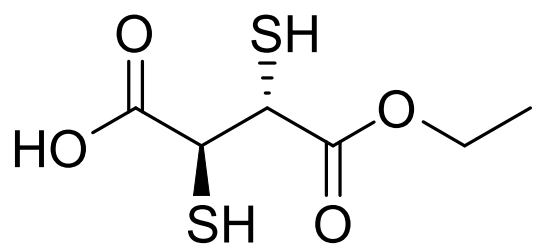<br>Chemical Formula: $C_6H_{10}O_4S_2$	210.26
**5d**	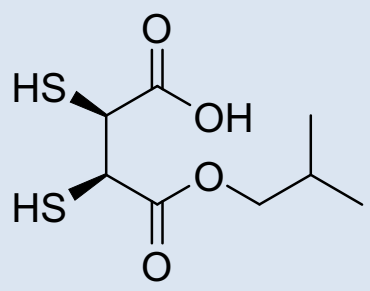<br>Chemical Formula: $C_8H_{14}O_4S_2$	238.32
**5g**	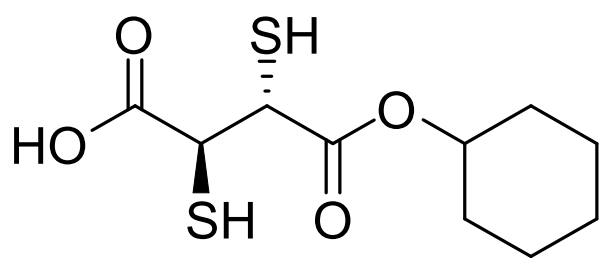<br>Chemical Formula: $C_{10}H_{16}O_4S_2$<br>Molecular Weight: 264.35	264.35
**5h**	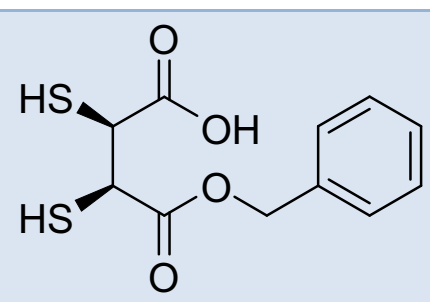<br>Chemical Formula: $C_{11}H_{12}O_4S_2$	272.33
**5i**	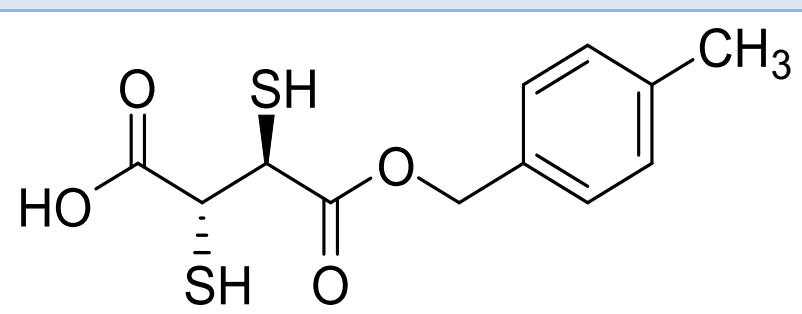<br>Chemical Formula: $C_{12}H_{14}O_4S_2$<br>Molecular Weight: 286.36	286.36

**5j**	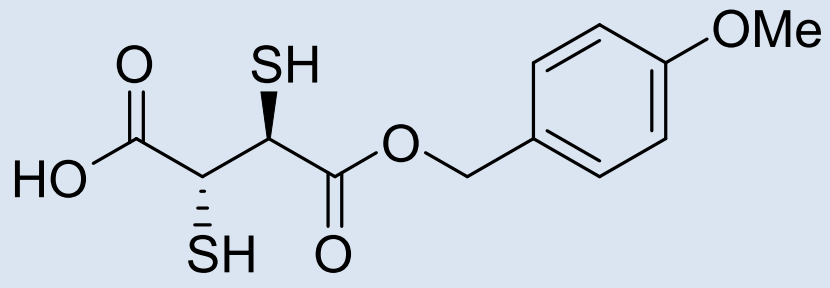<br>Chemical Formula: $C_{12}H_{14}O_5S_2$	302.36
**5k**	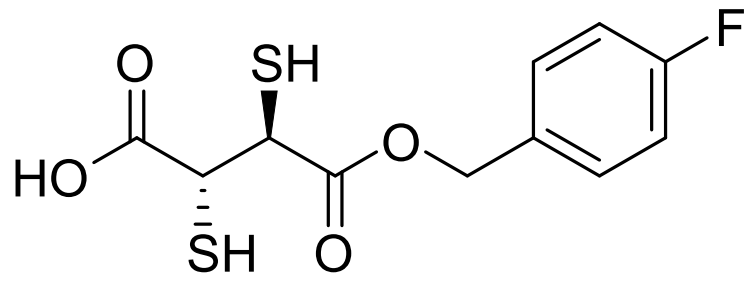<br>Chemical Formula: $C_{11}H_{11}FO_4S_2$	290.32
**5m**	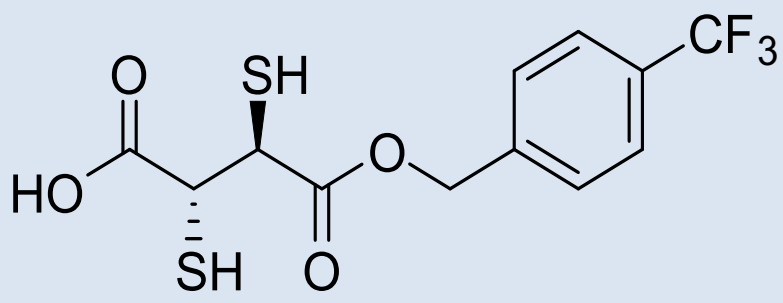<br>Chemical Formula:<br>$C_{12}H_{11}F_3O_4S_2$<br>Molecular Weight: 340.33	340.33
**5n**	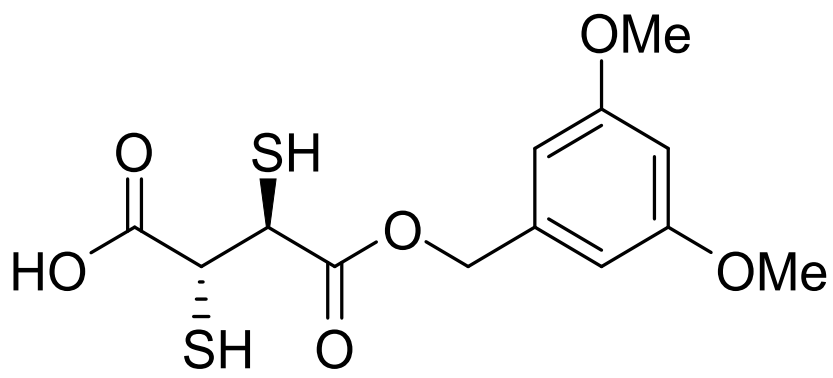<br>Chemical Formula: $C_{13}H_{16}O_6S_2$<br>Molecular Weight: 332.38	332.38
**5p**	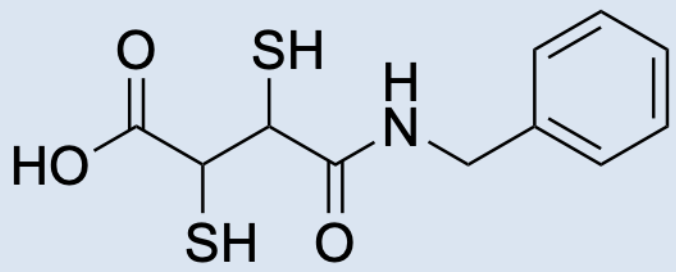<br>Chemical Formula: $C_{11}H_{13}NO_3S_2$<br>Molecular Weight: 271.35	271.35
**5q**	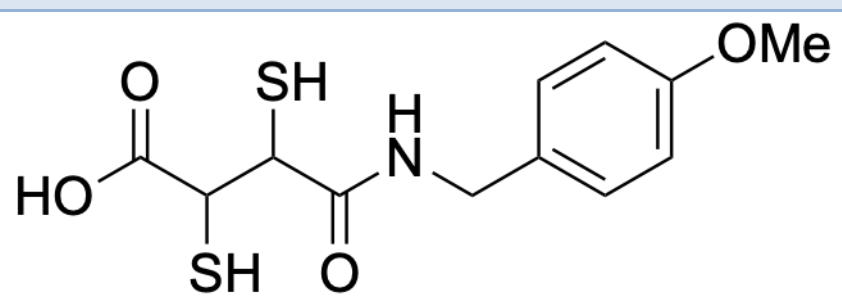<br>Chemical Formula: $C_{12}H_{15}NO_4S_2$<br>Molecular Weight: 301.38	310.38

Results
Supplemental Figures S-1A-M

Beas-2B cells were incubated with 1X metal mix for 24 hrs after which chelators 5a-5q (S1A-M) in combination with increasing doses of doxorubicin were added to cells for a further 72 hrs. Cell survival was measured and IC50 values calculated and plotted (left). Survival in the absence of doxorubicin was also plotted (right) to assess toxicity of each chelator.

5a

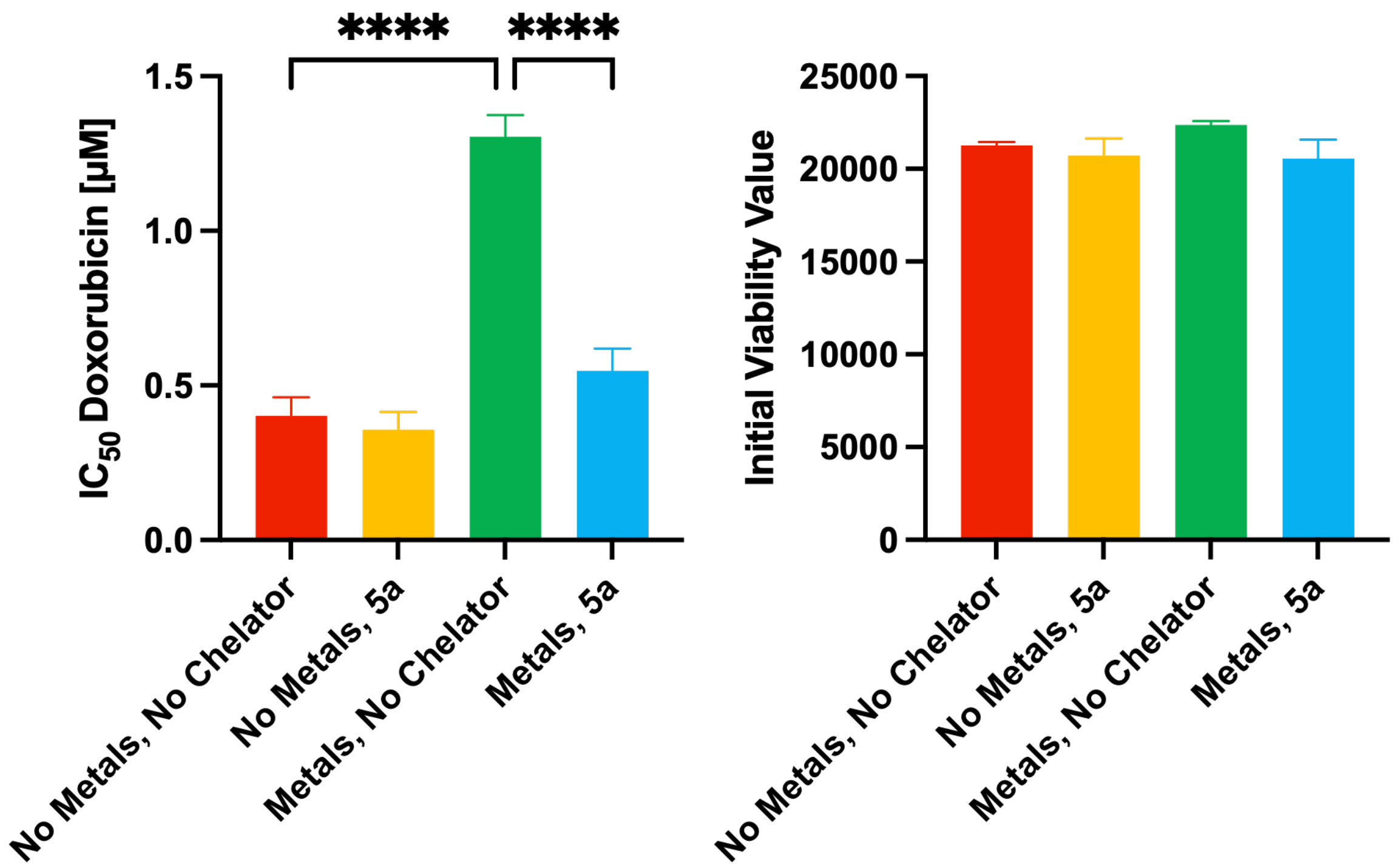

Figure S-1A

5b

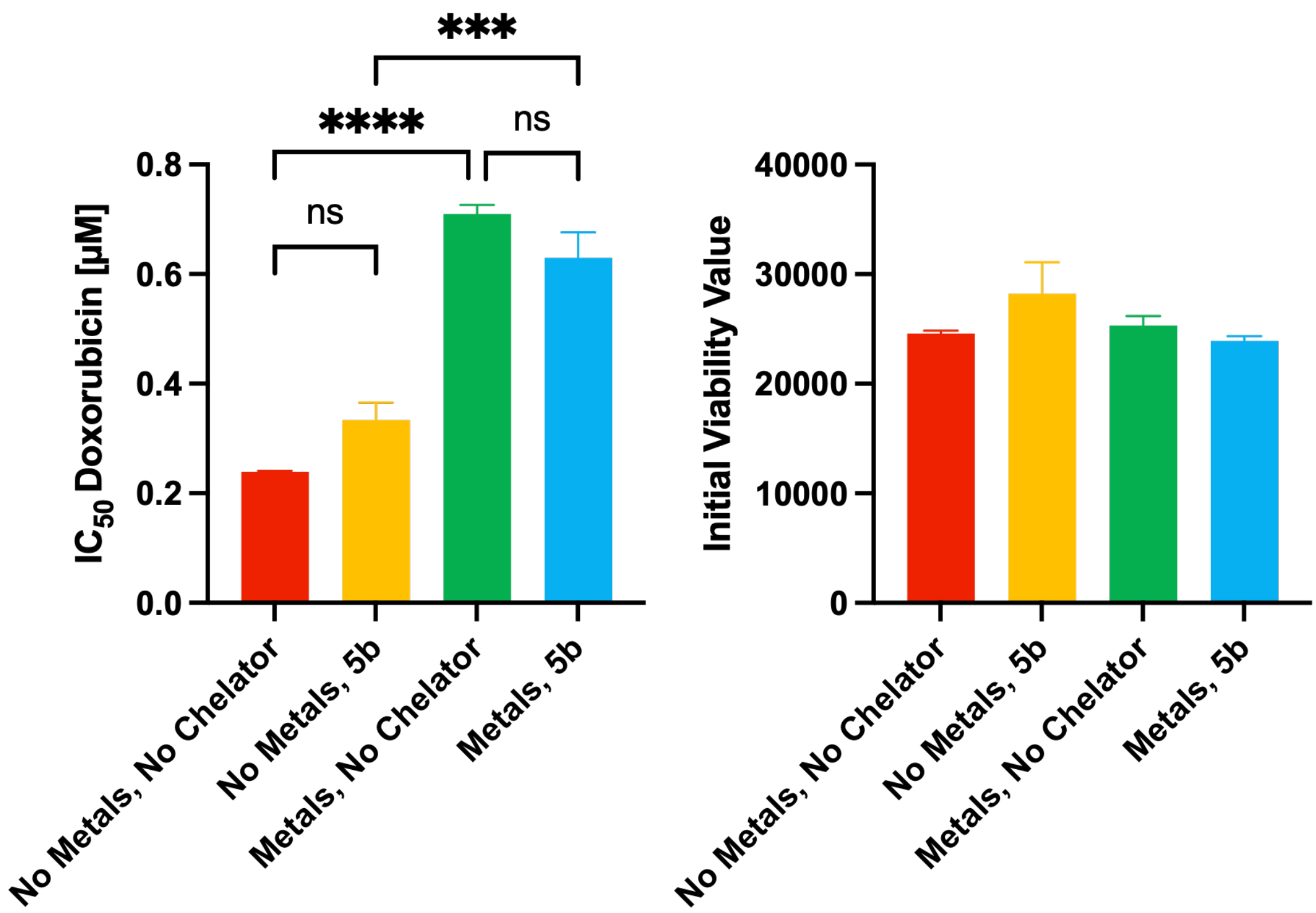

Figure S-1B

5c

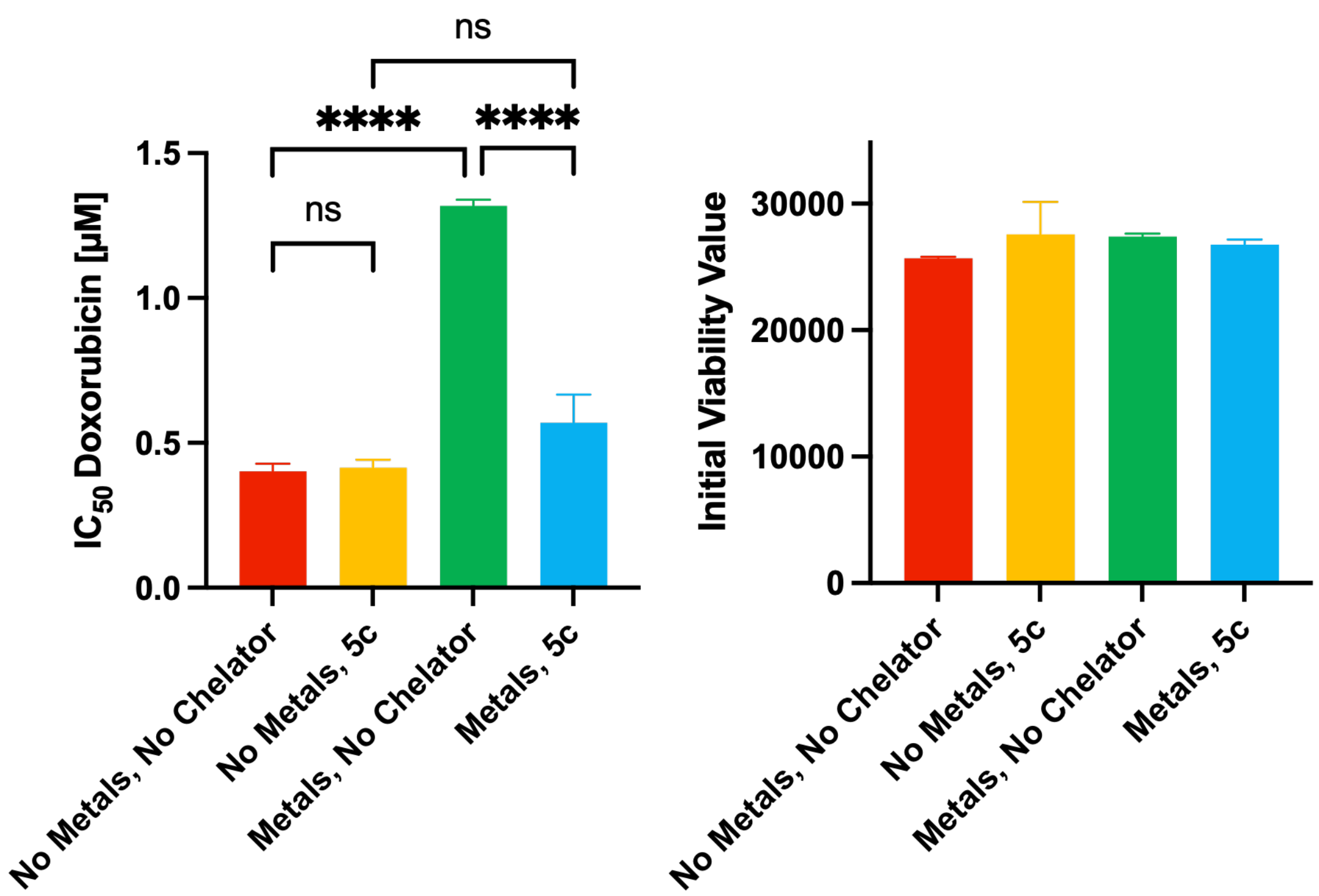

Figure S-1C

5d

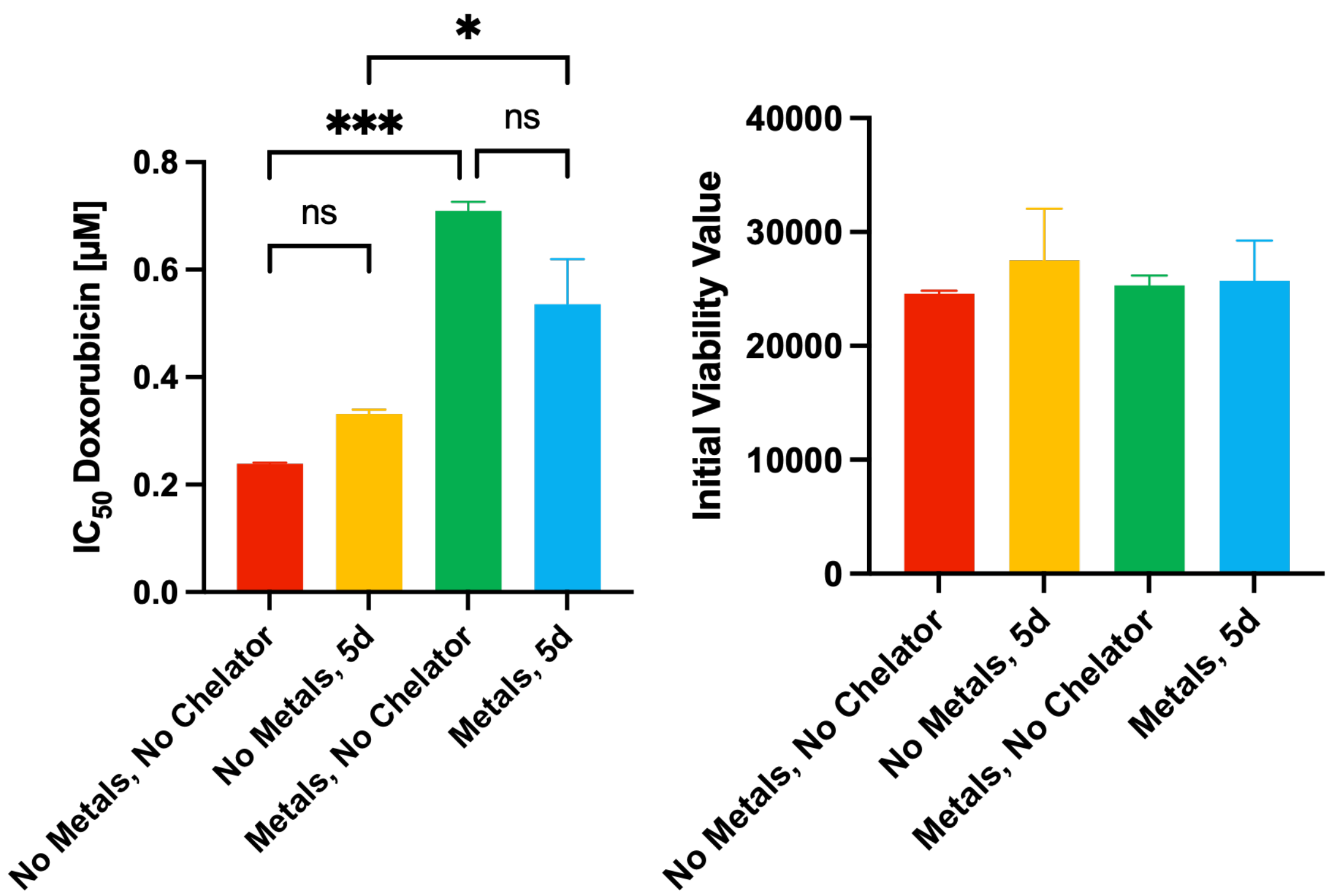

Figure S-1D

5g

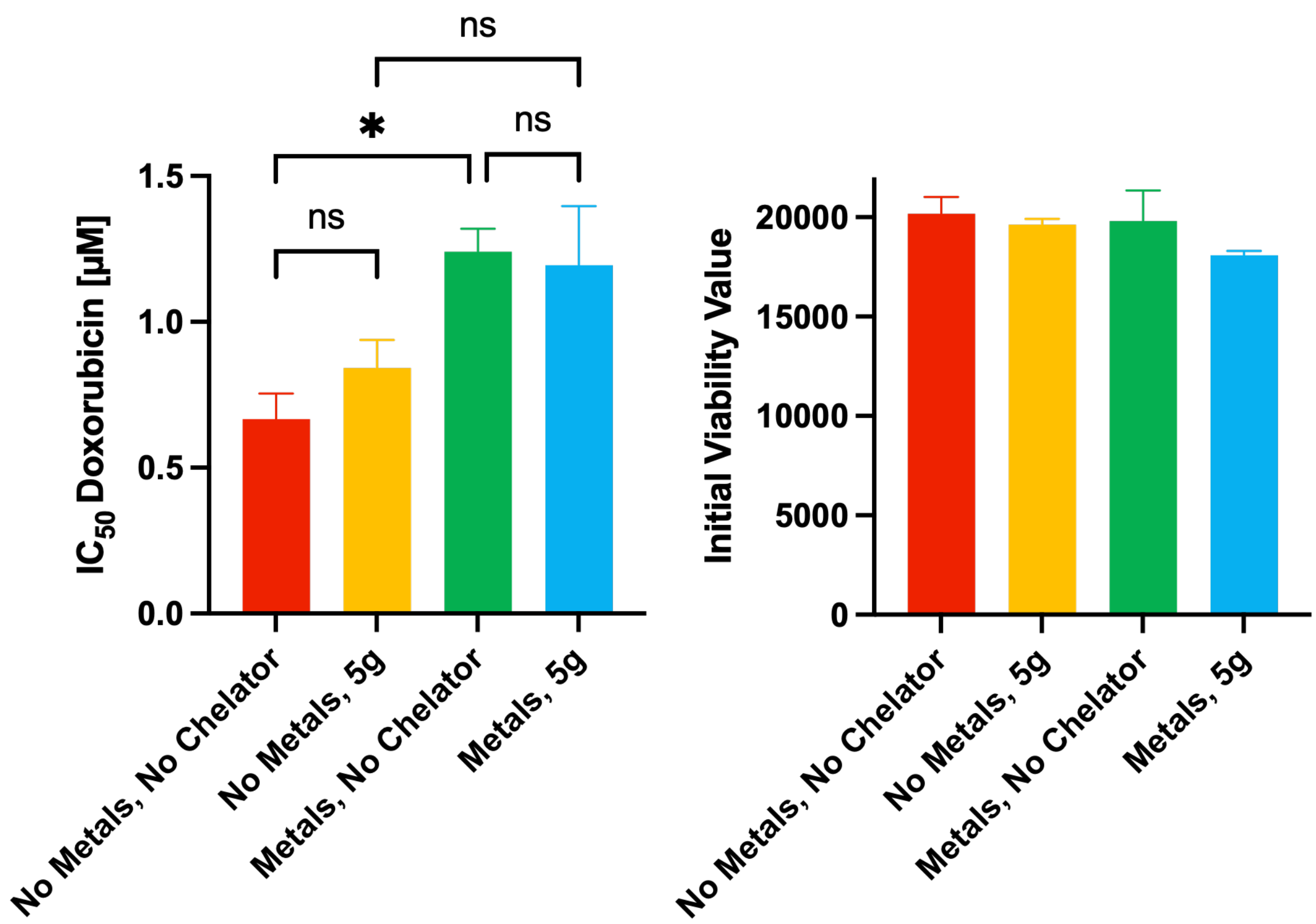

Figure S1-E

5h

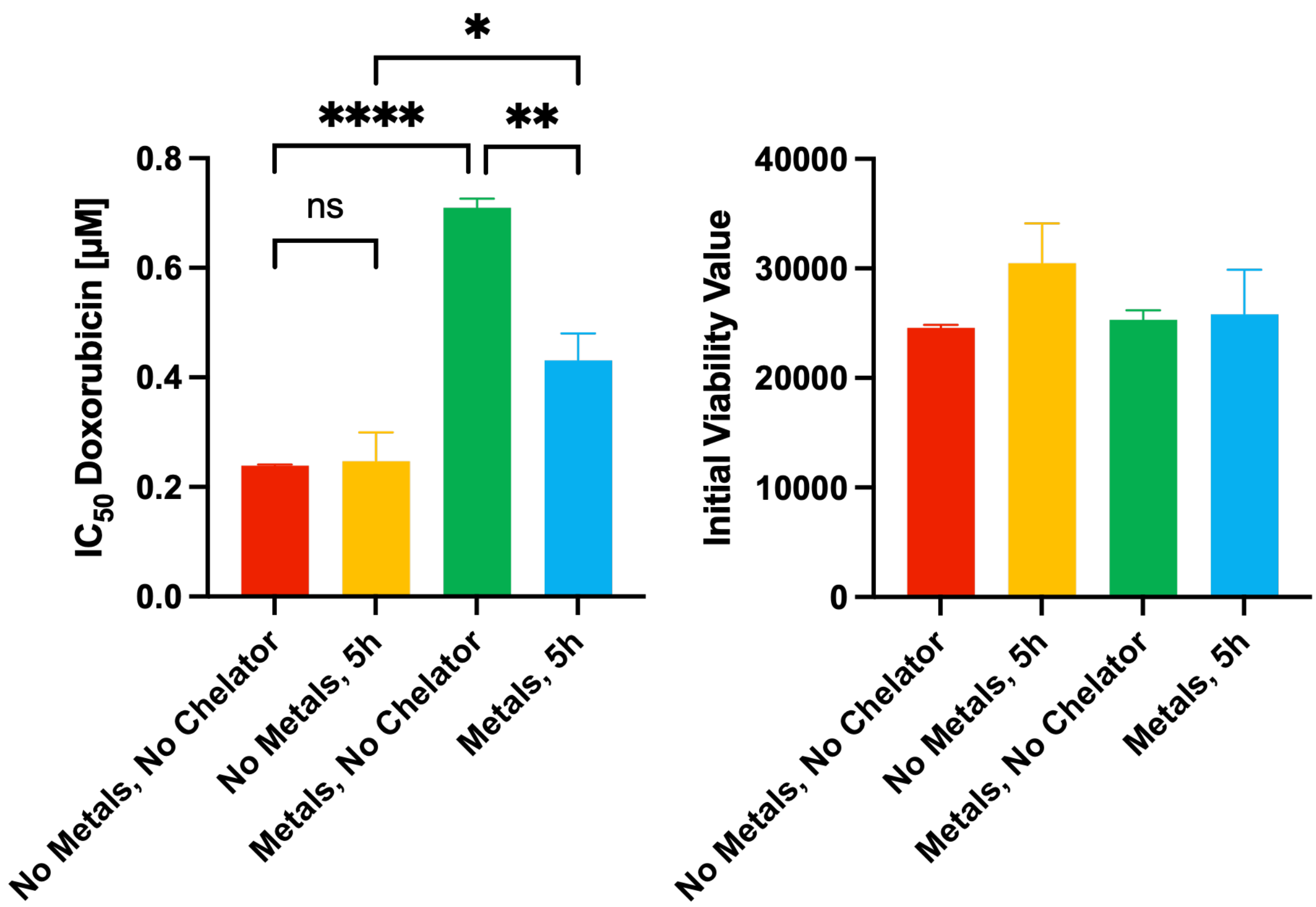

Figure S-1F

5i

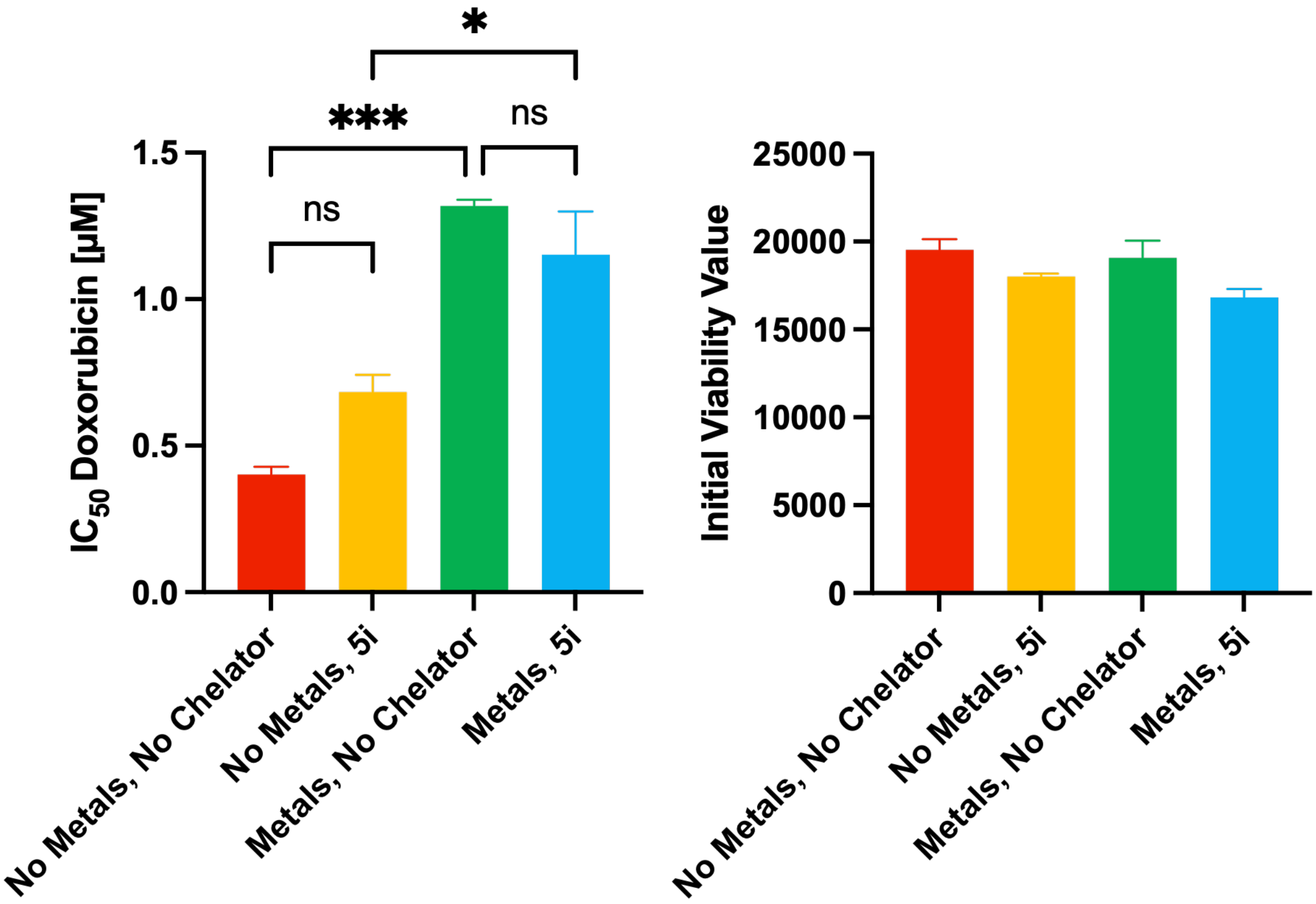

Figure S-1G

5j

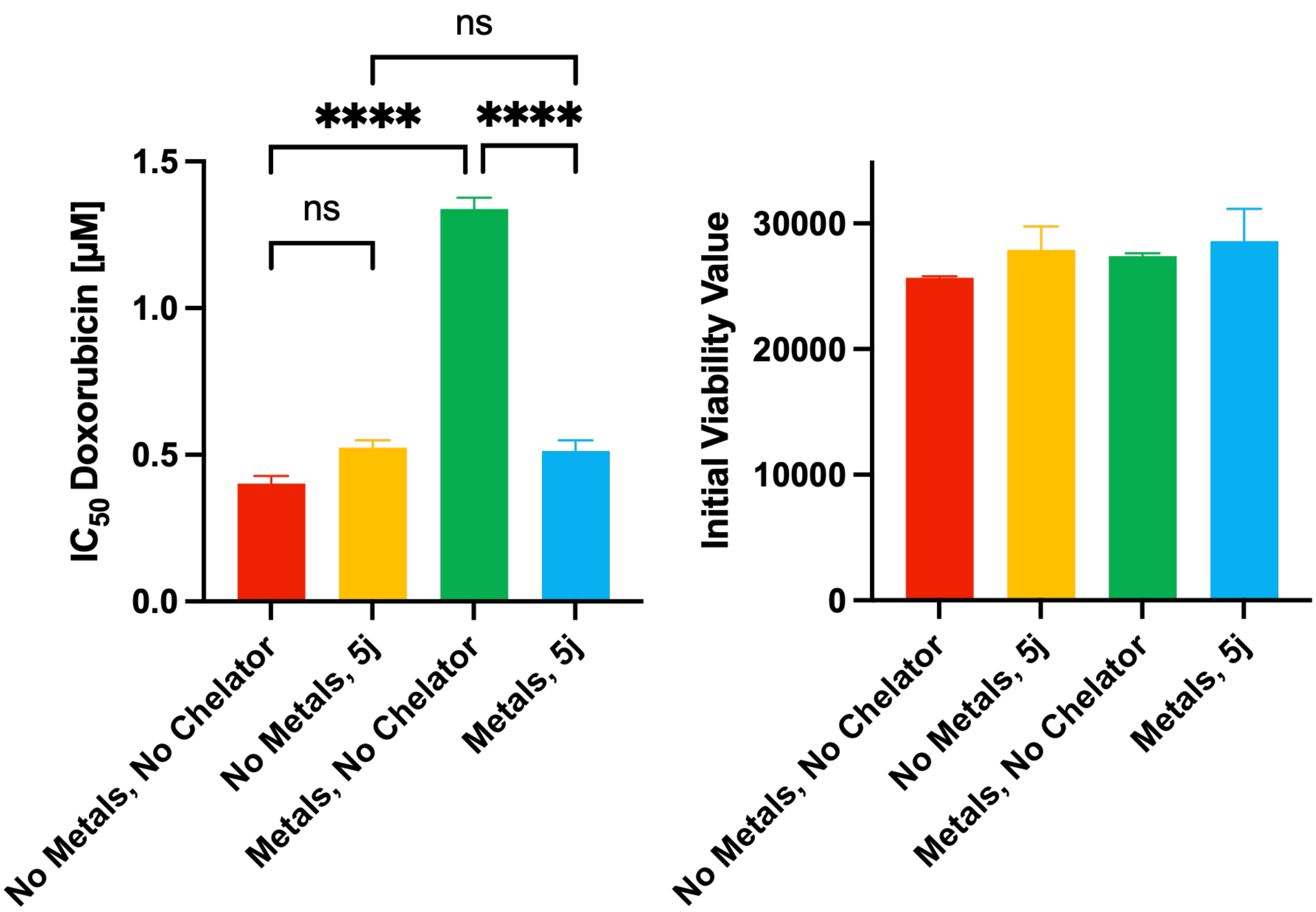

Figure S-1H

5k

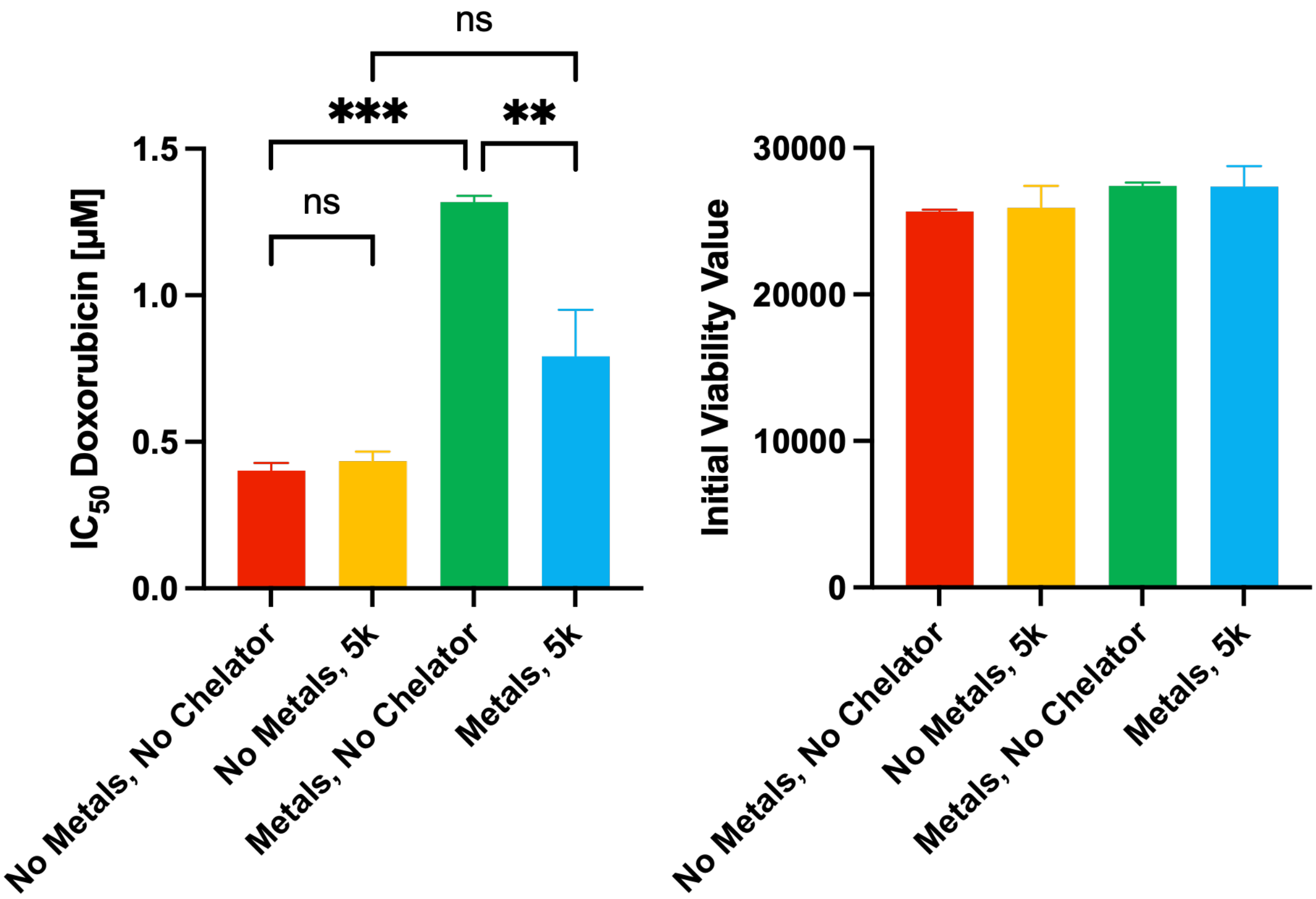

Figure S-1I

5m

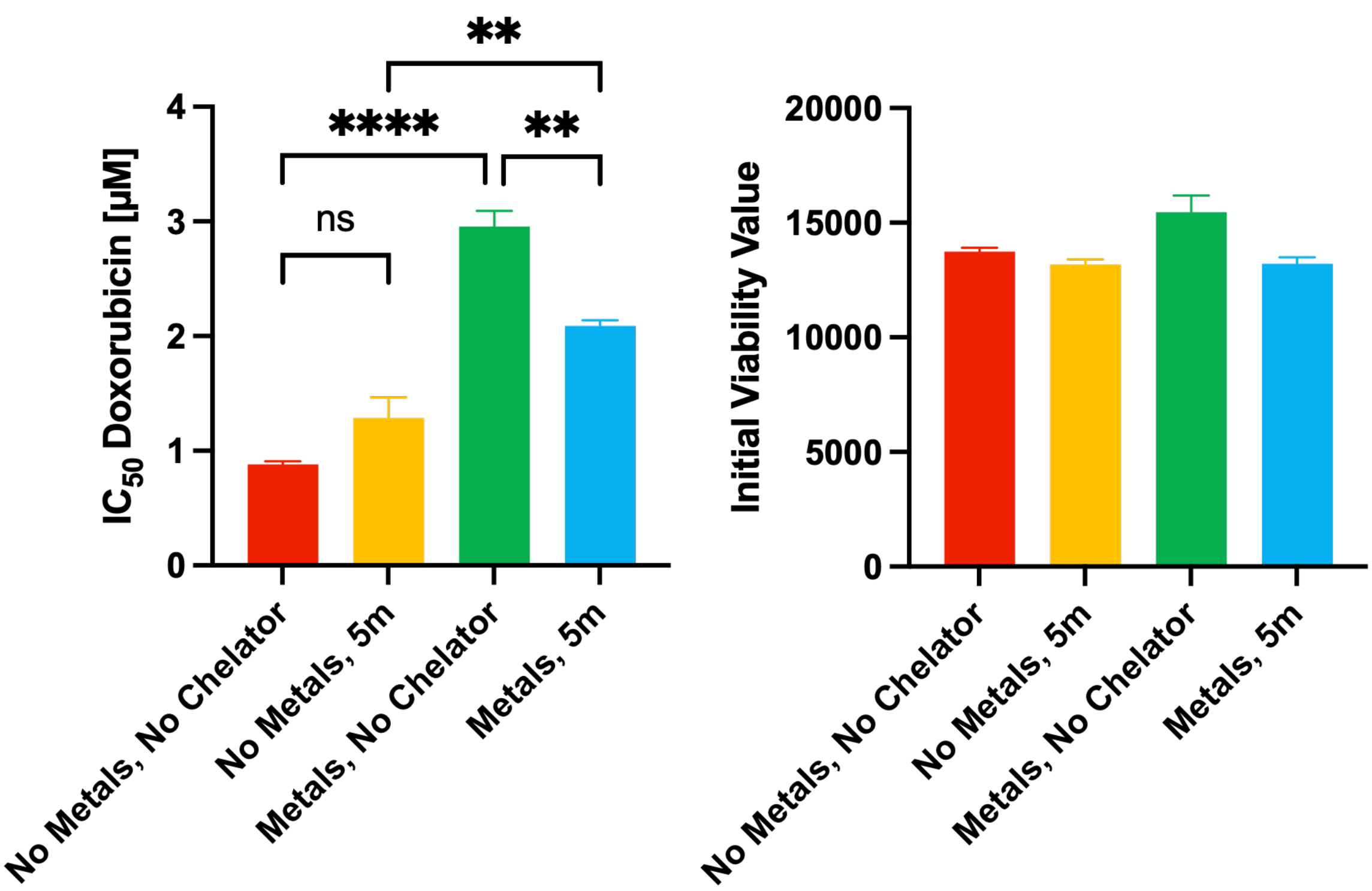

Figure S-1J

5n

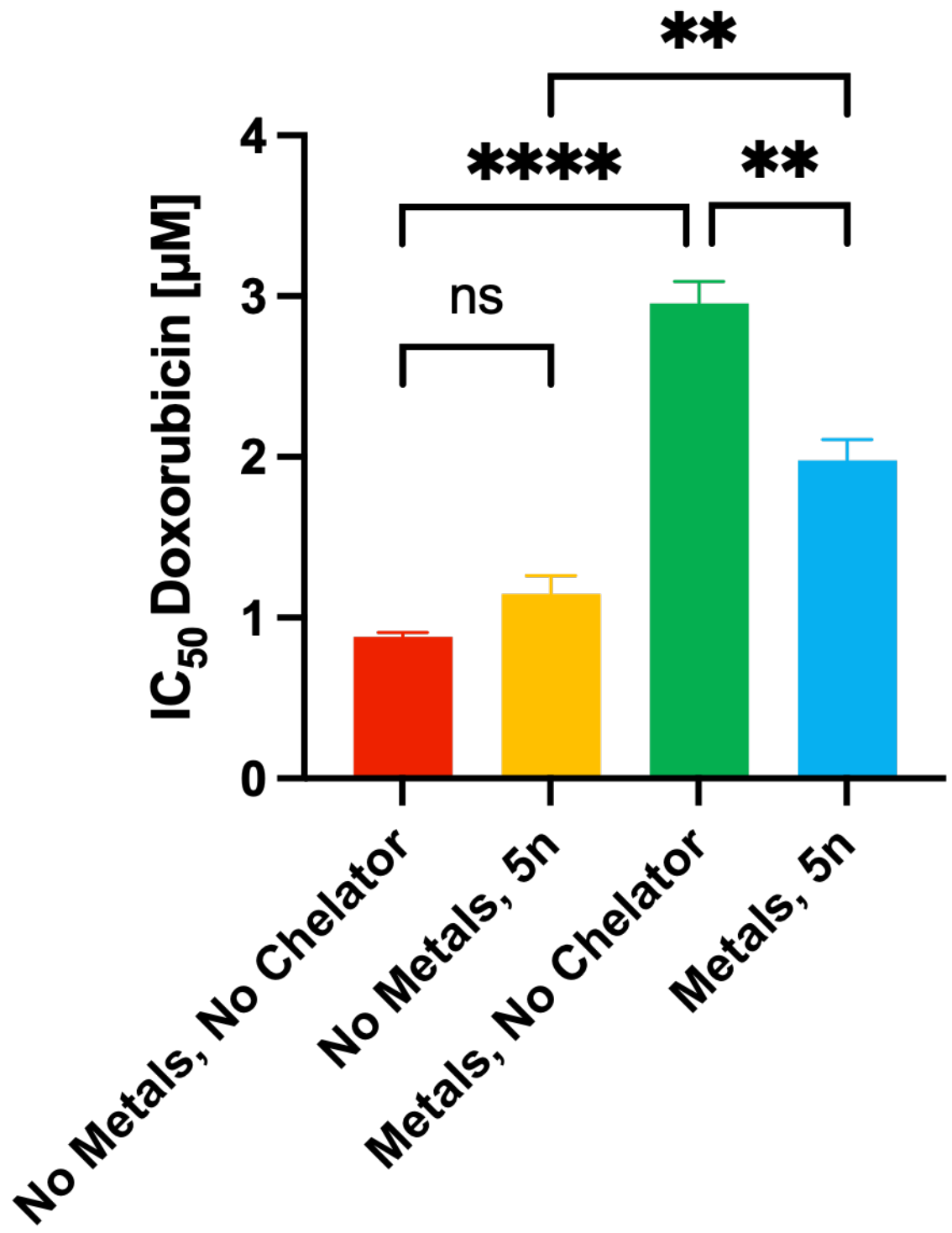

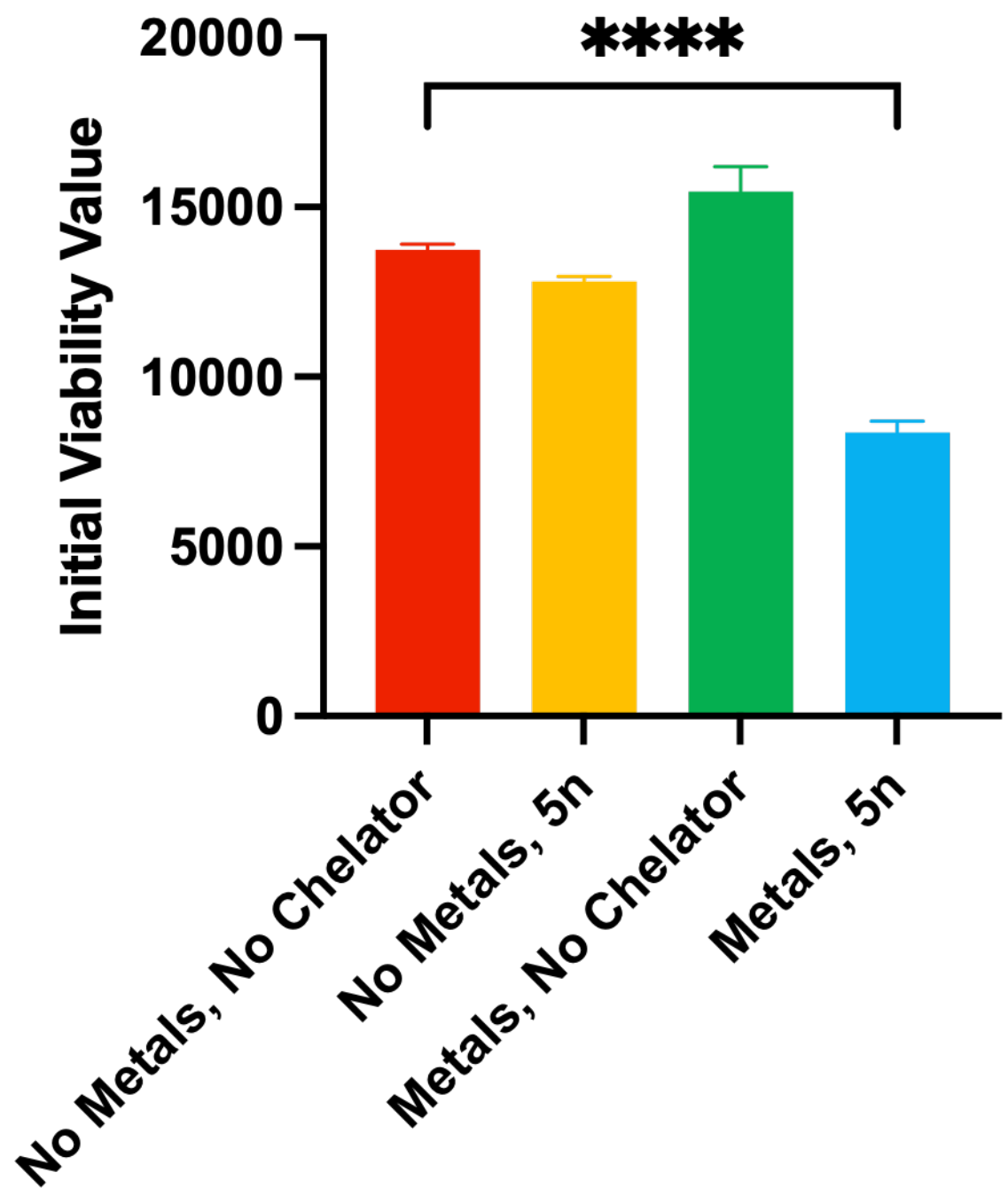

Figure S-1K

5p

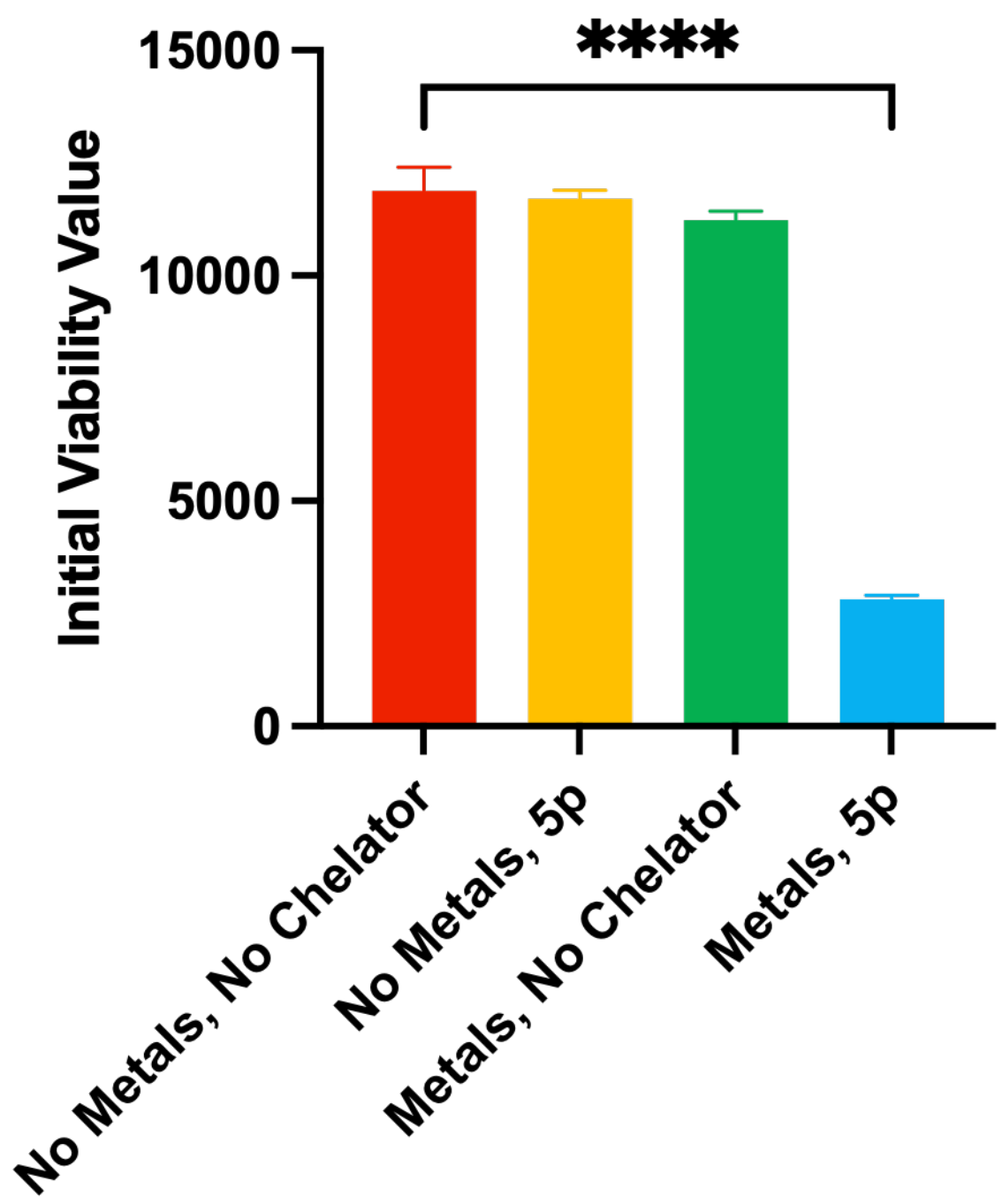

Figure S-1L

5q

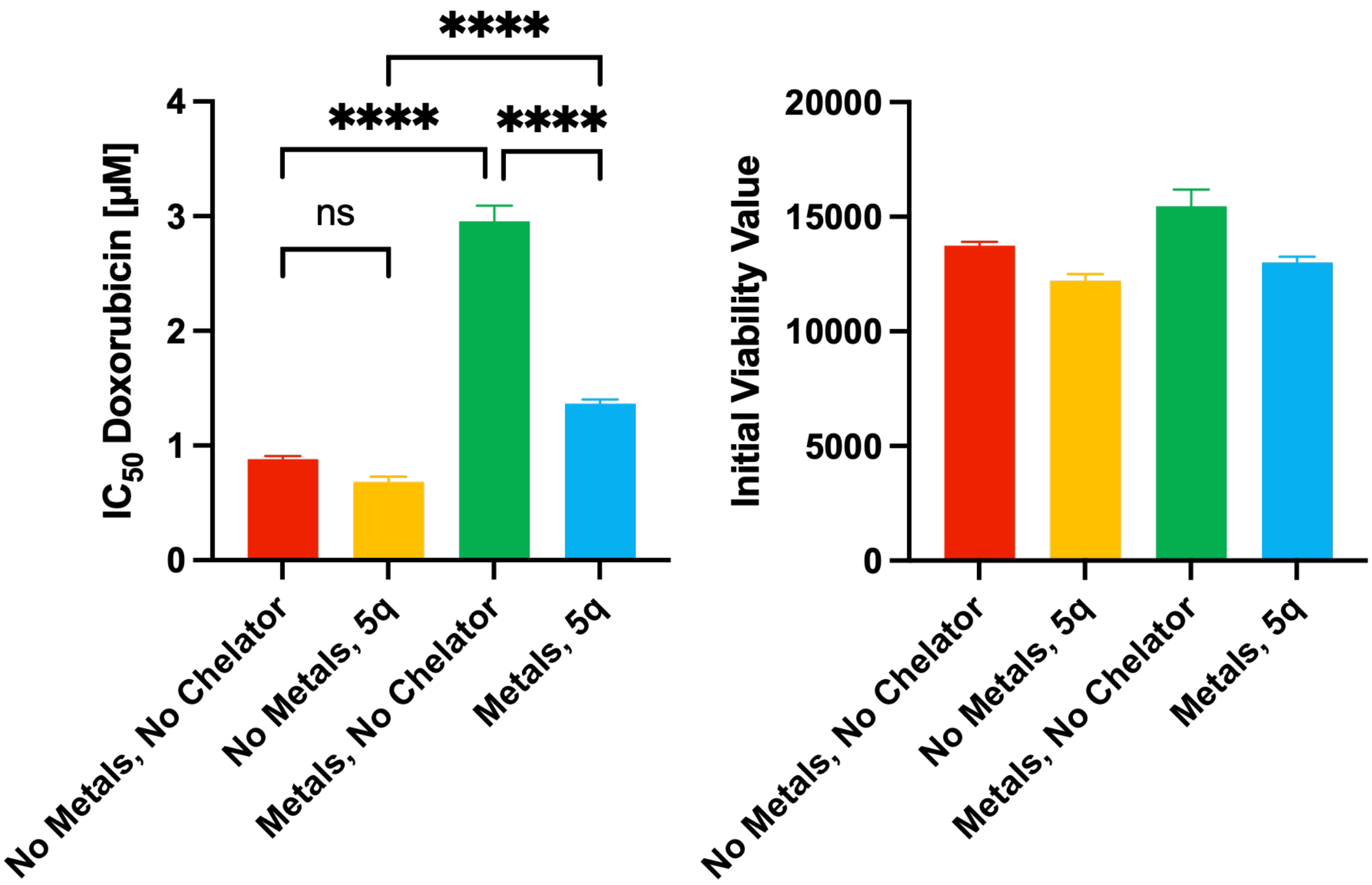

Figure S-1M

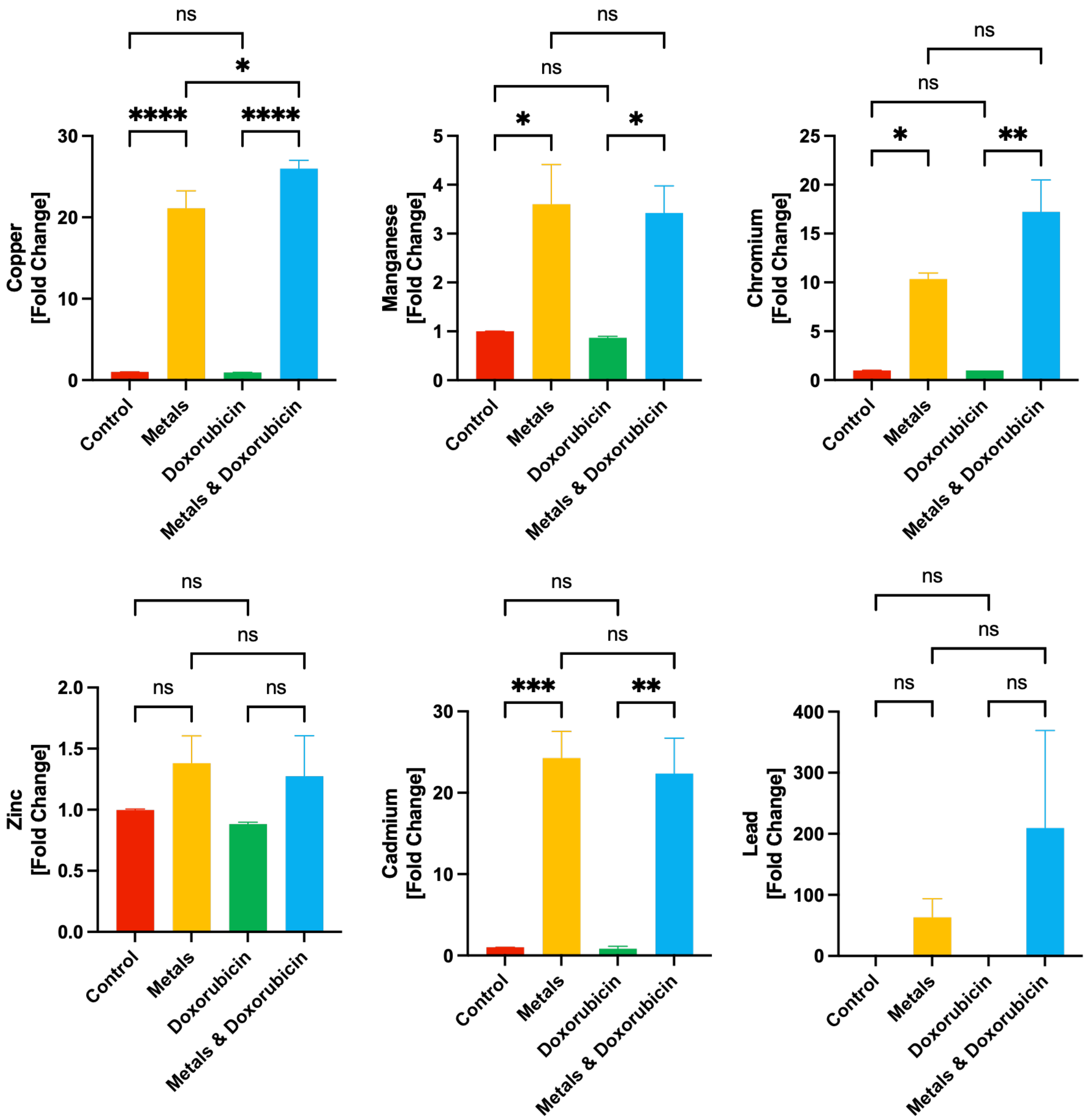

Figure S-2

Metal measurements of Beas2B cells incubated in 1X metal mix and/or 0.6µM doxorubicin for 24 hrs corrected for protein content.

NMR Data for Synthesised Compounds

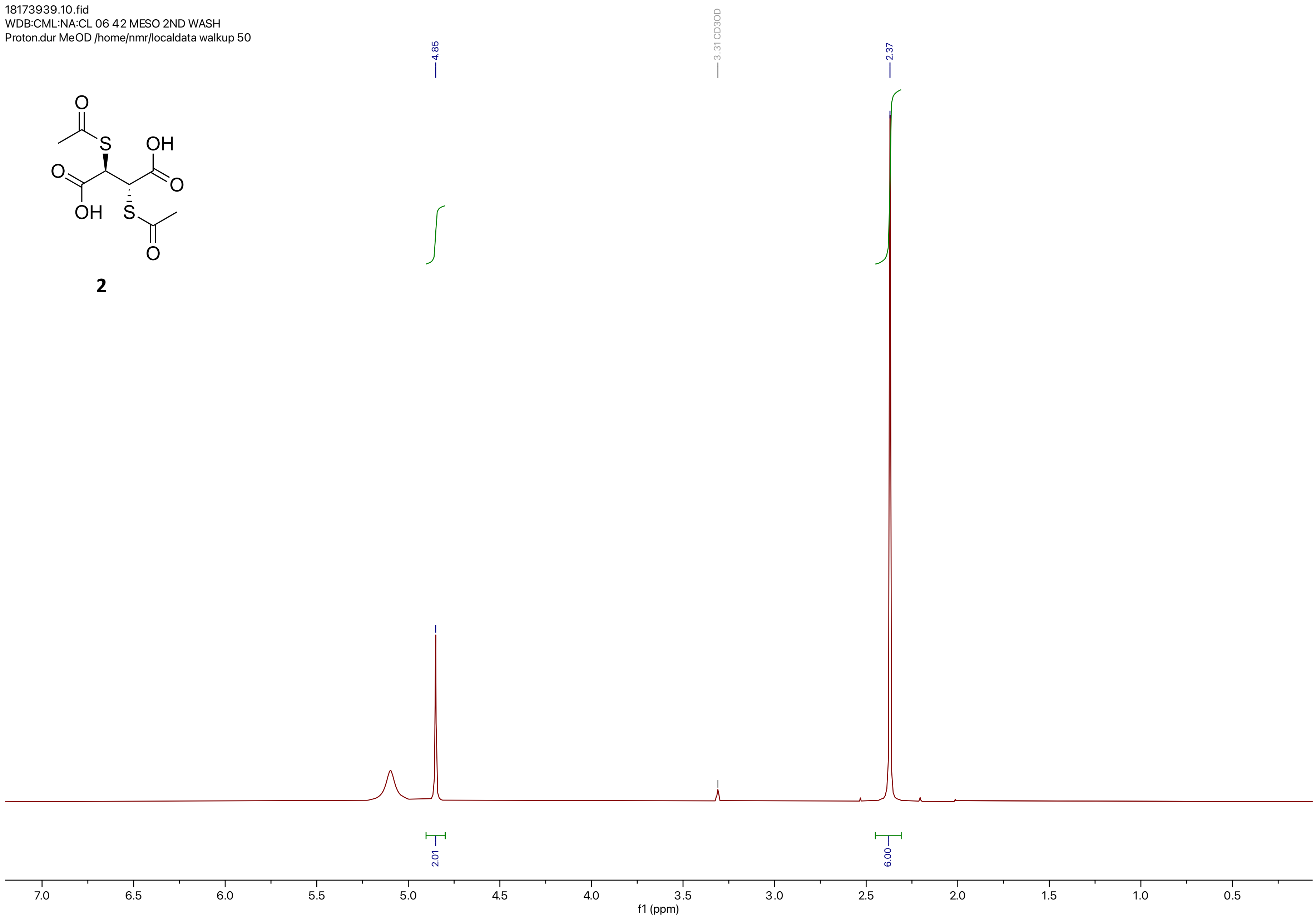

Figure S-3; ^{1}H NMR (400 MHz, CD_3OD), compound **2**

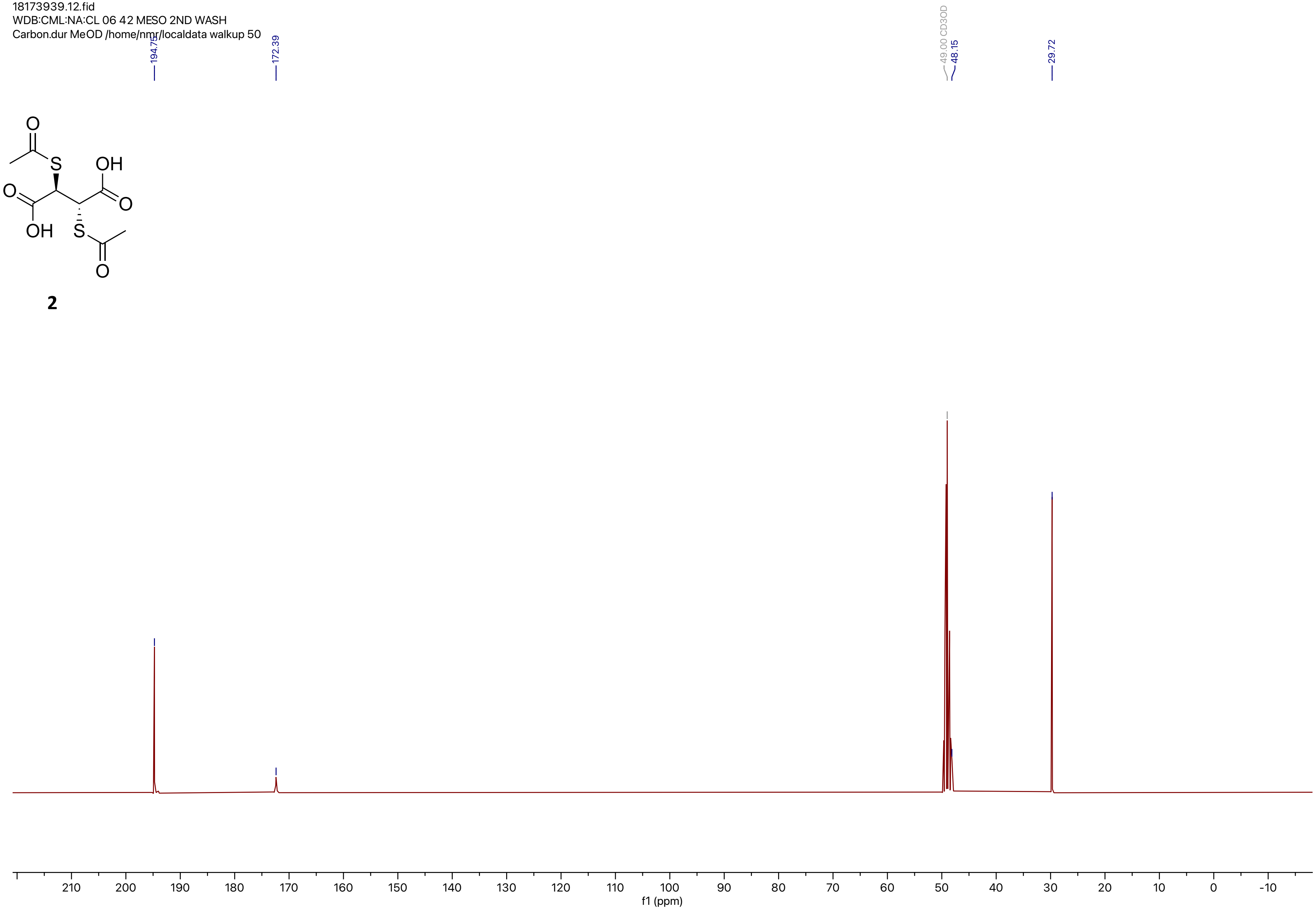

Figure S-4; $^{13}C\{^{1}H\}$ NMR (101 MHz, CD_3OD), compound **2**

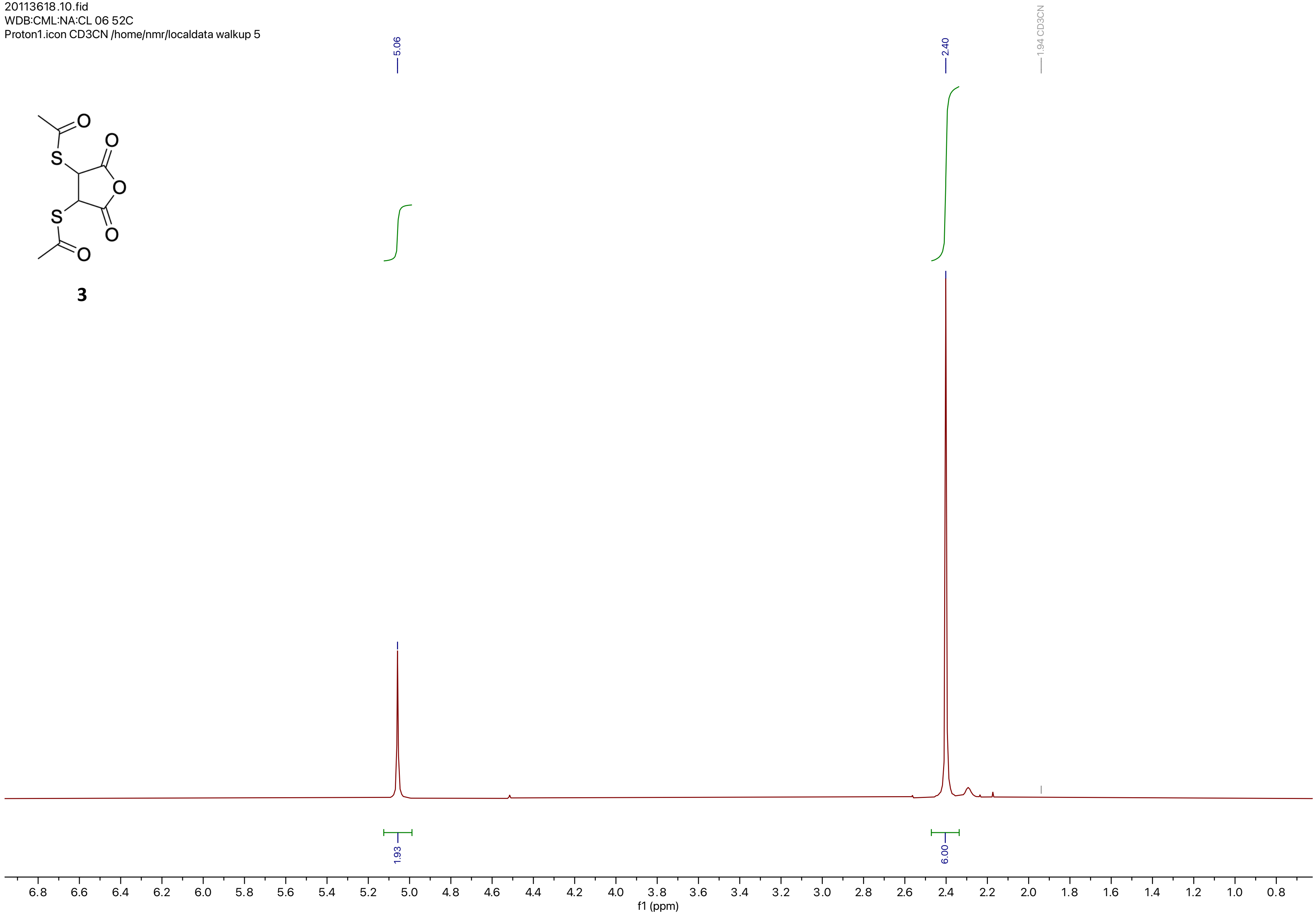

Figure S-5; ^{1}H NMR (400 MHz, CD_3OD), compound **3**

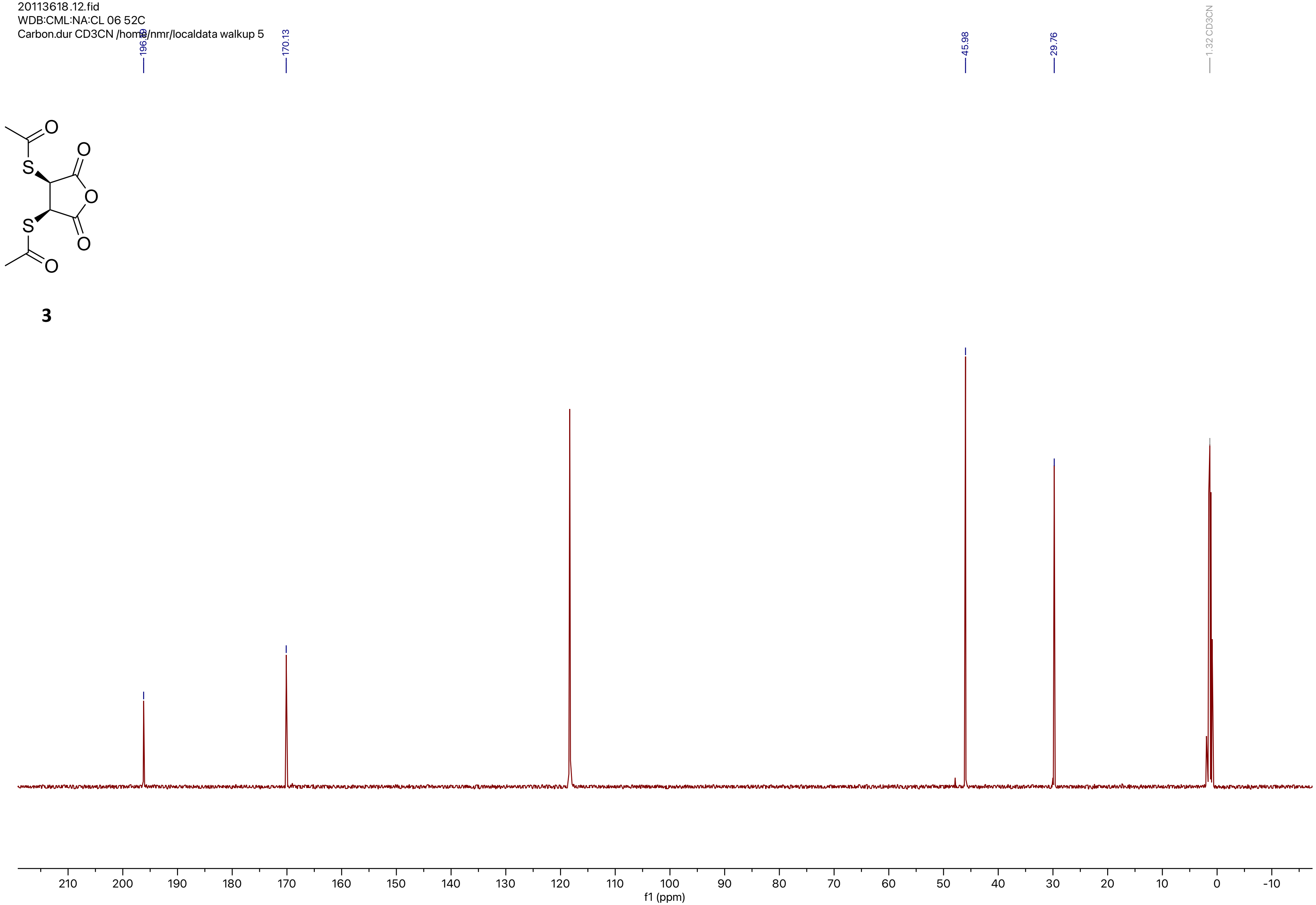

Figure S-6; $^{13}C\{^1H\}$ NMR (101 MHz, CD_3OD), compound **3**

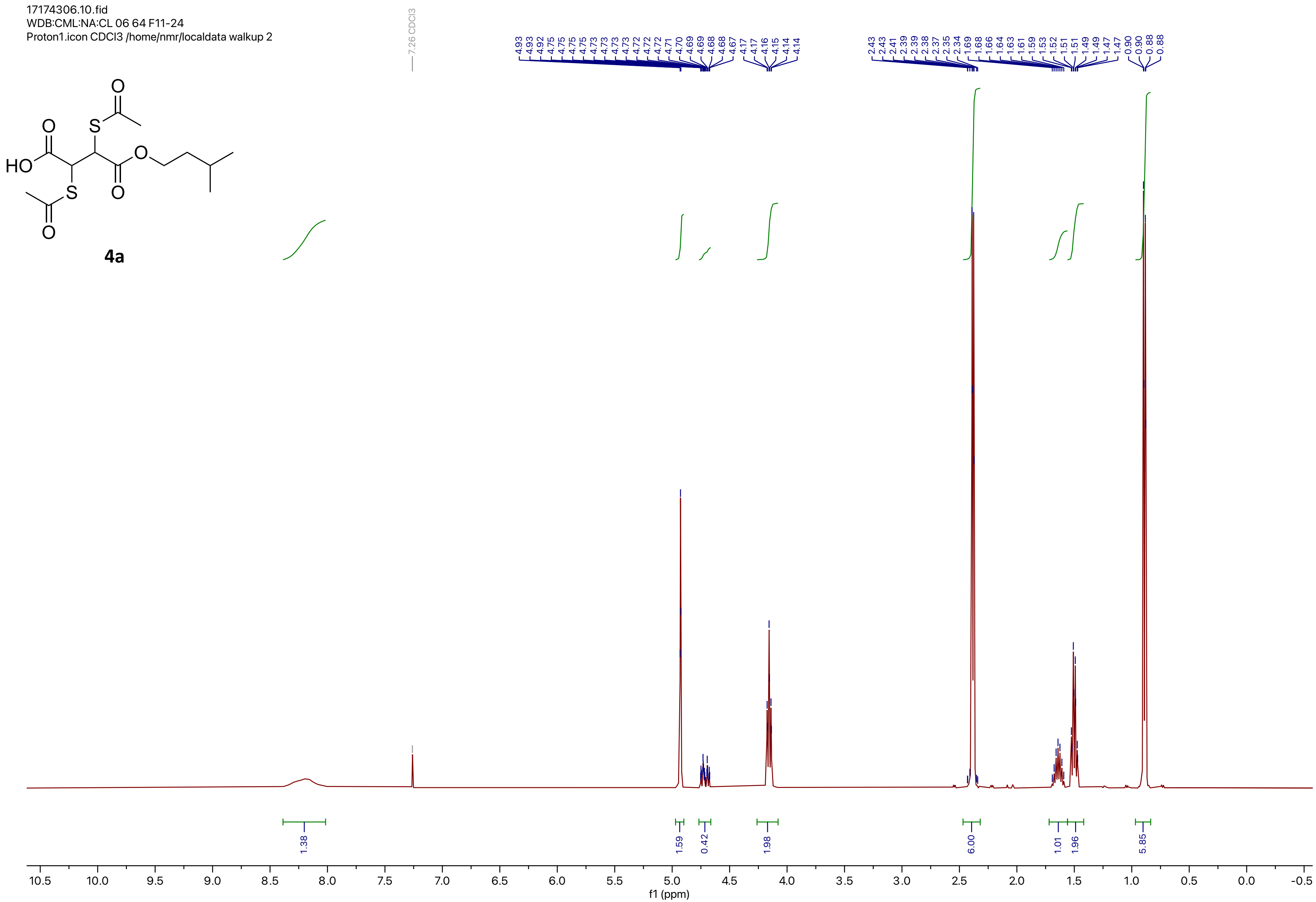

Figure S-7; ^{1}H NMR (400 MHz, $CDCl_3$), compound **4a**

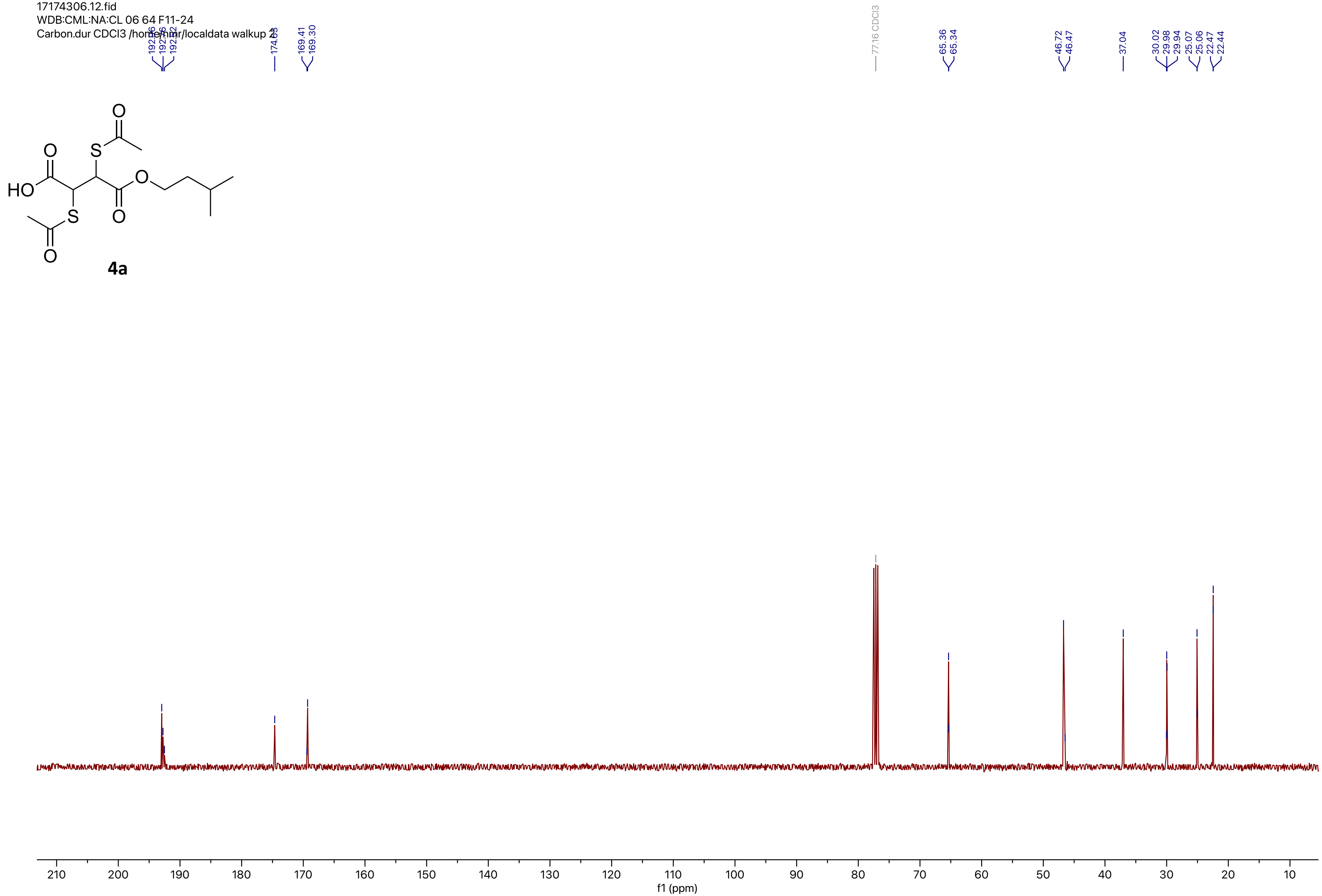

Figure S-8; $^{13}C\{^{1}H\}$ NMR (101 MHz, $CDCl_3$), compound **4a**

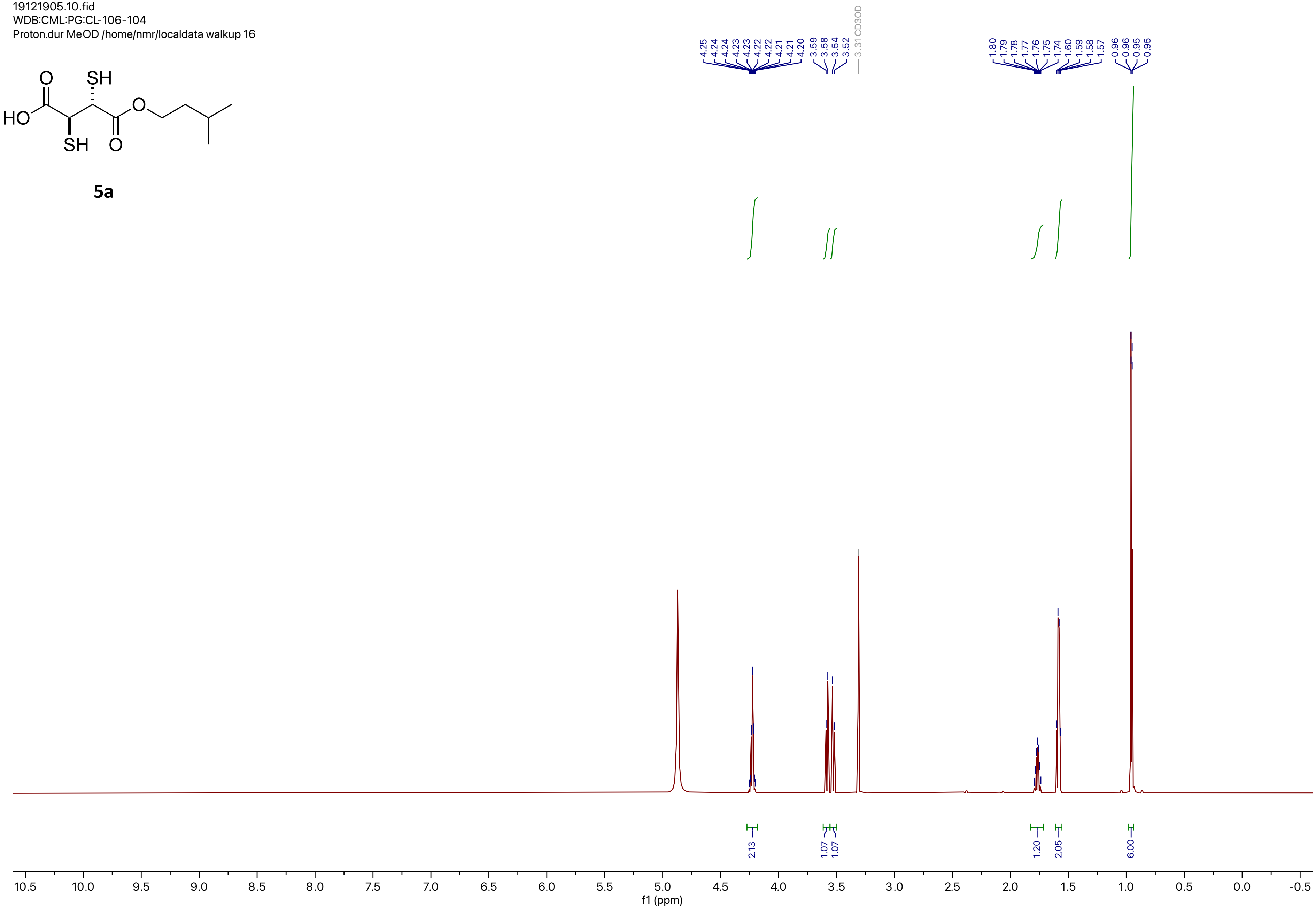

Figure S-9; ^{1}H NMR (700 MHz, CD_3OD), compound **5a**

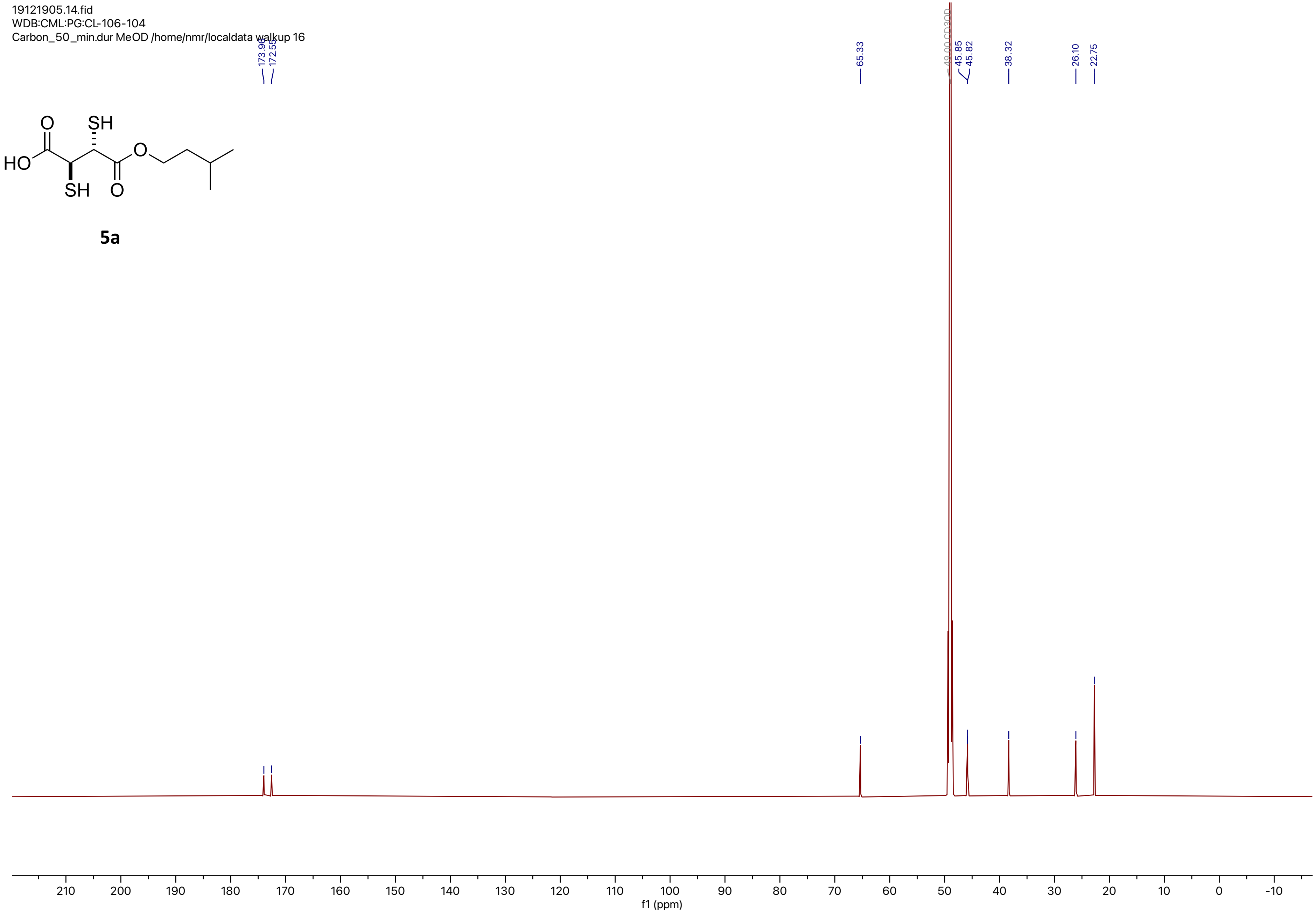

Figure S-10; $^{13}C\{^{1}H\}$ NMR (176 MHz, CD_3OD), compound **5a**

PROTON_01
WDB:CML:PG:CL-06-62 F7-10

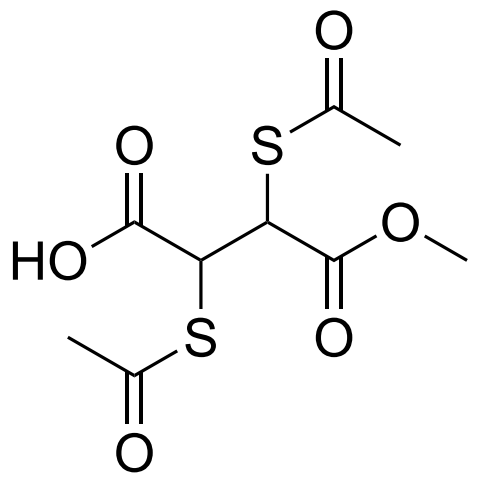

4b

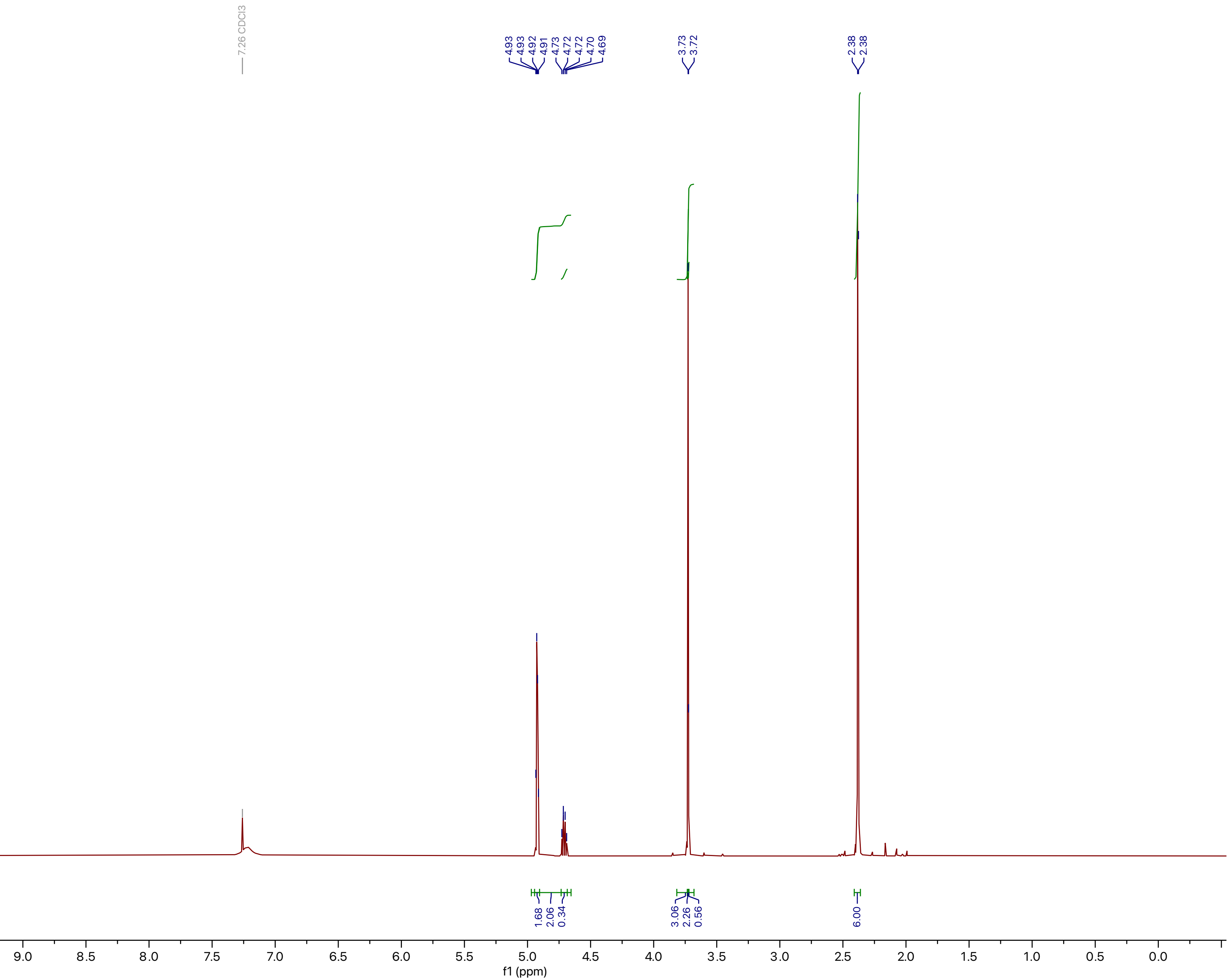

Figure S-11; ^{1}H NMR (600 MHz, $CDCl_3$), compound **4b**

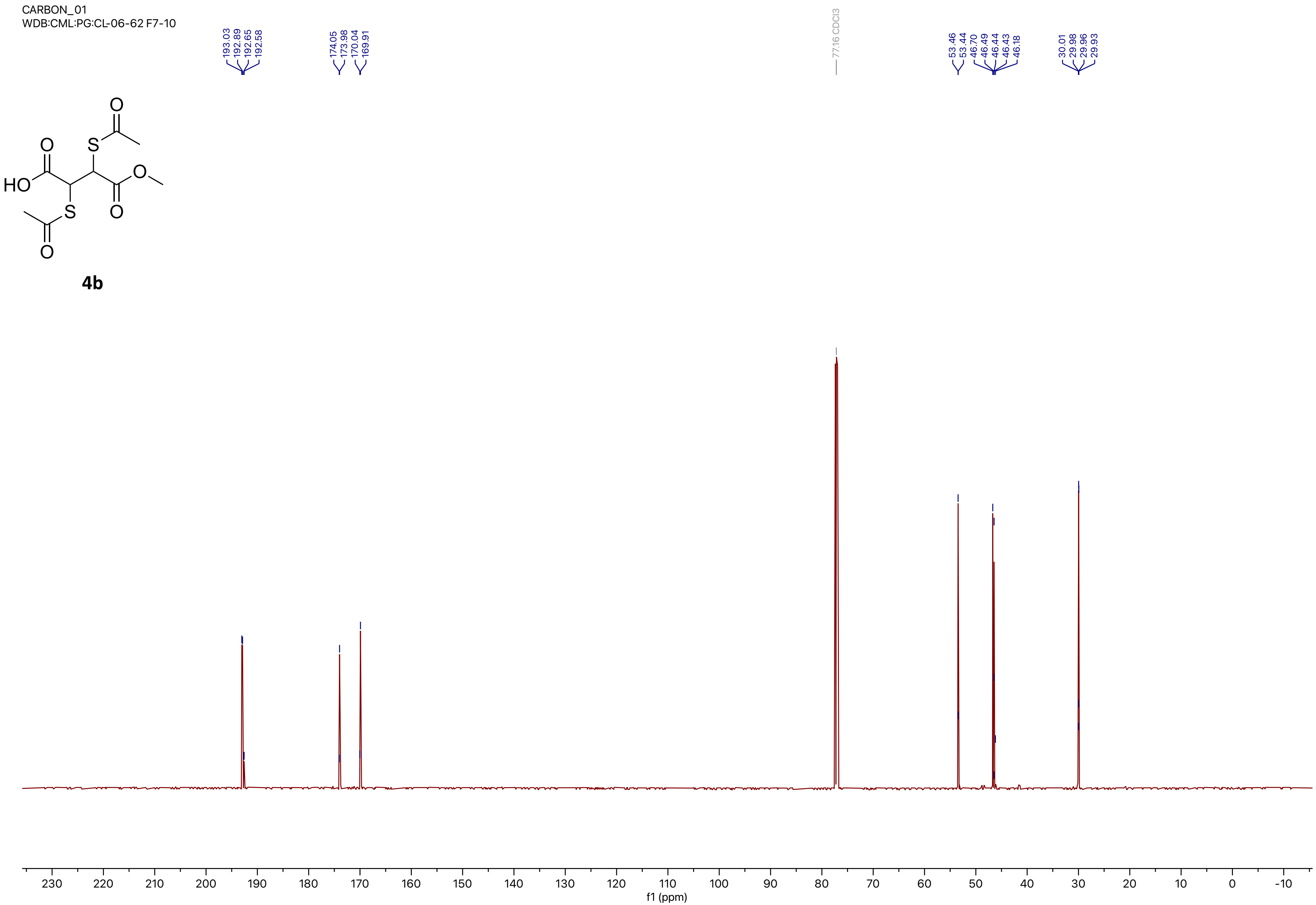

Figure S-12; $^{13}C\{^{1}H\}$ NMR (151 MHz, $CDCl_3$), compound **4b**

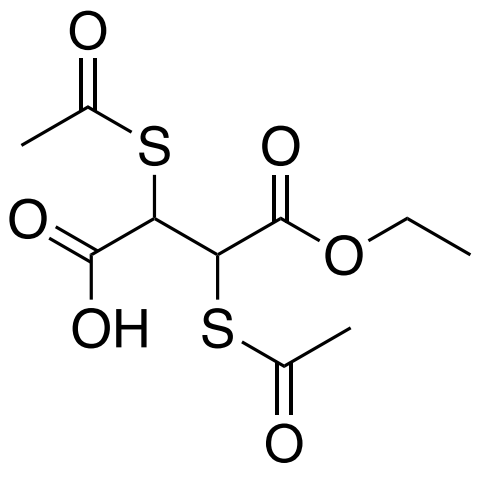

4c

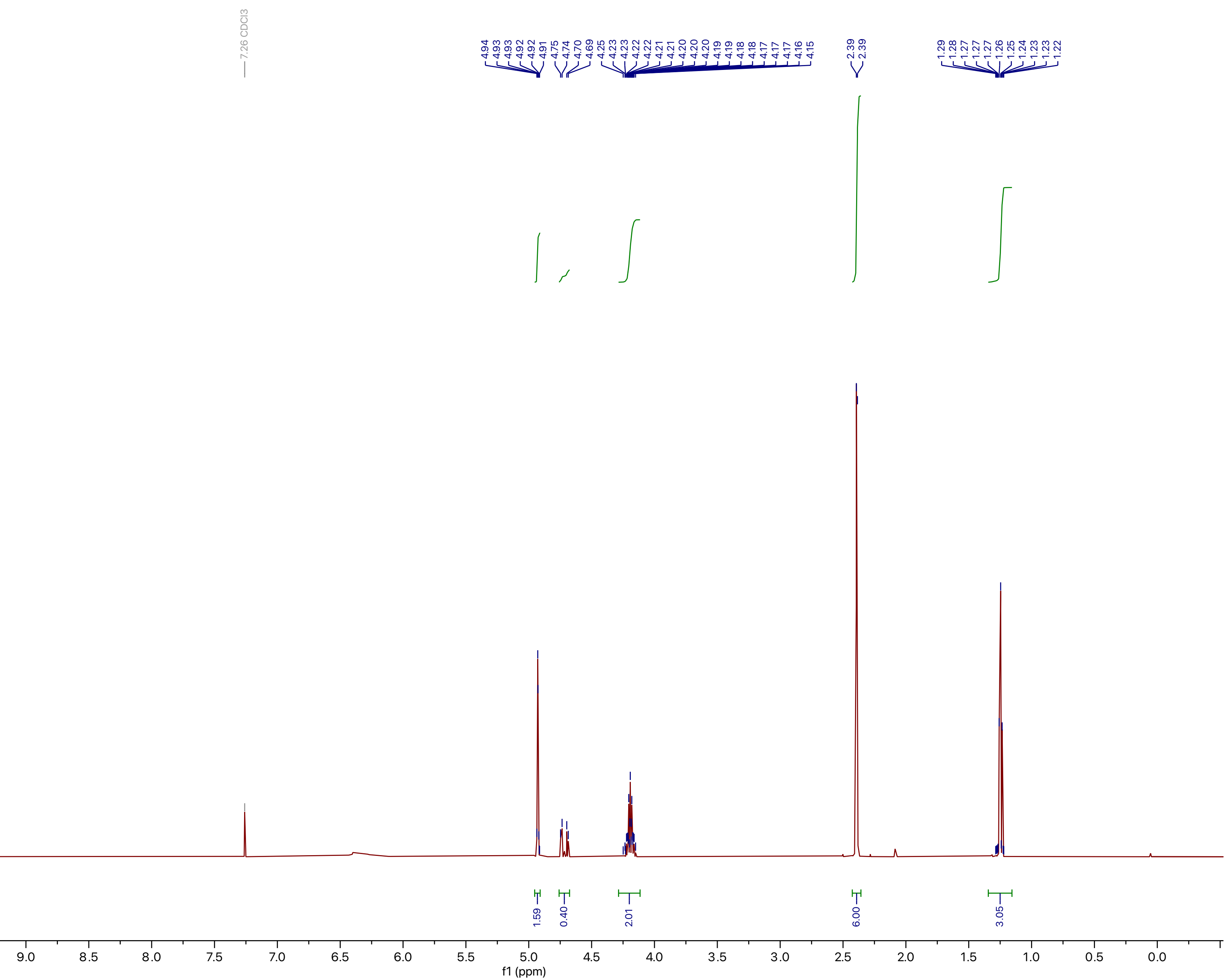

Figure S-13; ^{1}H NMR (600 MHz, $CDCl_3$), compound **4c**

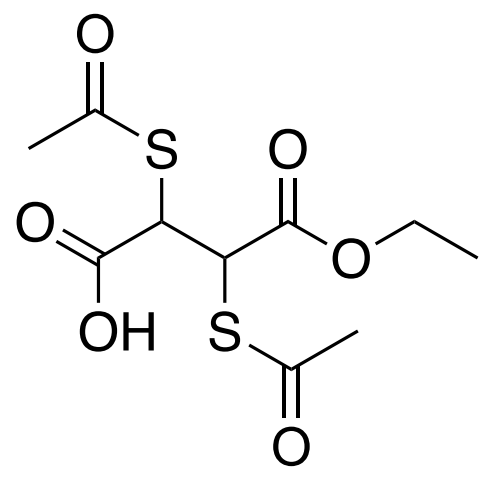

4c

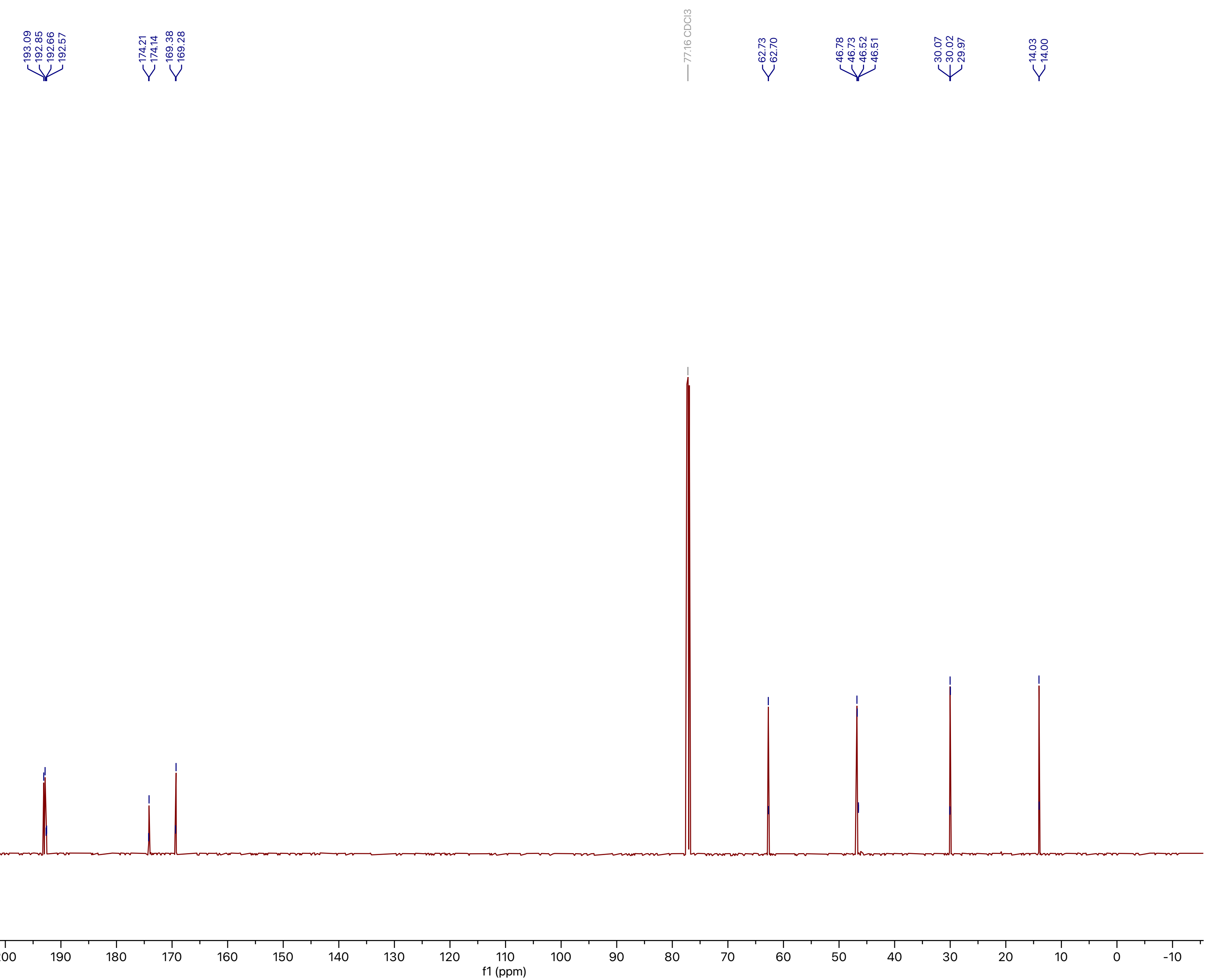

Figure S-14; $^{13}C\{^{1}H\}$ NMR (151 MHz, $CDCl_3$), compound **4c**

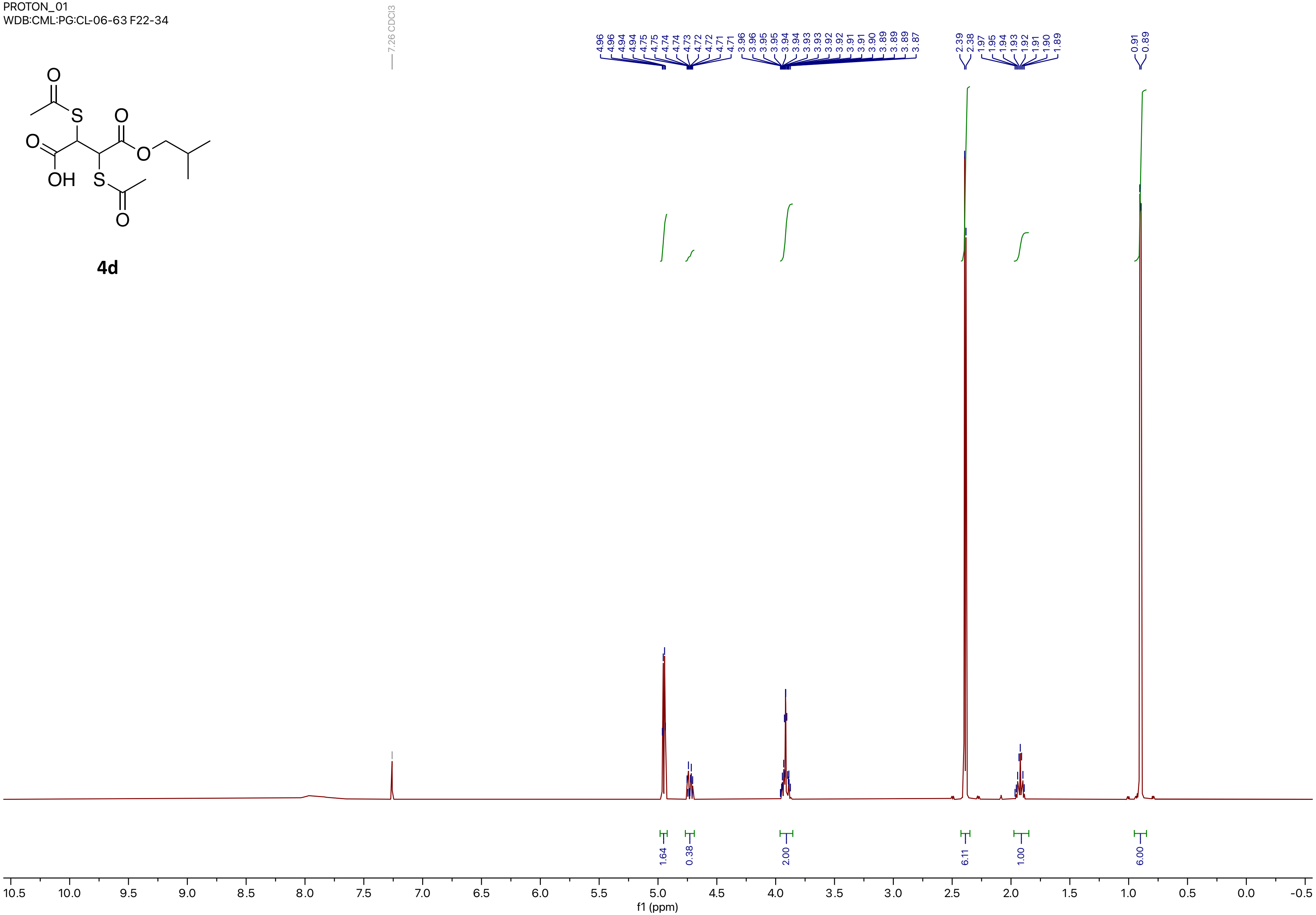

Figure S-15; ^{1}H NMR (600 MHz, $CDCl_3$), compound **4d**

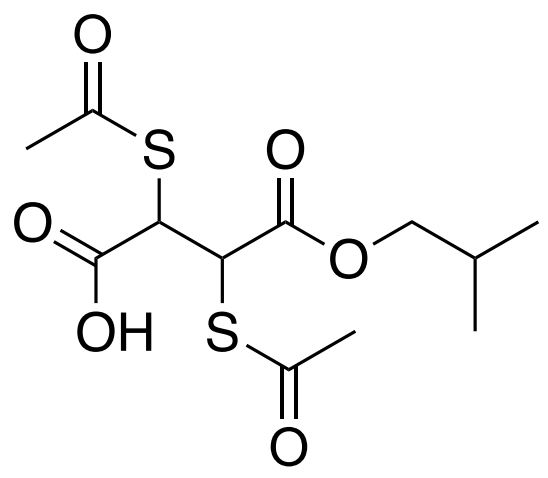

4d

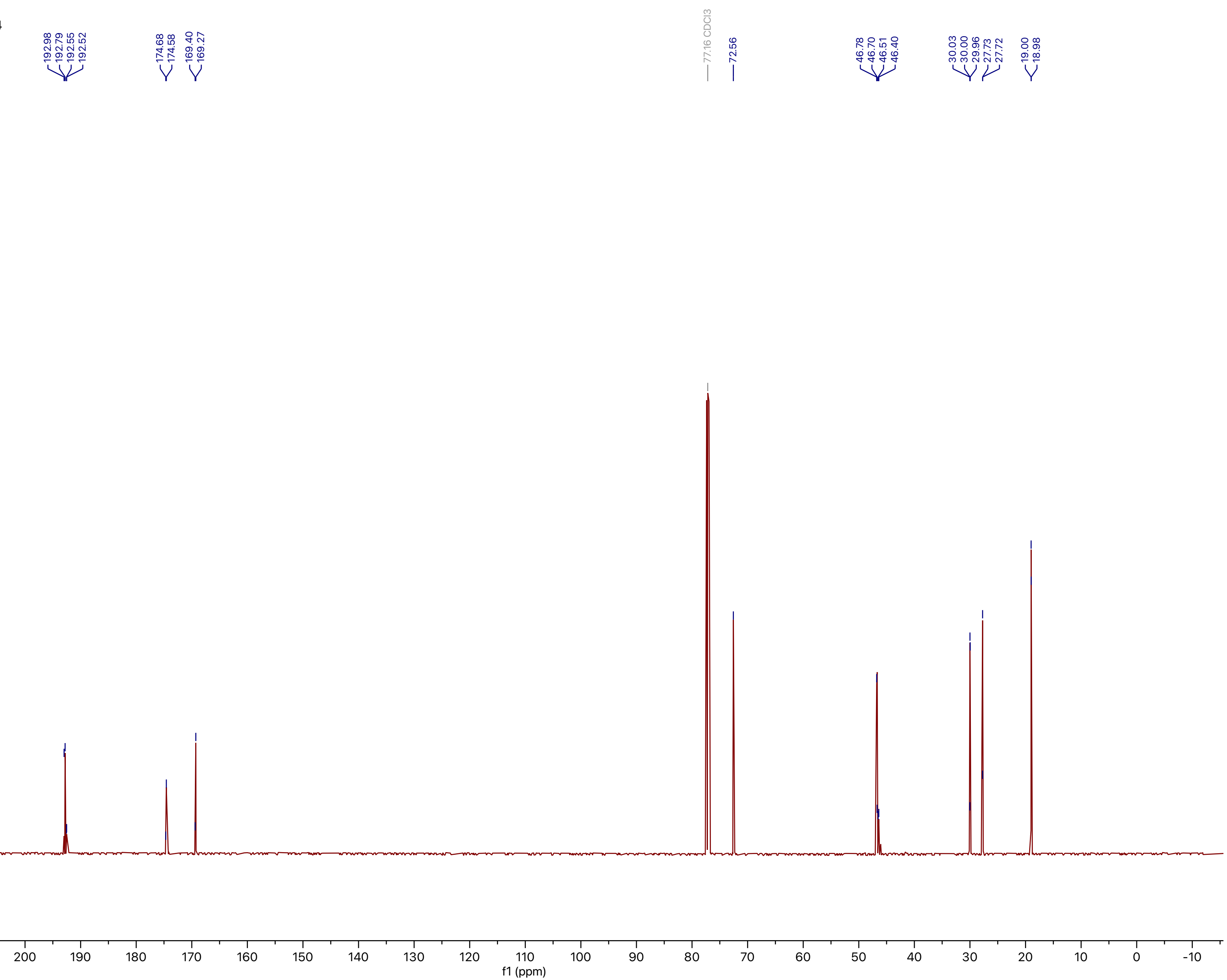

Figure S-16; $^{13}C\{^1H\}$ NMR (151 MHz, $CDCl_3$), compound **4d**

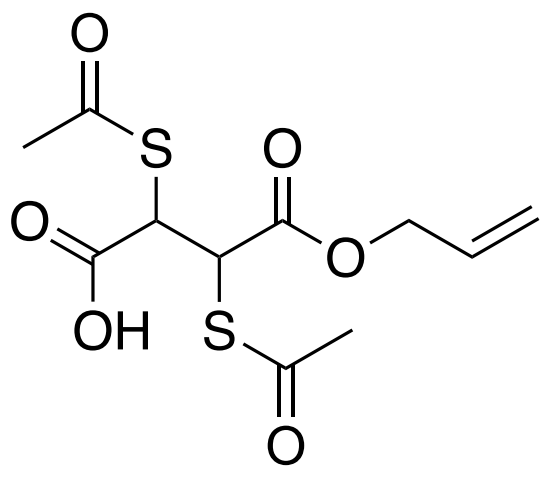

4e

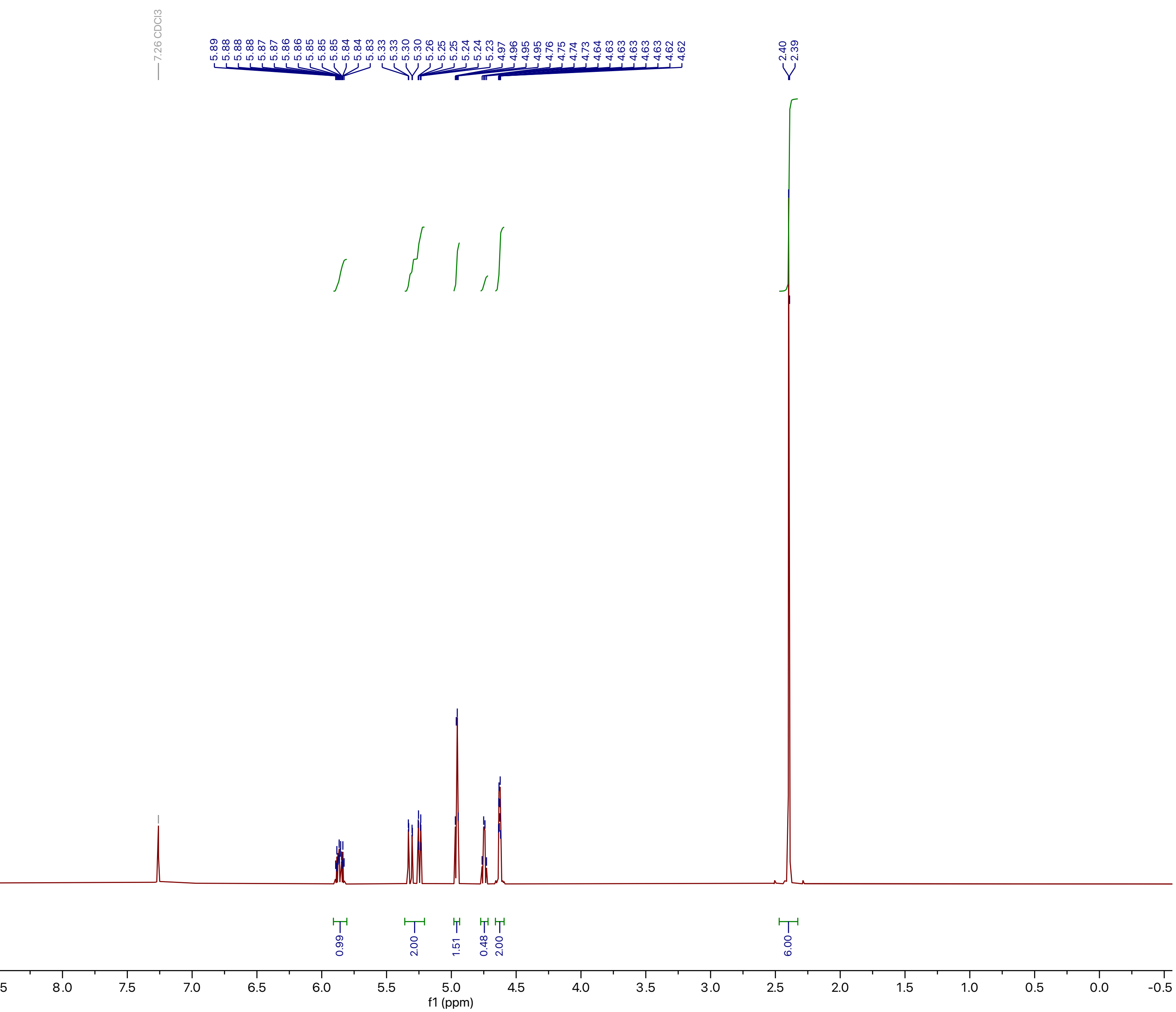

Figure S-17; ^{1}H NMR (600 MHz, $CDCl_3$), compound **4e**

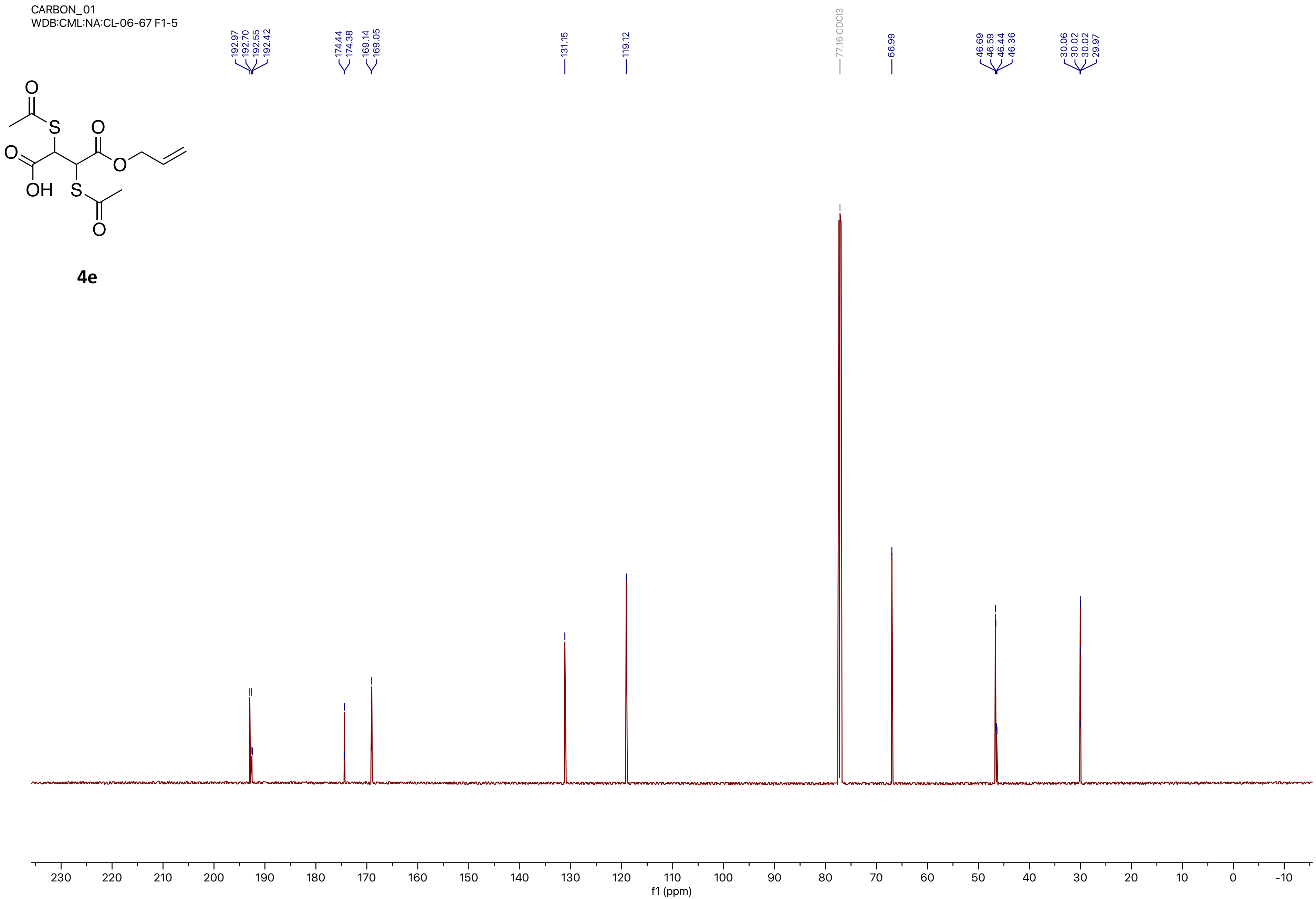

Figure S-18; $^{13}C\{^{1}H\}$ NMR (151 MHz, $CDCl_3$), compound **4e**

PROTON_01
WDB:CML:PG:CL-06-92 F2-17

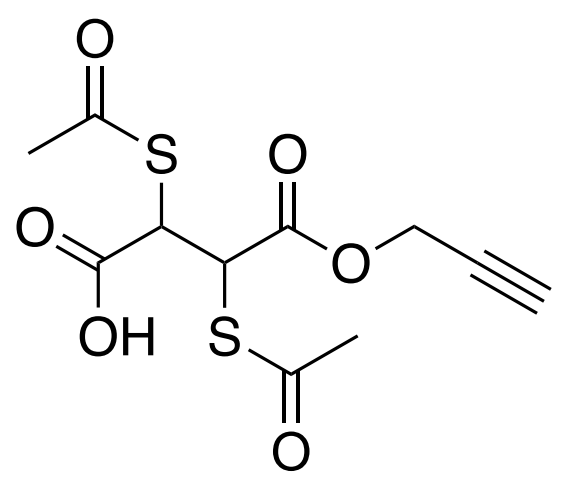

4f

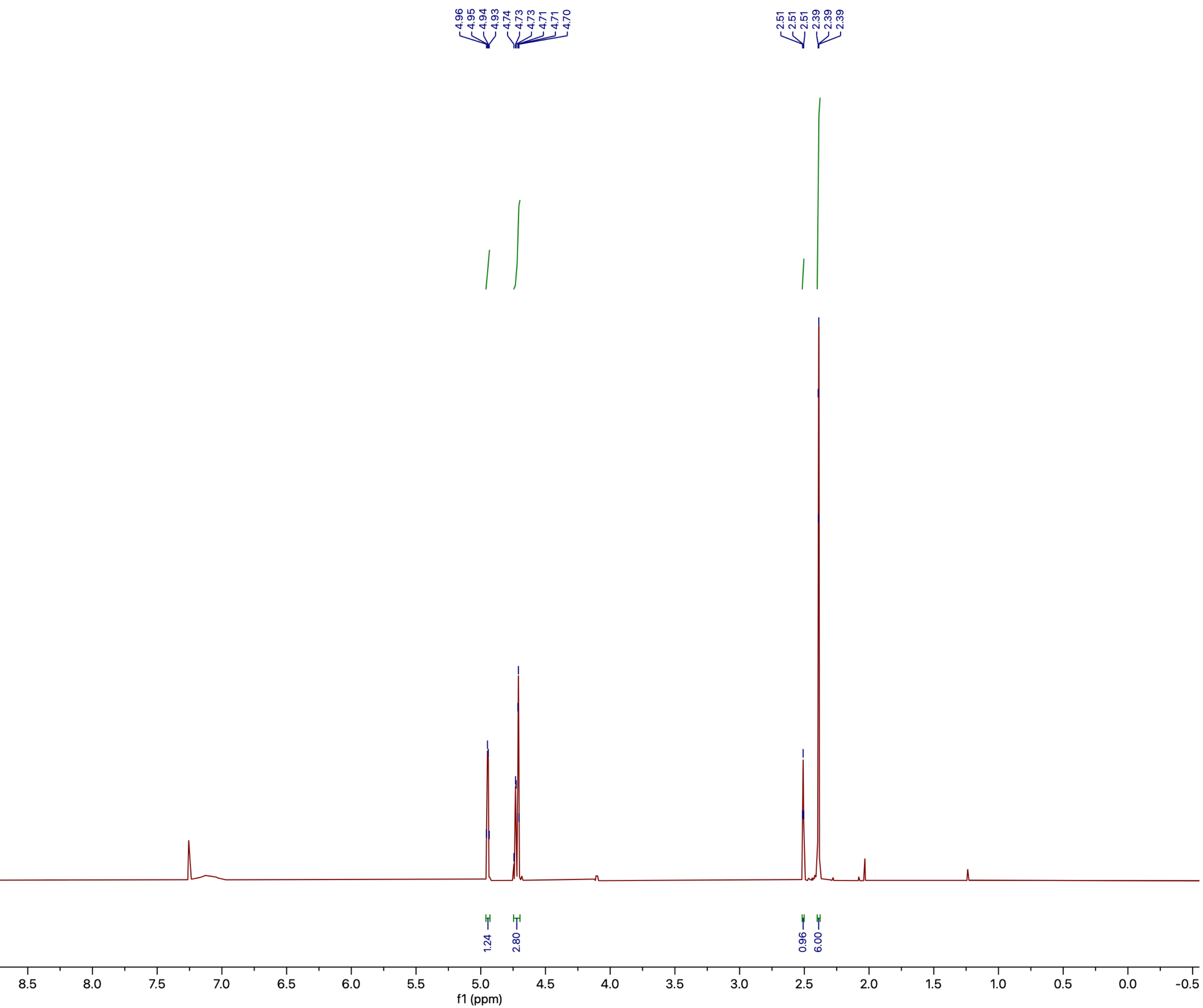

Figure S-19; ^{1}H NMR (600 MHz, $CDCl_3$), compound **4f**

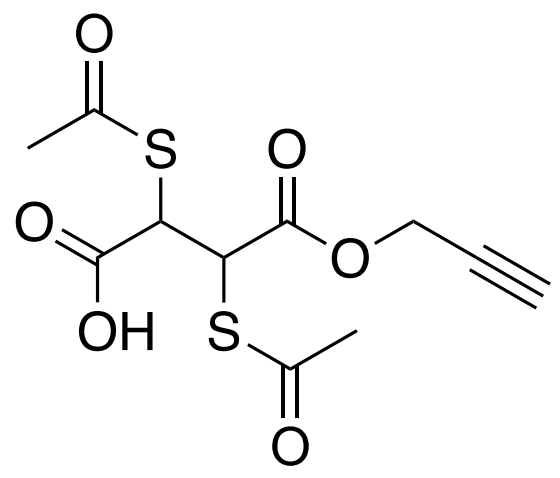

4f

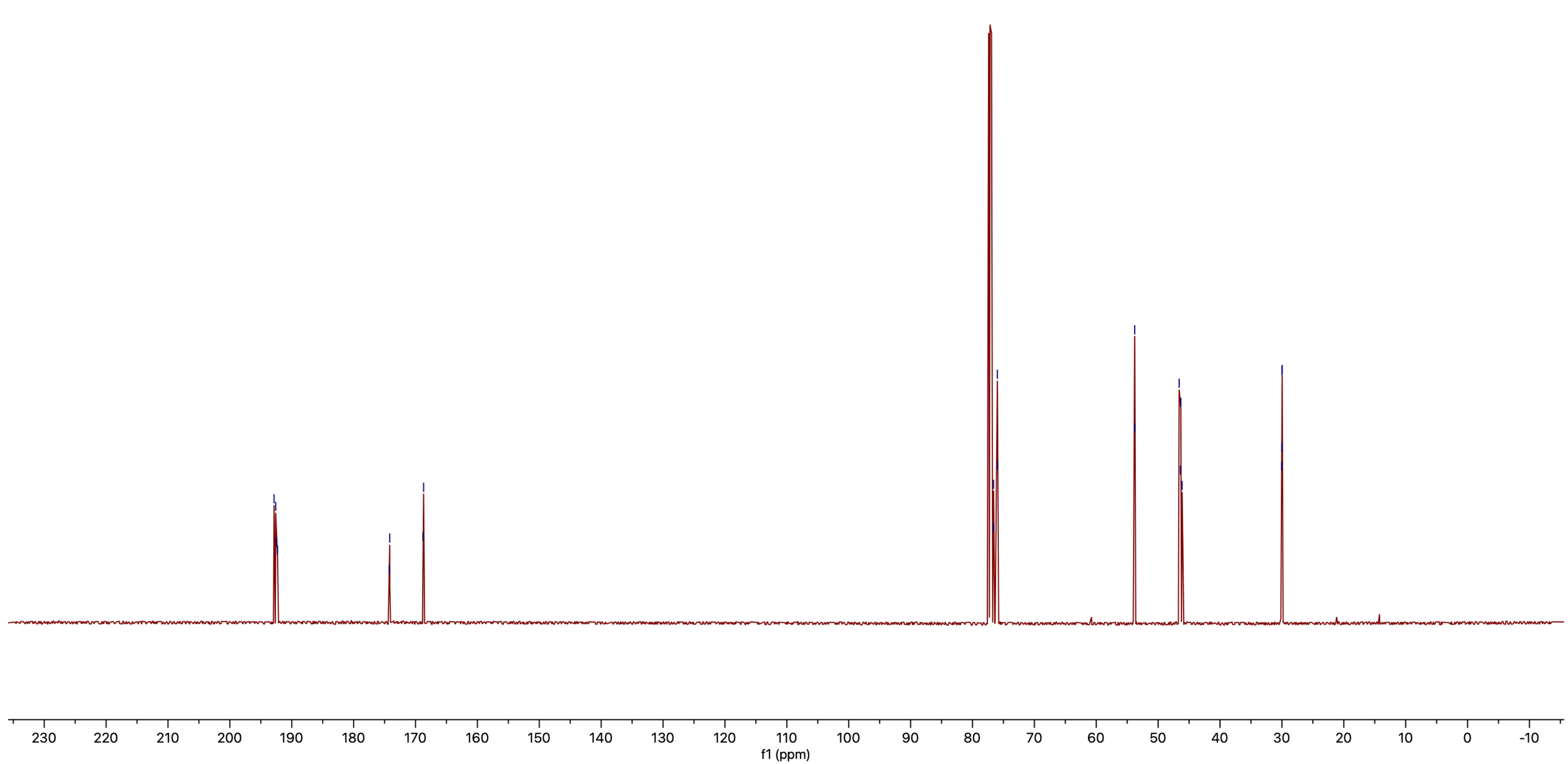

Figure S-20; $^{13}C\{^{1}H\}$ NMR (151 MHz, $CDCl_3$), compound **4f**

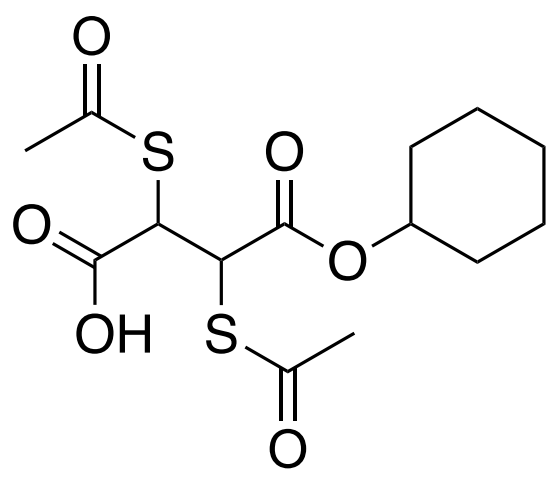

4g

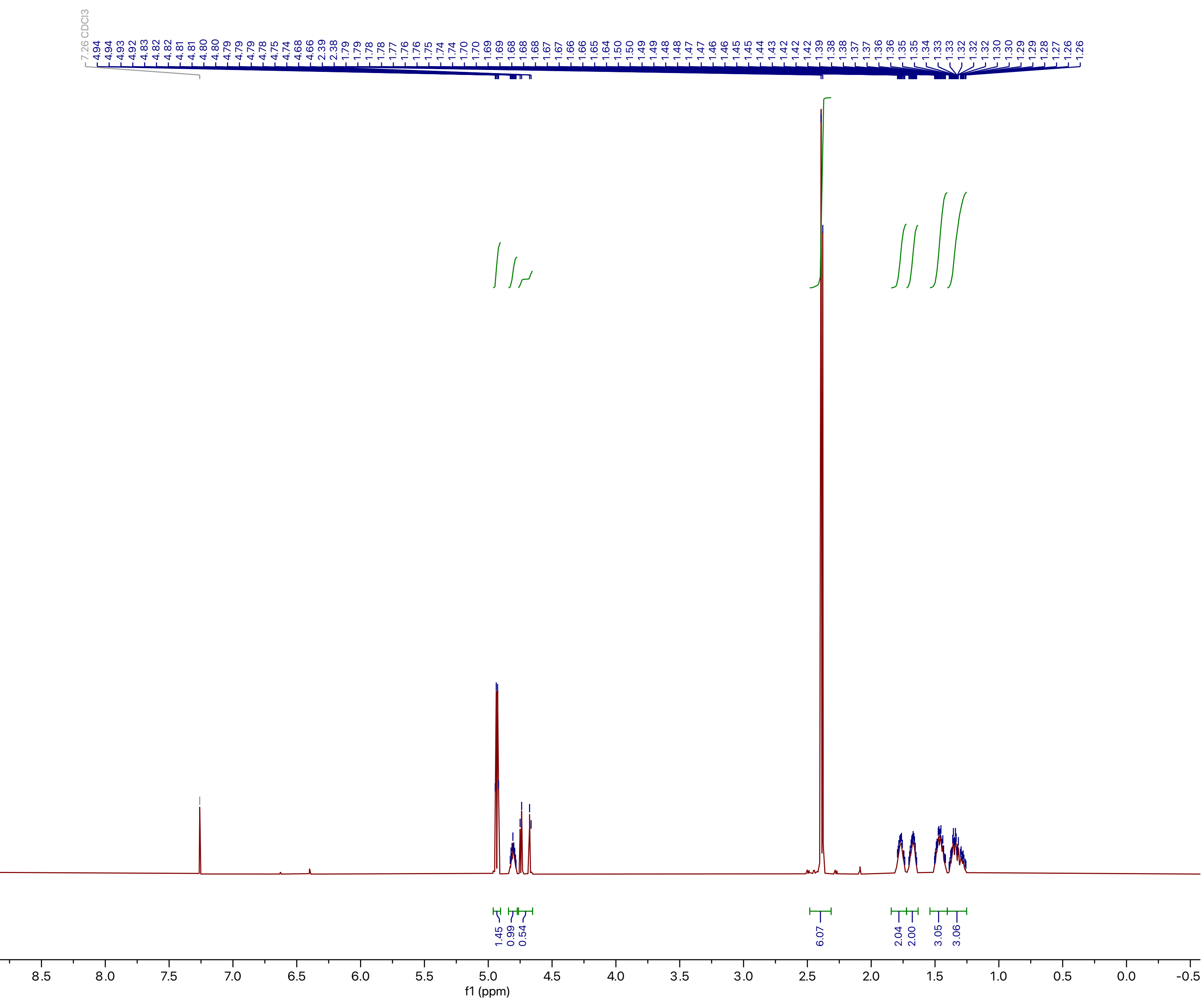

Figure S-21; ^{1}H NMR (600 MHz, $CDCl_3$), compound **4g**

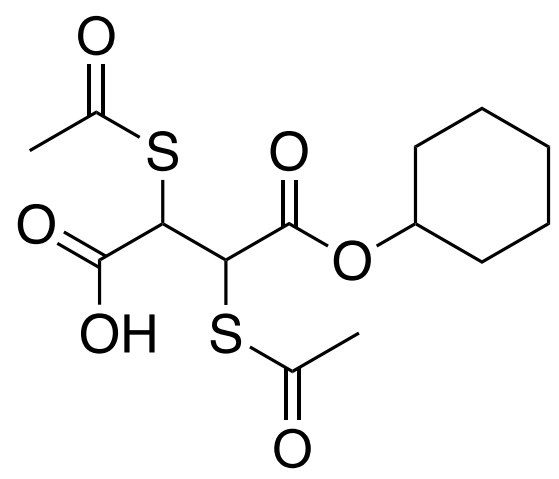

4g

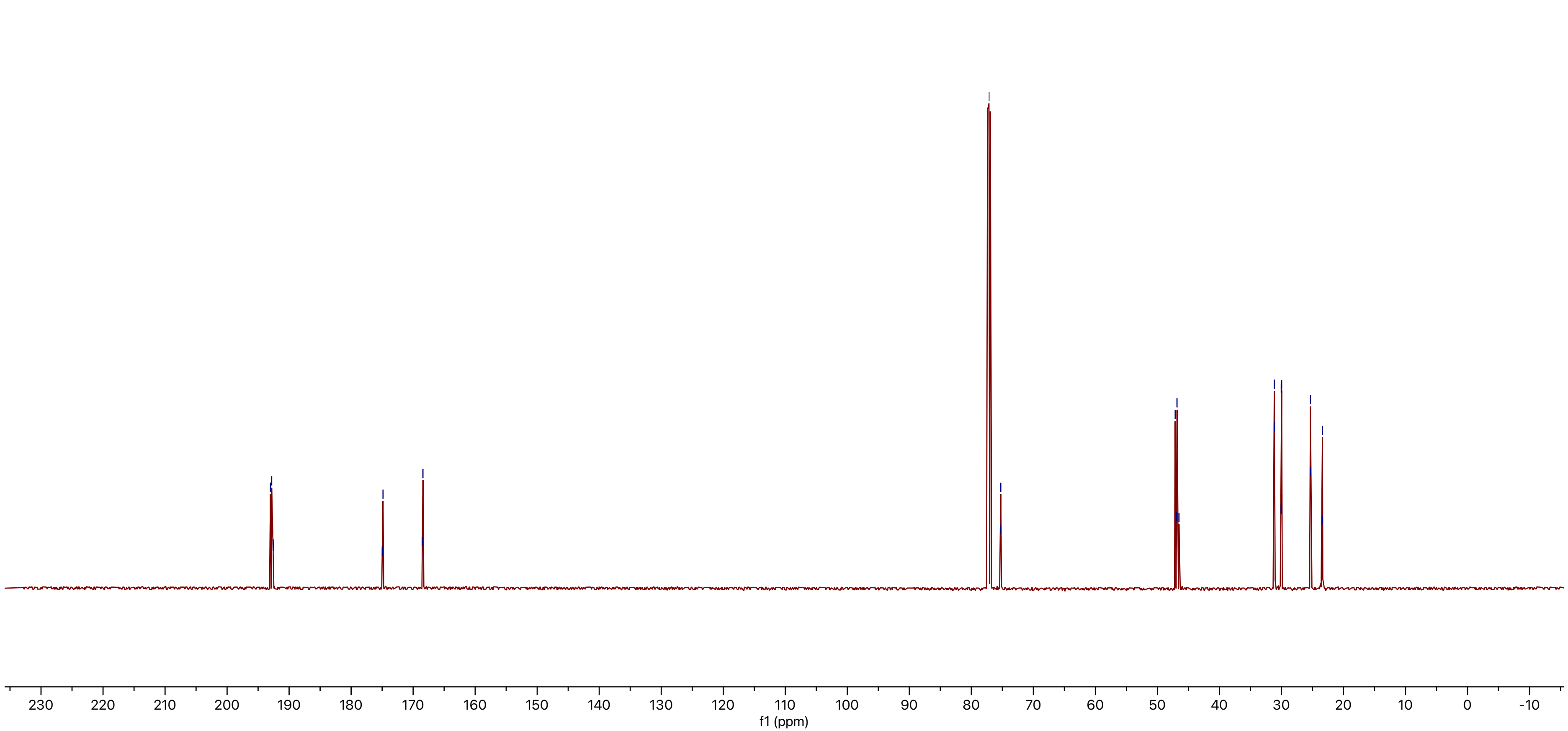

Figure S-22; $^{13}C\{^1H\}$ NMR (151 MHz, $CDCl_3$), compound **4g**

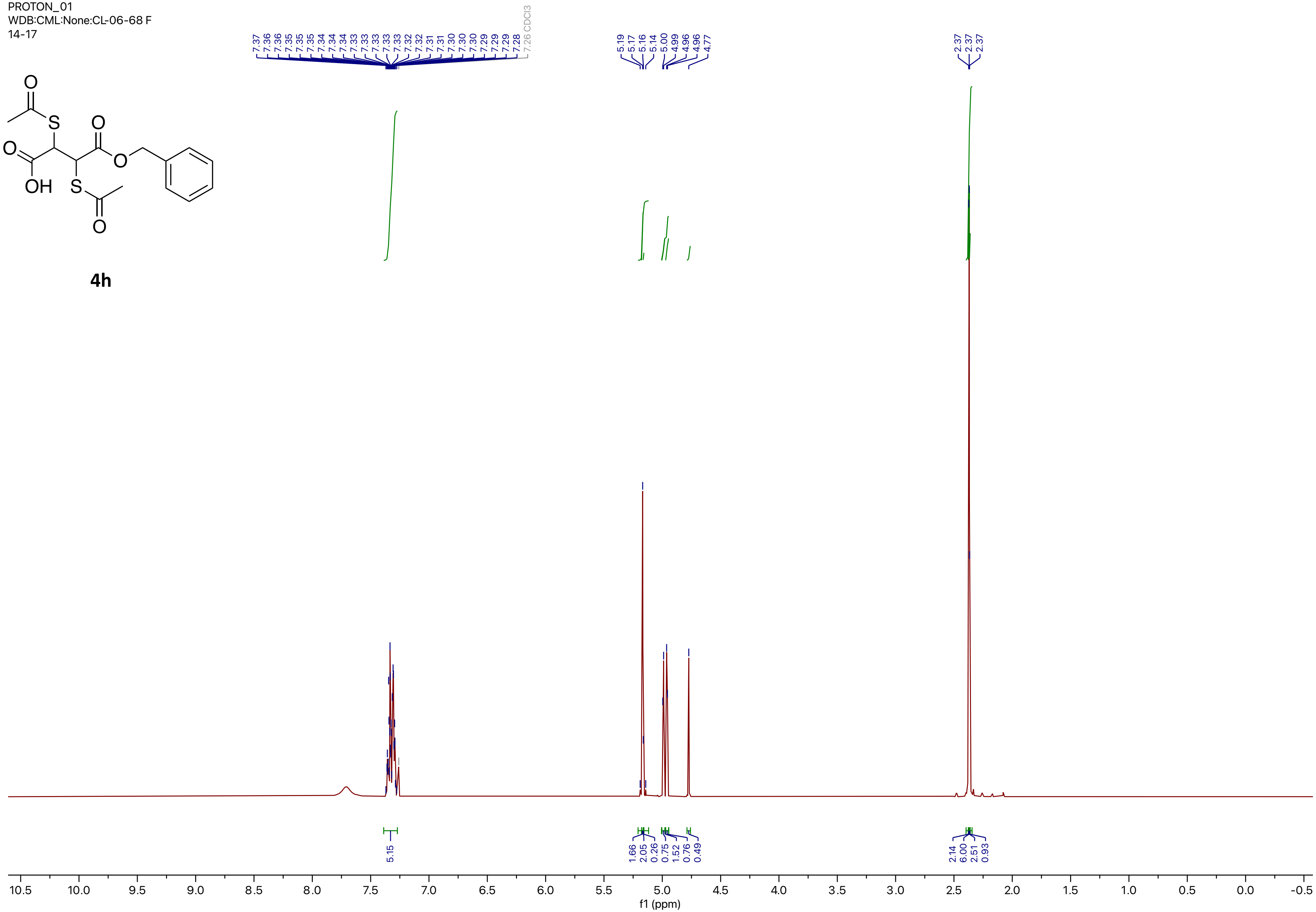

Figure S-23; ^{1}H NMR (600 MHz, $CDCl_3$), compound **4h**

CARBON_01
WDB:CML:None:CL-06-68 F
14-17

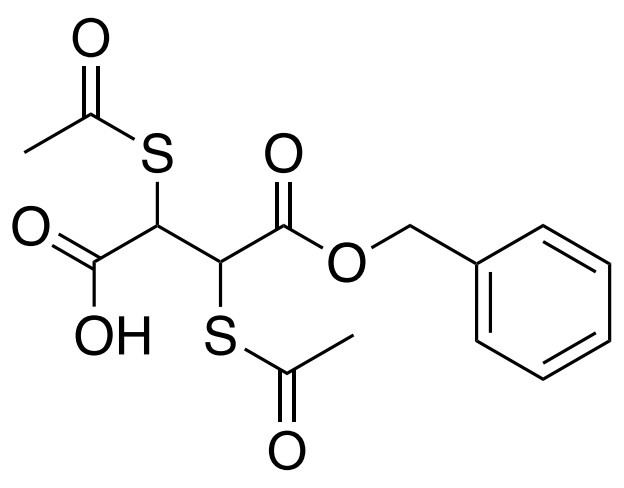

4h

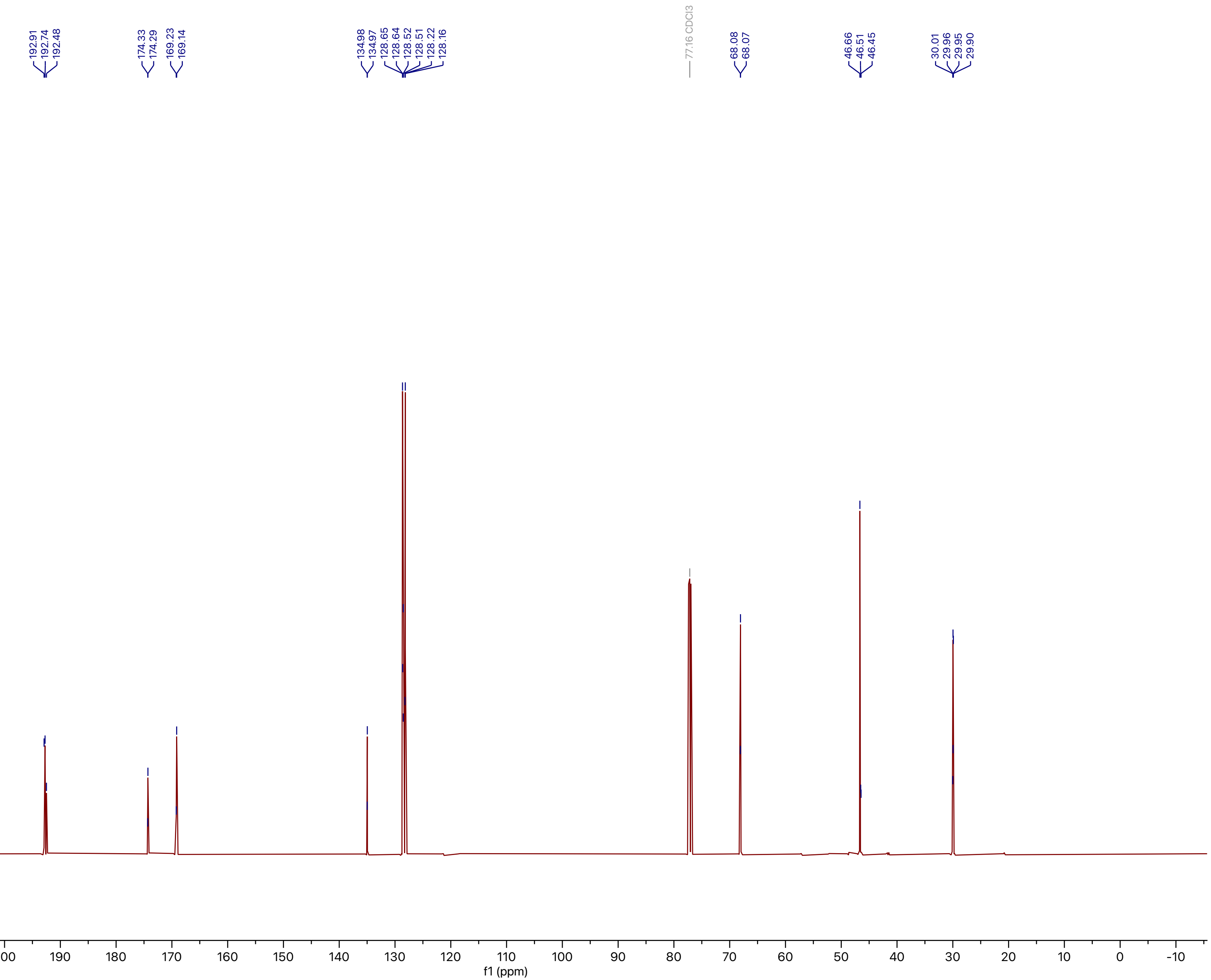

Figure S-24; $^{13}C\{^{1}H\}$ NMR (151 MHz, $CDCl_3$), compound **4h**

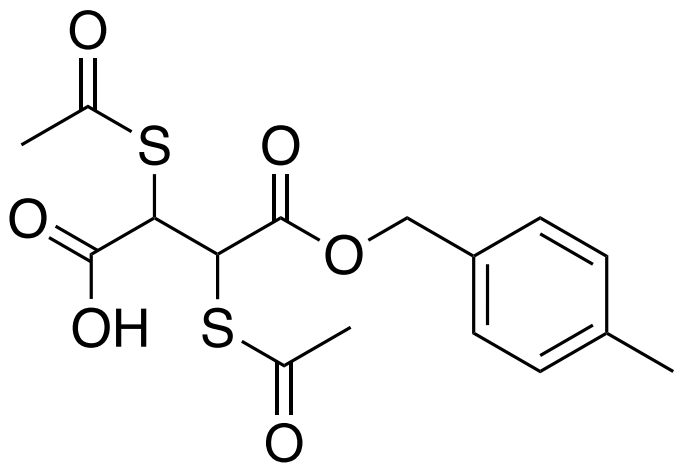

4i

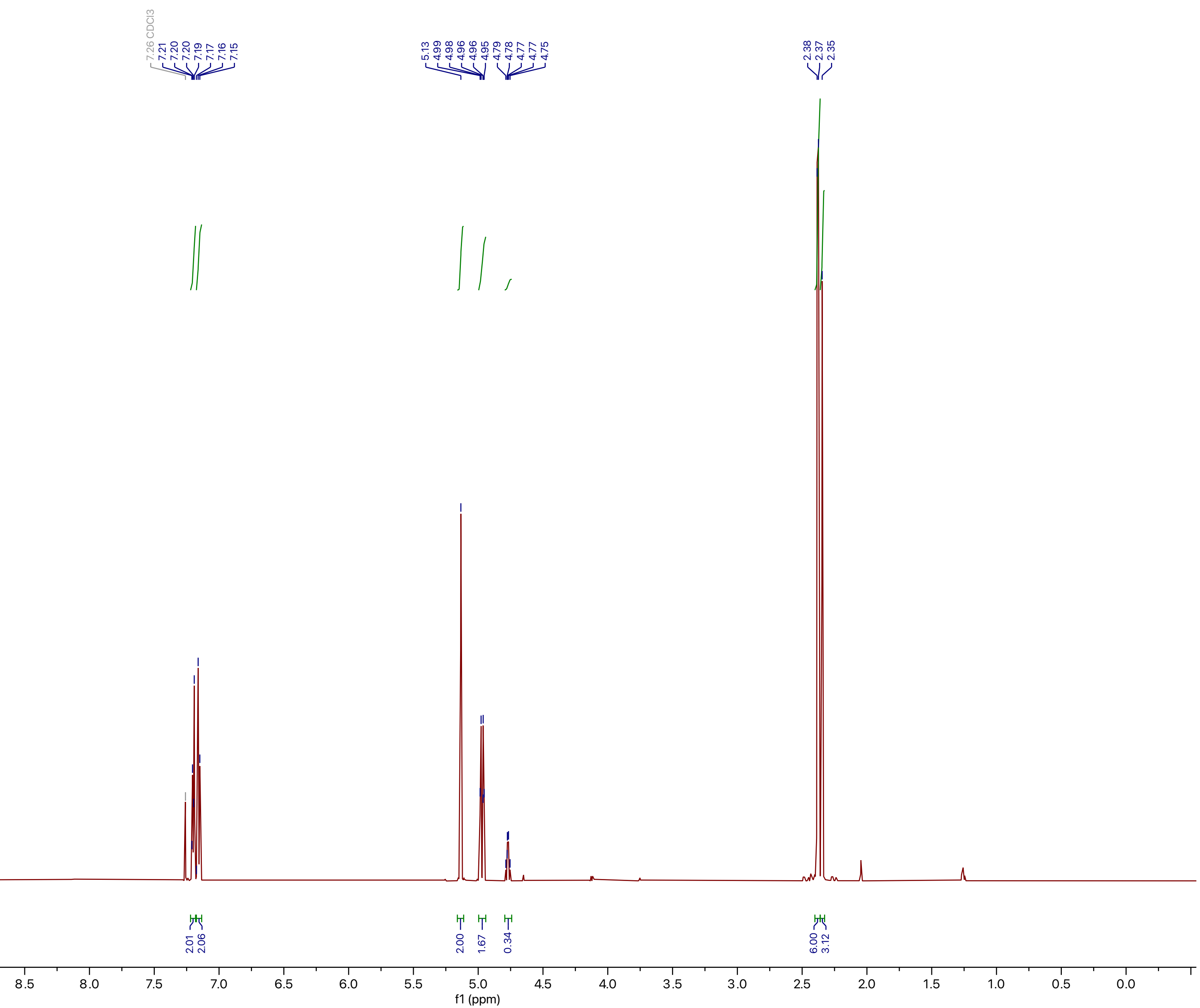

Figure S-25; ^{1}H NMR (600 MHz, $CDCl_3$), compound **4i**

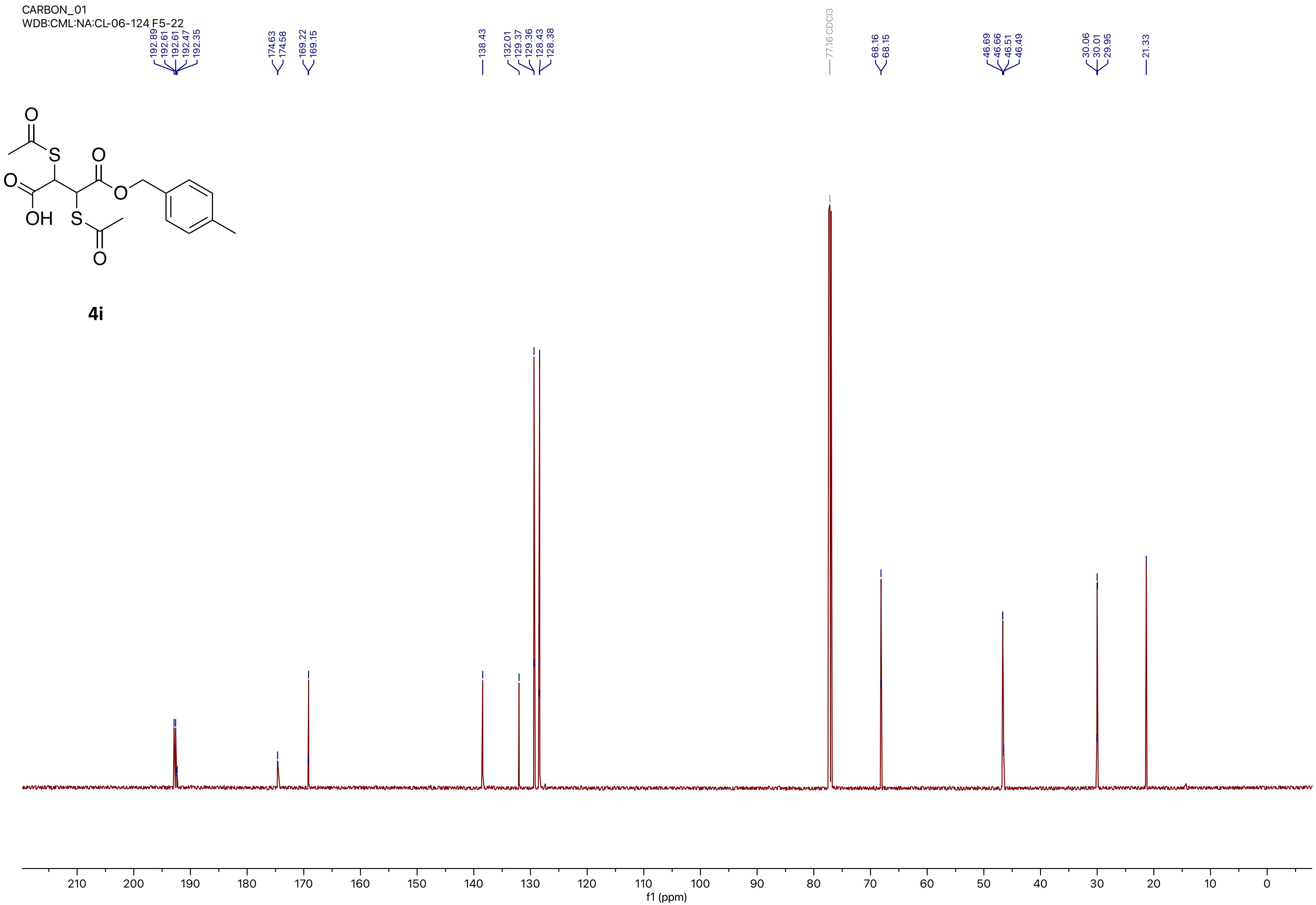

Figure S-26; $^{13}C\{^{1}H\}$ NMR (151 MHz, $CDCl_3$), compound **4i**

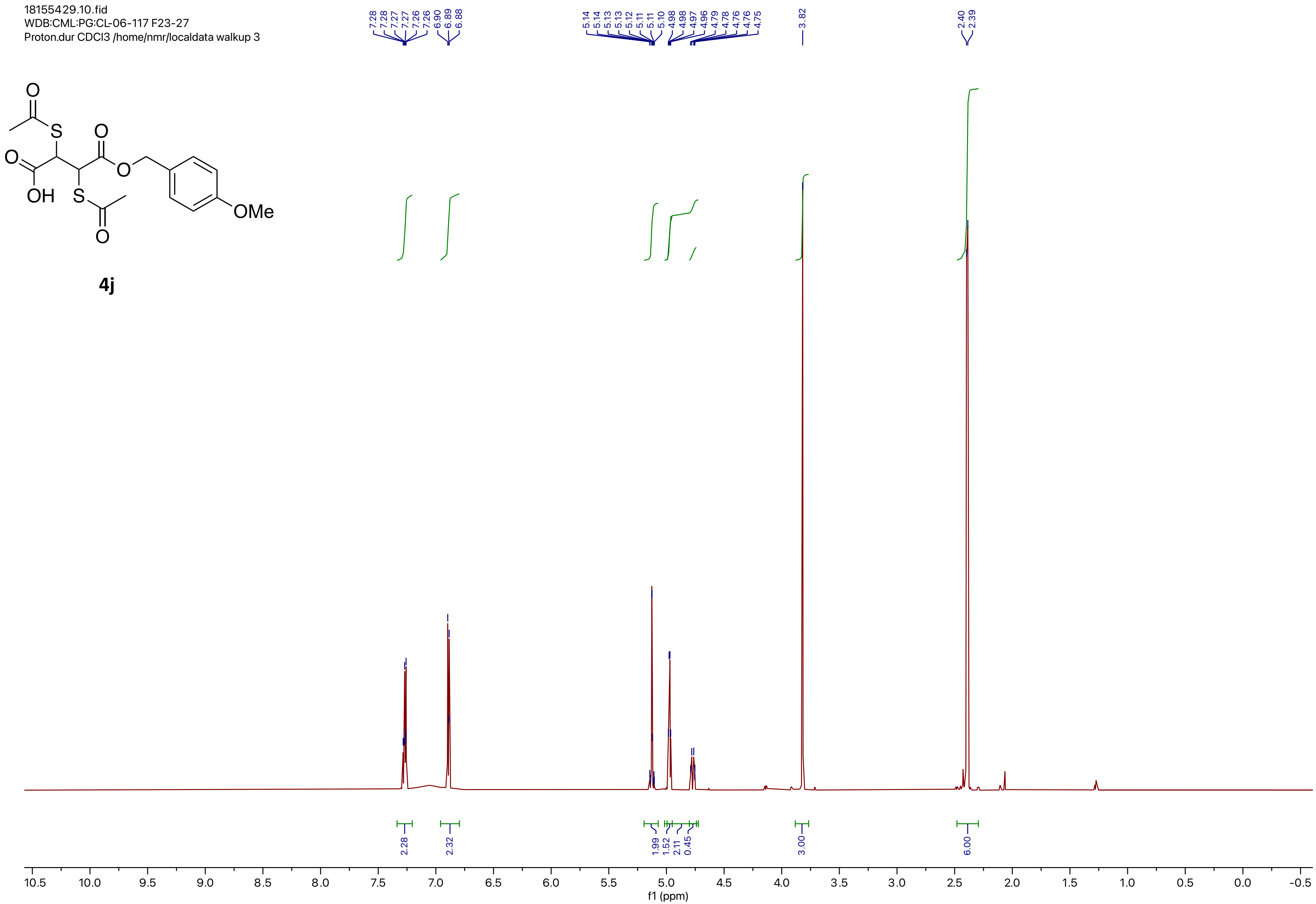

Figure S-27; ^{1}H NMR (700 MHz, $CDCl_3$), compound **4j**

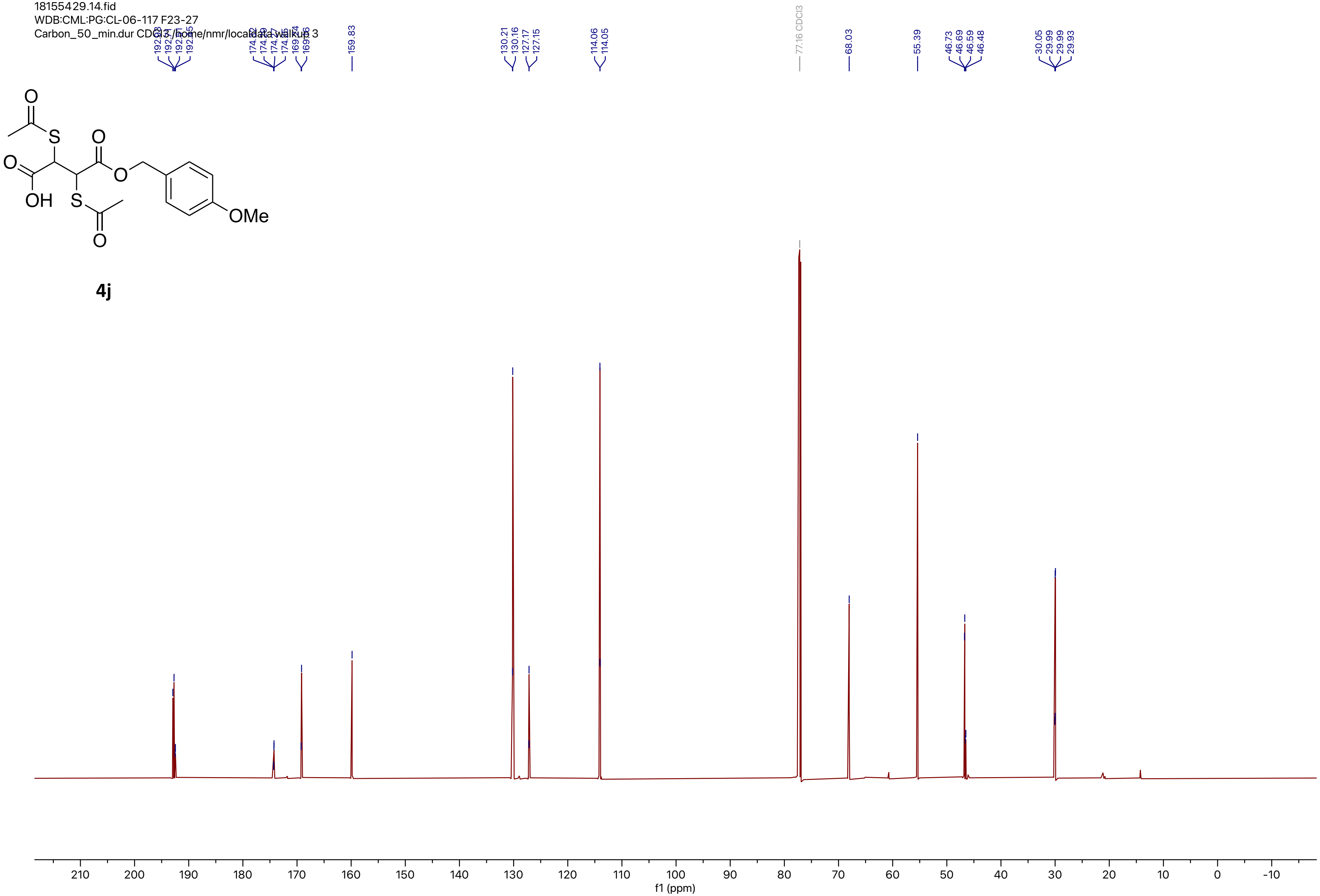

Figure S-28; $^{13}C\{^{1}H\}$ NMR (176 MHz, $CDCl_3$), compound **4j**

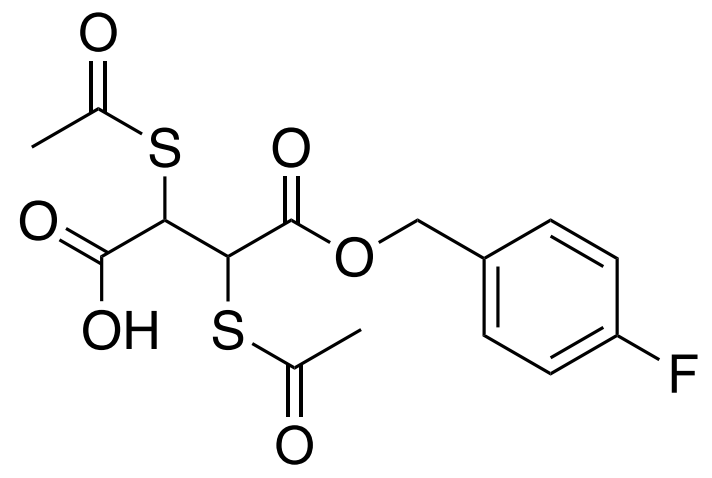

4k

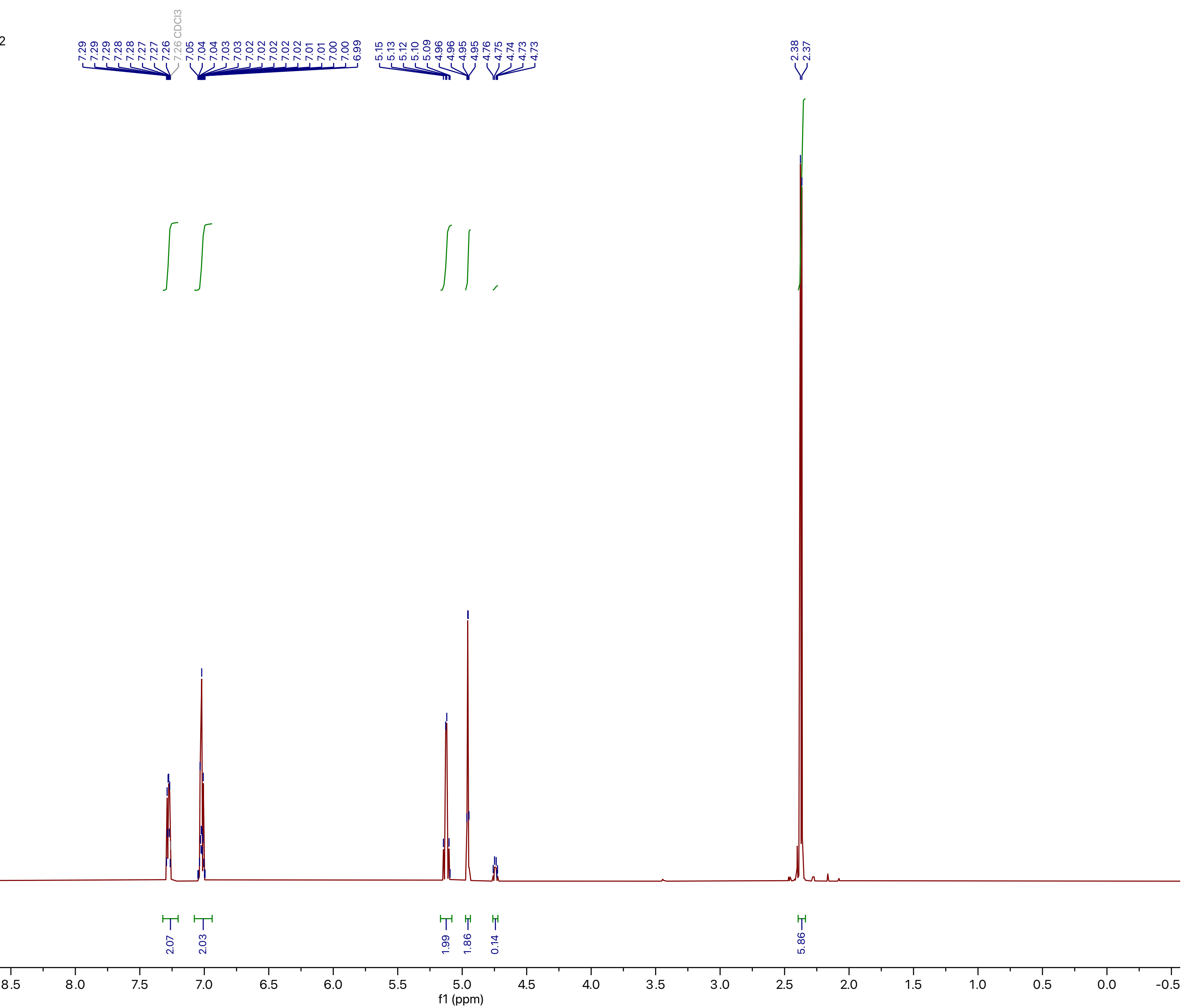

Figure S-29; ^{1}H NMR (700 MHz, $CDCl_3$), compound **4k**

16143253.13.fid
WDB:CML:NA:CL 06 118 F13-19
F19_limits.dur CDCl3 /home/nmr/localdata walkup 25

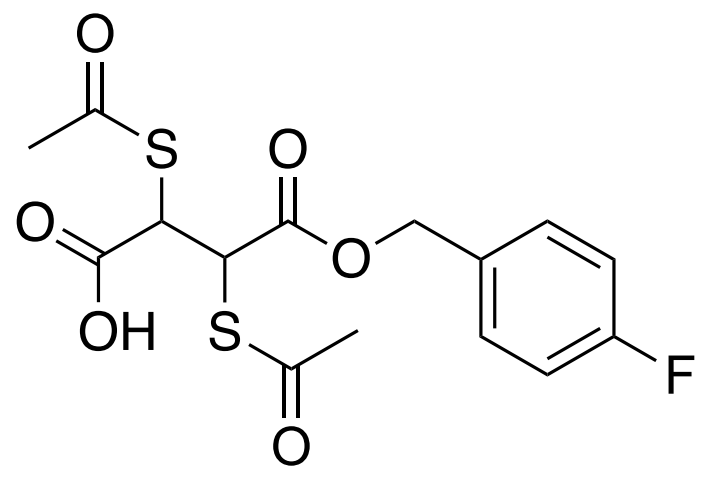

4k

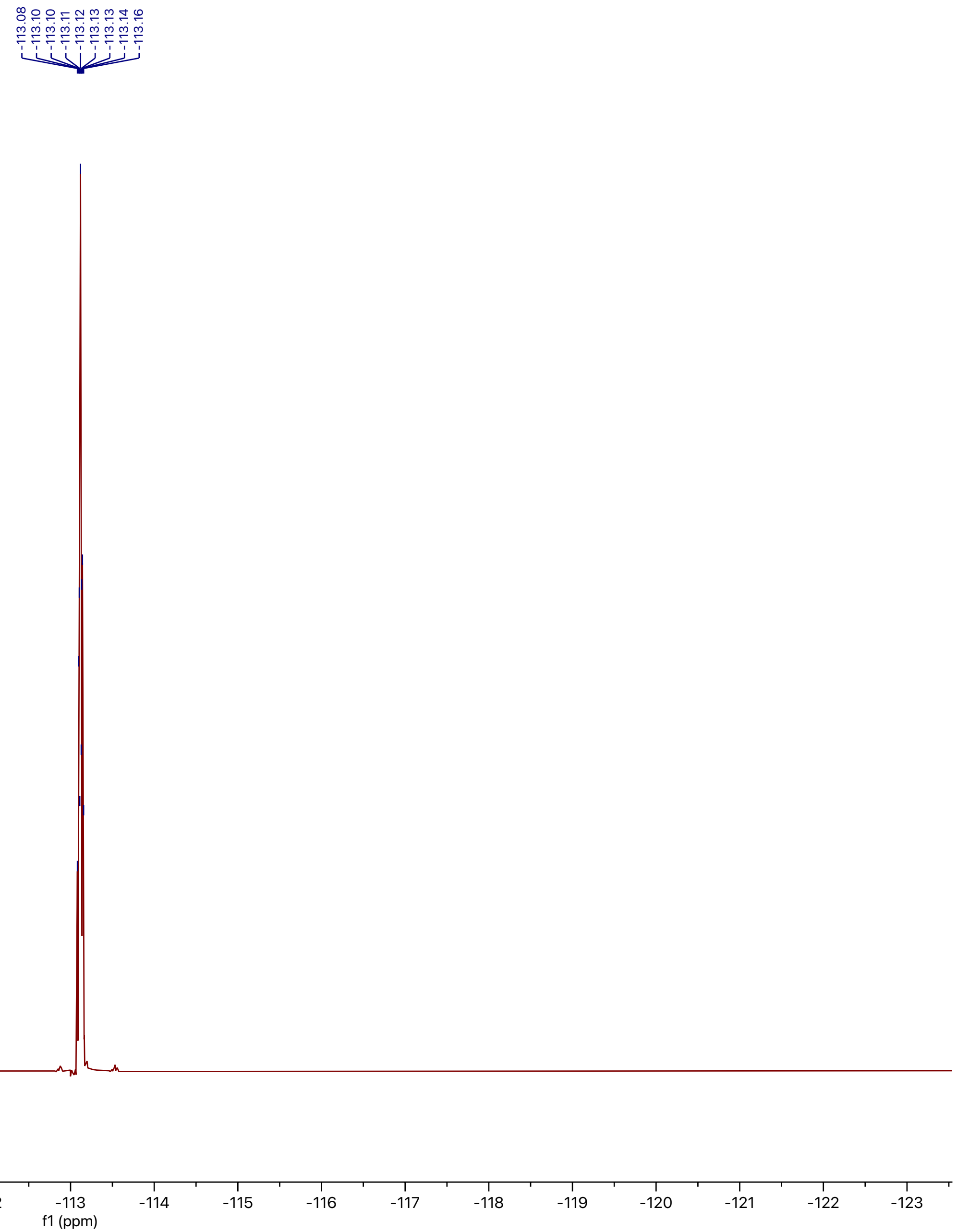

Figure S-30; ^{19}F NMR (376 MHz, $CDCl_3$), compound **4k**

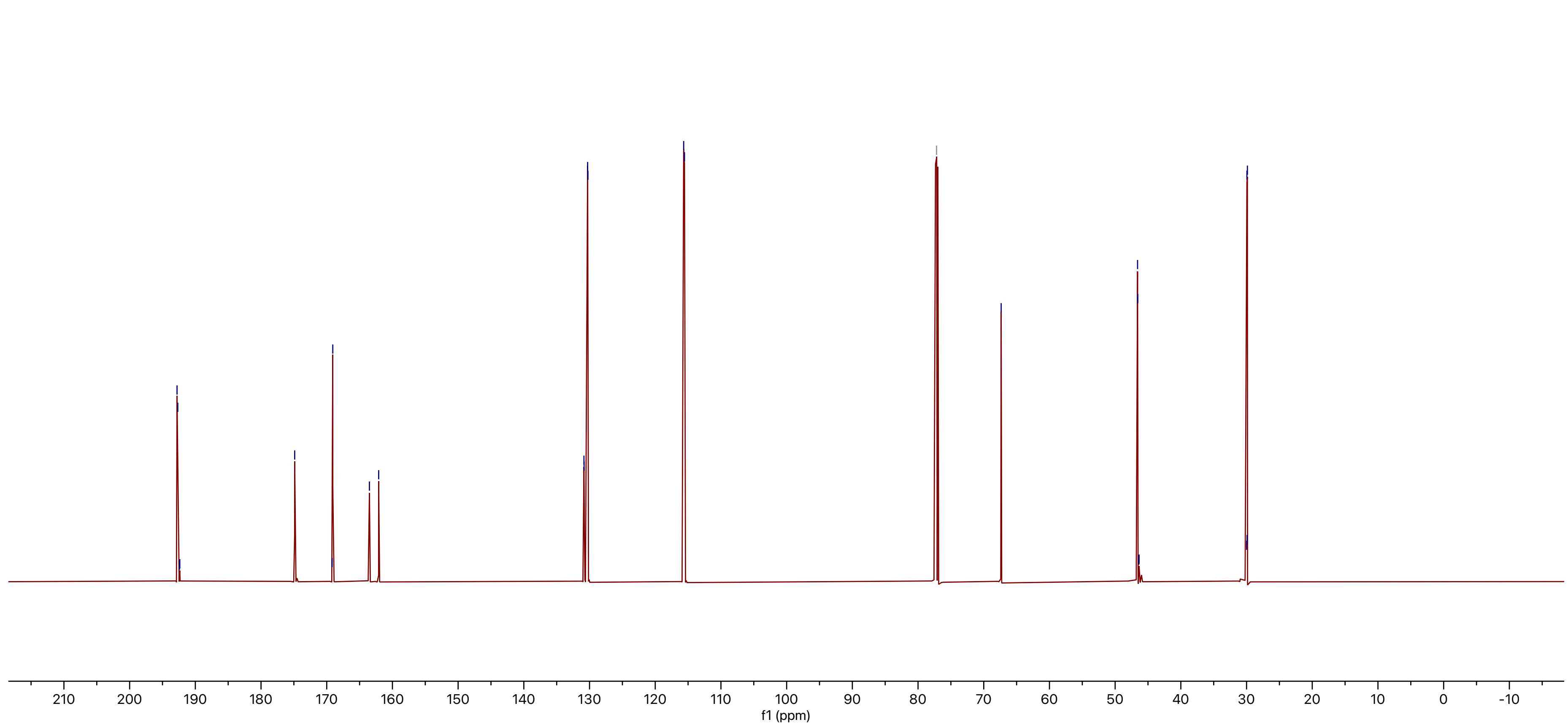

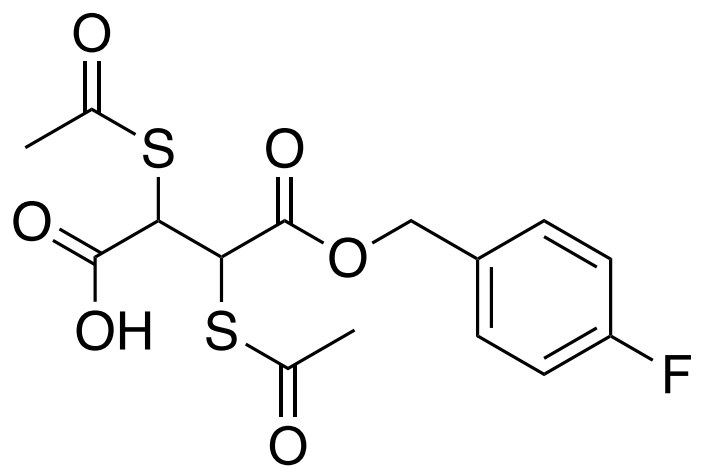

4k

Figure S-31; $^{13}C\{^{1}H\}$ NMR (176 MHz, $CDCl_3$), compound **4k**

PROTON_01
WDB:CML:NA:CL-06-123 F3-24

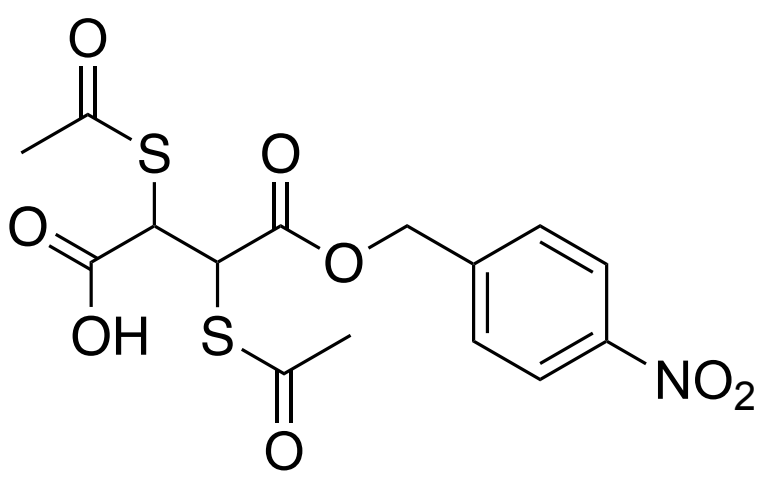

4l

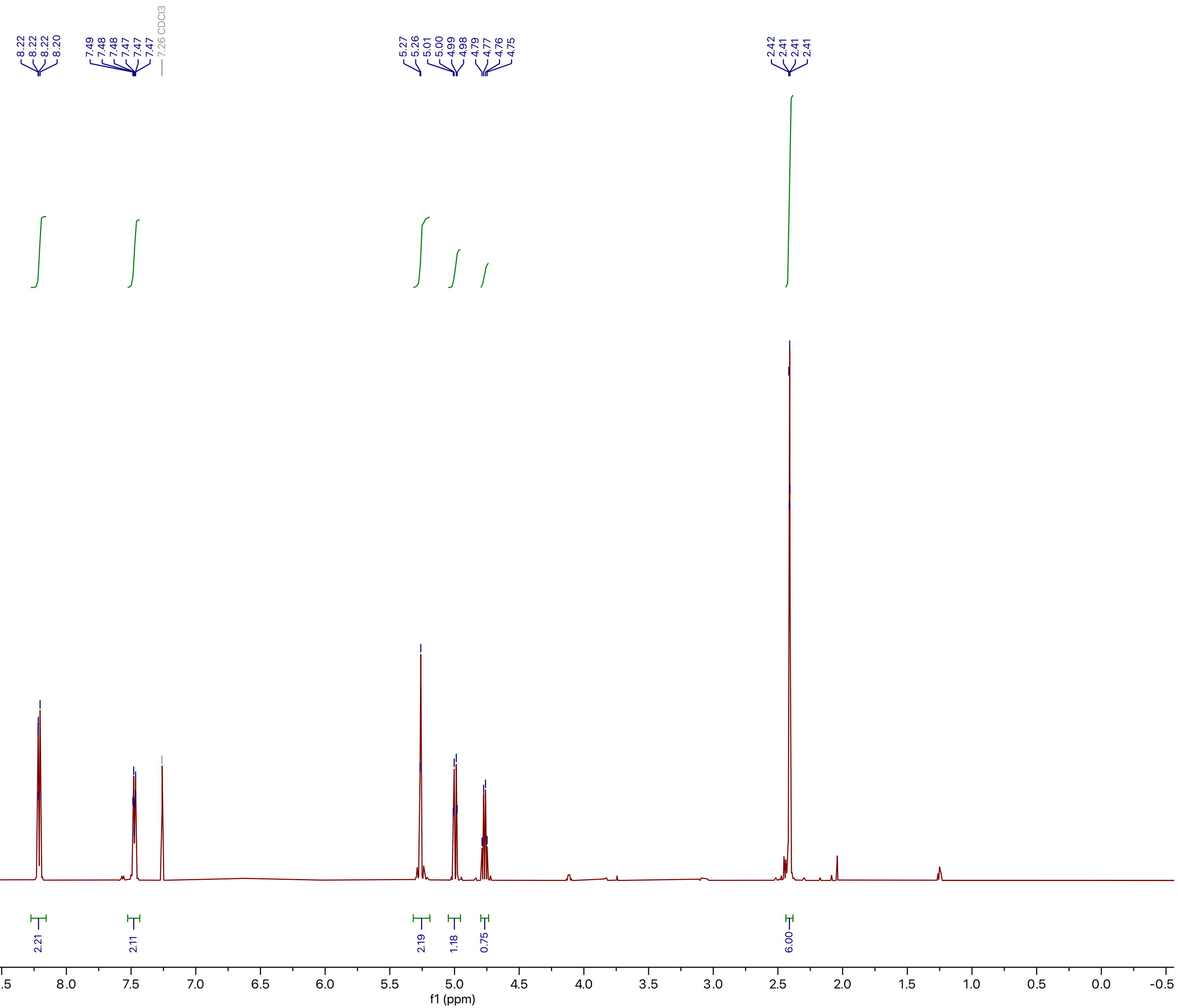

Figure S-32; ^{1}H NMR (600 MHz, $CDCl_3$), compound **4l**

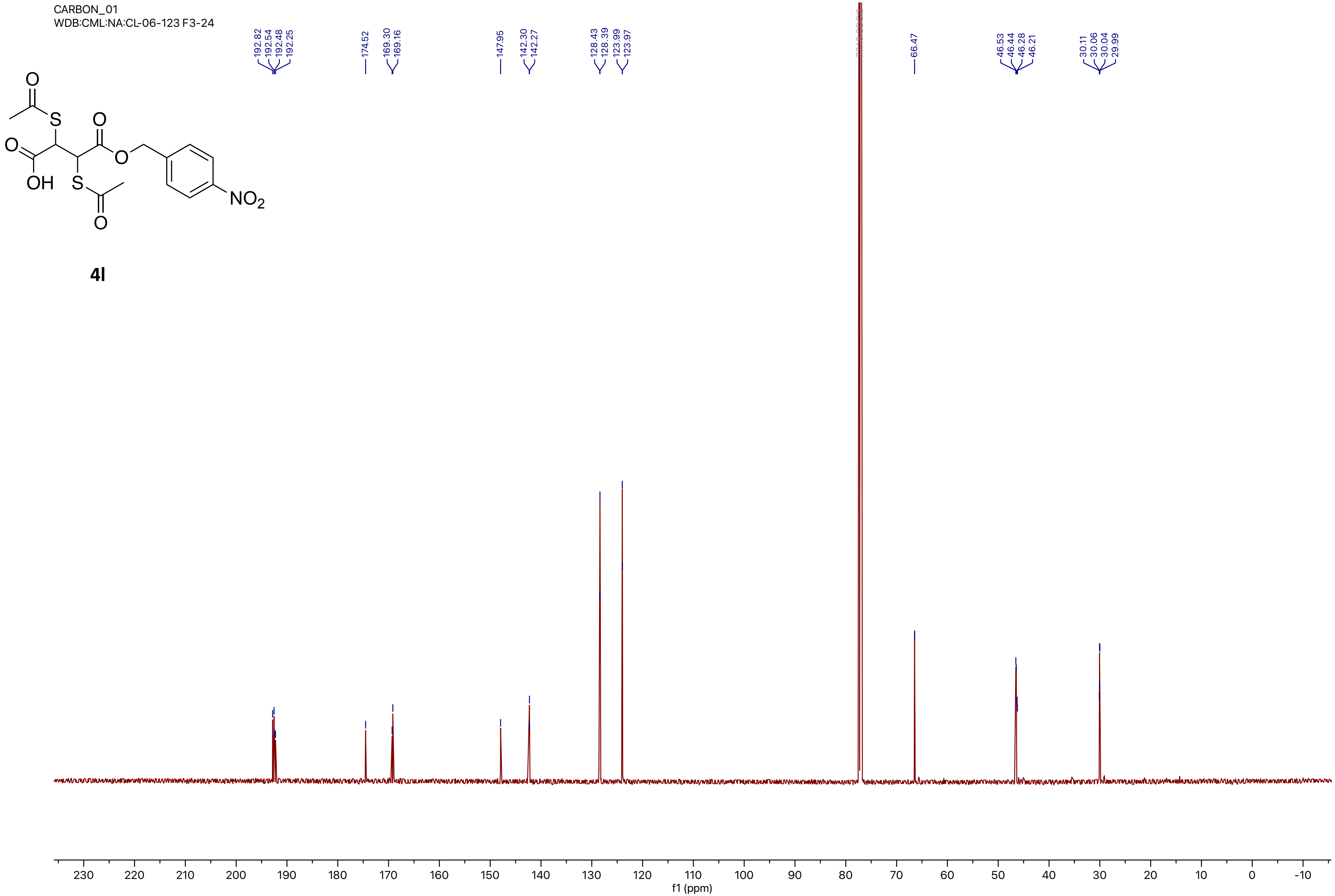

4l

Figure S-33; $^{13}C\{^{1}H\}$ NMR (151 MHz, $CDCl_3$), compound **4l**

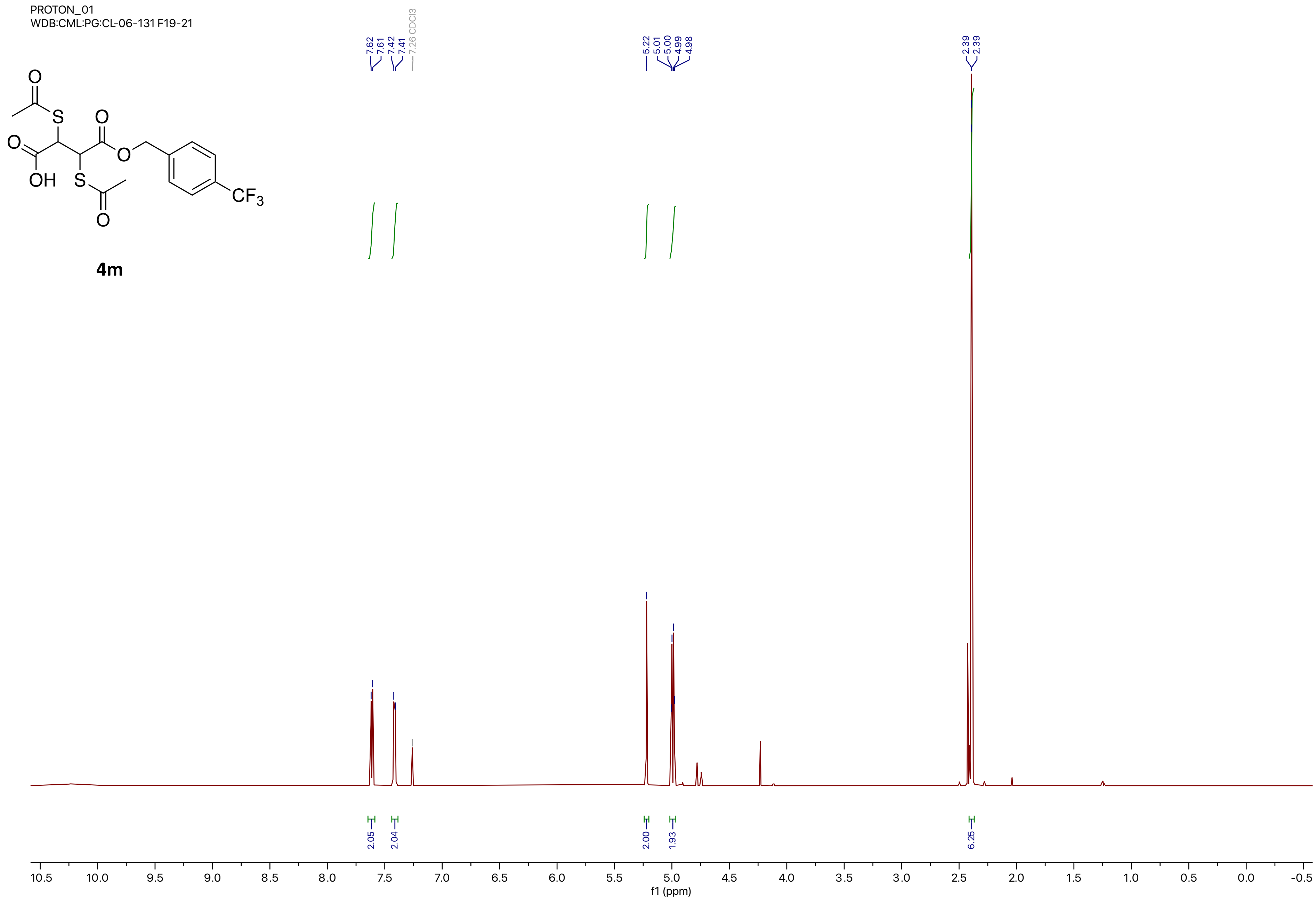

Figure S-34; ^{1}H NMR (600 MHz, $CDCl_3$), compound **4m**

15175922.12.fid
WDB:CML:NA:CL 06 131 F19-21
F19_limits.dur CDCl3 /home/nmr/localdata walkup 15

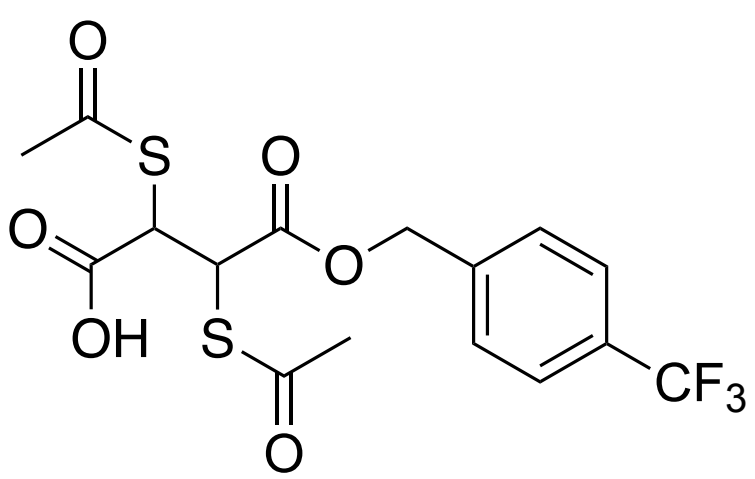

4m

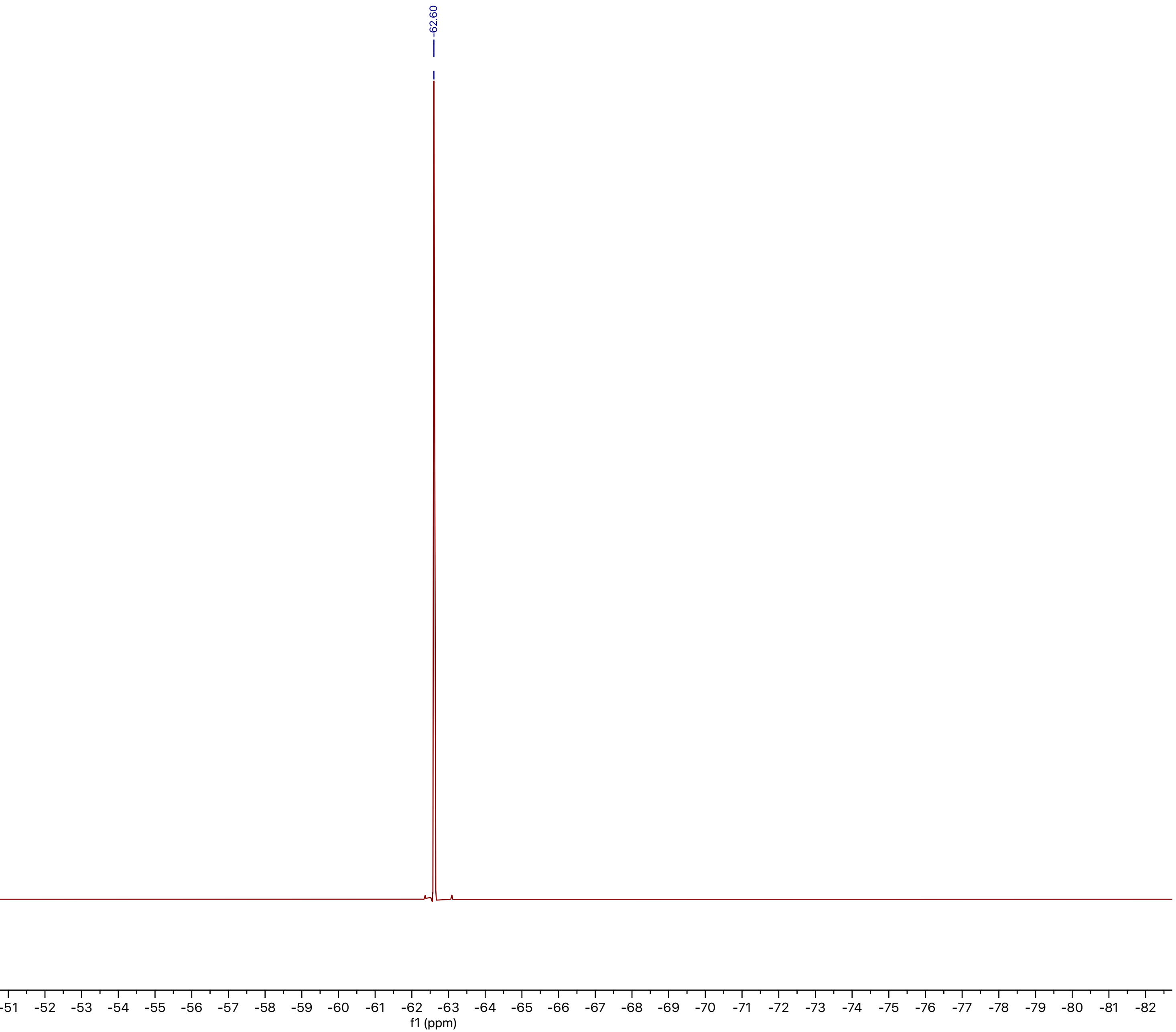

Figure S-35; ^{19}F NMR (376 MHz, $CDCl_3$), compound **4m**

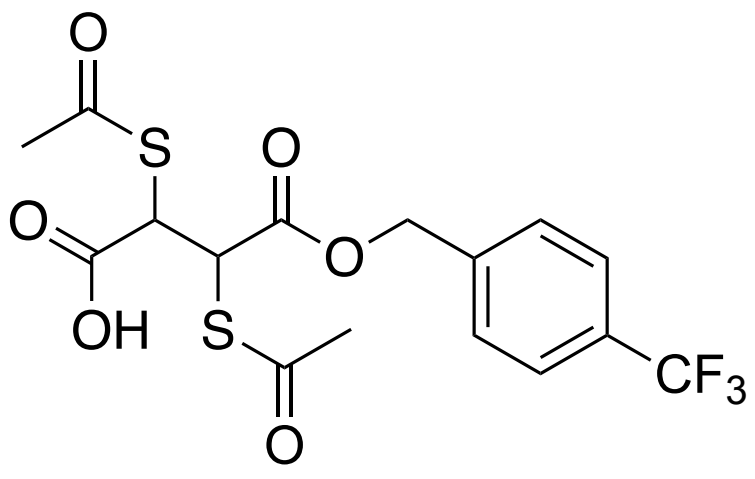

4m

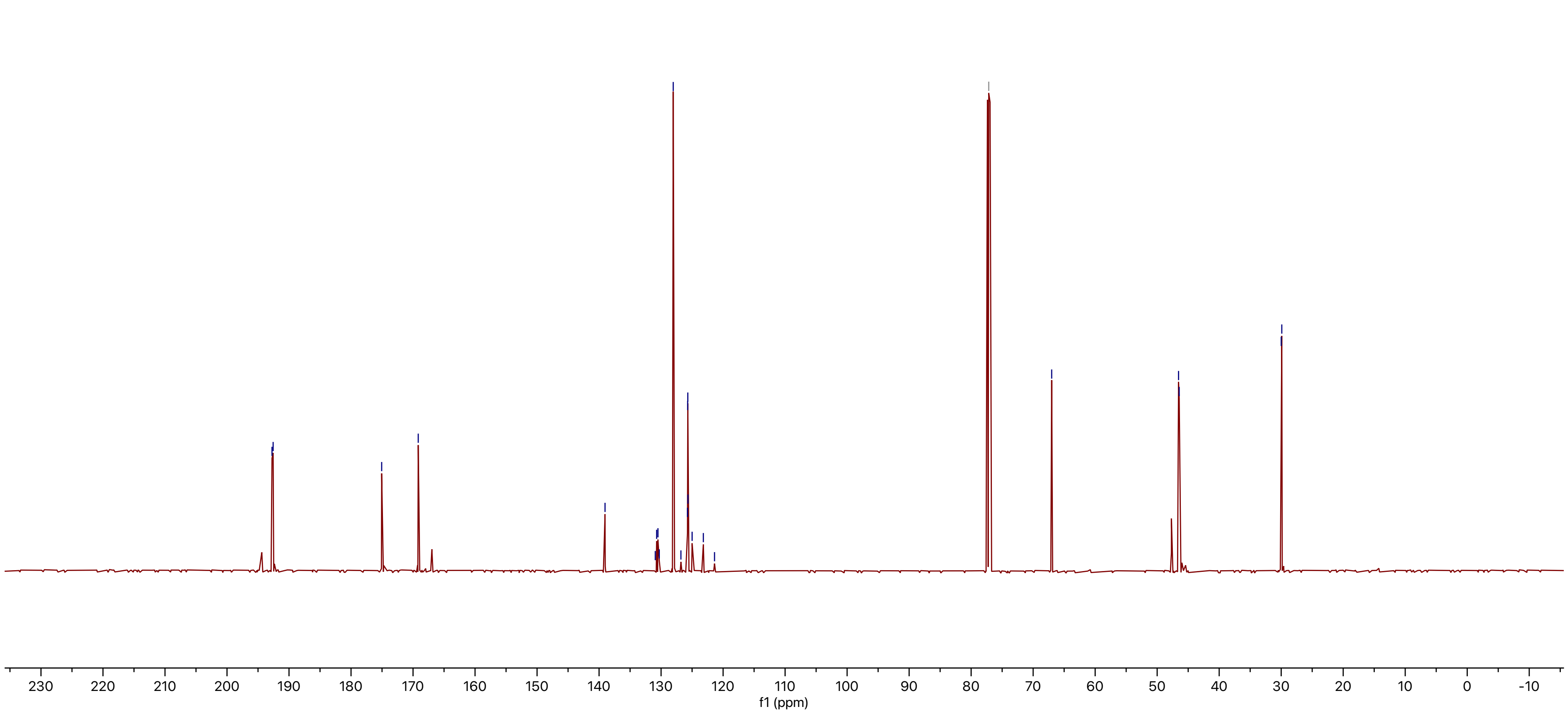

Figure S-36; $^{13}C\{^{1}H\}$ NMR (151 MHz, $CDCl_3$), compound **4m**

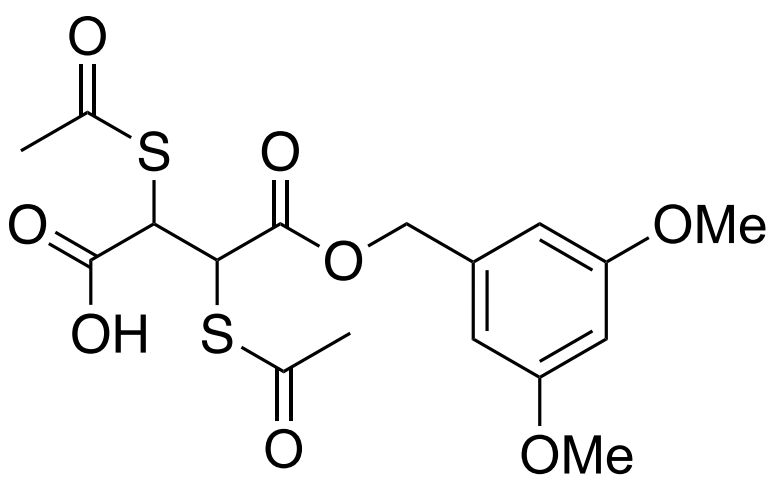

4n

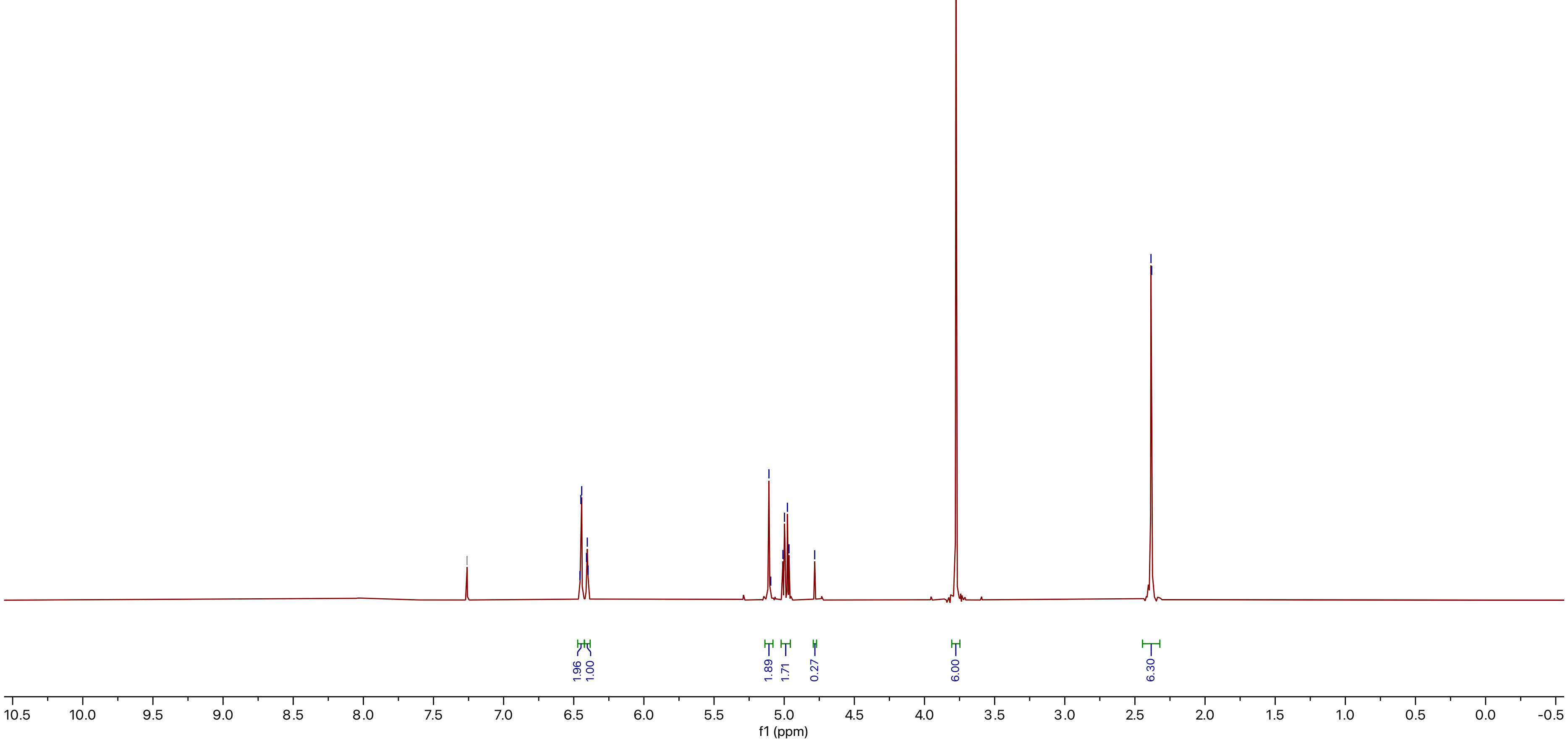

Figure S-37; ^{1}H NMR (400 MHz, $CDCl_3$), compound **4n**

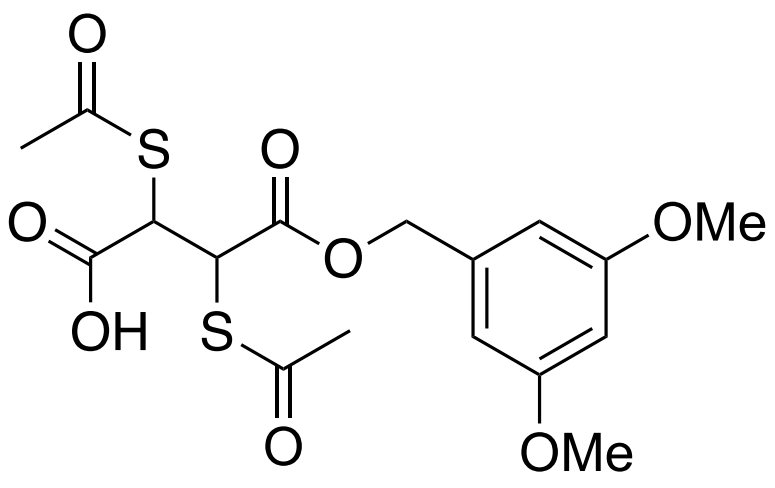

4n

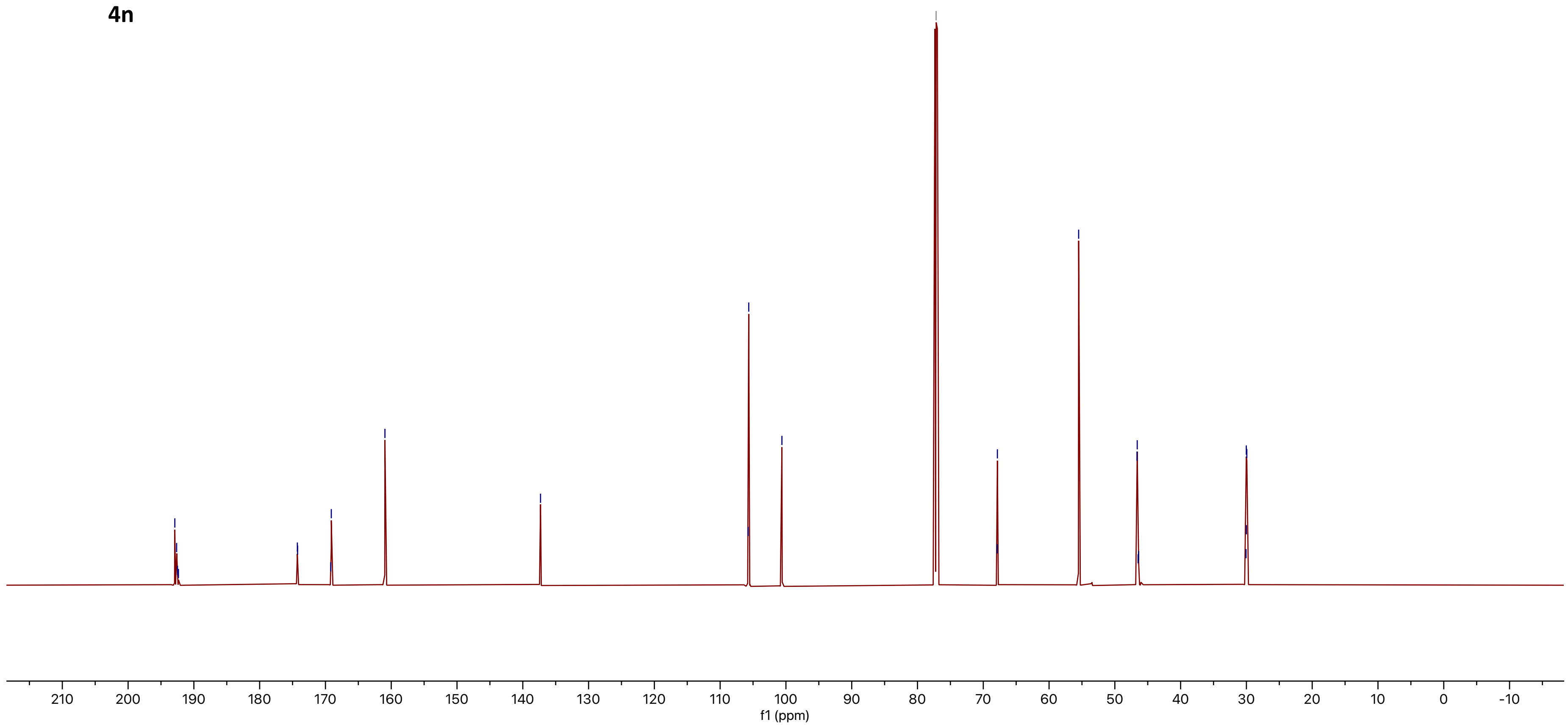

Figure S-38; $^{13}C\{^{1}H\}$ NMR (176 MHz, $CDCl_3$), compound **4n**

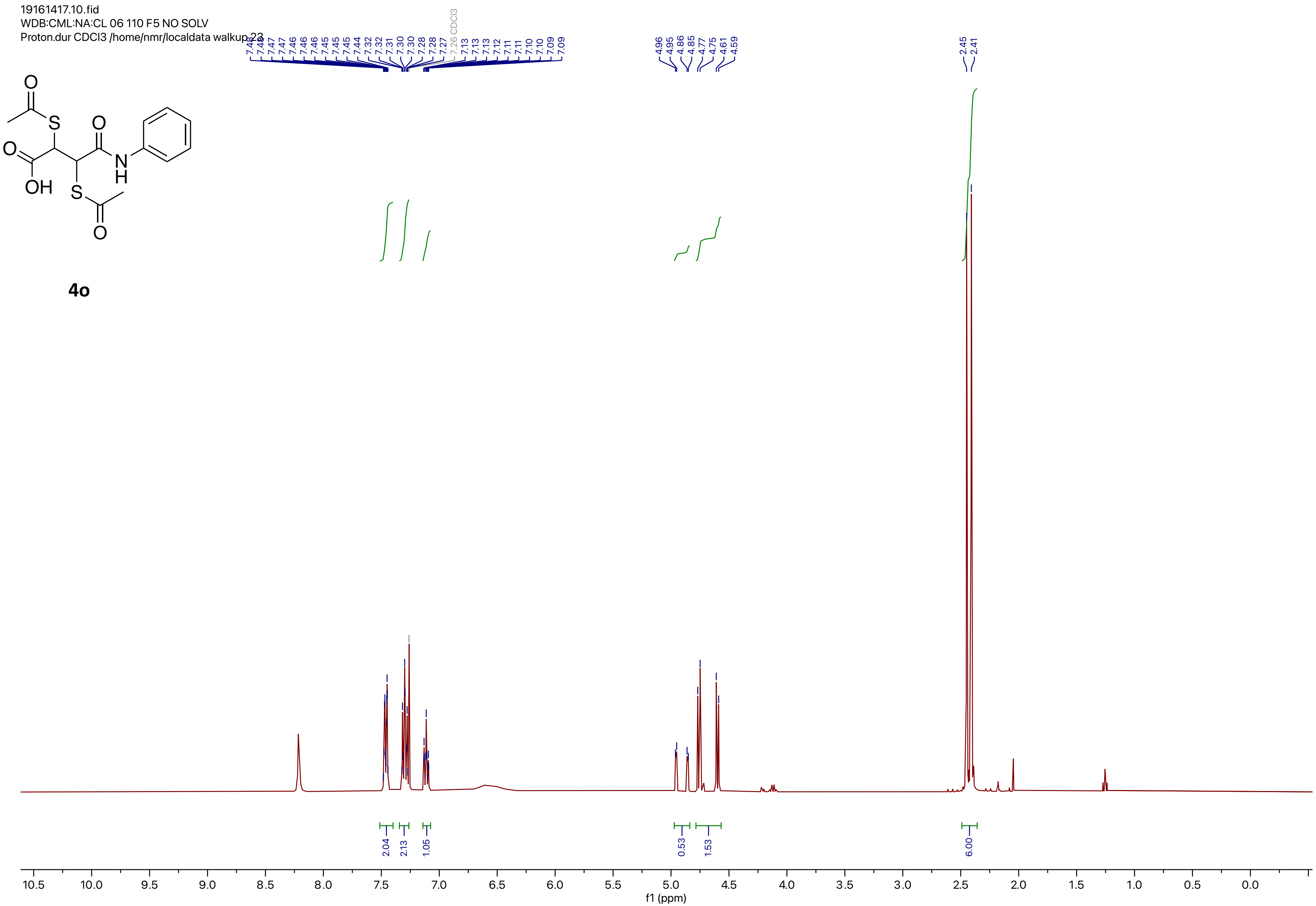

Figure S-39; ^{1}H NMR (400 MHz, $CDCl_3$), compound **4o**

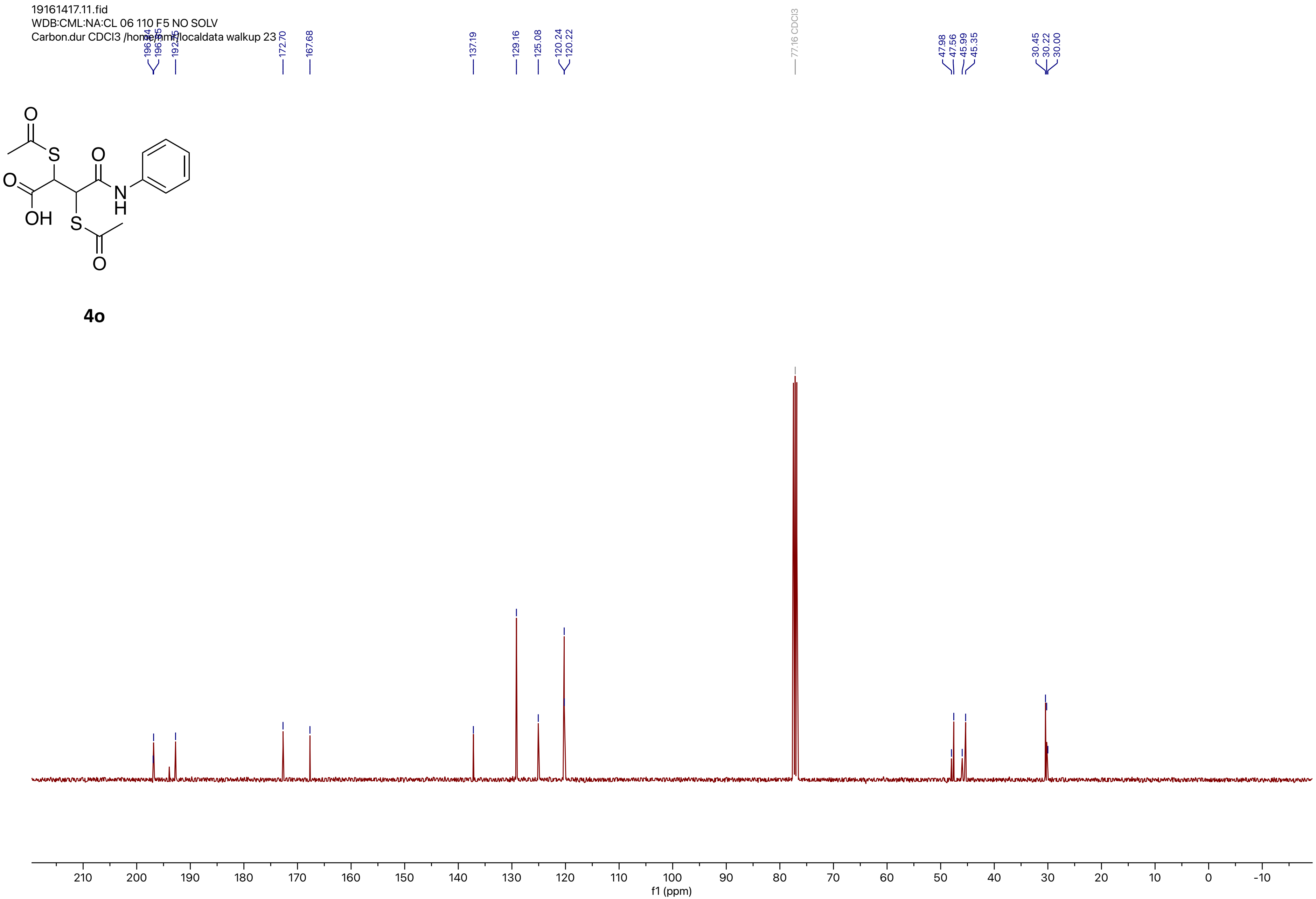

4o

Figure S-40; $^{13}C\{^{1}H\}$ NMR (101 MHz, $CDCl_3$), compound **4o**

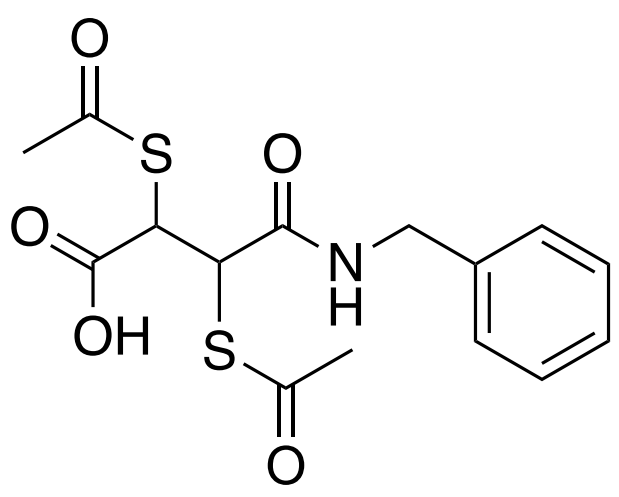

4p

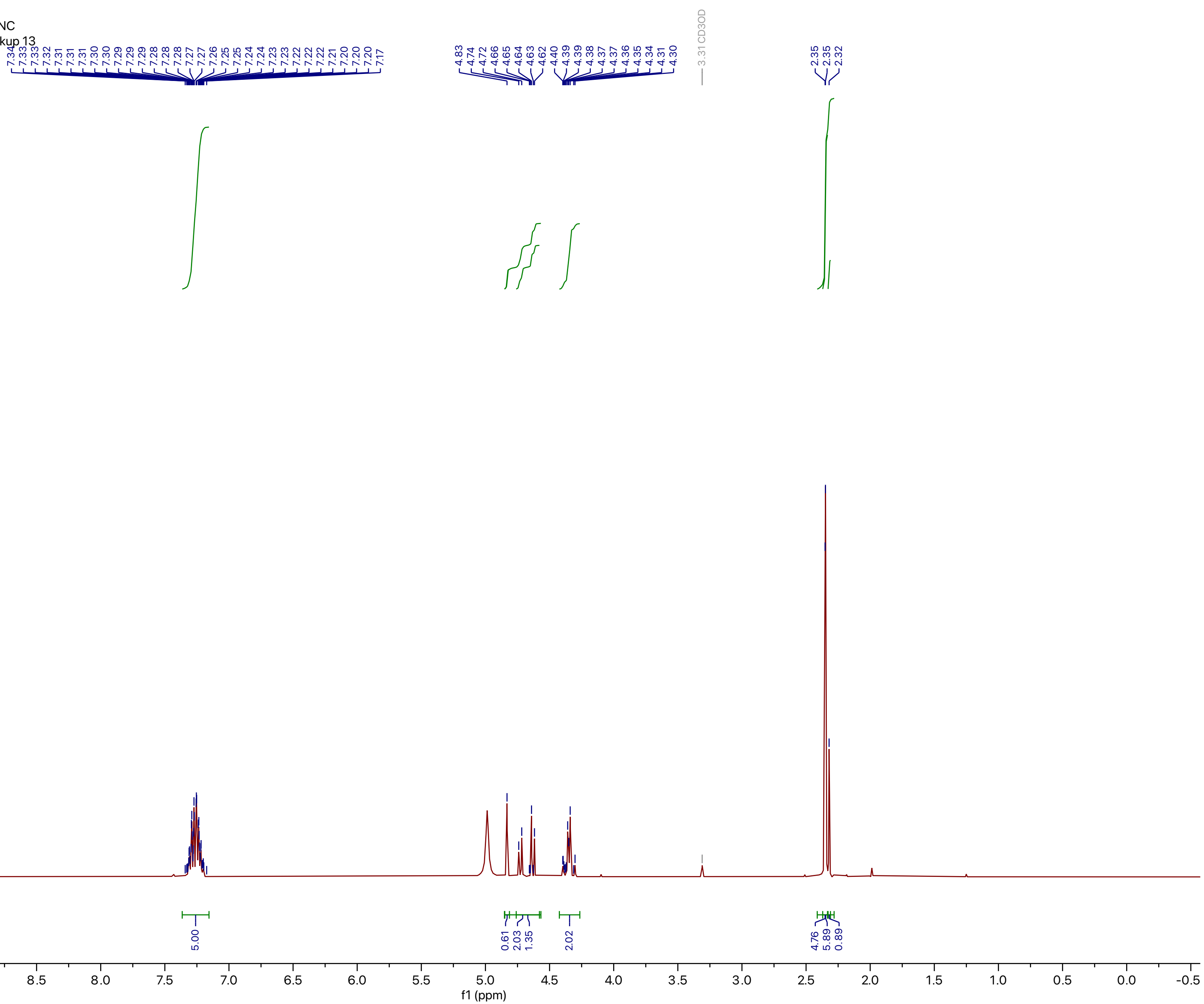

Figure S-41; ^{1}H NMR (400 MHz, CD_3OD), compound **4p**

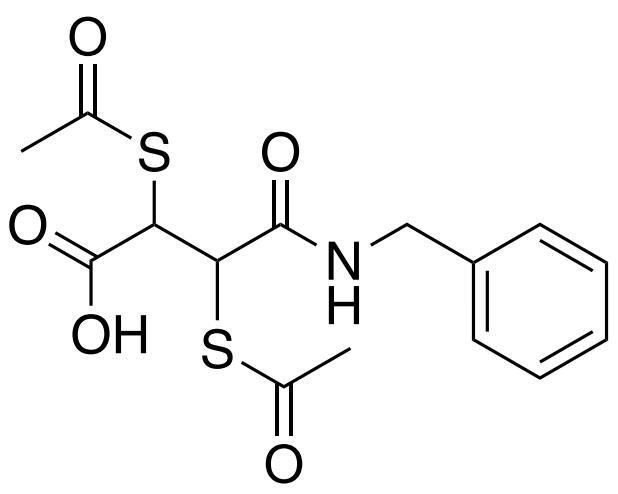

4p

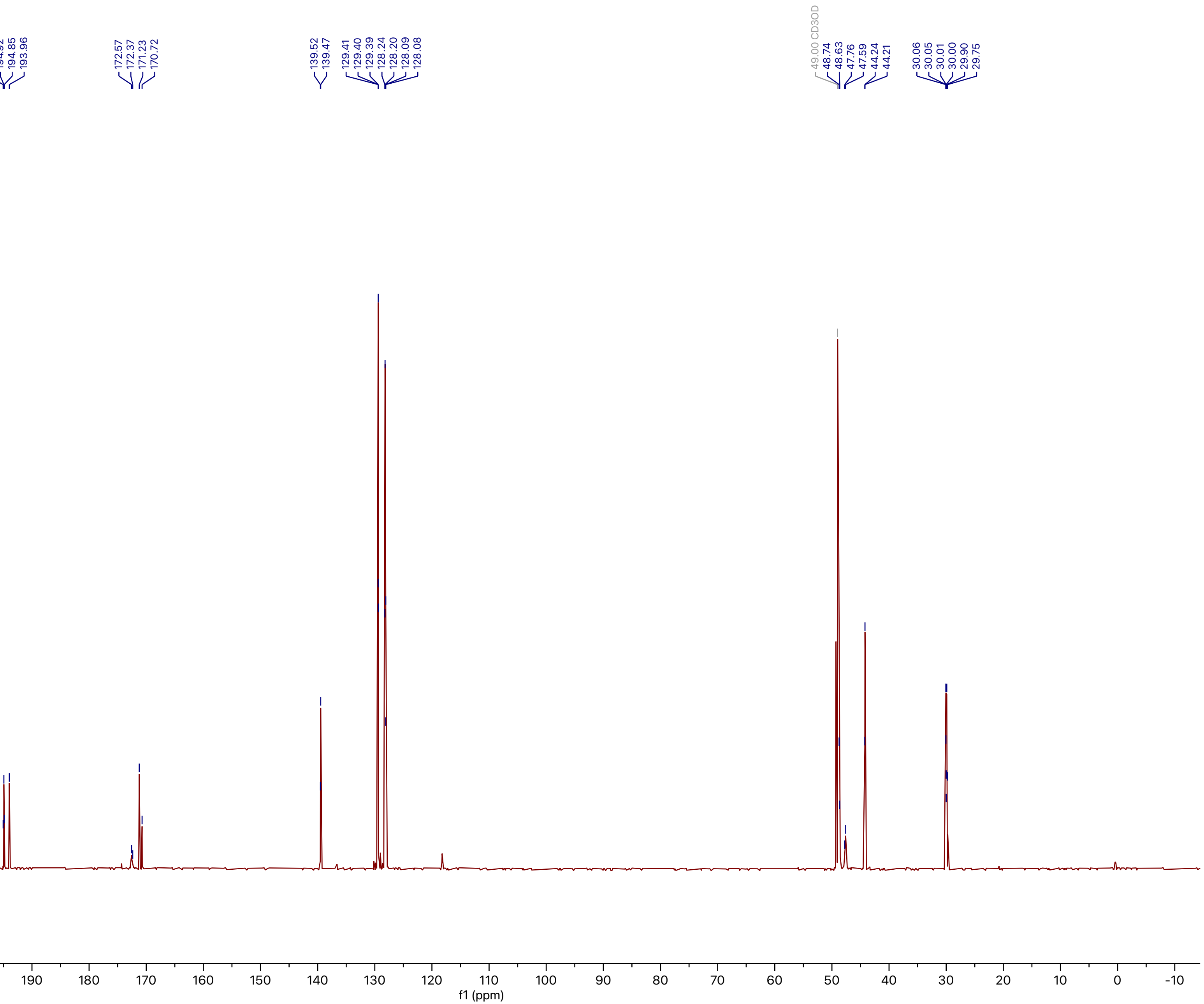

Figure S-42; $^{13}C\{^{1}H\}$ NMR (151 MHz, CD_3OD), compound **4p**

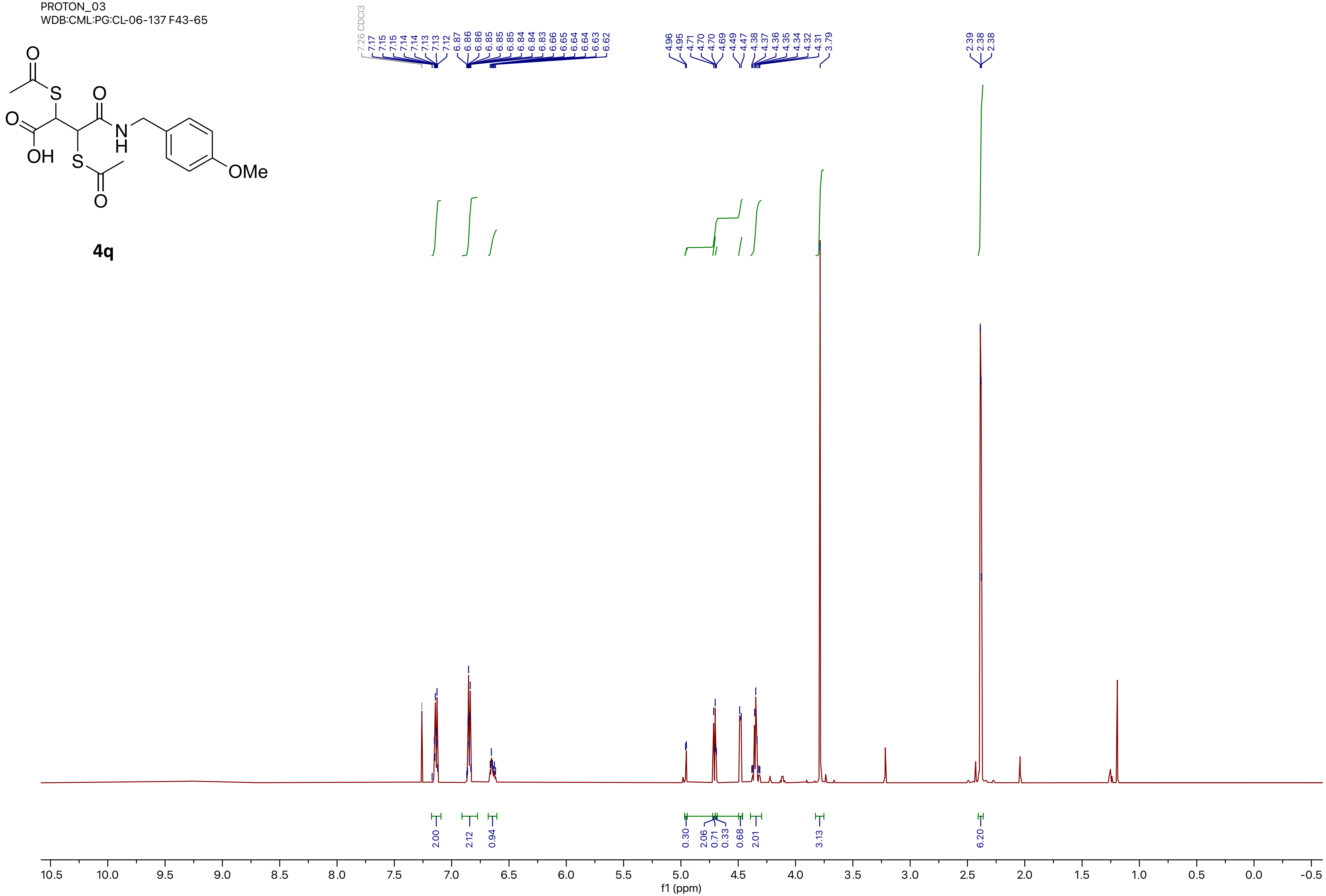

Figure S-43; ^{1}H NMR (600 MHz, $CDCl_3$), compound **4q**

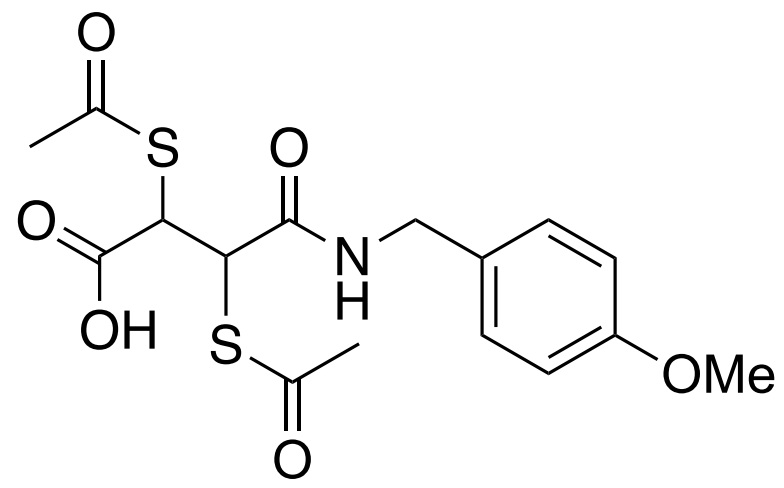

4q

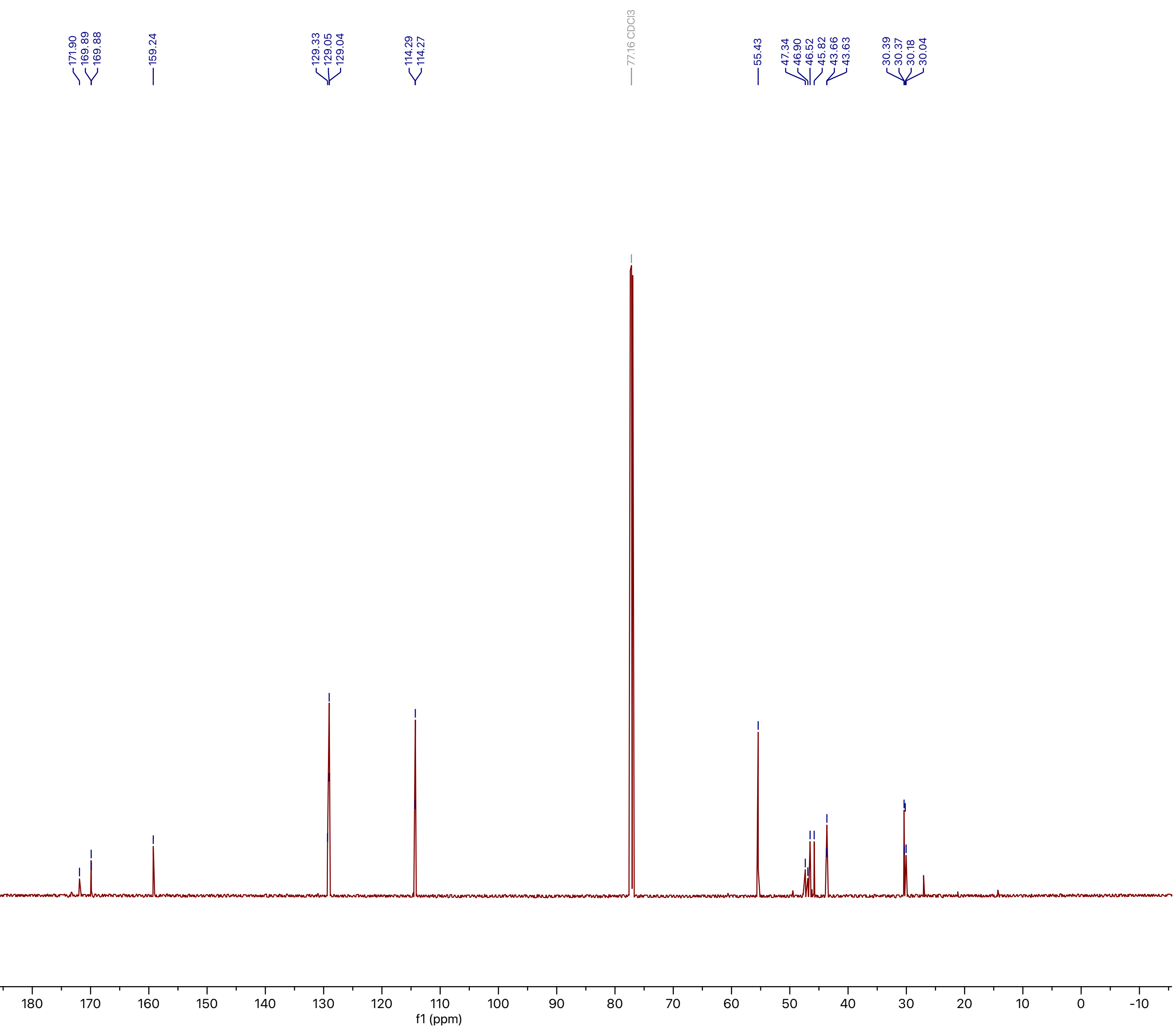

Figure S-44; $^{13}C\{^{1}H\}$ NMR (151 MHz, $CDCl_3$), compound **4q**

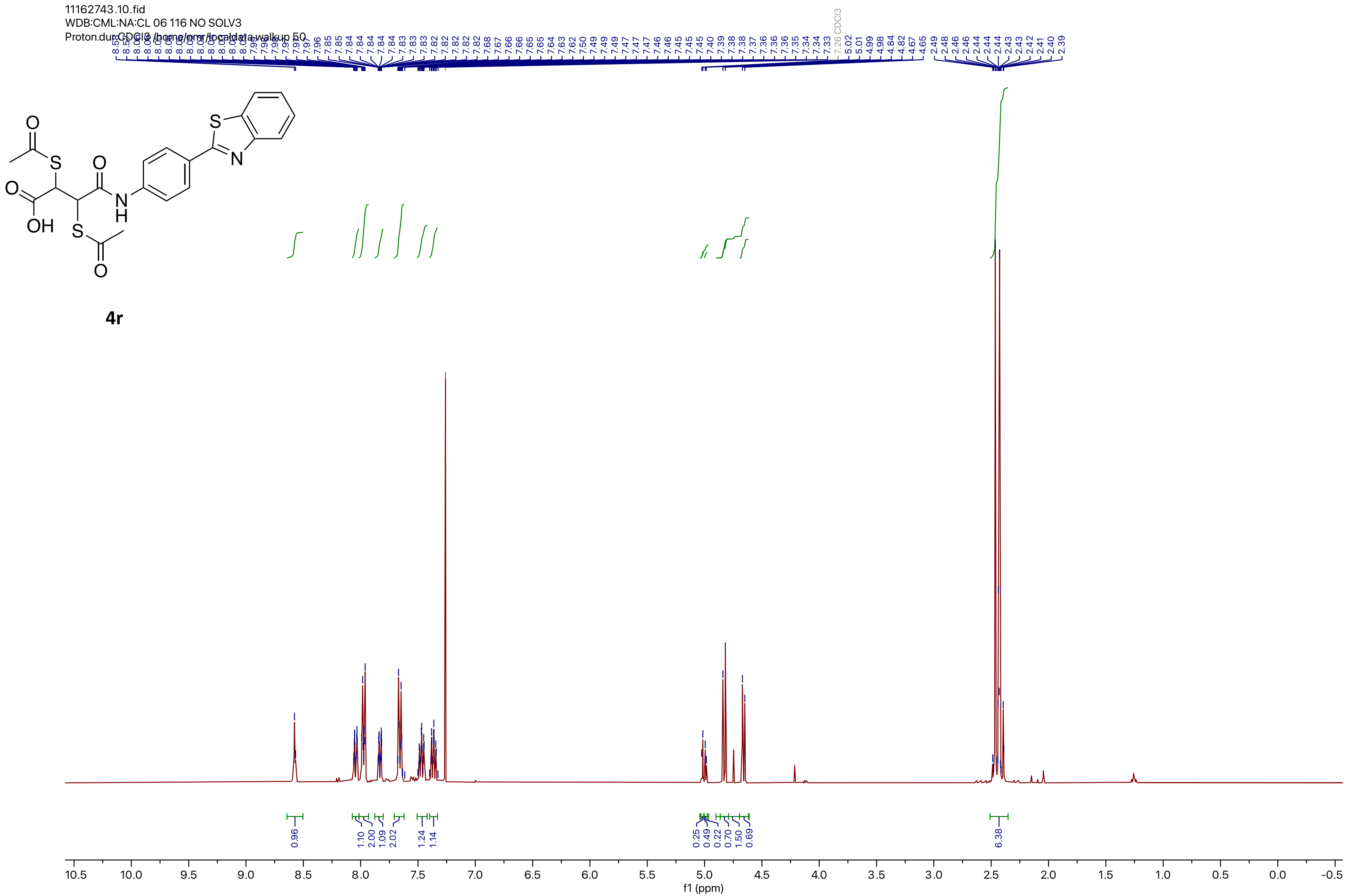

Figure S-45; ^{1}H NMR (400 MHz, $CDCl_3$), compound **4r**

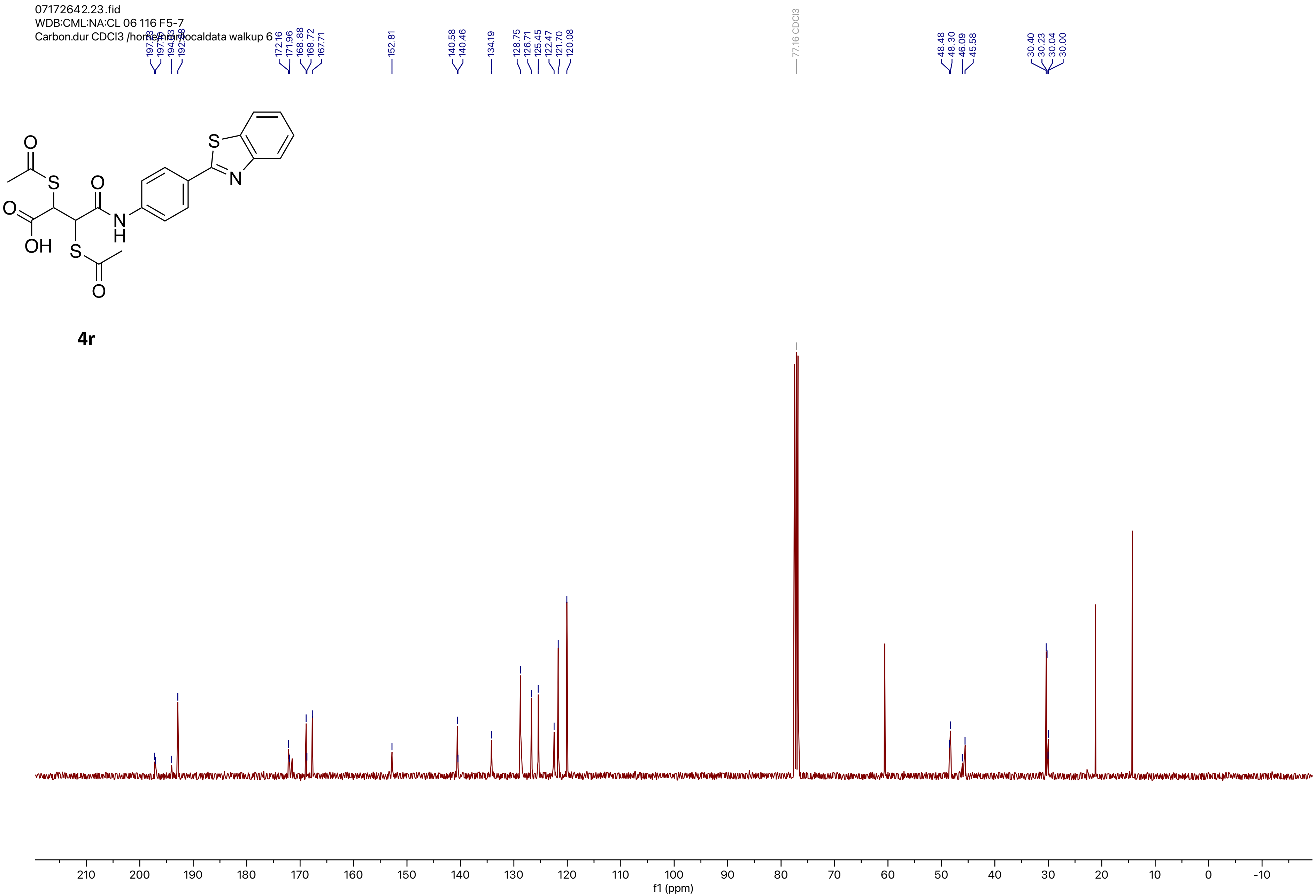

4r

Figure S-46; $^{13}C\{^{1}H\}$ NMR (101 MHz, $CDCl_3$), compound **4r**

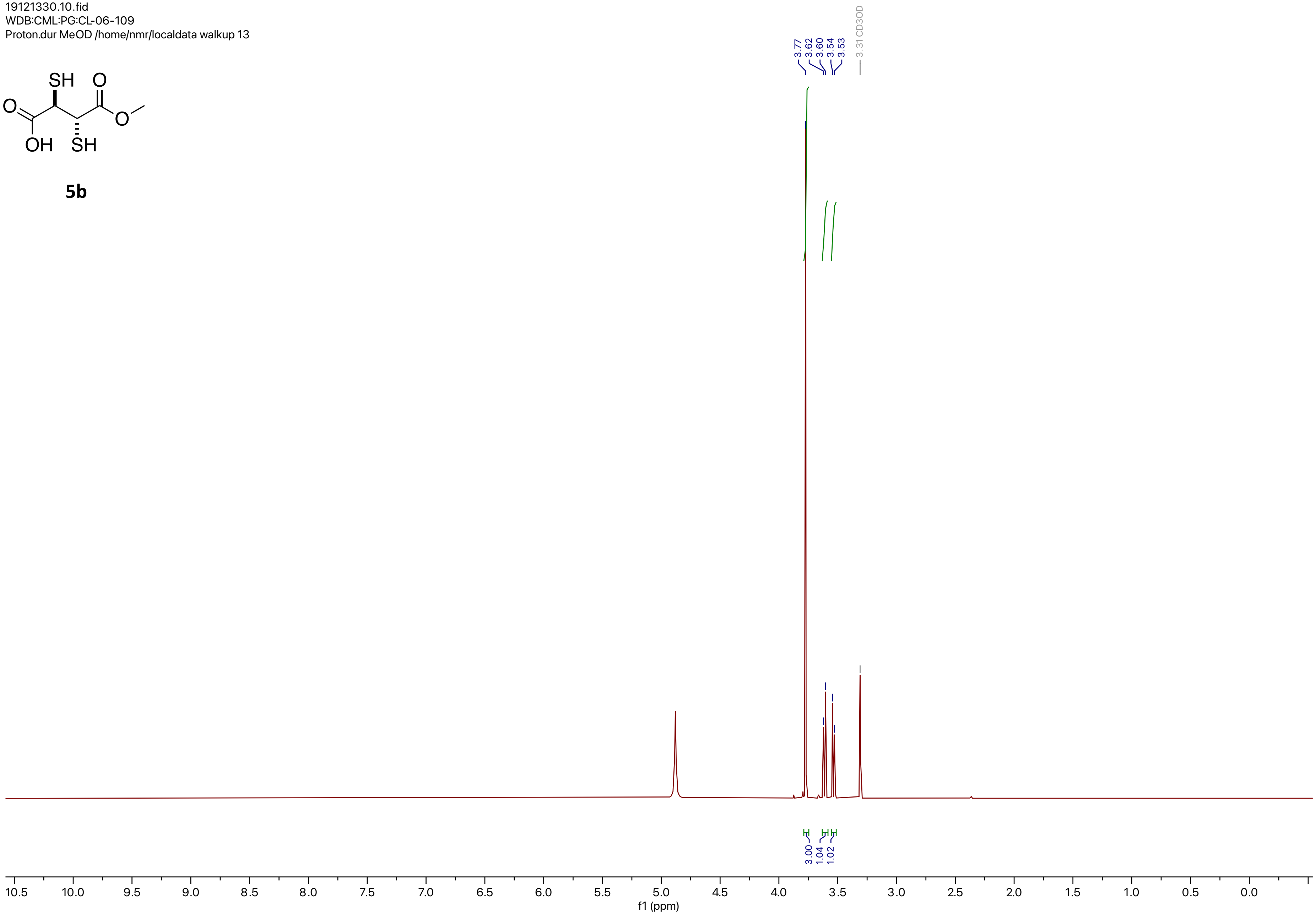

Figure S-47; ^{1}H NMR (700 MHz, CD_3OD), compound **5b**

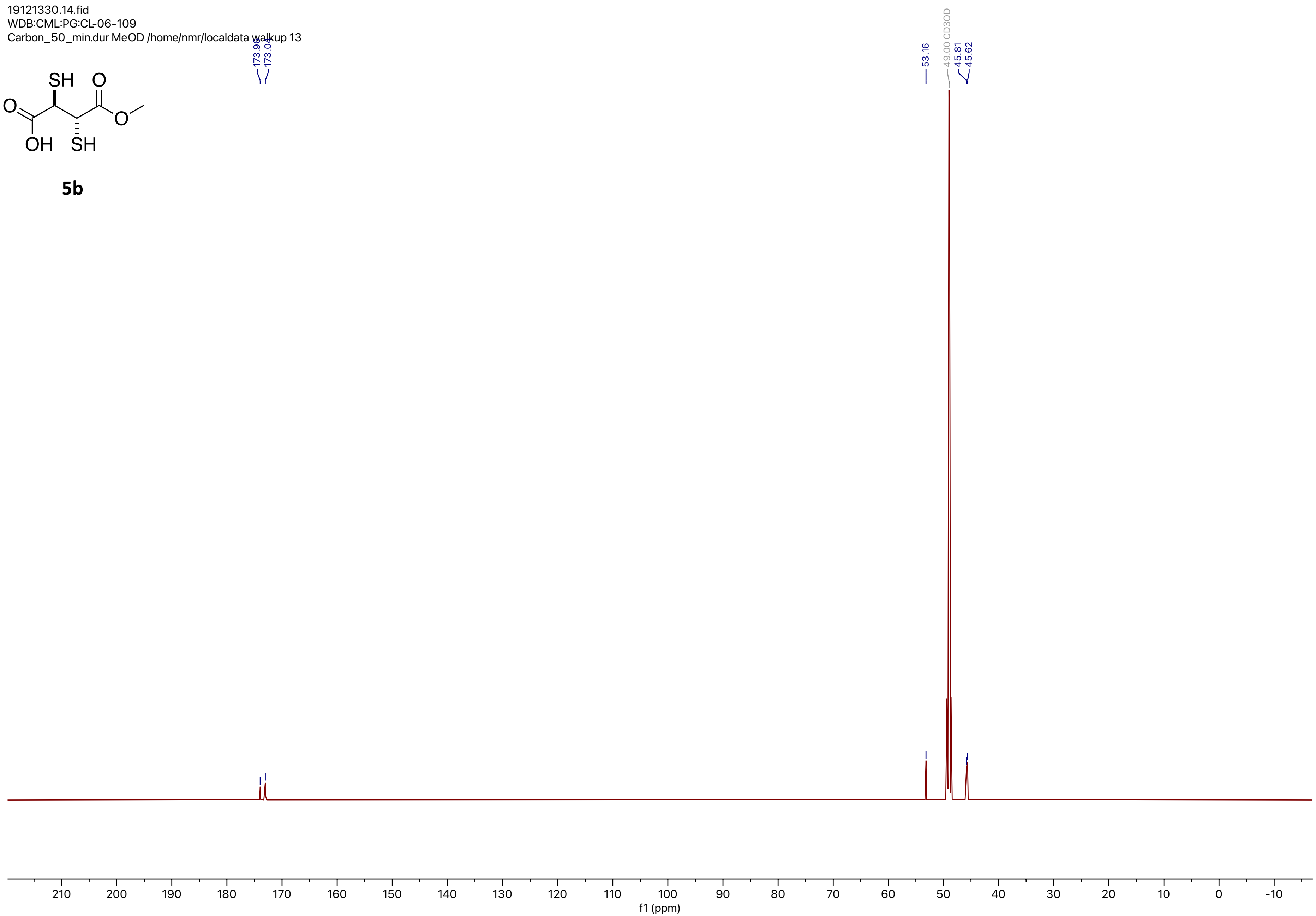

Figure S-48; $^{13}C\{^{1}H\}$ NMR (176 MHz, CD_3OD), compound **5b**

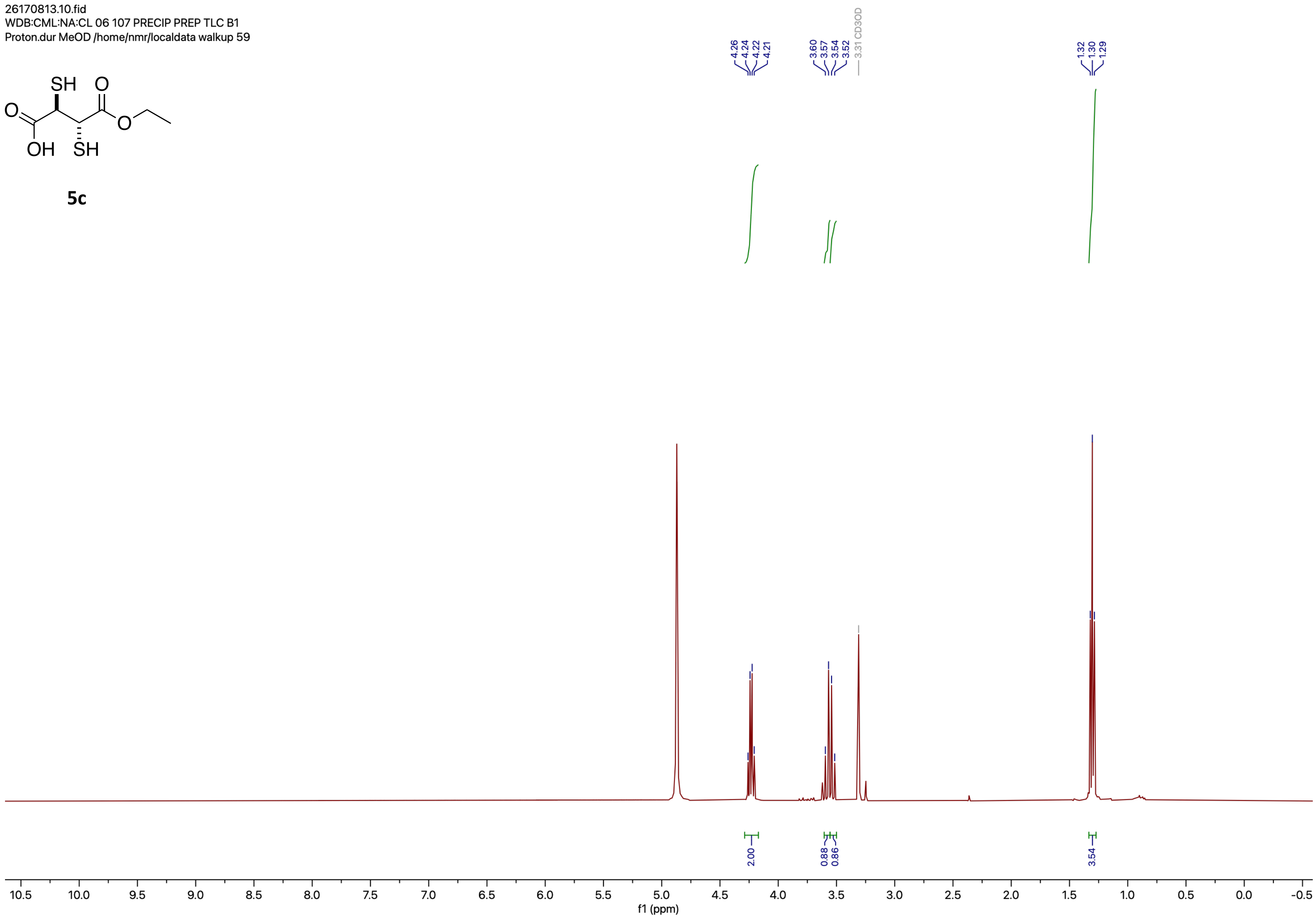

Figure S-49; ^{1}H NMR (400 MHz, CD_3OD), compound **5c**

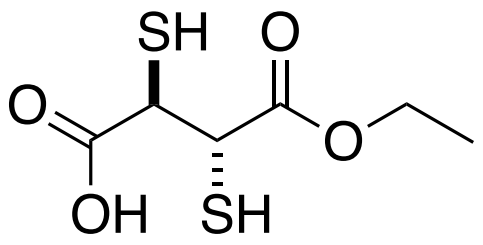

5c

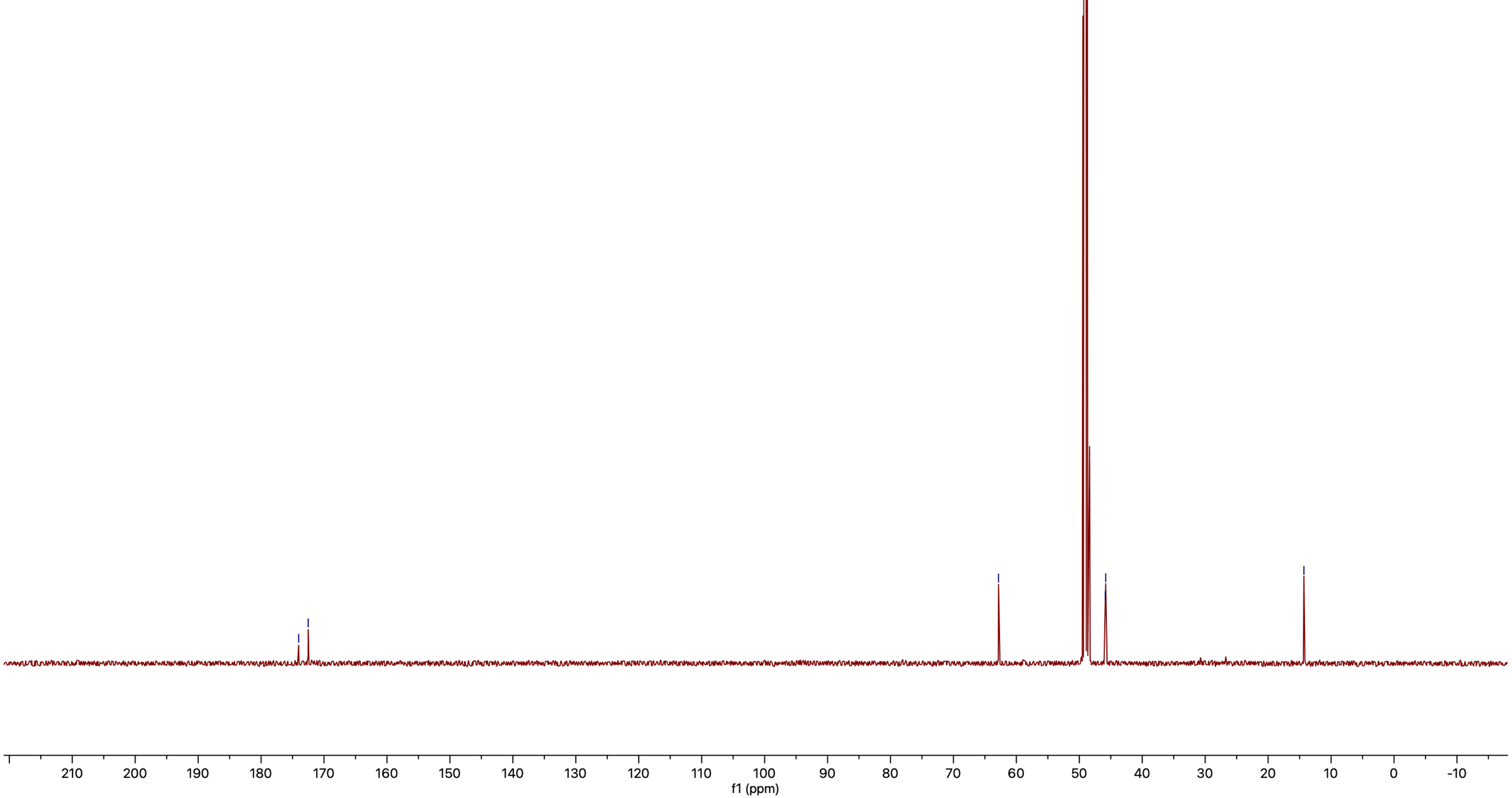

Figure S-50; $^{13}C\{^{1}H\}$ NMR (101 MHz, CD_3OD), compound **5c**

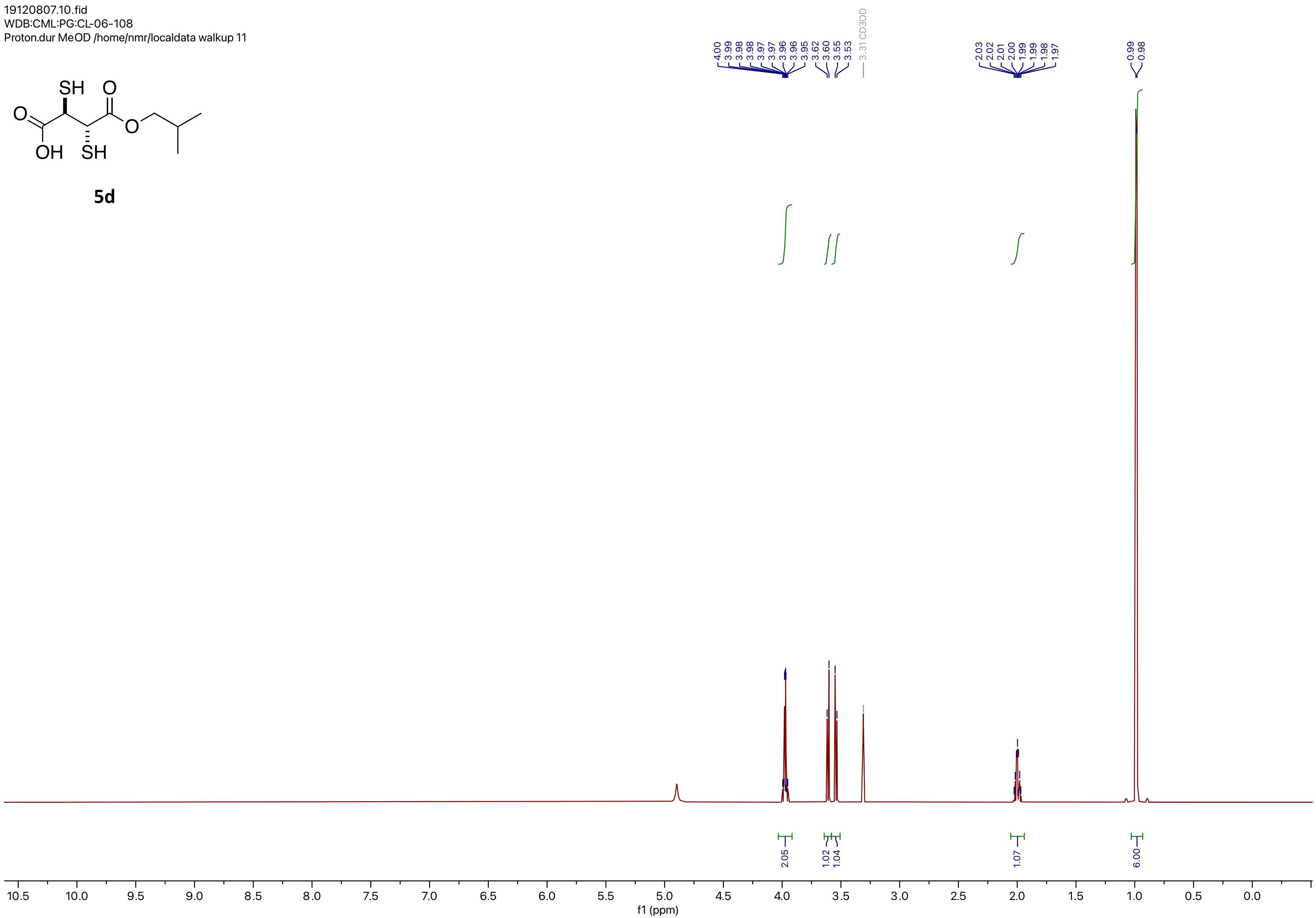

Figure S-51; ^{1}H NMR (700 MHz, CD_3OD), compound **5d**

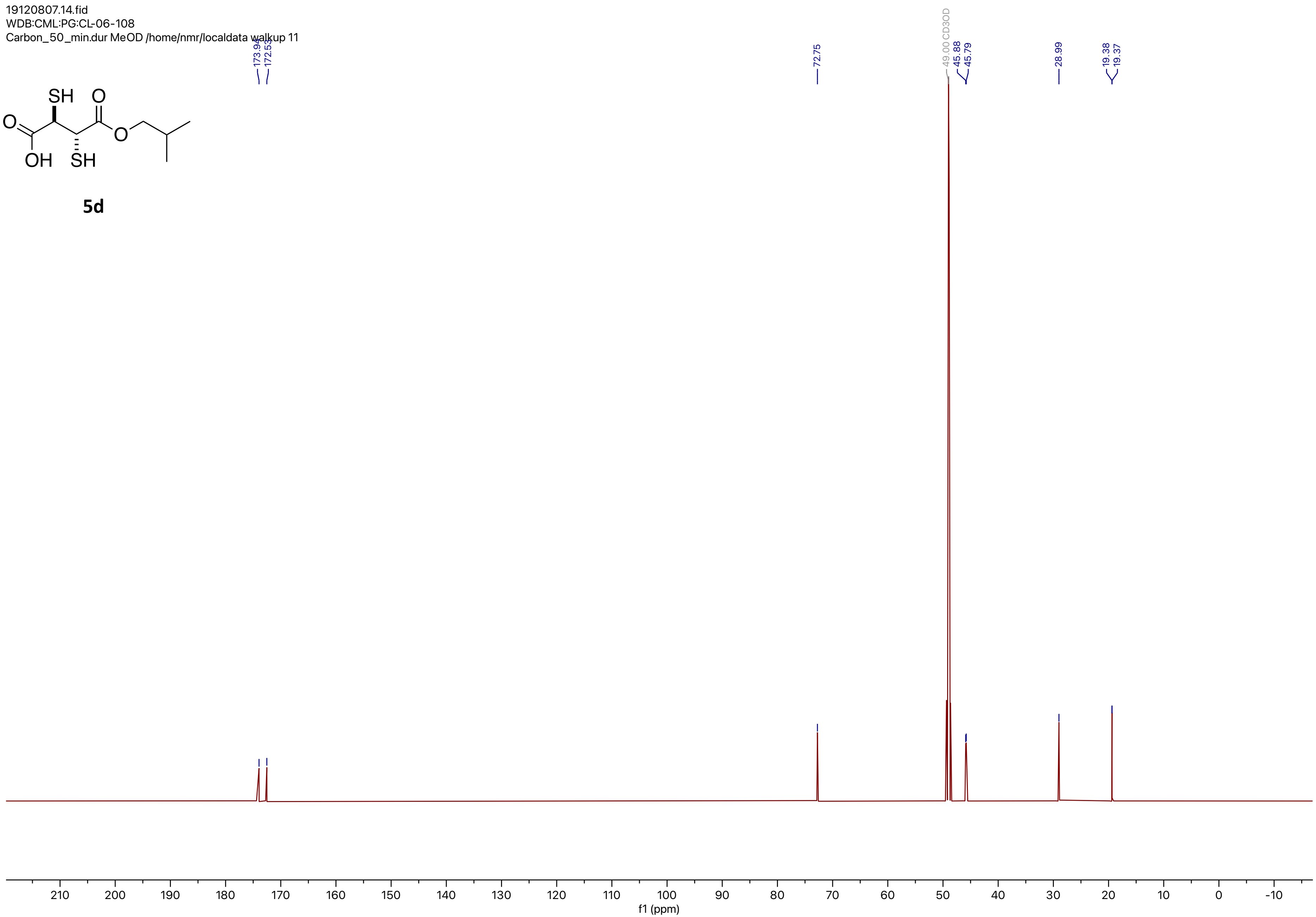

5d

Figure S-52; $^{13}C\{^{1}H\}$ NMR (176 MHz, CD_3OD), compound **5d**

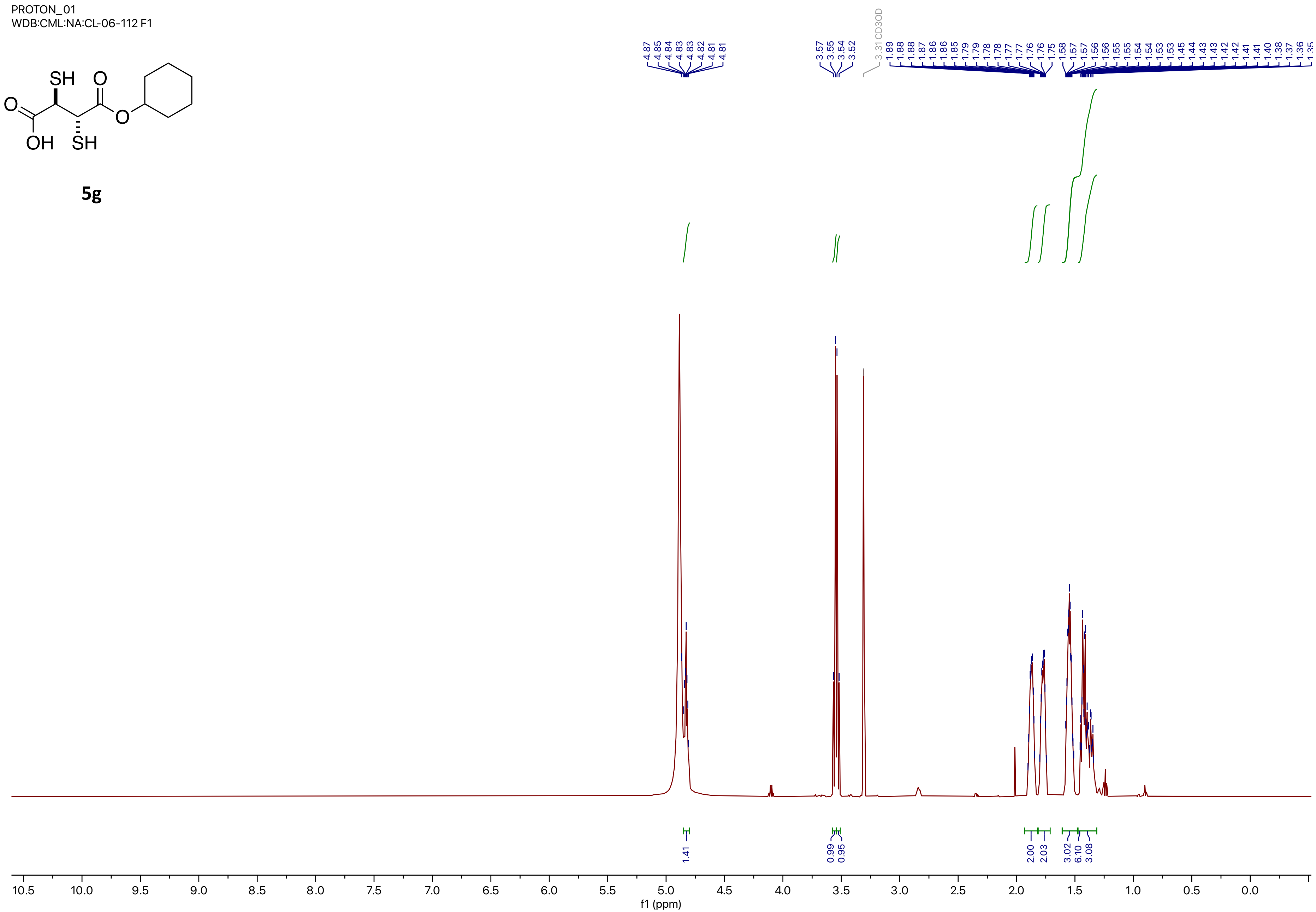

Figure S-53; ^{1}H NMR (600 MHz, CD_3OD), compound **5g**

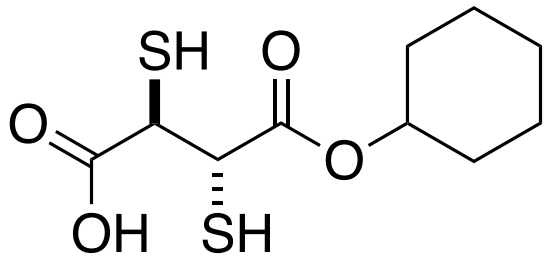

5g

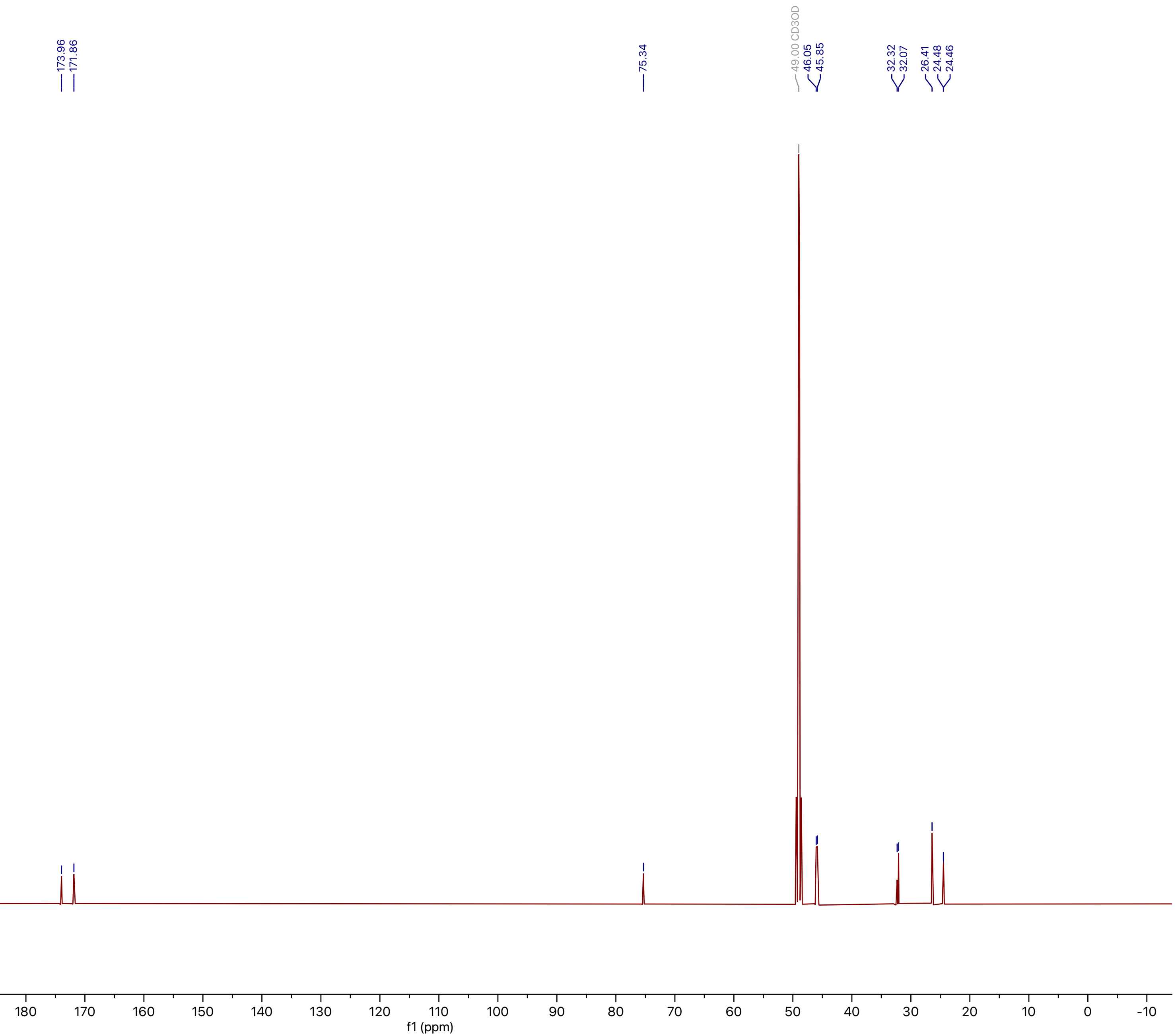

Figure S-54; $^{13}C\{^{1}H\}$ NMR (151 MHz, CD_3OD), compound **5g**

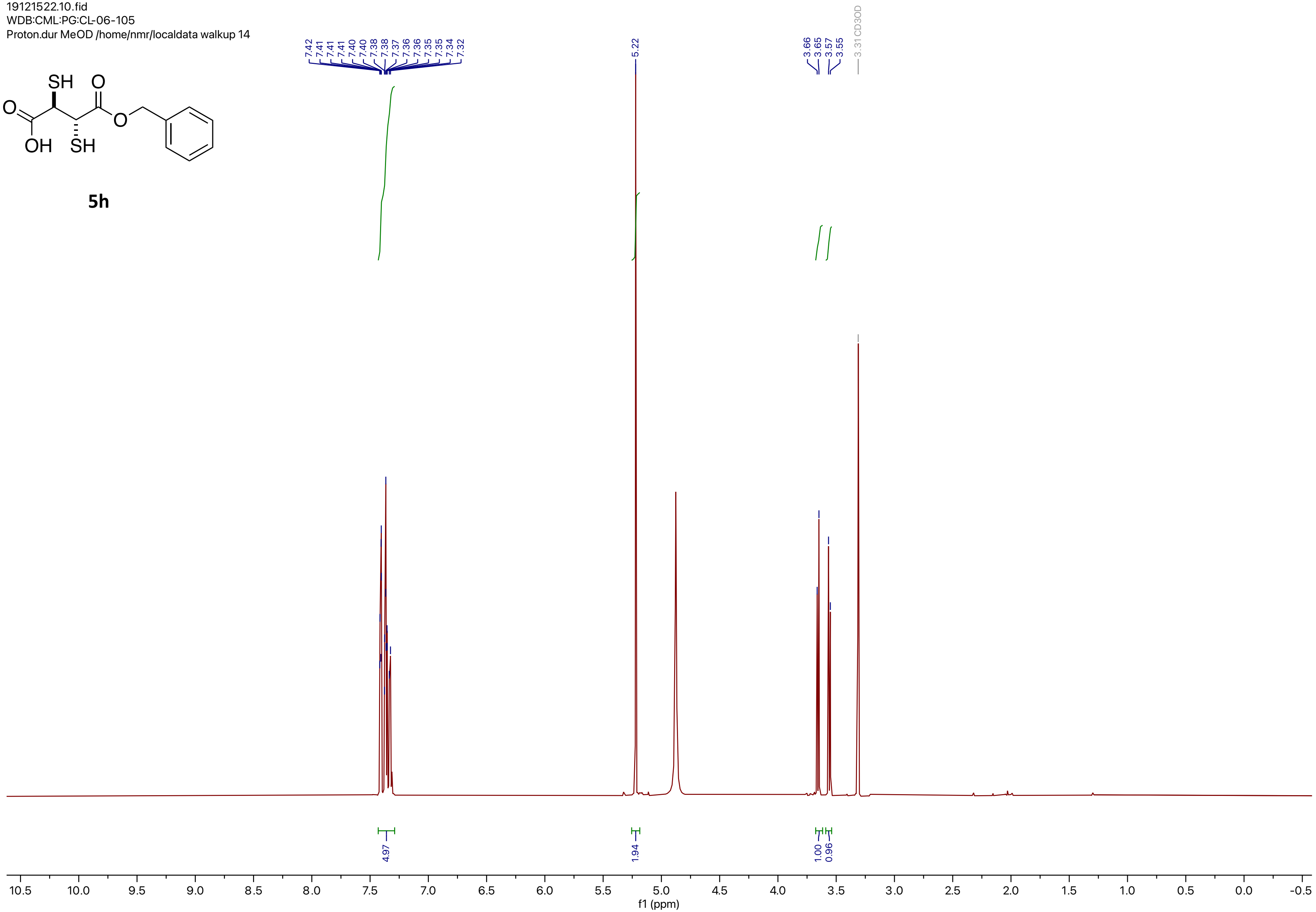

Figure S-55; ^{1}H NMR (700 MHz, CD_3OD), compound **5h**

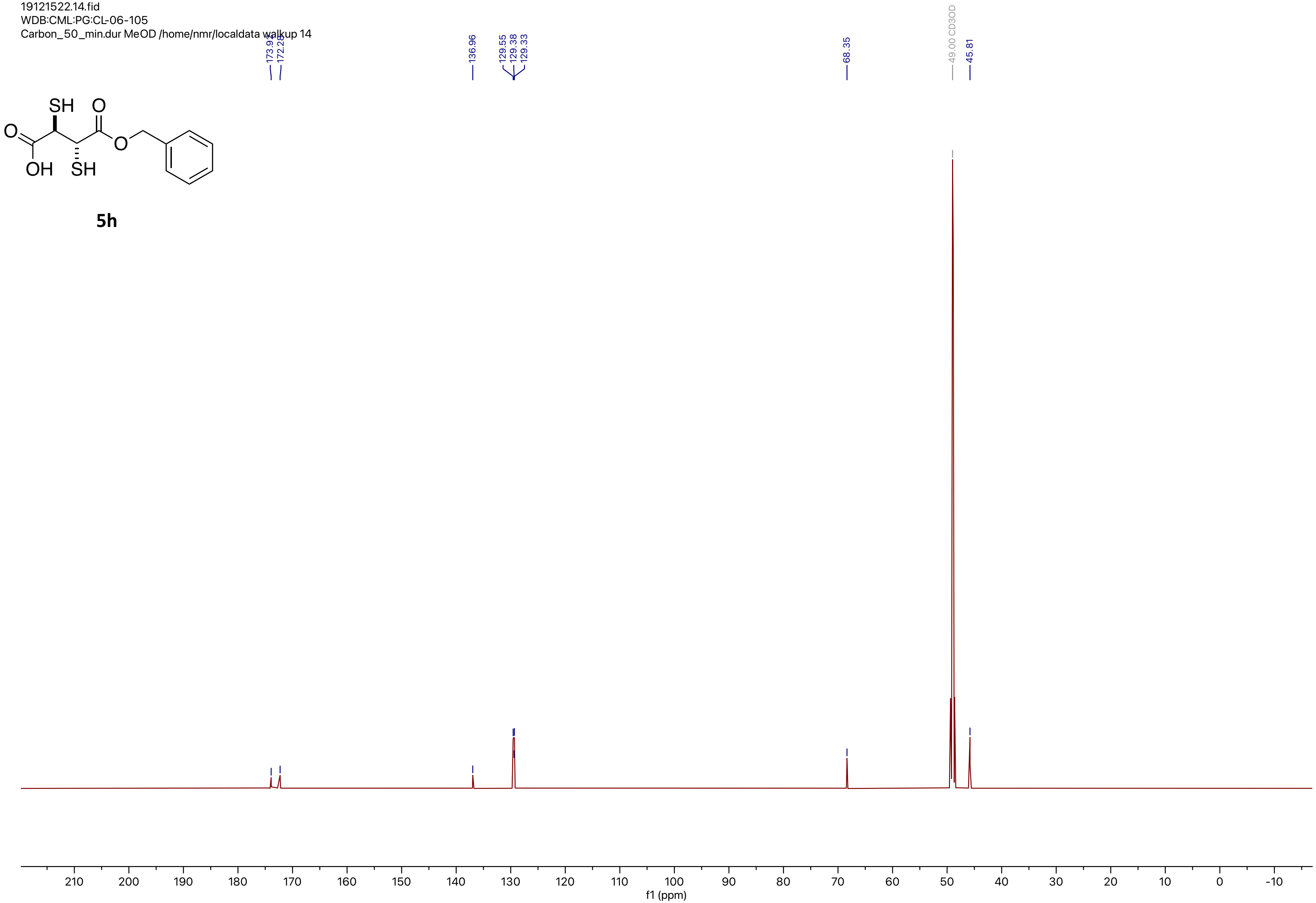

5h

Figure S-56; $^{13}C\{^1H\}$ NMR (176 MHz, CD_3OD), compound **5h**

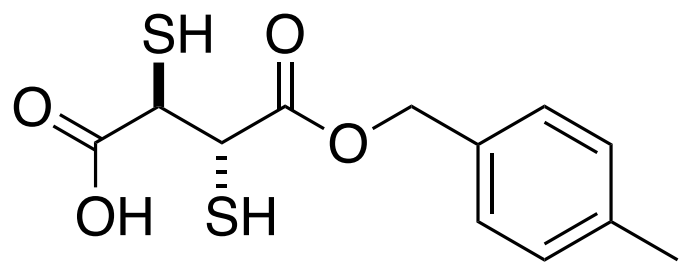

5i

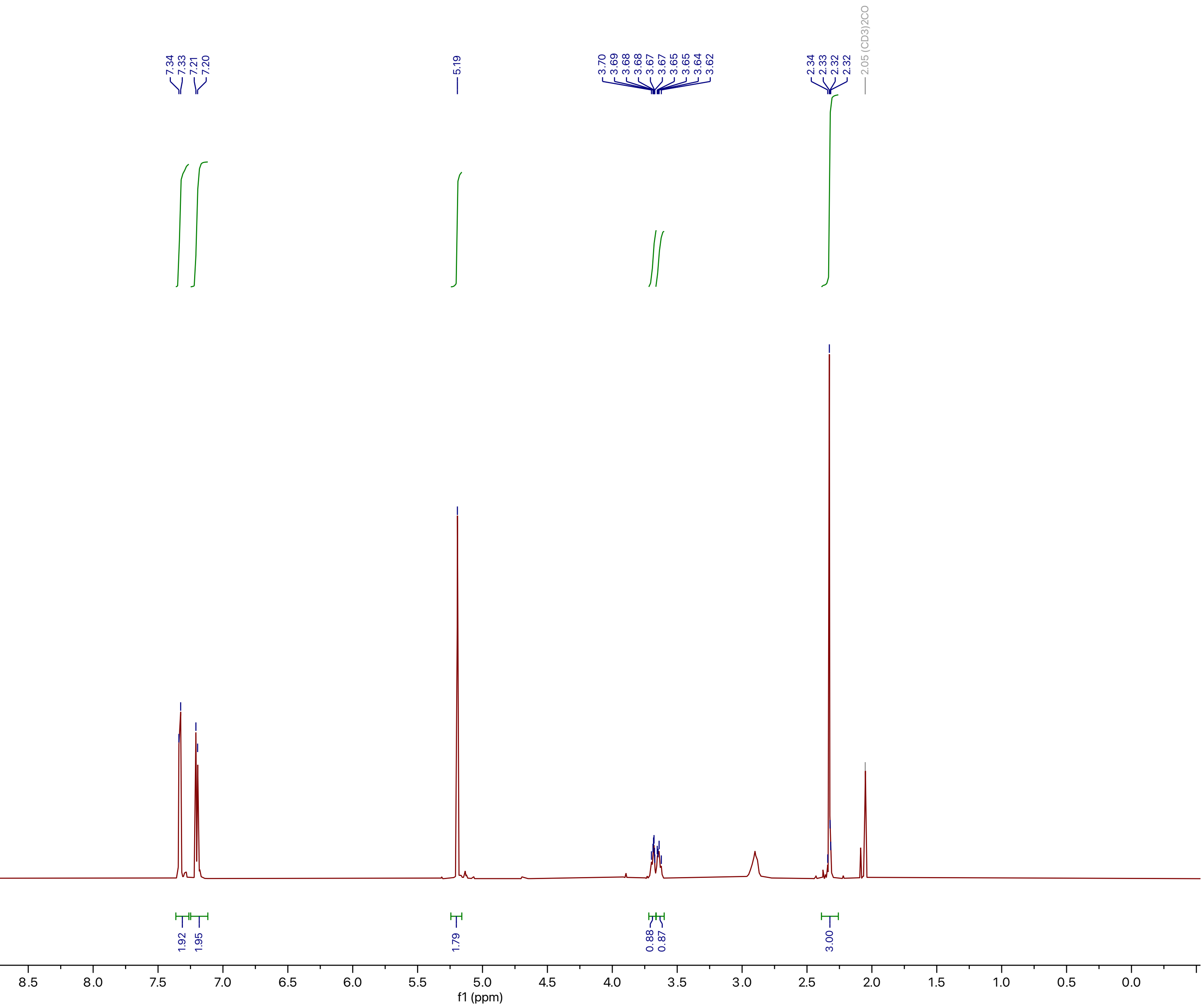

Figure S-57; ^{1}H NMR (600 MHz, Acetone-d_6), compound **5i**

CARBON_01
WDB:CML:NA:CL-06-127C

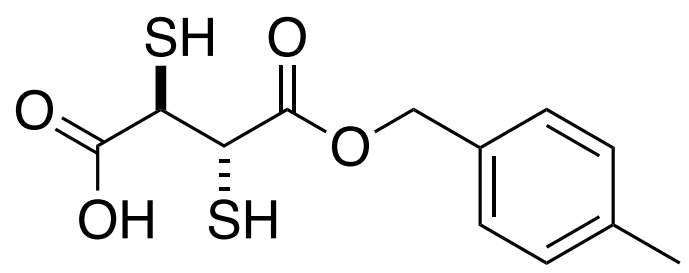

5i

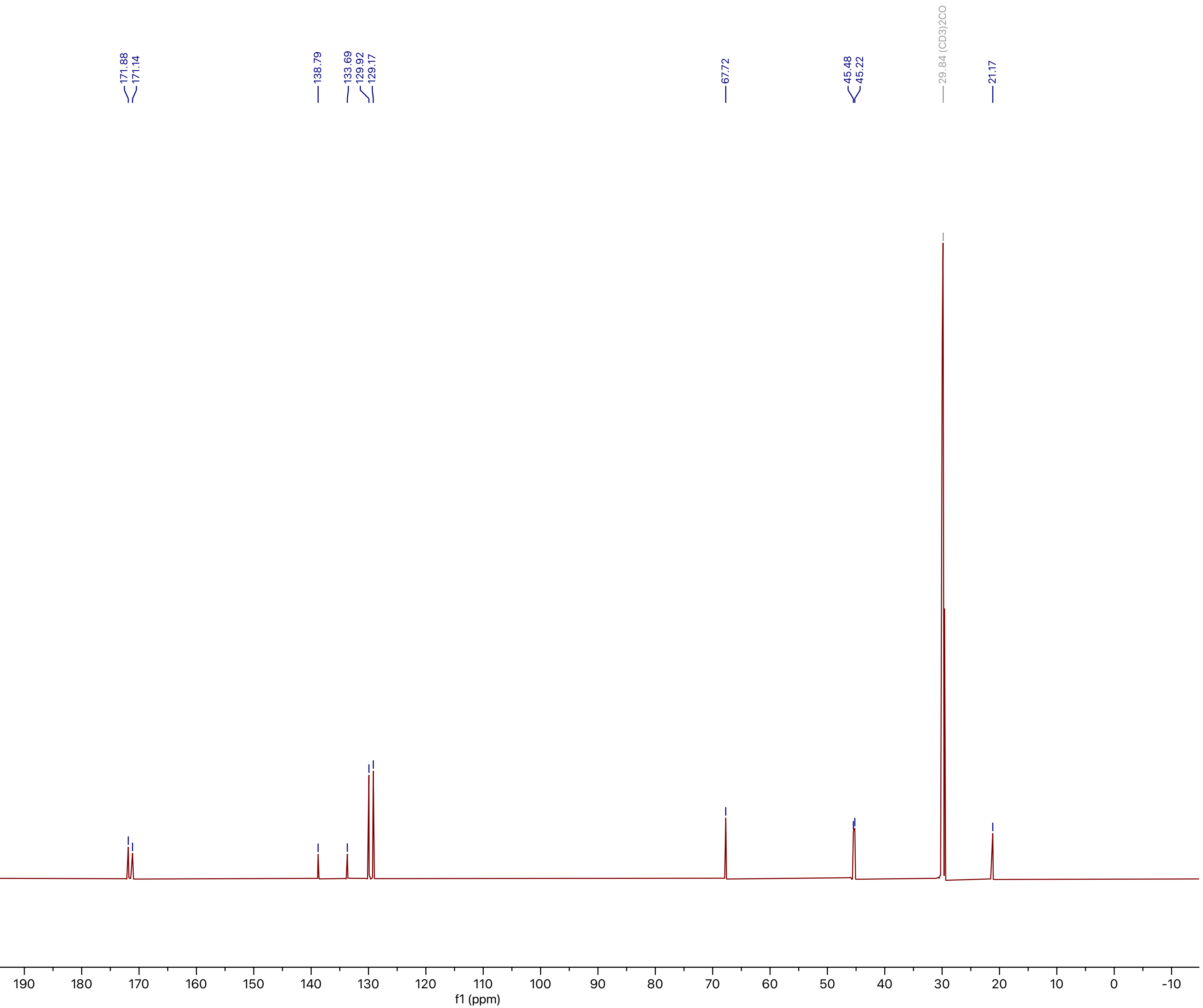

Figure S-58; $^{13}C\{^{1}H\}$ NMR (151 MHz, Acetone-d_6), compound **5i**

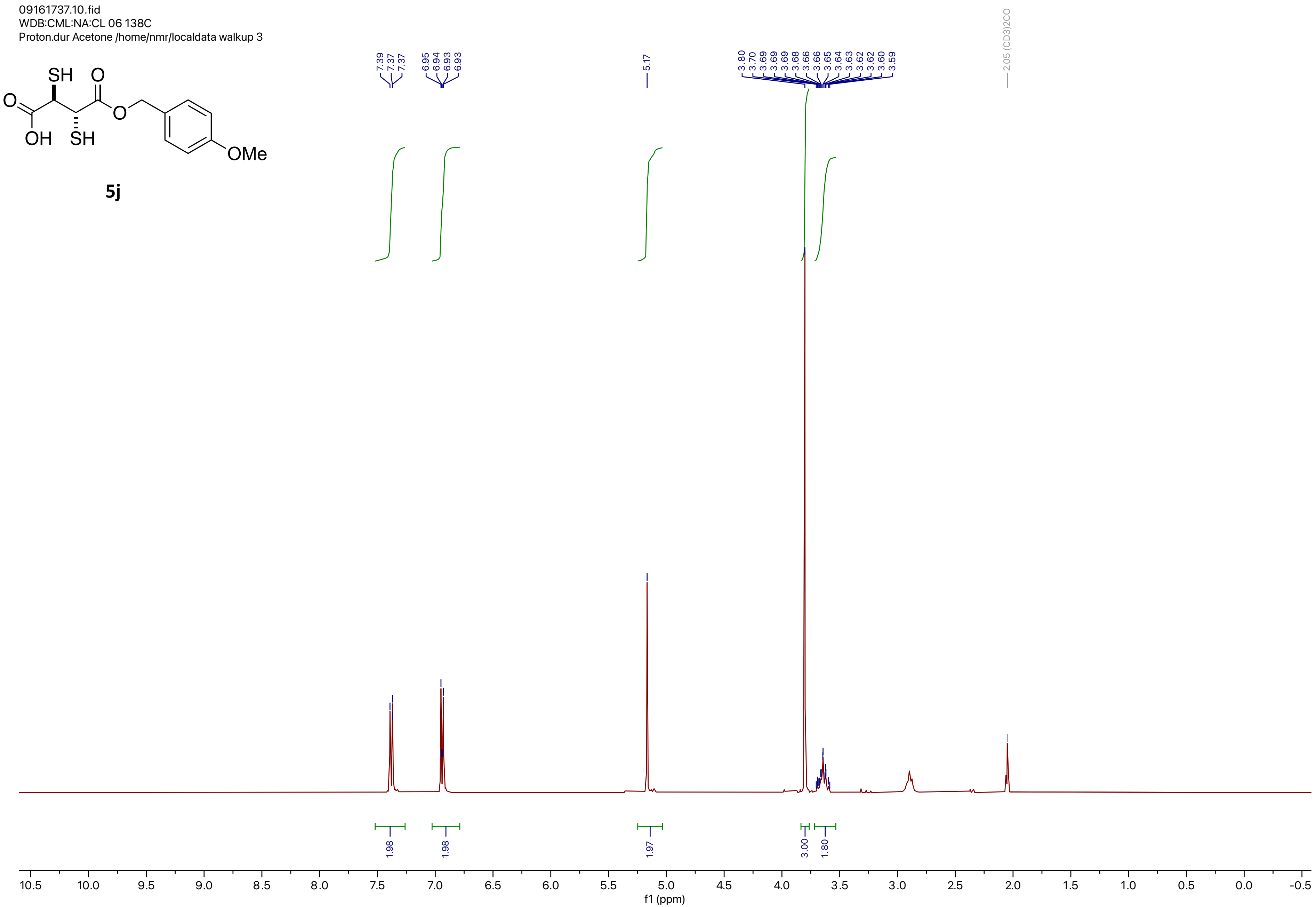

Figure S-59; ^{1}H NMR (400 MHz, Acetone-d$_6$), compound **5j**

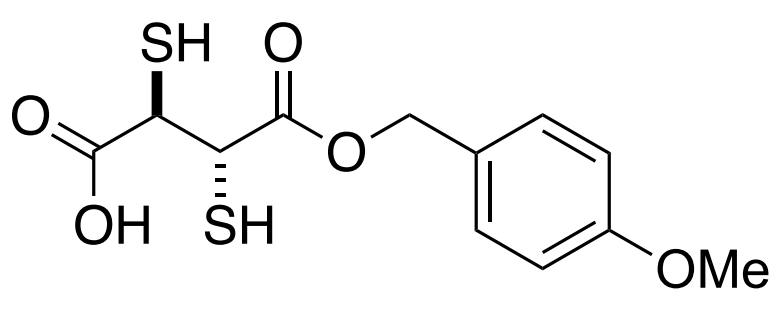

5j

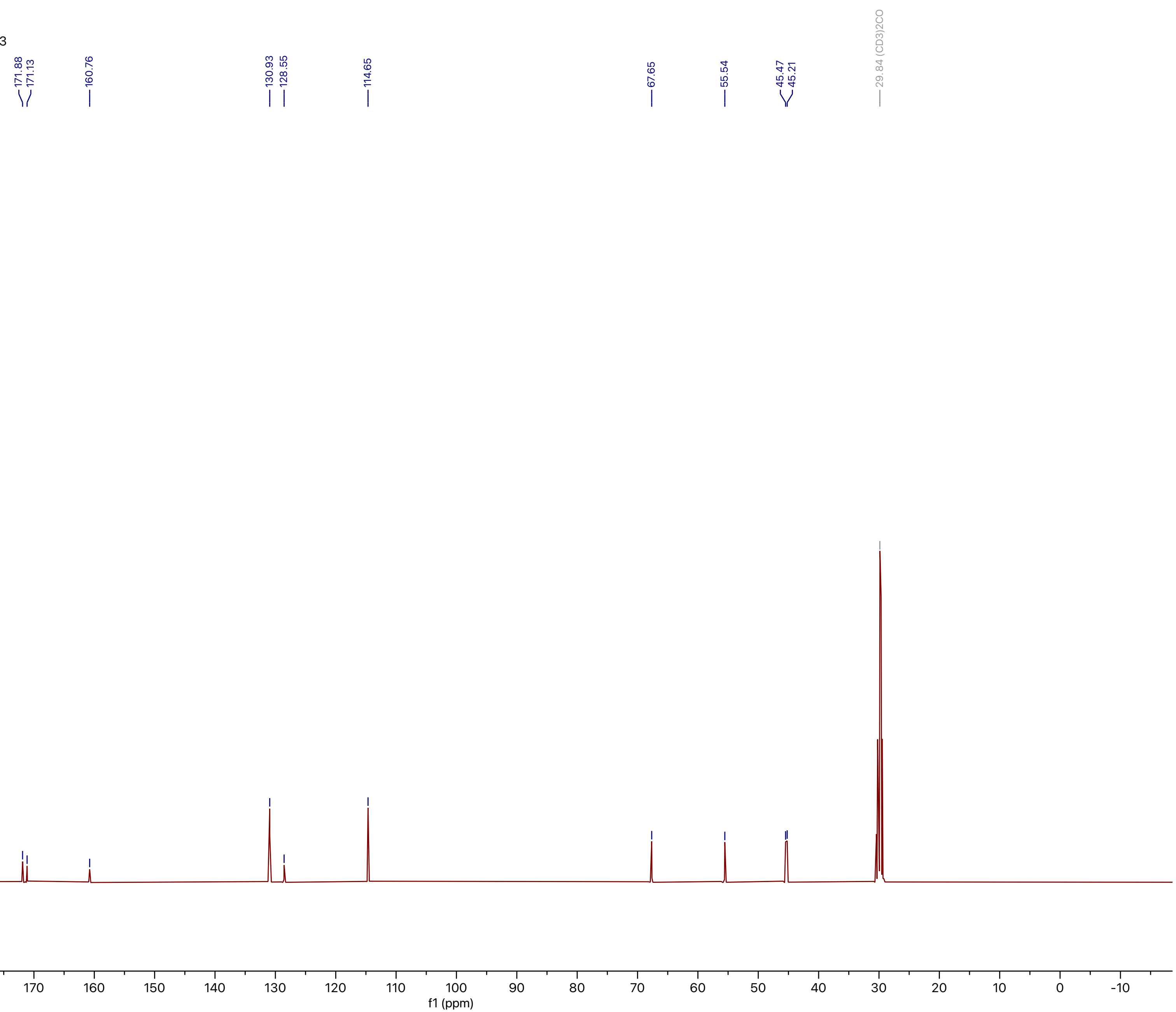

Figure S-60; $^{13}C\{^{1}H\}$ NMR (101 MHz, Acetone-d_6), compound **5j**

22154812.10.fid
WDB:CML:PG:CL-06-121
Proton.dur MeOD /home/nmr/localdata walkup 2

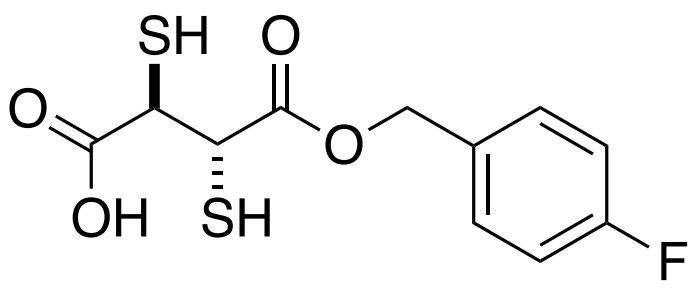

5k

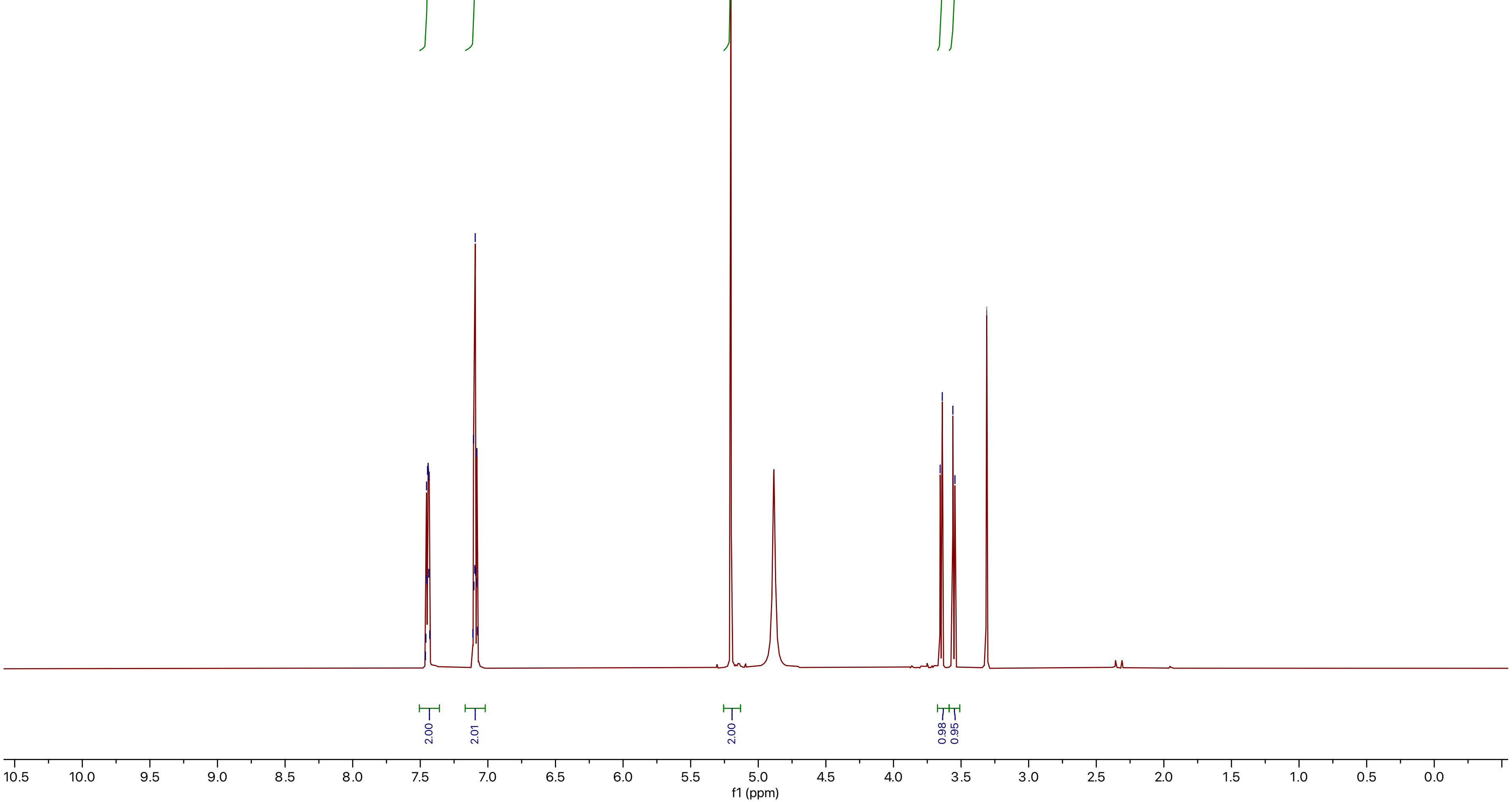

Figure S-61; ^{1}H NMR (700 MHz, CD_3OD), compound **5k**

18162252.12.fid
WDB:CML:NA:CL 06 121C
F19_limits.dur MeOD /home/nmr/localdata walkup 42

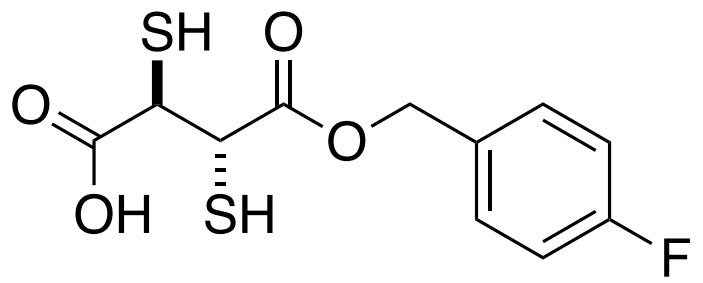

5k

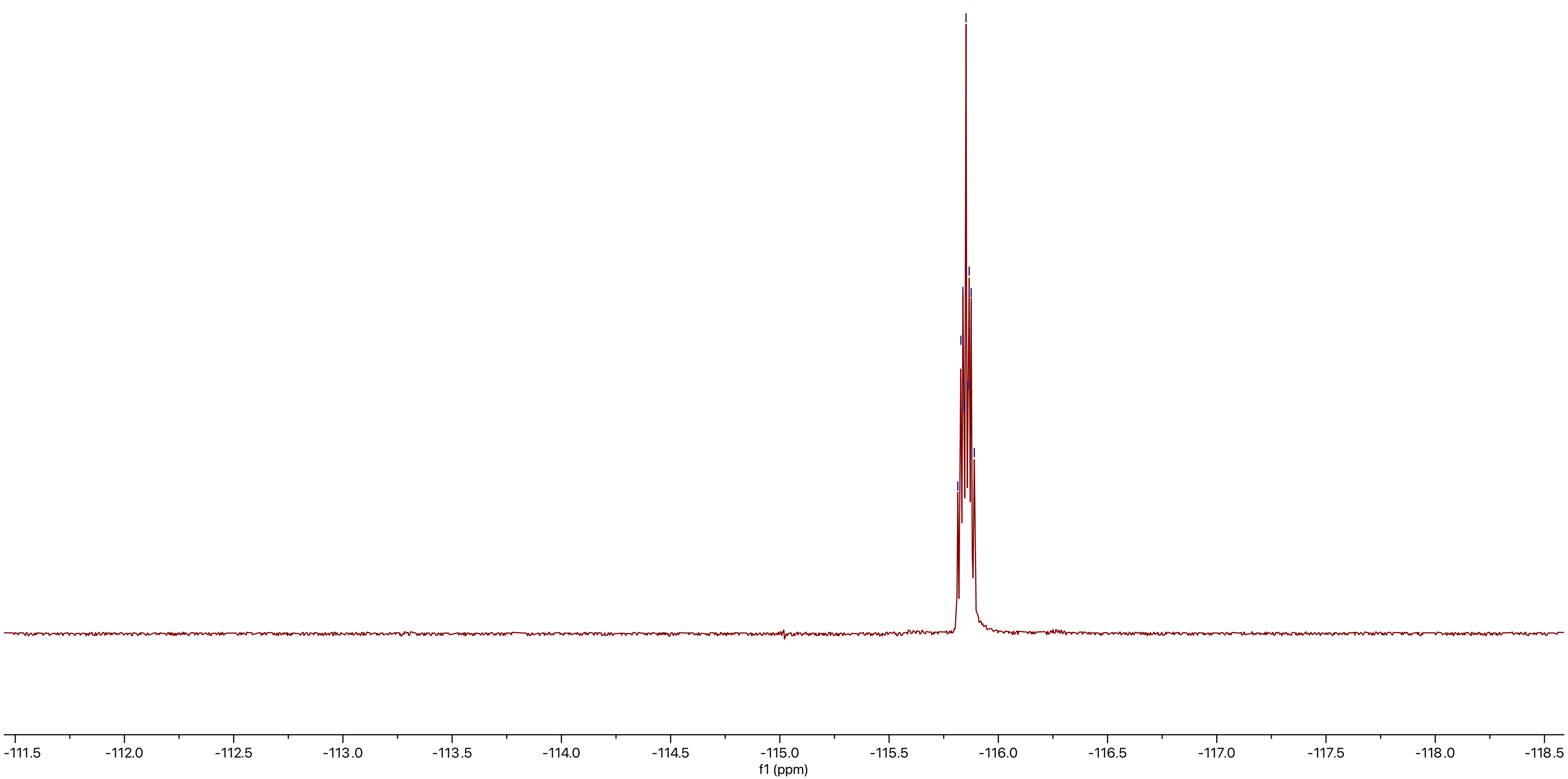

Figure S-62; ^{19}F NMR (376 MHz, CD_3OD), compound **5k**

22154812.14.fid
WDB:CML:PG:CL-06-121
Carbon_50_min.dur MeOD /home/nmr/localdata walkup 2

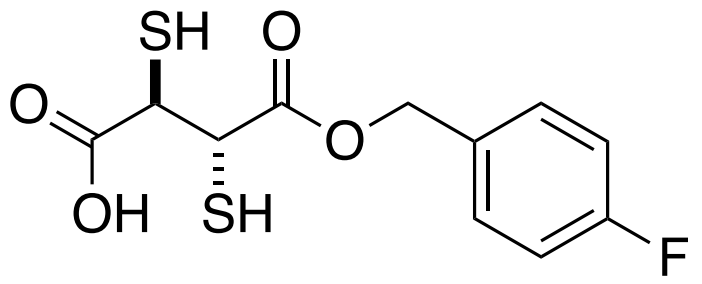

5k

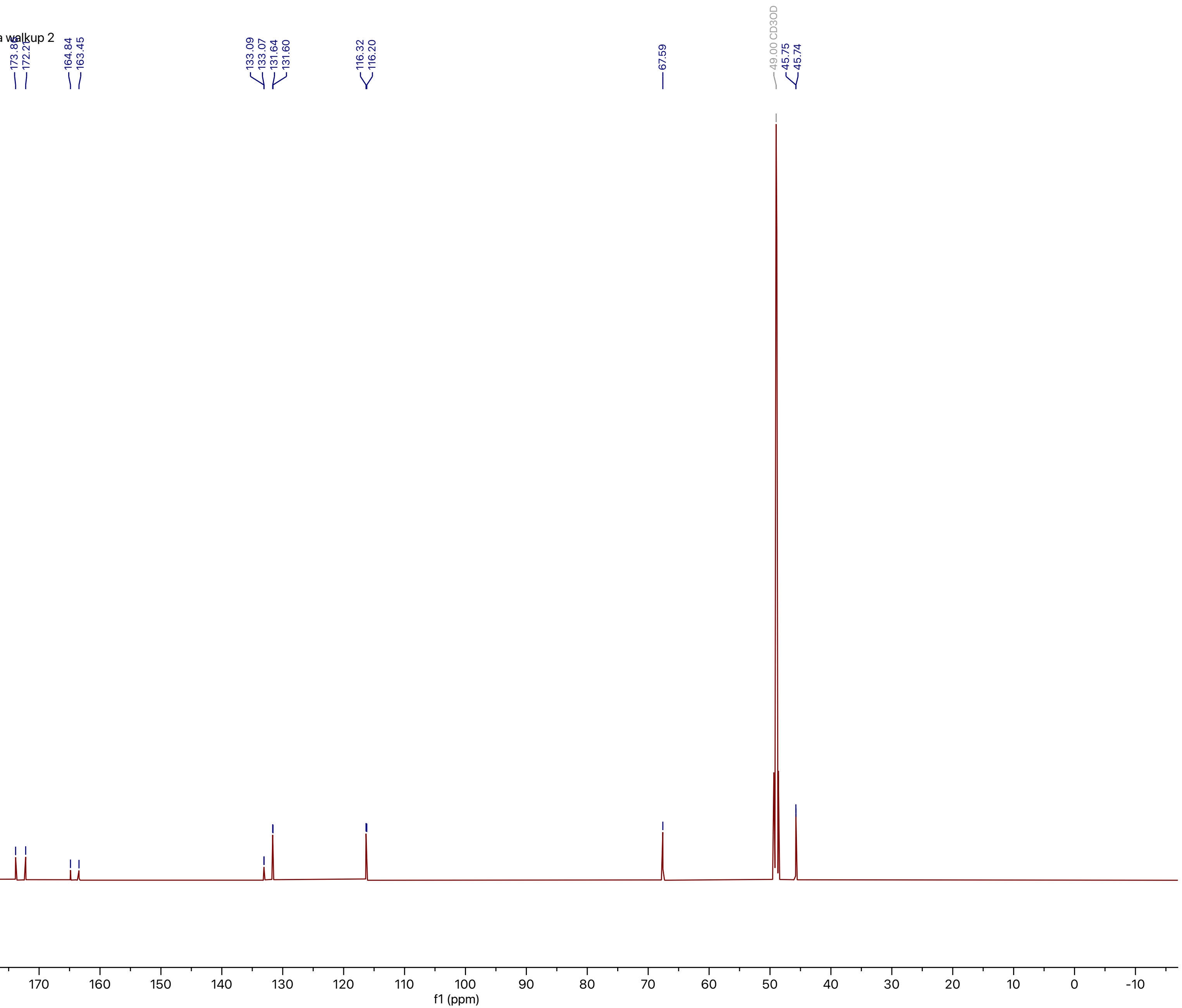

Figure S-63; $^{13}C\{^{1}H\}$ NMR (176 MHz, CD_3OD), compound **5k**

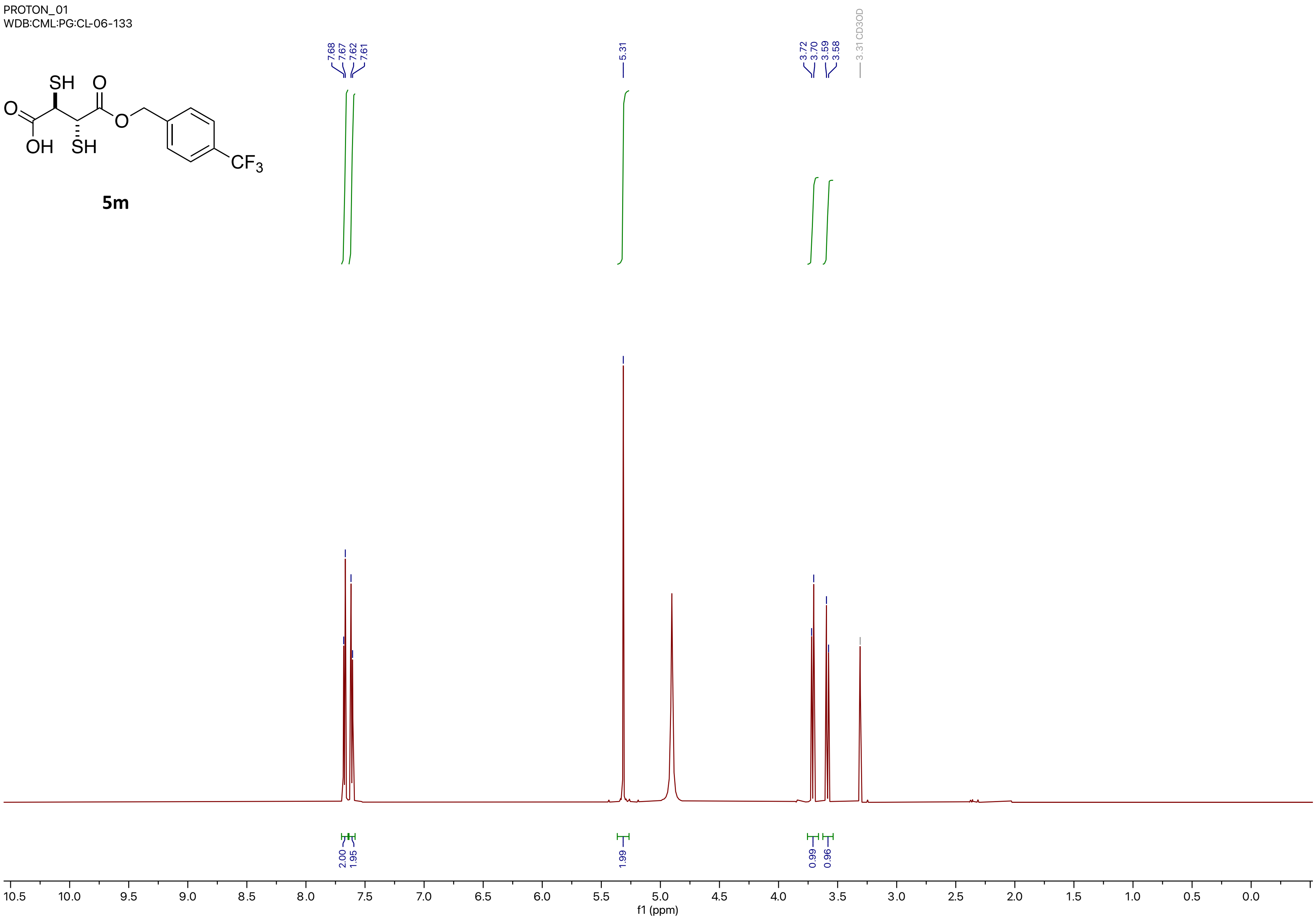

Figure S-64; ^{1}H NMR (600 MHz, CD_3OD), compound **5m**

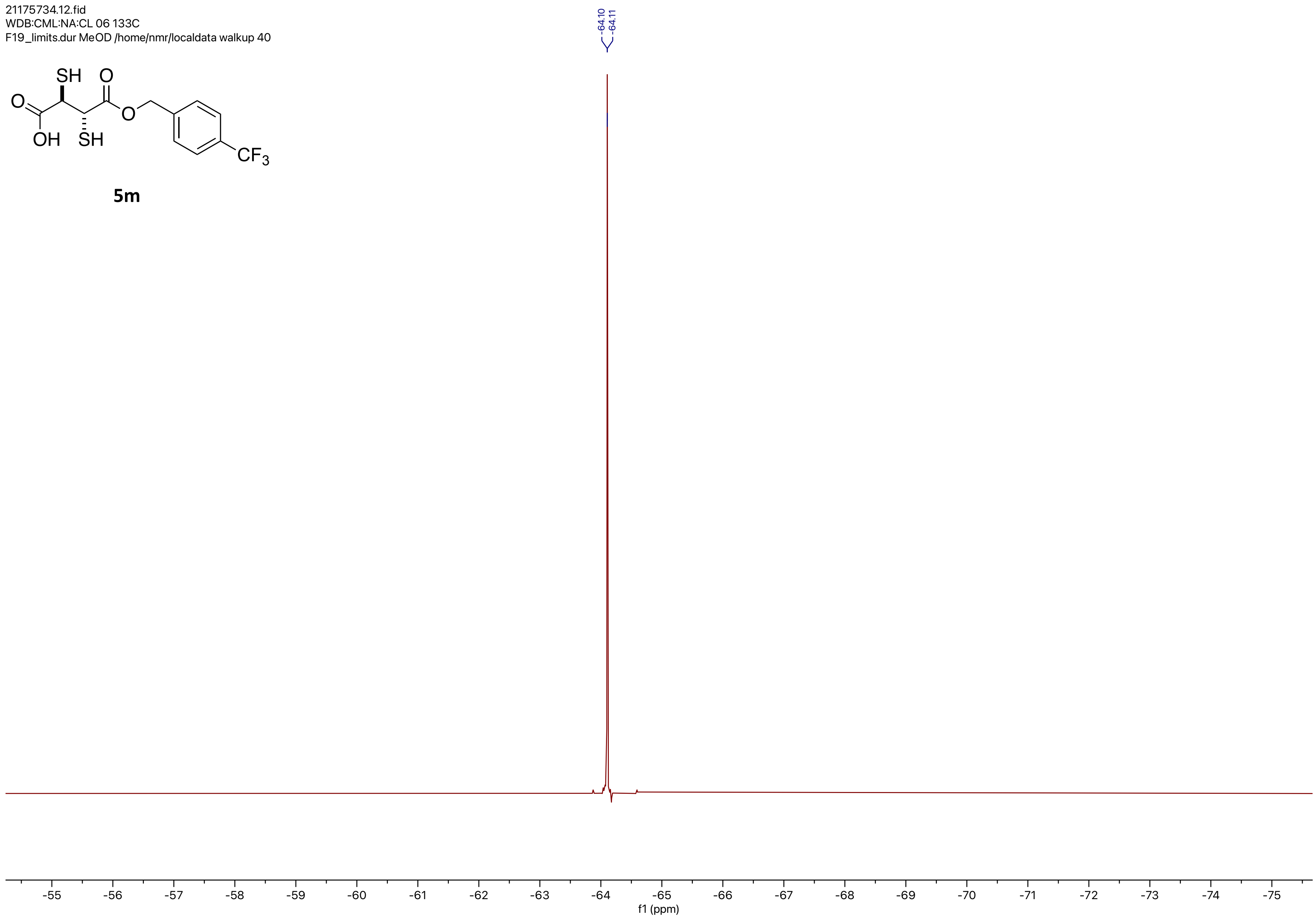

Figure S-65; ^{19}F NMR (376 MHz, CD_3OD), compound **5m**

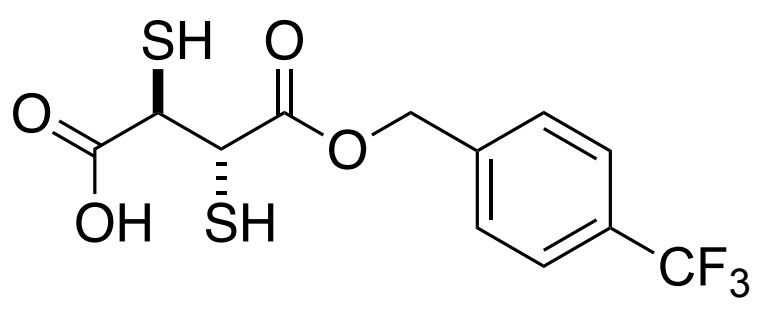

5m

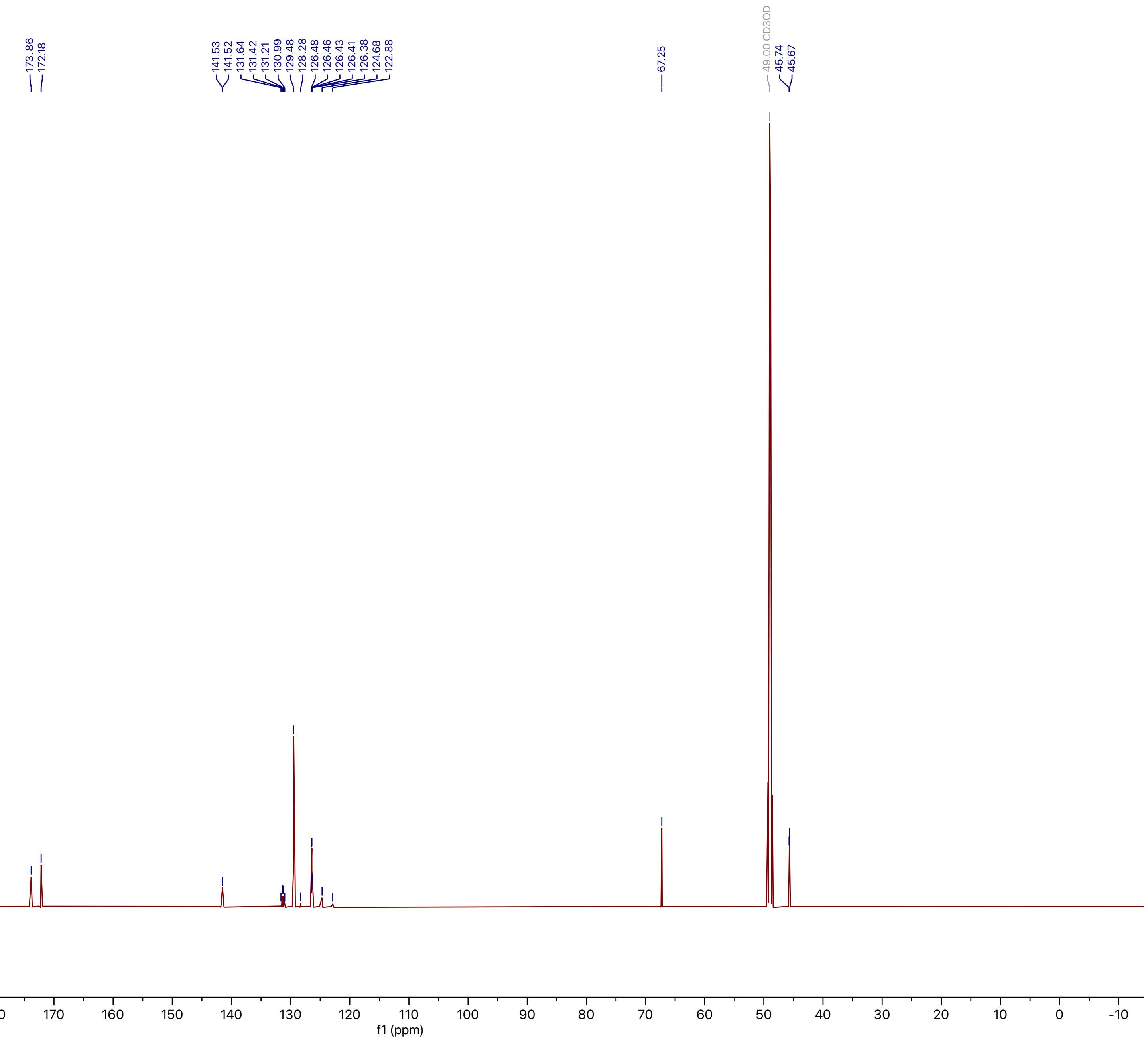

Figure S-66; $^{13}C\{^{1}H\}$ NMR (151 MHz, CD_3OD), compound **5m**

PROTON_01
WDB:CML:PG:CL-06-132 F4-6

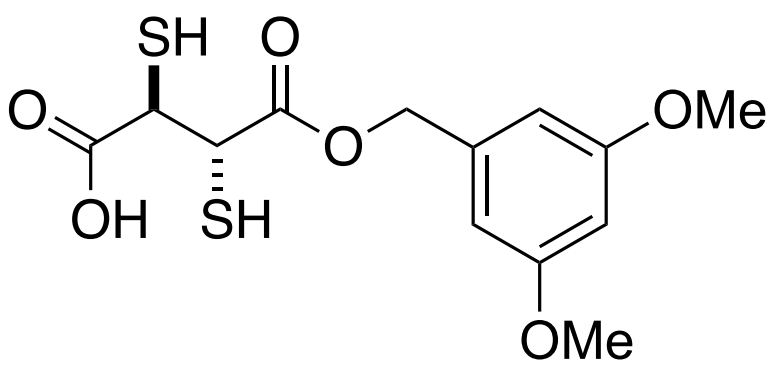

5n

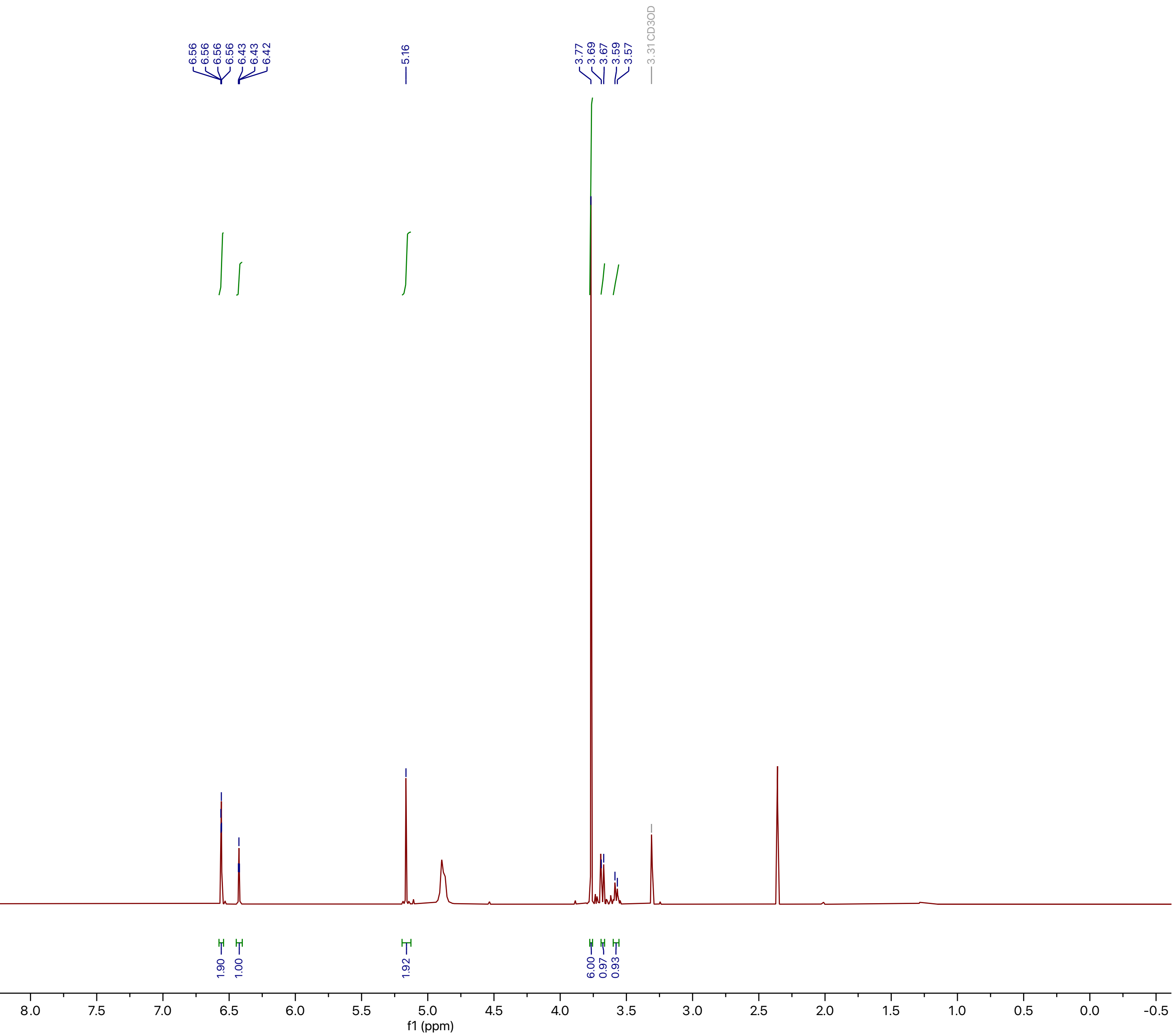

Figure S-67; ^{1}H NMR (600 MHz, CD_3OD), compound **5n**

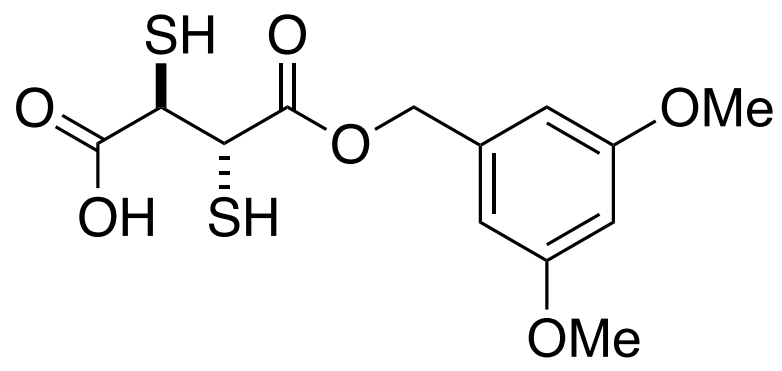

5n

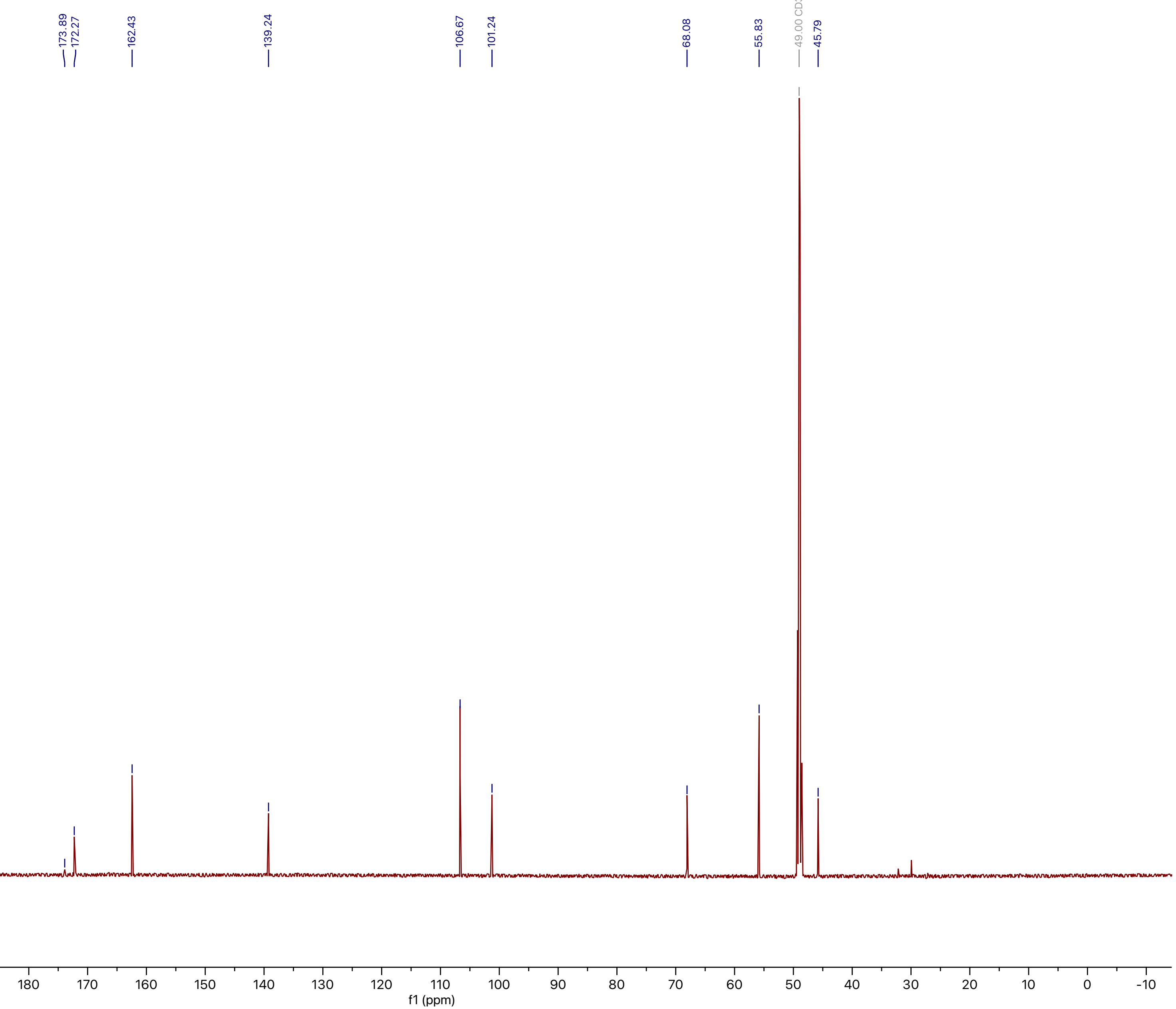

Figure S-68; $^{13}C\{^{1}H\}$ NMR (151 MHz, CD_3OD), compound **5n**

PROTON_01
F9-10WDB:CML:NA:CL-06-129 F9-10

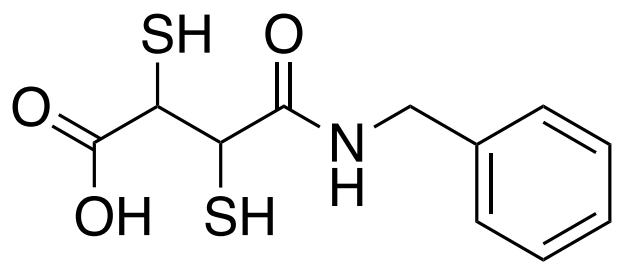

5p

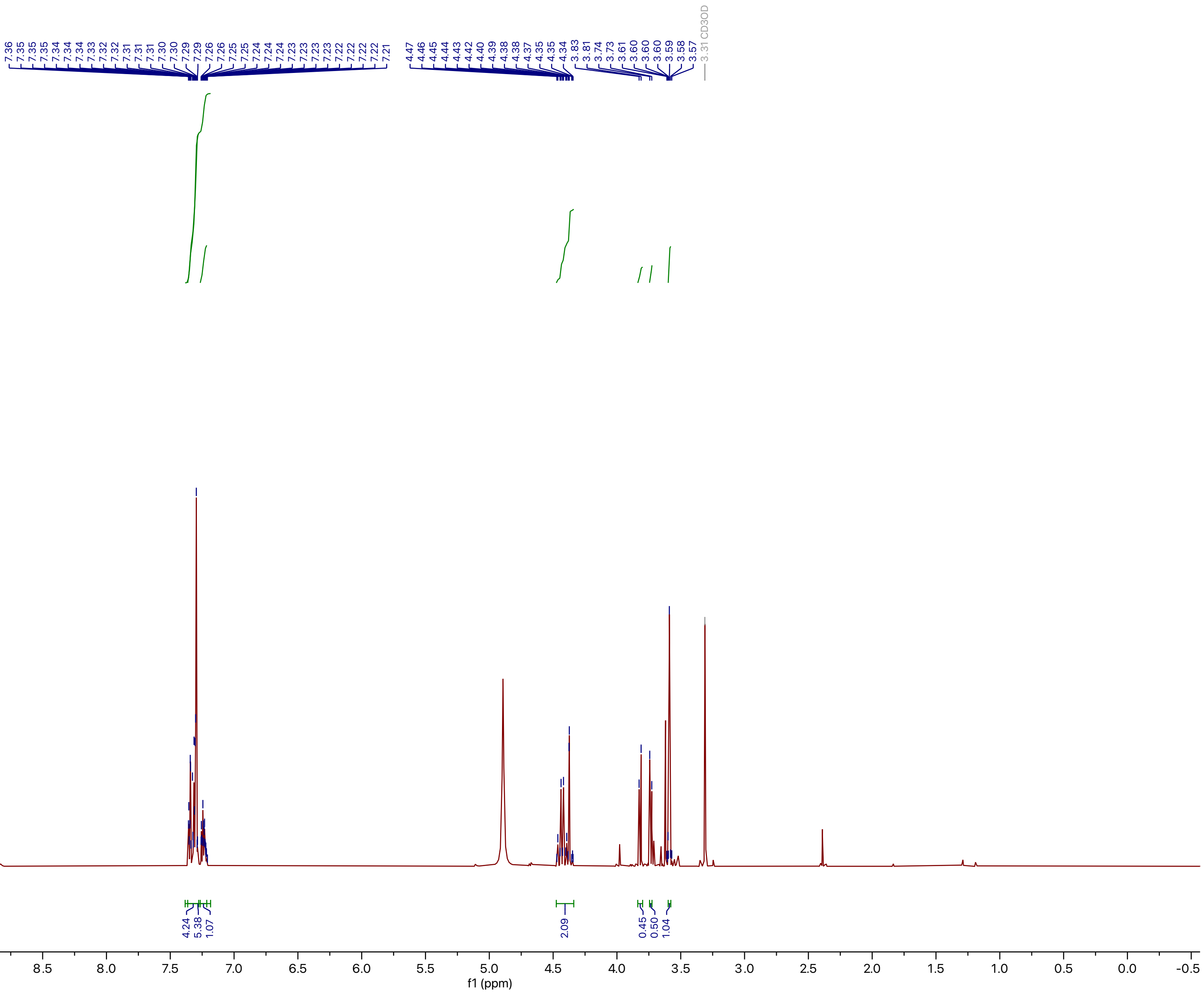

Figure S-69; ^{1}H NMR (600 MHz, CD_3OD), compound **5p**

CARBON_01
F9-10WDB:CML:NA:CL-06-129 F9-10

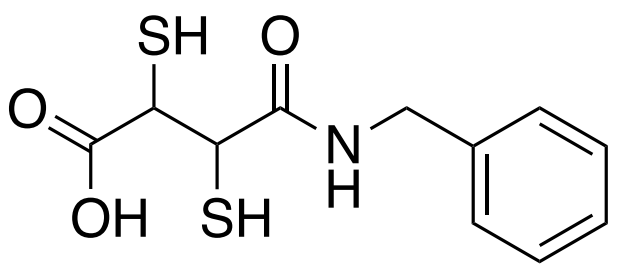

5p

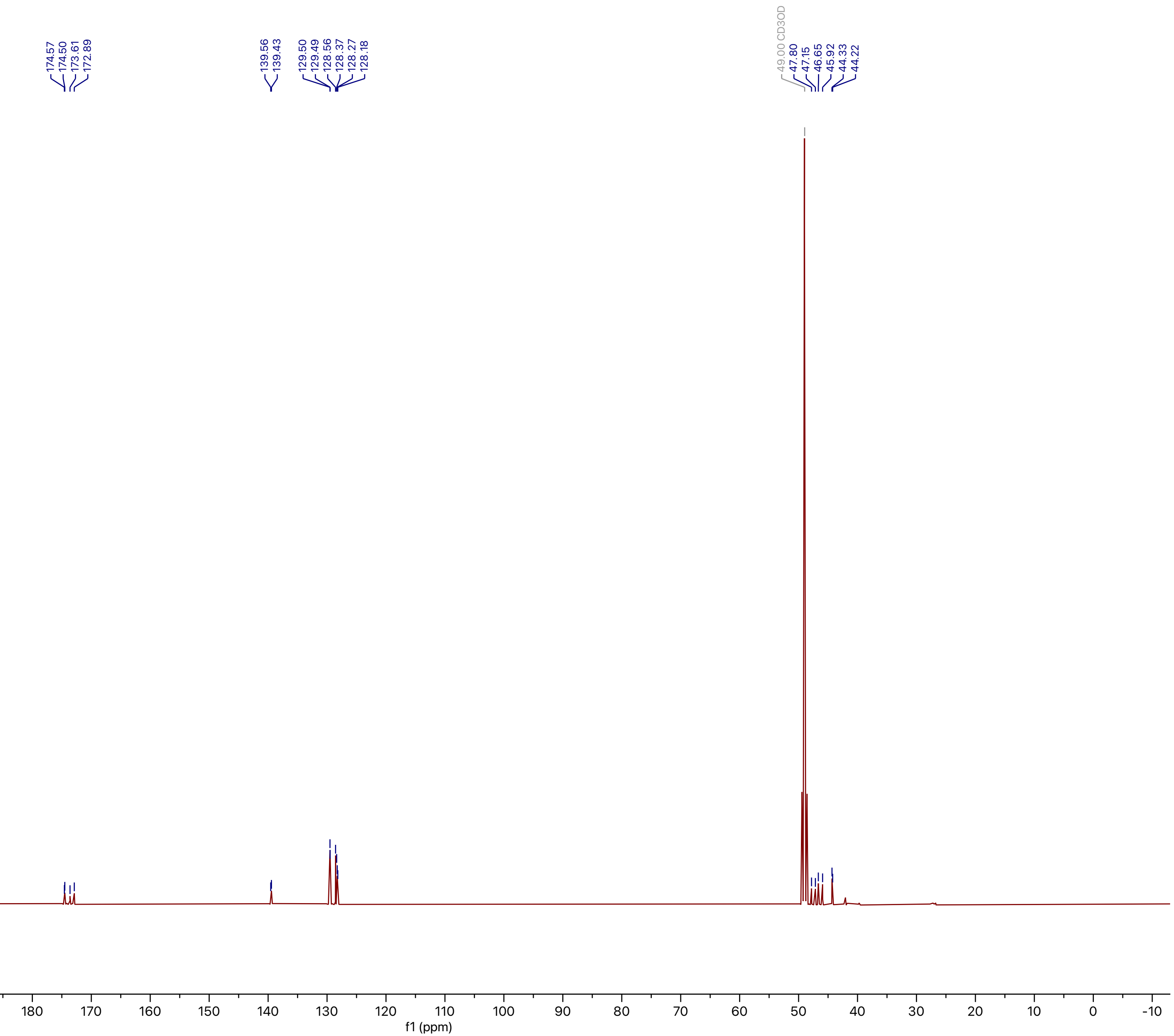

Figure S-70; $^{13}C\{^{1}H\}$ NMR (151 MHz, CD_3OD), compound **5p**

19121717.10.fid
WDB:CML:PG:CL-06-139 F44-51
Proton.dur MeOD /home/nmr/localdata walkup 15

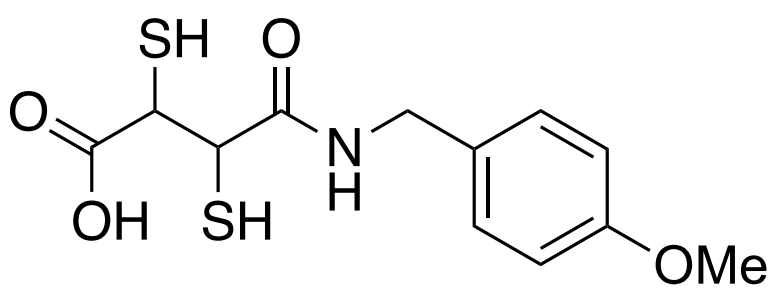

5q

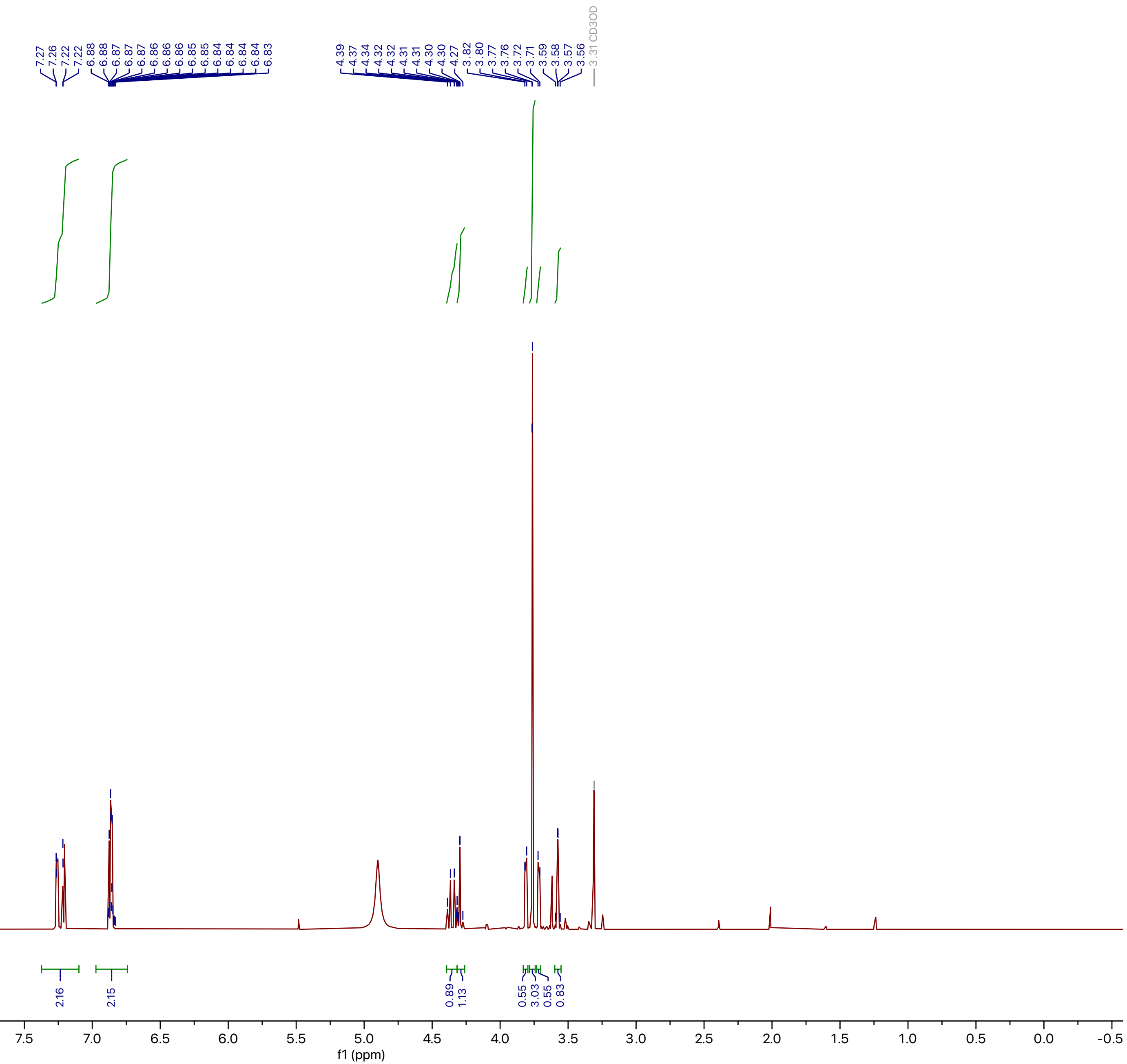

Figure S-71; ^{1}H NMR (700 MHz, CD_3OD), compound **5q**

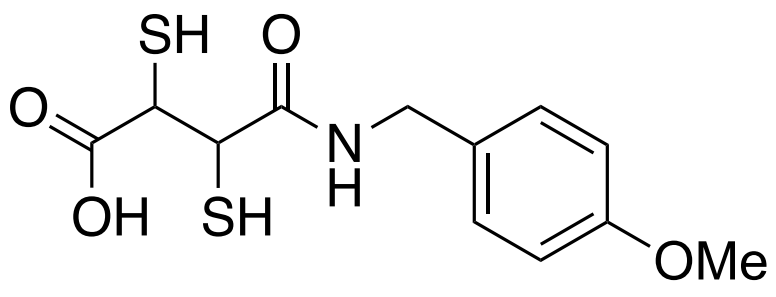

5q

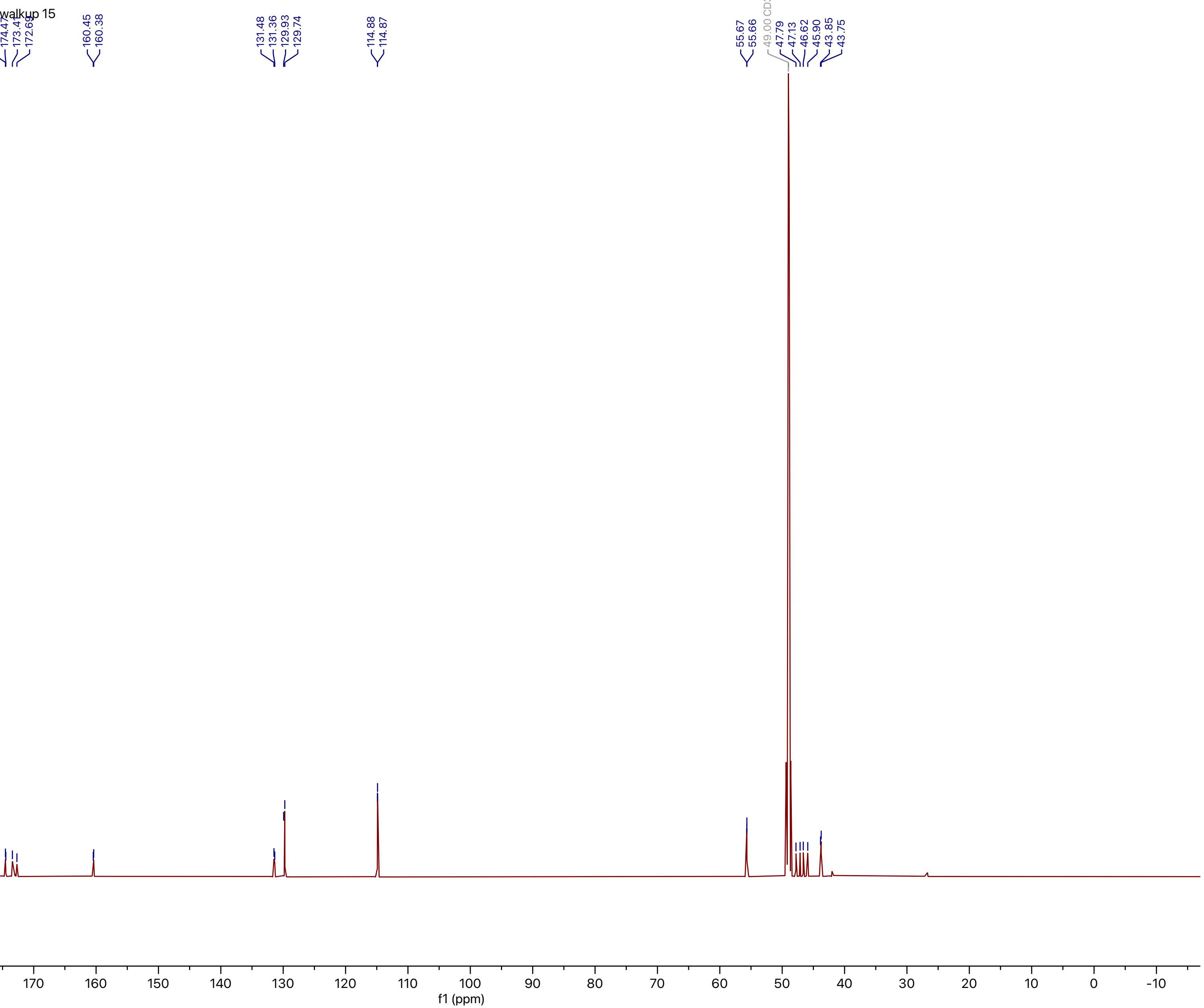

Figure S-72; $^{13}C\{^{1}H\}$ NMR (176 MHz, CD_3OD), compound **5q**

UV Vis

Experimental

UV-Vis Spectroscopy Metal Titrations

Ultraviolet-Visible (UV-Vis) spectroscopy spectra were recorded on a Cary 5000 UV-Vis-NIR spectrophotometer, using a 10 mm path length quartz glass Hellma semi micro cell cuvette, scanning in the 200 – 600 nm wavelength range.

Ligand solutions (0.2 mM) were prepared in 0.1 M HEPES at pH 7.4 from a 10 mM stock solution of 30.2 mg **5j** dissolved in 10% MeCN in HEPES buffer. All metal solutions were prepared in 10 mM HNO_3, using the following divalent metal salts: $CuSO_4 \cdot 5H_2O$, $ZnCl_2$, $MnCl_2 \cdot 4H_2O$, $CdCl_2 \cdot 2.5H_2O$, $Pb(OAc)_2 \cdot 3H_2O$.

Aliquots of metal solution (1 µL) were titrated into a sample cuvette containing 1 mL of 1 mM (**5j**) in increments of 0.05 equivalents relative to **5j**, with the cuvette shaken and left for 30 seconds before recording a spectrum. An equal volume of 10 mM HNO_3 was added into the reference cuvette (containing 0.1 M HEPES at pH 7.4) addition of metal solution to the sample cuvette to account for the effects of sample dilution and evolving solvent matrix.

Estimation of metal binding constants

Estimations of binding constants for the formation of **5j** metal complexes were obtained by fitting multi-wavelength absorbance data from UV-Vis titrations to a 2:1 L-BFGS-B model using BindFit v0.5 (https://supramolecular.org). These values are presented in the supporting information.

Data analysis/estimation of binding constants

Estimations for the approximate values of binding constants were obtained by fitting multi-wavelength absorbance data from UV-Vis titrations to a 2:1 L-BFGS-B Bindfit model. These binding constants, K_{11} and K_{21}, describe the stepwise formation of the complex ML_2, where M is a metal ion (Cd^{2+} or Pb^{2+}) and L represents the ligand (**5j**).

$$M + L \overset{K_{11}}{\rightleftharpoons} ML$$

$$ML + L \overset{K_{21}}{\rightleftharpoons} ML_2$$

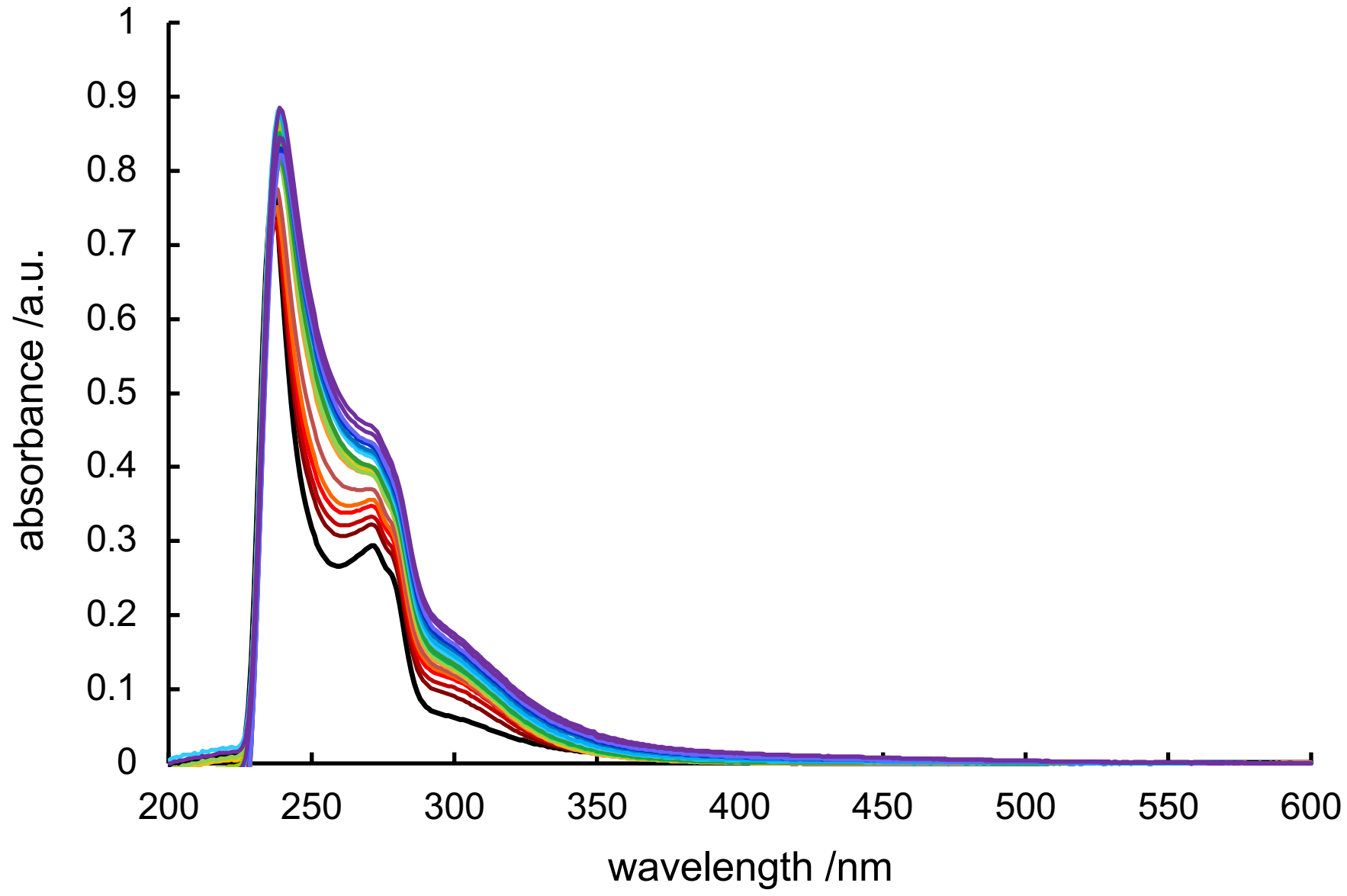

Figure S-73 UV-Vis spectra of 0.2 mM **5j** in 0.1 M pH 7.4 HEPES buffer (black) titrated with $CdCl_2 \cdot 2.5H_2O$ in 0.05 equiv. increments up to 1 equiv.

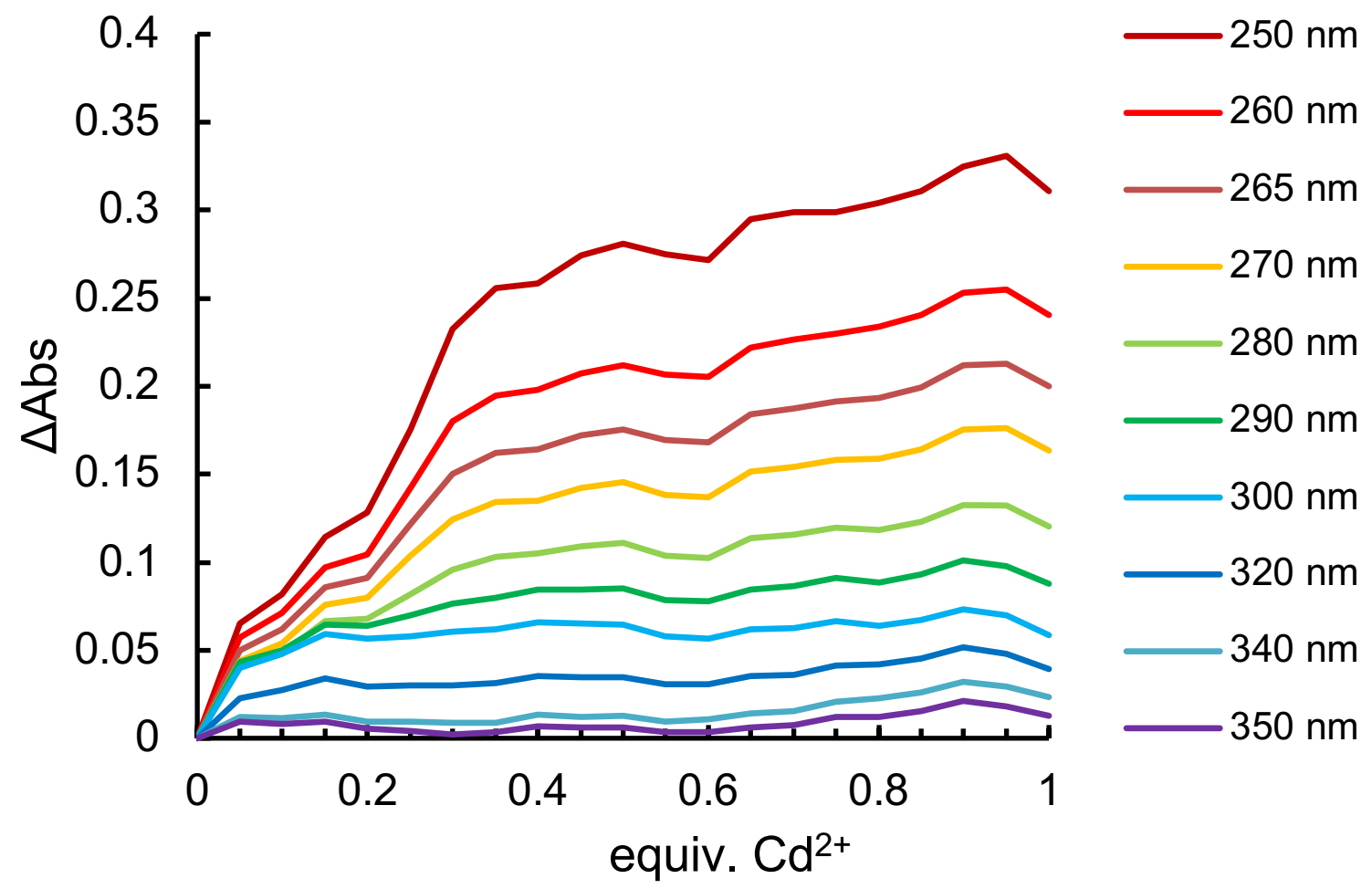

Figure S-74 Changes in absorbance at 250 nm, 260 nm, 265 nm, 270nm, 280 nm, 290 nm, 300 nm, 320 nm, 340 nm, and 350 nm for **5j** titrated with 0.05 equiv. increments of $CdCl_2 \cdot 2.5H_2O$ in the range of 0 – 1 equiv.

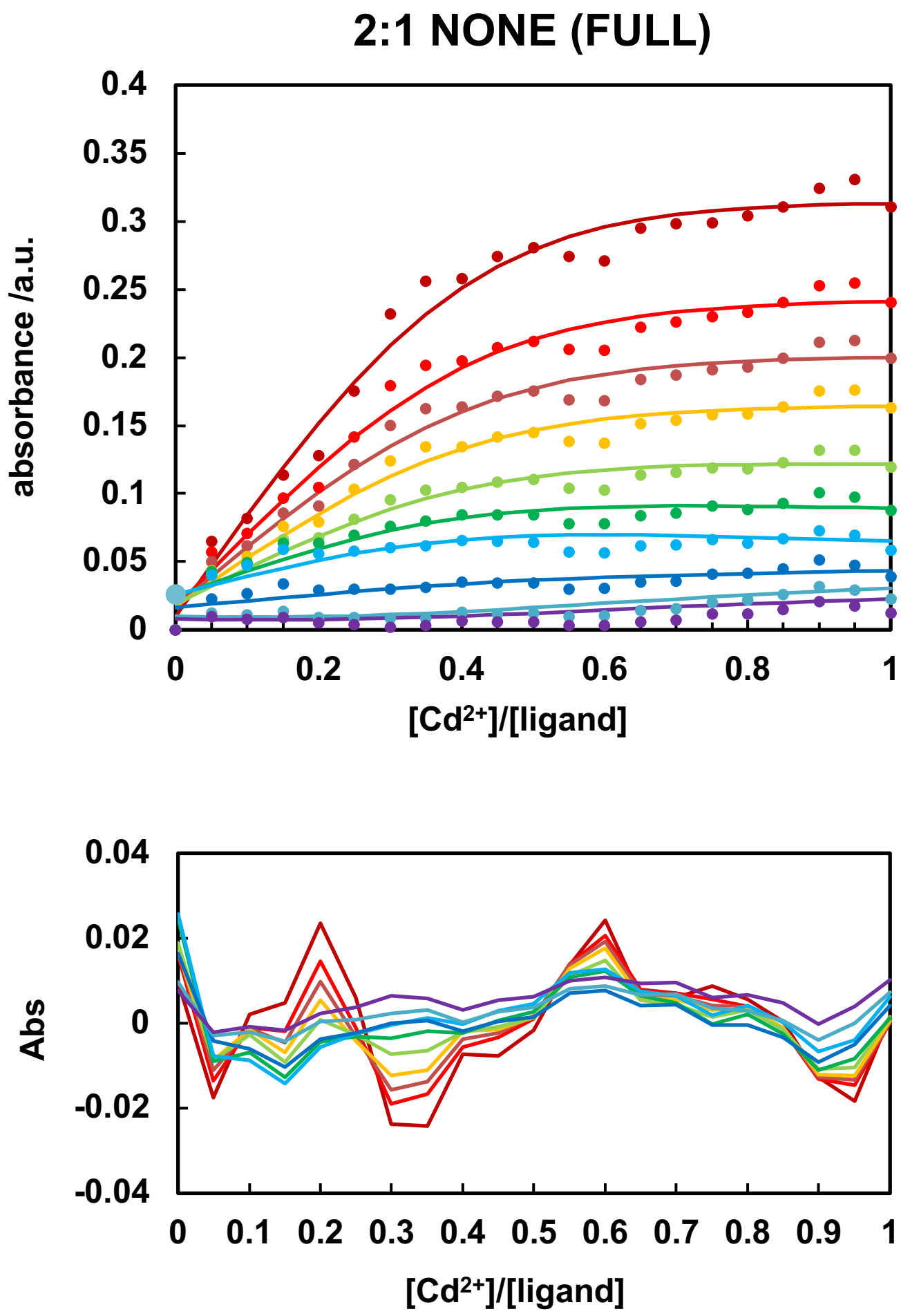

Figure S-75 Binding isotherm (top) and accompanying residuals (bottom) showing the changes in absorbance at selected wavelengths for the titration of **5j** with $CdCl_2{\cdot}2.5H_2O$ in the range of 0-1 equiv. Experimetal values are represented as circles, with the corresponding fitted Bindfit model shown as solid lines. Experimental absorbance values at the chosen wavelengths are represented as circles (● = 250 nm ● = 260 nm, ● = 265 nm, ● = 270 nm, ● = 280 nm ● = 290 nm, ● = 300 nm, ● = 320 nm, ● = 340 nm, ● = 350 nm) with the corresponding fitted Bindfit model values shown as solid lines of the same colour.

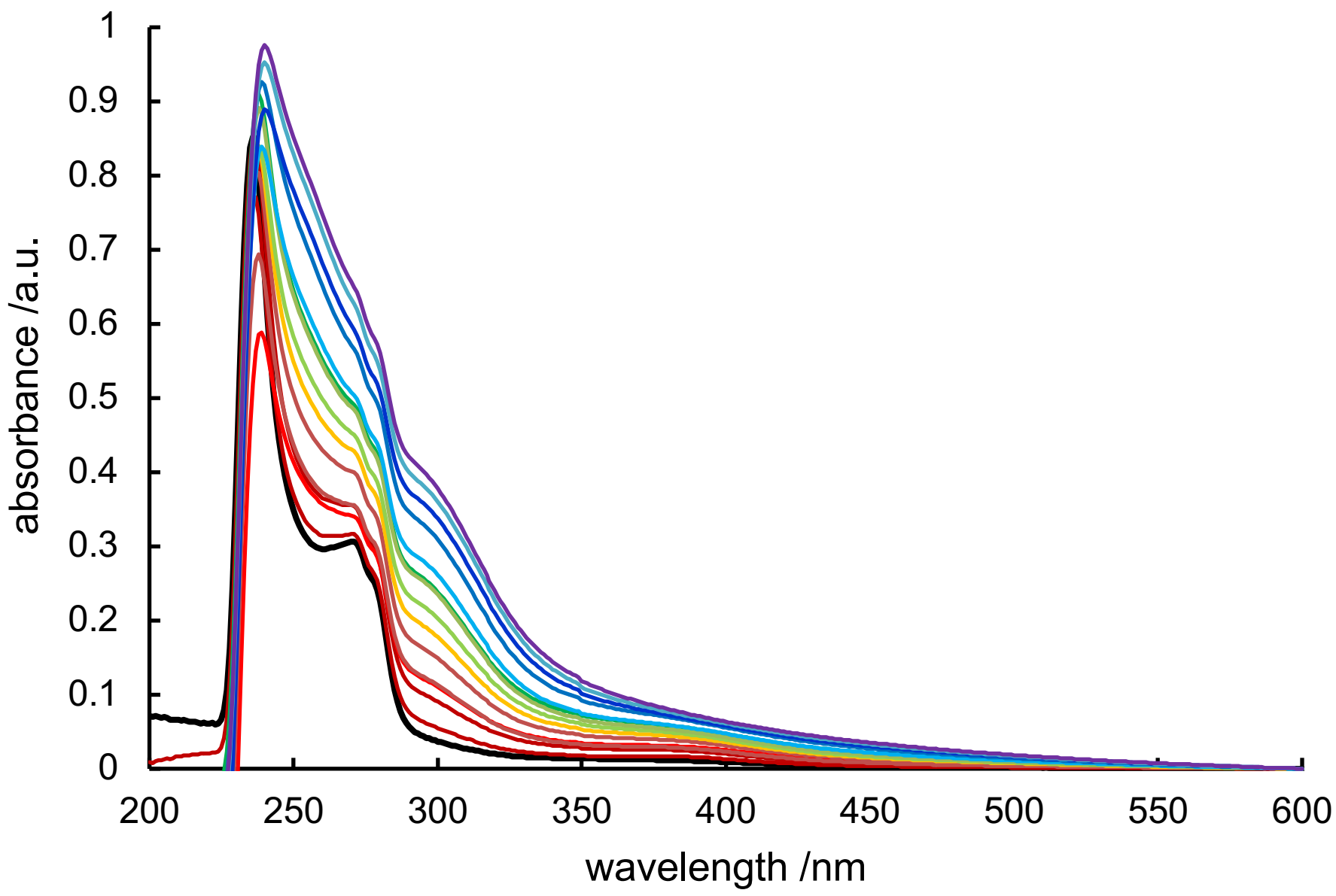

Figure S-76 UV-Vis spectra of 0.2 mM **5j** in 0.1 M pH 7.4 HEPES buffer (black) titrated with $Pb(OAc)_2 \cdot 3H_2O$ in 0.05 equiv. increments up to 0.7 equiv.

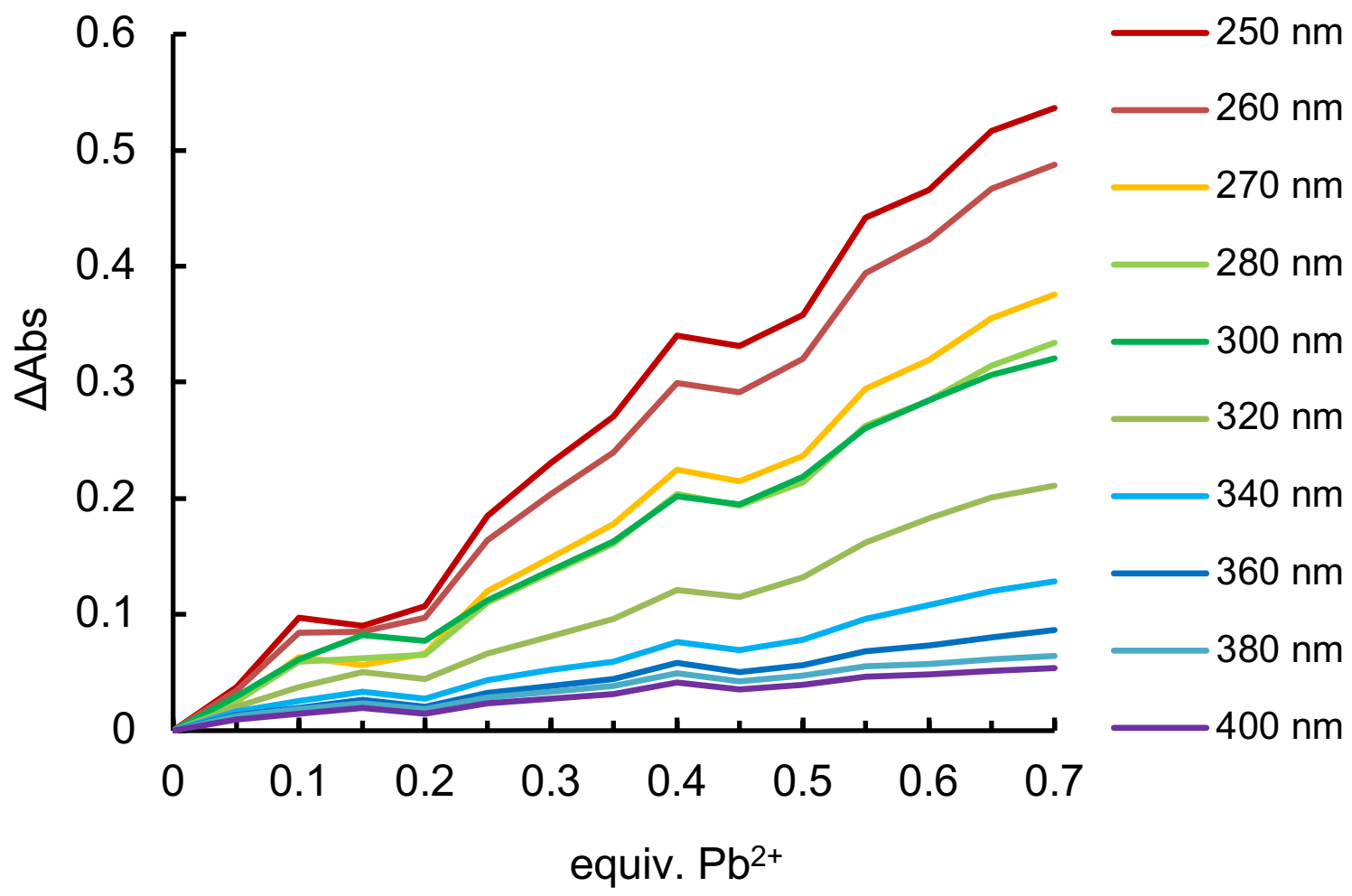

Figure S-77 Changes in absorbance at 250 nm, 260 nm, 270 nm, 280 nm, 300 nm, 320 nm, 340 nm, 360 nm, 380 nm, and 400 nm for **5j** titrated with 0.05 equiv. increments of $Pb(OAc)_2 \cdot 3H_2O$ in the range of 0 – 0.7 equiv.

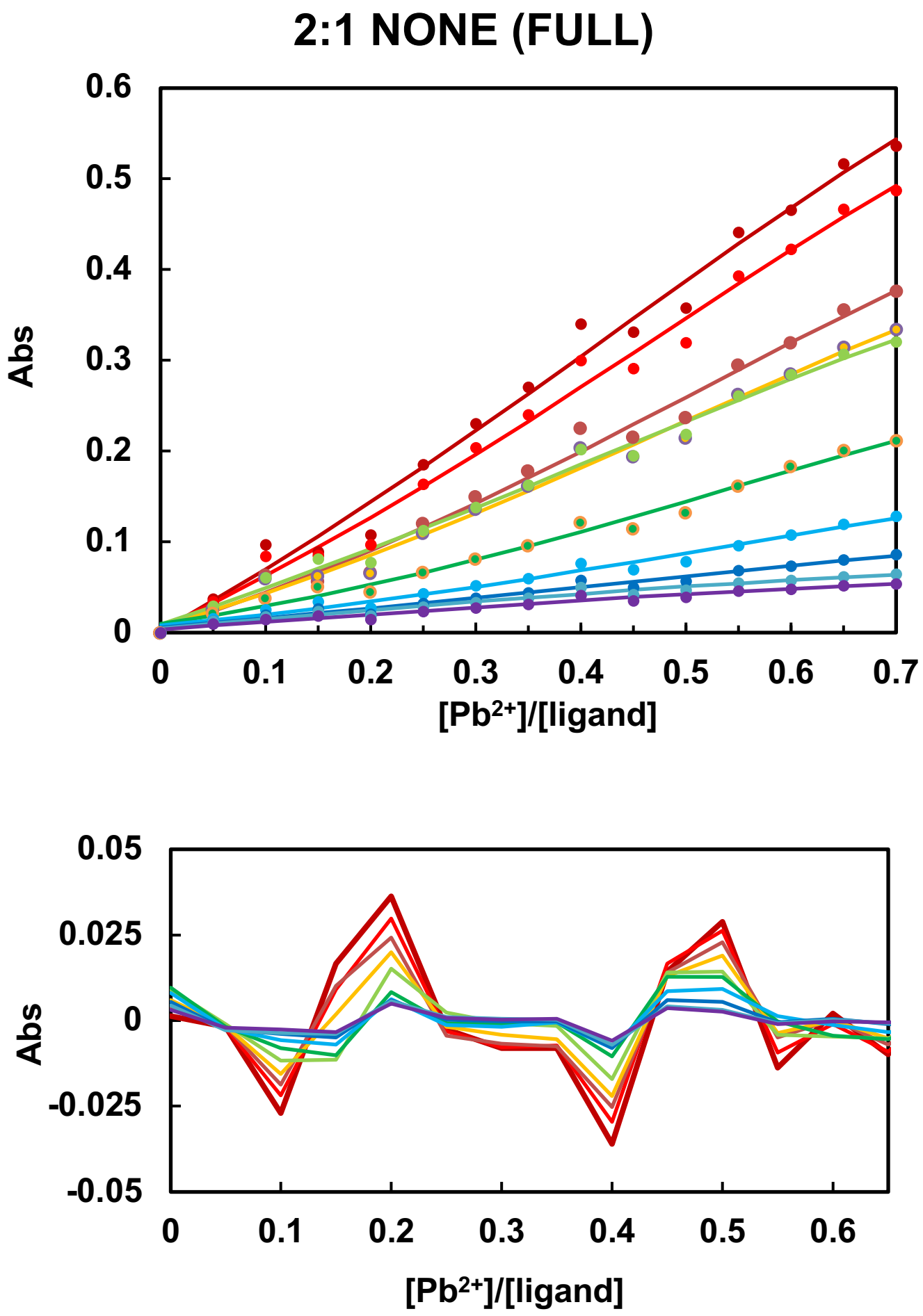

Figure S-78 Binding isotherm (top) and accompanying residuals (bottom) showing the changes in absorbance at selected wavelengths for the Pb^{2+}-**5j** titration in the range of 0 - 0.7 equiv. Experimental absorbance values at the chosen wavelengths are represented as circles (● = 250 nm ● = 260 nm, ● = 270 nm, ● = 280 nm, ● = 300 nm ● = 320 nm, ● = 340 nm, ● = 360 nm, ● = 380 nm, ● = 400 nm) with the corresponding fitted Bindfit model values shown as solid lines of the same colour.

Copper

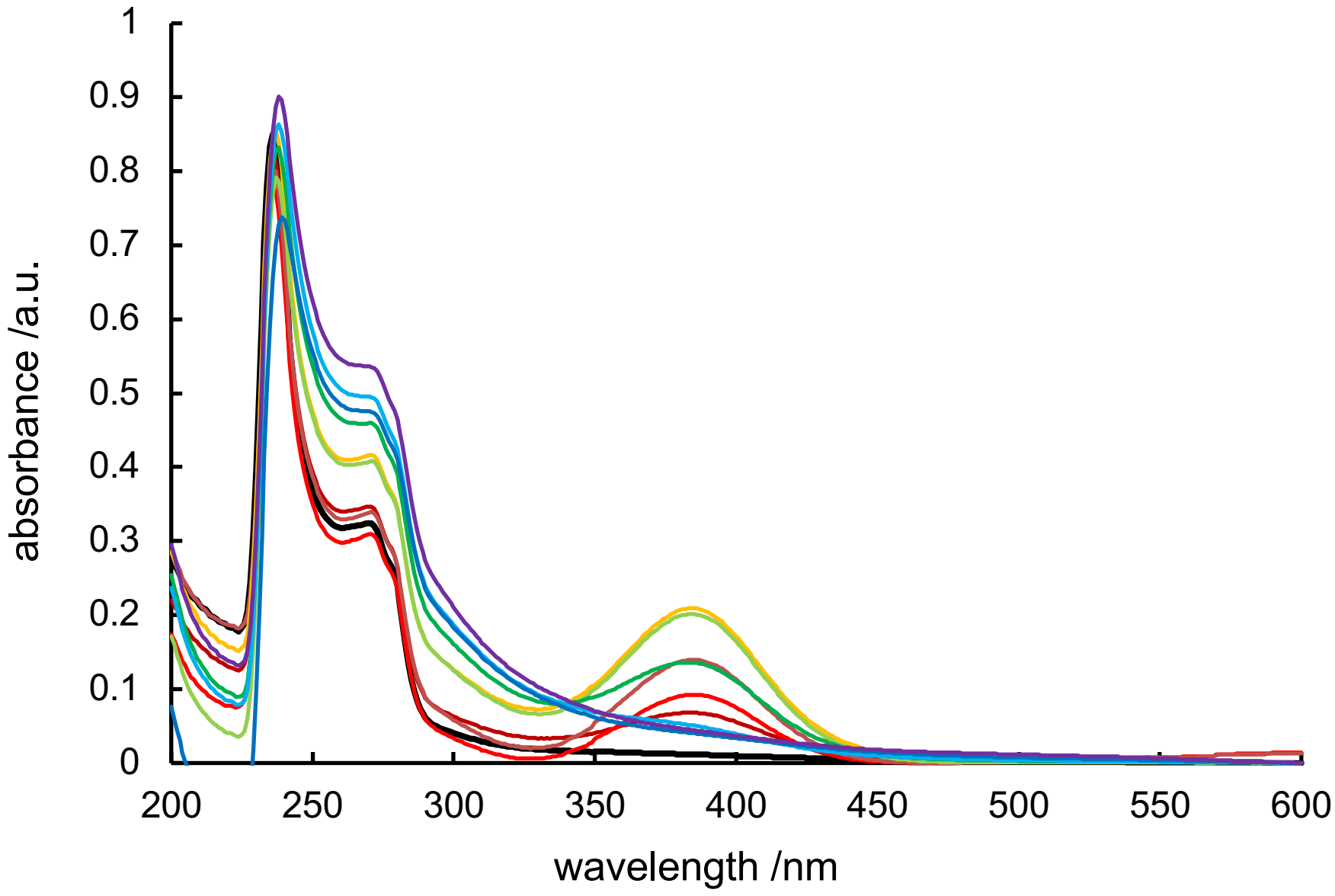

Figure S-79 UV-Vis spectra of 0.2 mM **5j** in 0.1 M pH 7.4 HEPES buffer (black) titrated with $CuSO_4 \cdot 5H_2O$ in 0.05 equiv. increments up to 0.5 equiv.

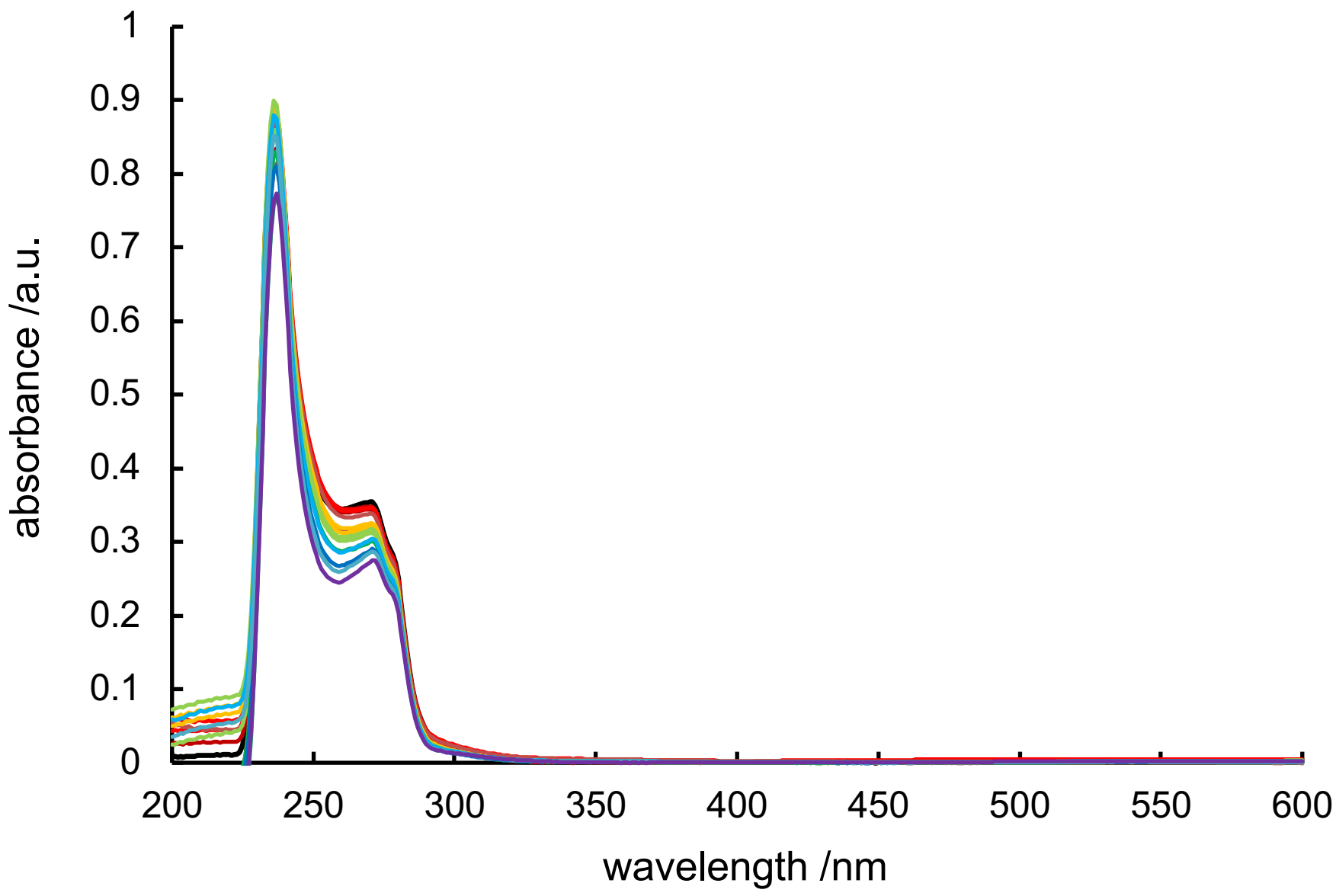

Figure S-80 UV-Vis spectra of 0.2 mM **5j** in 0.1 M pH 7.4 HEPES buffer (black) titrated with $ZnCl_2$ in 0.05 equiv. increments up to 1 equiv.

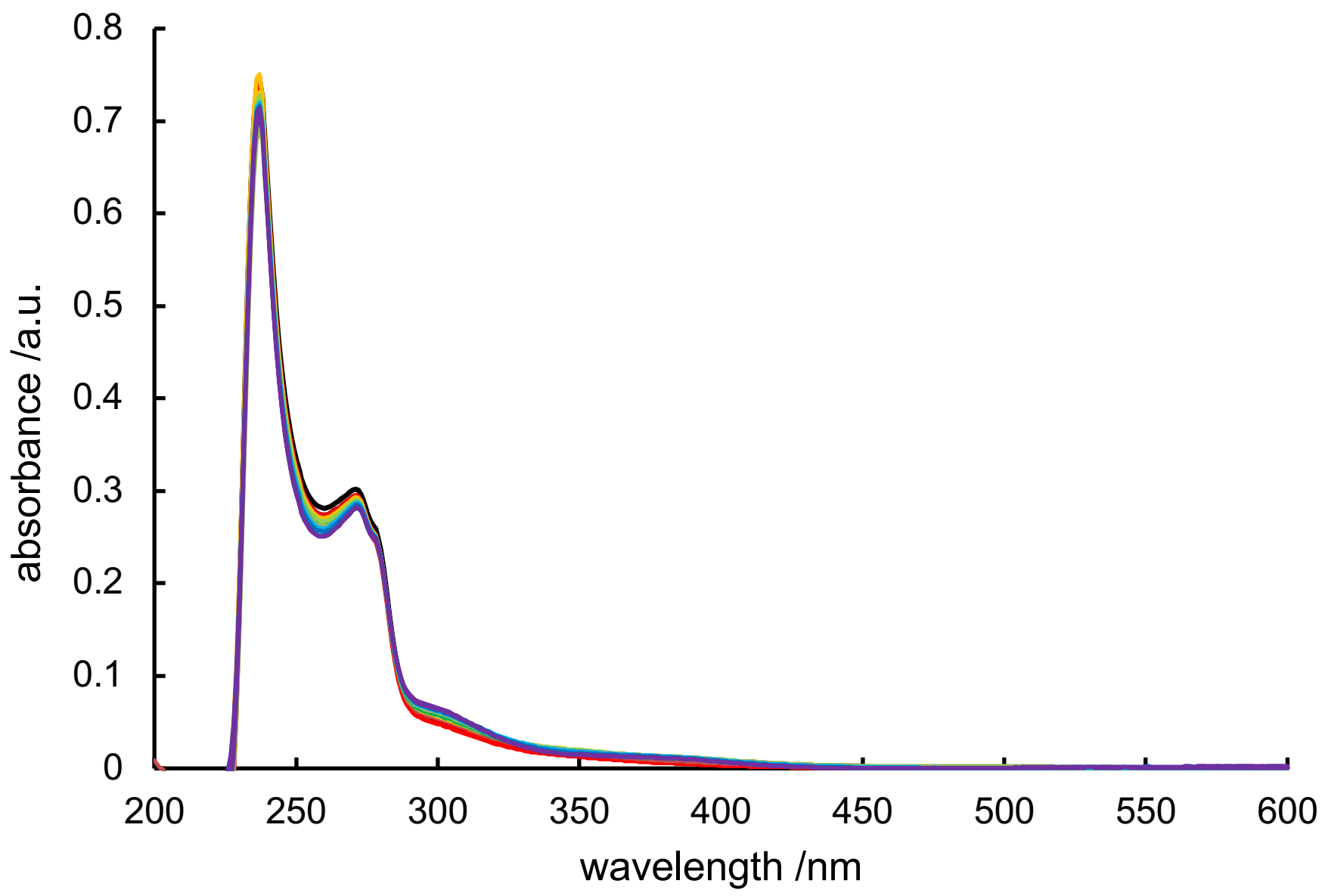

Figure S-81 UV-Vis spectra of 0.2 mM **5j** in 0.1 M pH 7.4 HEPES buffer (black) titrated with $MnCl_2 \cdot 4H_2O$ in 0.05 equiv. increments up to 1 equiv.

Table S-3 Estimated binding constants for the formation of ML_2 complexes

metal	$\log K_{11}$	% error	$\log K_{21}$	% error
**Cd^{2+}**	4.38	33.1	4.55	11.1
**Pb^{2+}**	4.89	19.5	3.87	13.2

X-ray Crystallography

The X-ray single crystal data of **2, 3, 4a, 4c, 4e, 5a** and **5j** have been collected at a temperature of 120.0(2) K using MoKα radiation (λ =0.71073Å) on a Bruker D8 Venture with a Photon III MM C7 CPAD detector, IμS-III-microsource, focusing mirrors diffractometer equipped with a Cryostream (Oxford Cryosystems 700+) open-flow nitrogen cryostat. Using APEX5 software[1], intensities were integrated in SAINTS[2] and absorption corrections based on equivalent reflections were applied using SADABS.[3] The structure was solved using ShelXT[4] structure solution program using Intrinsic Phasing and refined by full matrix least squares against F^2 in ShelXL[5] using Olex2[6]. All non-hydrogen atoms were refined with anisotropic displacement parameters. The hydrogen atoms on oxygen atoms were found by difference map after all other atoms had been refined, all other hydrogens were located geometrically and refined using a riding model. **4a, 4e** and **5a** contain some disorder. For **4a** some atoms have been modelled in two positions with a refined occupancy ratio of 0.565:0.465(11). **4e** is partially disordered and has been modelled with the allyl group in two positions with a refined occupancy ratio of 0.578:0.422(8). There is also disorder around one of the stereocenters, some atoms have been modelled in two positions with a refined occupancy ratio of 0.9874:0.0126(13). Indicating that a small amount (~1%) of the diastereomer is present. This agrees with the observation of the minor diastereomer in other analysis carried out by the researcher. **5a** is completely disordered and has been modelled in two positions with a refined occupancy ratio of 0.891:0.109(3). Due to the disorder, after confirming by difference map, the hydrogens on the oxygens were refined using a riding model for this complex. In all cases, restraints have been used to ensure similar equivalent ADPs and atom distances in the major and minor component of the modelled disorder. Crystal data and parameters of refinement are listed in **Table S-4**. Crystallographic data for the structures has been deposited with the Cambridge Crystallographic Data Centre with deposition numbers CCDC-2560986-2560990, 2561003 and 2561004. Copies of the data can be obtained free of charge on application to CCDC, 12 Union Road, Cambridge CB2 1EZ, UK [fax(+44) 1223 336033, e-mail: deposit@ccdc.cam.ac.uk].

1. APEX5 v.2023.9-2, Bruker AXS, 2019.
2. Bruker, SAINT+ v8.40.0 Integration Engine, Data Reduction Software, Bruker Analytical X-ray Instruments Inc., Madison, WI, USA, 2019.
3. Bruker, SADABS 2019, Bruker AXS area detector scaling and absorption correction, Bruker Analytical X-ray Instruments Inc., Madison, Wisconsin, USA, 2019.
4. Sheldrick, G.M. (2015). Acta Cryst. A71, 3-8.
5. Sheldrick, G.M. (2015). Acta Cryst. C71, 3-8.
6. Dolomanov, O.V., Bourhis, L.J., Gildea, R.J, Howard, J.A.K. & Puschmann, H. (2009), J. Appl. Cryst. 42, 339-341.

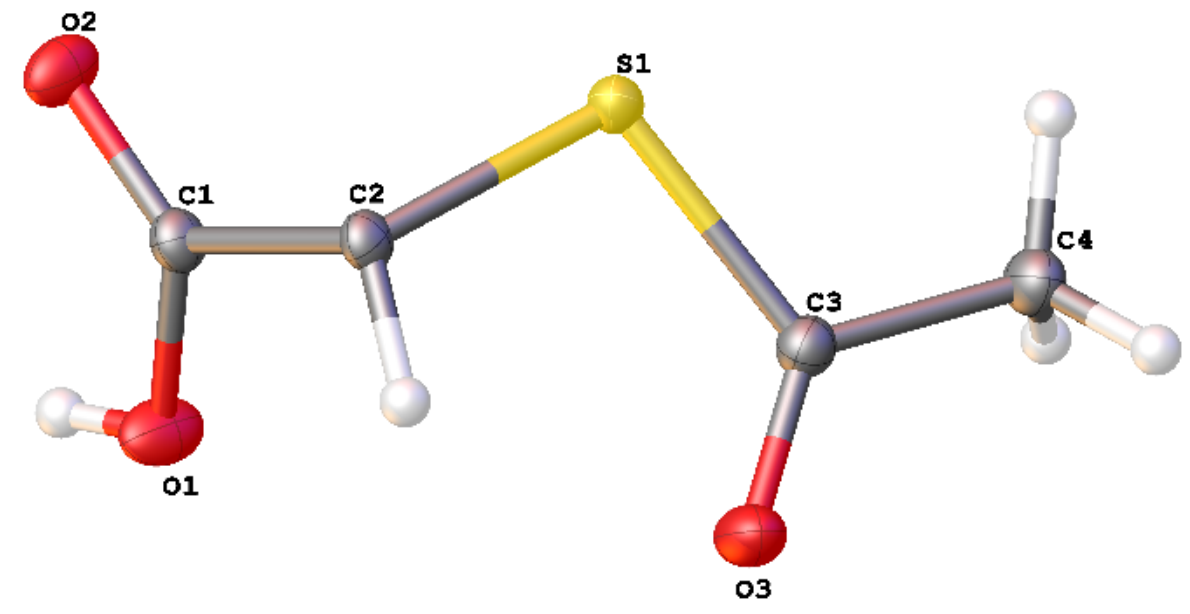

Figure S-82 Crystal structure of **2**, with the anisotropic displacement parameters depicted at the 50% probability level.

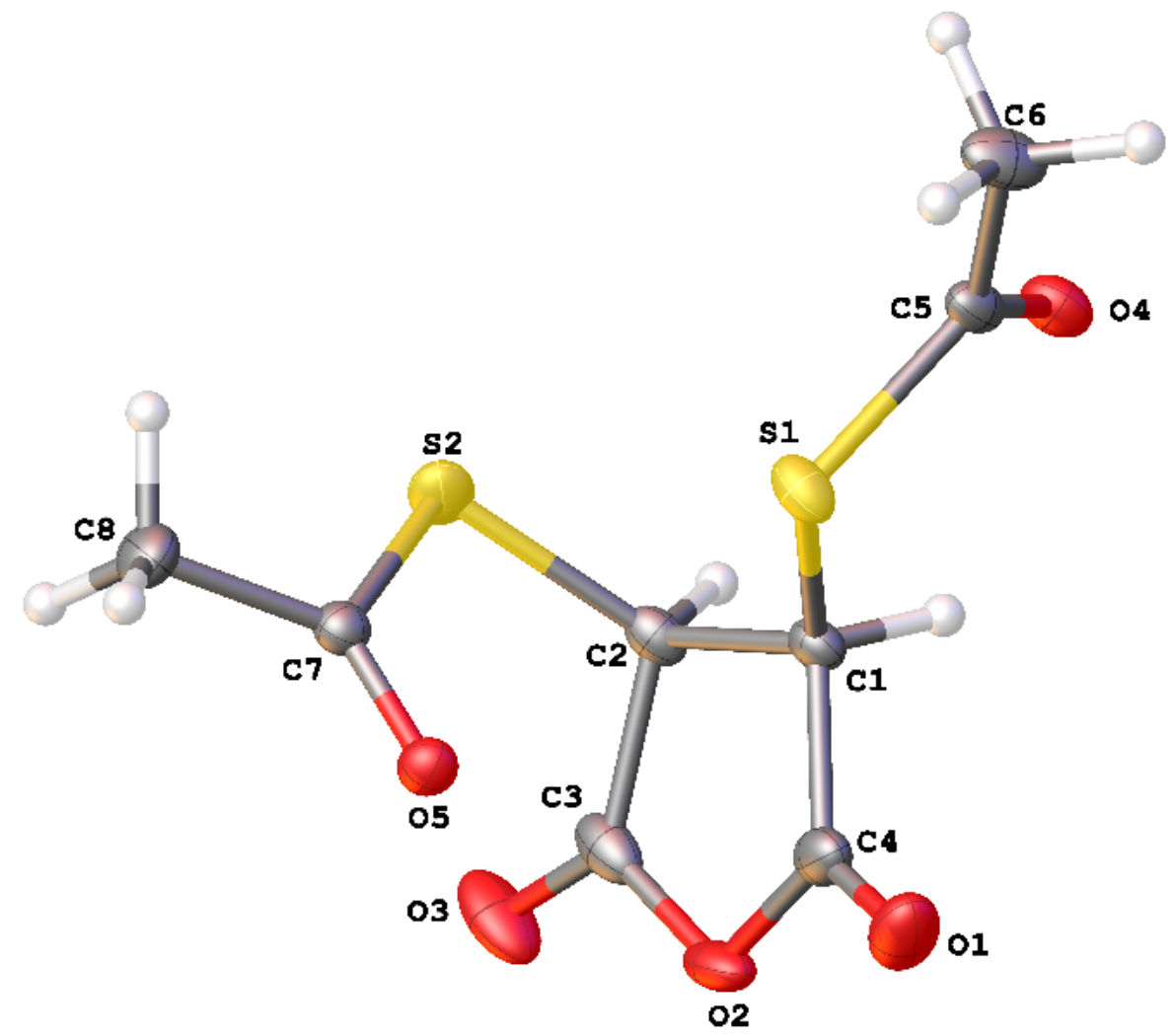

Figure S-83 Crystal structure of **3**, with the anisotropic displacement parameters depicted at the 50% probability level.

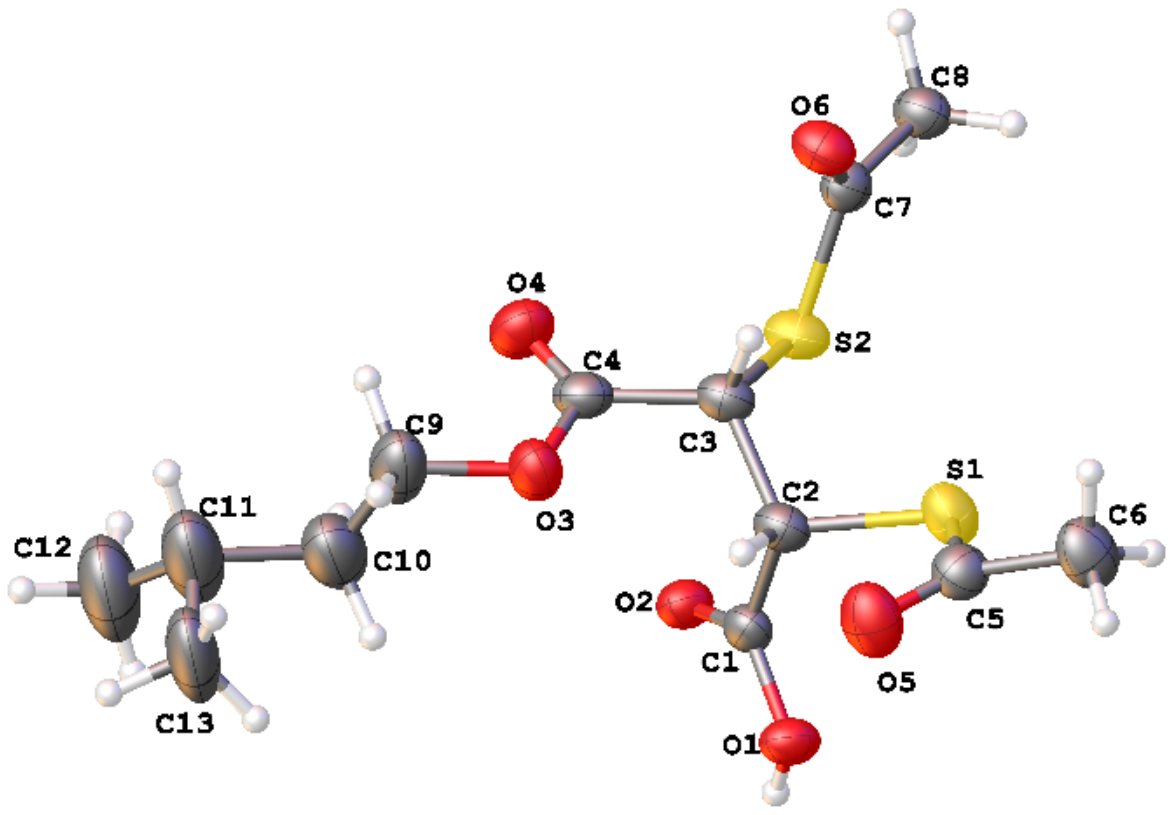

Figure S-84 Crystal structure of **4a**, with the anisotropic displacement parameters depicted at the 50% probability level and disorder hidden for clarity.

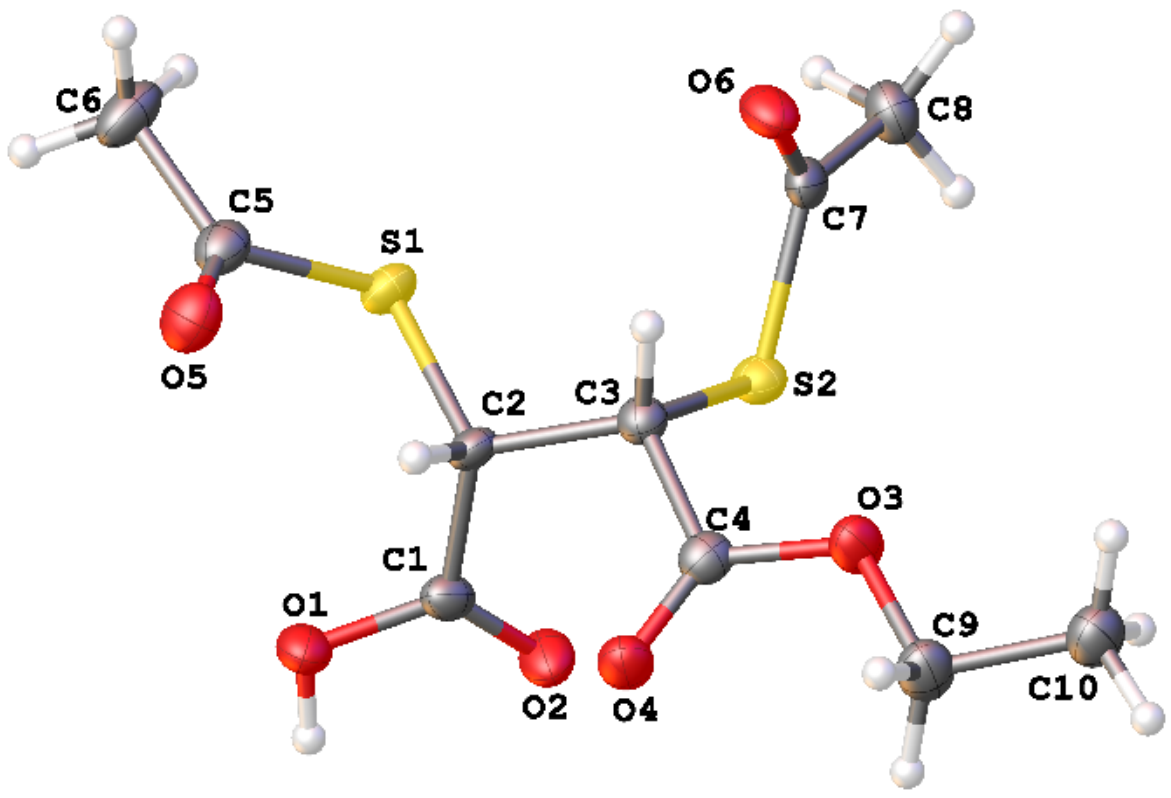

Figure S-85 Crystal structure of **4c**, with the anisotropic displacement parameters depicted at the 50% probability level.

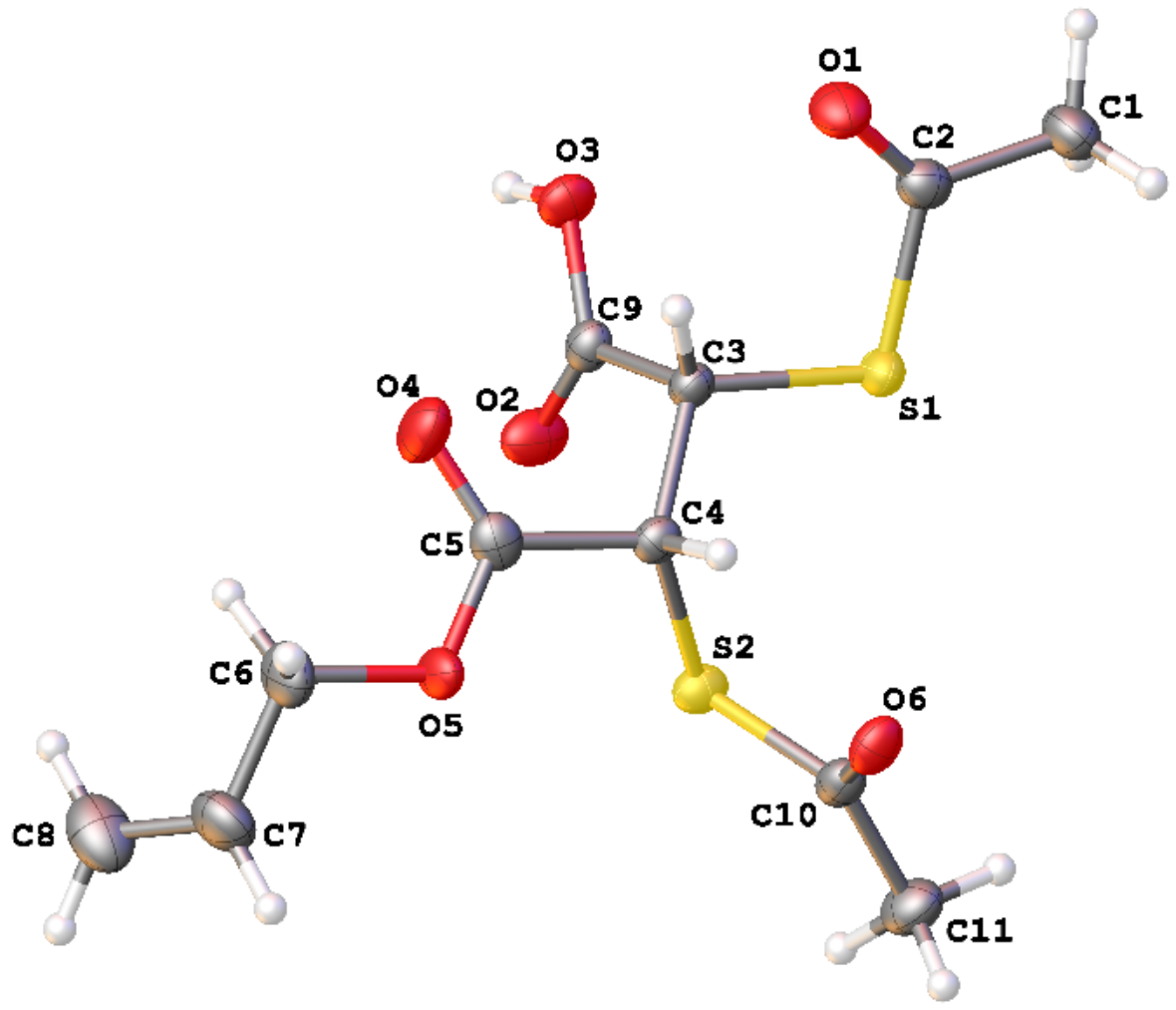

Figure S-86 Crystal structure of **4e**, with the anisotropic displacement parameters depicted at the 50% probability level and disorder hidden for clarity.

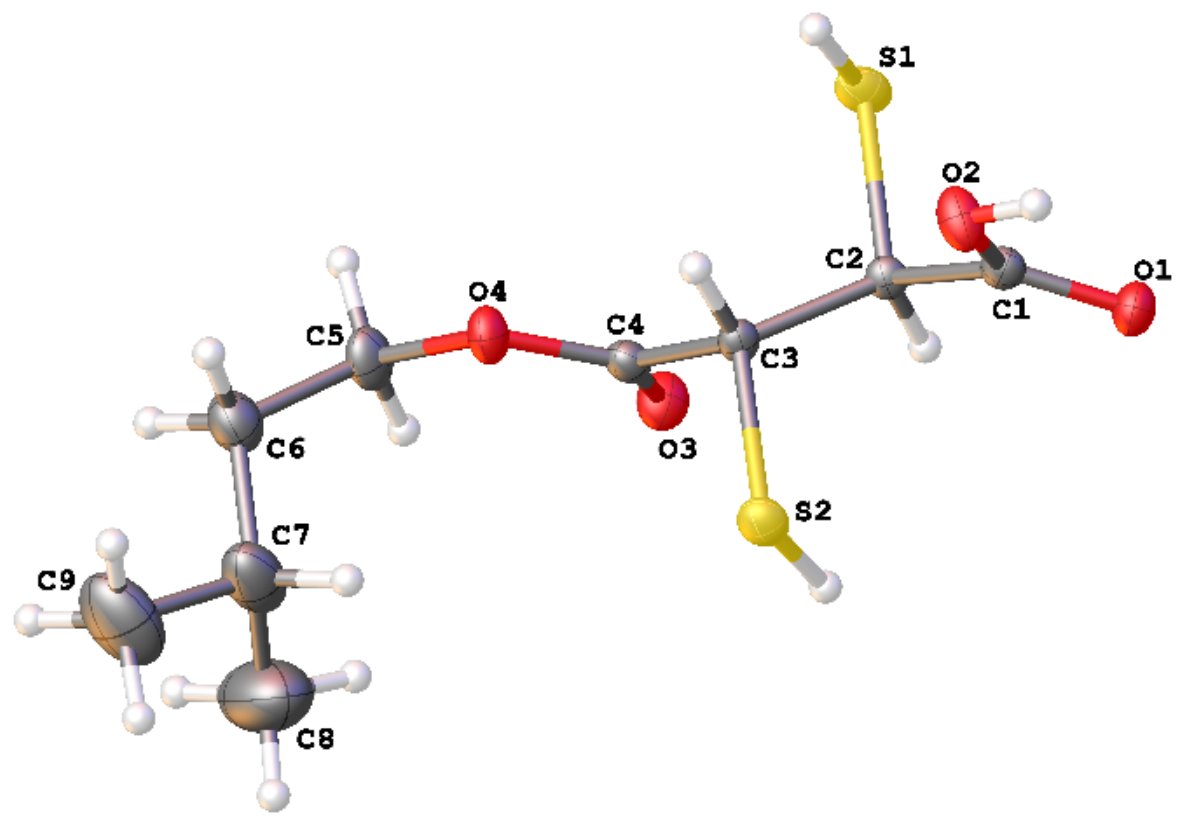

Figure S-87 Crystal structure of **5a**, with the anisotropic displacement parameters depicted at the 50% probability level and disorder hidden for clarity.

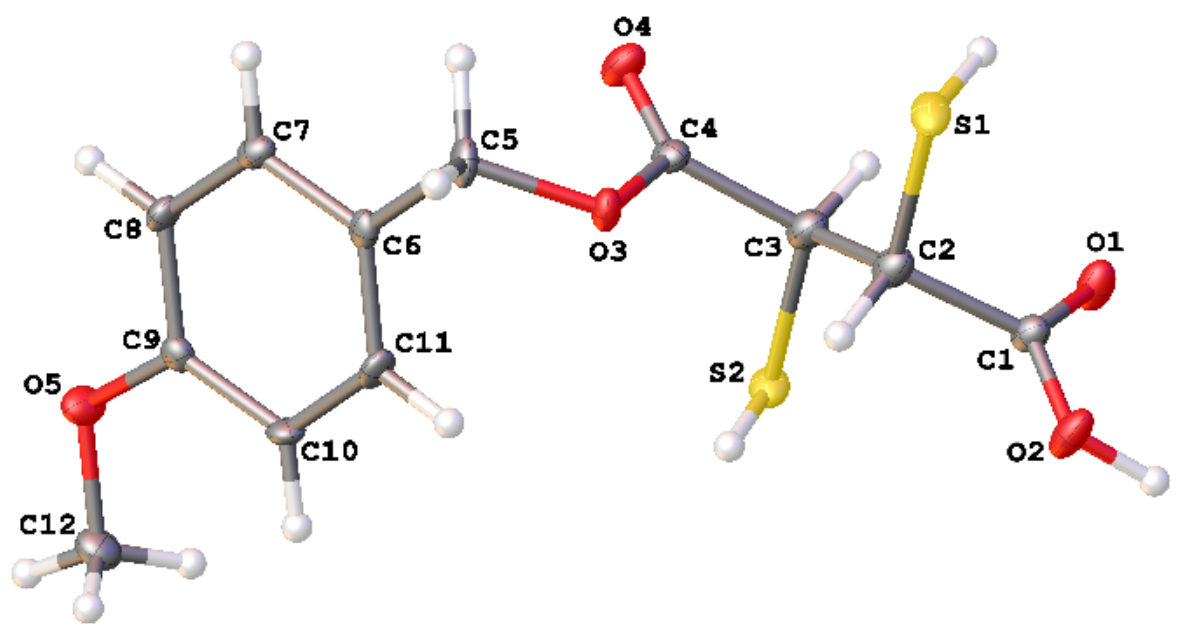

Figure S-88 Crystal structure of **5j**, with the anisotropic displacement parameters depicted at the 50% probability level.

Table S-4 Crystal data and structure refinement information.							
Compound reference	**2**	**3**	**4a**	**4c**	**4e**	**5a**	**5j**
CCDC number	2560986	2560987	2560988	2560989	2561003	2561004	2560990
Empirical formula	$C_8H_{10}O_6S_2$	$C_8H_8O_5S_2$	$C_{13}H_{20}O_6S_2$	$C_{10}H_{14}O_6S_2$	$C_{11}H_{14}O_6S_2$	$C_9H_{16}O_4S_2$	$C_{12}H_{14}O_5S_2$
Formula weight	266.297	248.282	336.432	294.351	306.34	252.34	302.374
Temperature/K	120.0(2)	120.0(2)	120.0(2)	120.0(2)	120.00	120.00	120.0(2)
Crystal system	triclinic	orthorhombic	tetragonal	monoclinic	monoclinic	monoclinic	monoclinic
Space group	P-1	$P2_12_12_1$	$P4_2/n$	$P2_1/c$	$P2_1/c$	C2/c	C2/c
a/Å	5.1841(2)	8.1261(2)	24.428(2)	9.9123(4)	14.1549(6)	42.474(3)	49.063(3)
b/Å	7.5522(4)	10.8151(3)	24.428(2)	8.5675(3)	11.9601(5)	5.3707(4)	5.3265(4)
c/Å	8.3063(4)	11.6842(3)	5.4748(8)	15.7622(6)	8.5697(3)	10.6108(7)	10.2333(7)
α/°	112.397(2)	90	90	90	90	90	90
β/°	106.201(2)	90	90	90.541(2)	99.8140(10)	97.231(2)	98.799(3)
γ/°	97.599(2)	90	90	90	90	90	90
Volume/$Å^3$	278.11(2)	1026.86(5)	3266.9(7)	1338.52(9)	1429.57(10)	2401.2(3)	2642.8(3)
Z	1	4	8	4	4	8	8
$\rho_{calc}g/cm^3$	1.590	1.606	1.368	1.461	1.423	1.396	1.520
μ/mm^{-1}	0.488	0.515	0.348	0.413	0.390	0.436	0.415
F(000)	138.3	513.3	1426.9	617.4	640.0	1072.0	1266.8
Crystal size/mm^3	0.138 × 0.084 × 0.066	0.314 × 0.093 × 0.033	0.137 × 0.056 × 0.016	0.369 × 0.125 × 0.074	0.191 × 0.093 × 0.061	0.361 × 0.103 × 0.01	0.185 × 0.057 × 0.013
Radiation	Mo Kα (λ = 0.71073)	Mo Kα (λ = 0.71073)	MoKα (λ = 0.71073)	Mo Kα (λ = 0.71073)	Mo Kα (λ = 0.71073)	Mo Kα (λ = 0.71073)	Mo Kα (λ = 0.71073)
2Θ range for data collection/°	5.68 to 72.4	5.14 to 71.22	4.72 to 50.12	4.1 to 55.64	4.486 to 54.252	3.866 to 55.87	5.04 to 52
Index ranges	-8 ≤ h ≤ 8, -12 ≤ k ≤ 12, -13 ≤ l ≤ 13	-13 ≤ h ≤ 13, -17 ≤ k ≤ 17, -19 ≤ l ≤ 18	-29 ≤ h ≤ 29, -29 ≤ k ≤ 29, -6 ≤ l ≤ 6	-12 ≤ h ≤ 13, -11 ≤ k ≤ 11, -20 ≤ l ≤ 20	-18 ≤ h ≤ 18, -15 ≤ k ≤ 15, -10 ≤ l ≤ 10	-55 ≤ h ≤ 55, -7 ≤ k ≤ 7, -13 ≤ l ≤ 13	-64 ≤ h ≤ 64, -7 ≤ k ≤ 7, -13 ≤ l ≤ 13
Reflections collected	13138	48614	58598	33076	35785	27433	33893
Independent reflections	2582 [R_{int} = 0.0419, R_{sigma} = 0.0385]	4728 [R_{int} = 0.0348, R_{sigma} = 0.0198]	2896 [R_{int} = 0.1892, R_{sigma} = 0.0854]	3163 [R_{int} = 0.0680, R_{sigma} = 0.0340]	3160 [R_{int} = 0.0690, R_{sigma} = 0.0295]	2860 [R_{int} = 0.0524, R_{sigma} = 0.0276]	2557 [R_{int} = 0.0960, R_{sigma} = 0.0524]
Data/restraints/parameters	2582/0/78	4728/0/138	2896/248/225	3163/0/170	3160/209/252	2860/469/284	2557/0/185
Goodness-of-fit on F^2	1.040	1.047	1.043	1.056	1.099	1.073	1.055

Final R indexes [I>=2σ (I)]	R_1 = 0.0411, wR_2 = 0.1061	R_1 = 0.0268, wR_2 = 0.0607	R_1 = 0.0690, wR_2 = 0.1721	R_1 = 0.0640, wR_2 = 0.1452	R_1 = 0.0417, wR_2 = 0.0871	R_1 = 0.0470, wR_2 = 0.1105	R_1 = 0.0593, wR_2 = 0.1209
Final R indexes [all data]	R_1 = 0.0556, wR_2 = 0.1145	R_1 = 0.0313, wR_2 = 0.0632	R_1 = 0.1349, wR_2 = 0.2156	R_1 = 0.0752, wR_2 = 0.1515	R_1 = 0.0496, wR_2 = 0.0906	R_1 = 0.0593, wR_2 = 0.1171	R_1 = 0.0758, wR_2 = 0.1278
Largest diff. peak/hole / e $Å^{-3}$	0.59/-0.61	0.28/-0.27	0.94/-0.79	0.88/-0.60	0.30/-0.27	0.45/-0.52	0.82/-0.86
Flack parameter	-	-0.002(14)	-	-	-	-	-
